# MagT1 regulated the odontogenic differentiation of BMMSCs induced byTGC-CM via ERK signaling pathway

**DOI:** 10.1186/s13287-019-1148-6

**Published:** 2019-01-31

**Authors:** Jian-mao Zheng, Yuan-yuan Kong, Yao-yin Li, Wen Zhang

**Affiliations:** 1 Department of Operative Dentistry and Endodontics, Guanghua School of Stomatology, Affiliated Stomatological Hospital, Sun Yat-sen University, Guangzhou, 510055 Guangdong China; 2 Guangdong Provincial Key Laboratory of Stomatology, Sun Yat-sen University, Guangzhou, Guangdong China; 3 Key Laboratory of Oral Medicine, Guangzhou Institute of Oral Disease, Stomatology Hospital of Guangzhou Medical University, Guangzhou, Guangdong China; 4 Department of Endodontics, Stomatology Hospital of Guangzhou Medical University, Guangzhou, Guangdong China; 5 Department of Pediatric Dentistry, Guanghua School of Stomatology, Affiliated Stomatological Hospital, Sun Yat-sen University, Guangzhou, Guangdong China

**Keywords:** MagT1, ERK/MAPK signaling pathway, Magnesium, Odontogenesis, BMMSCs

## Abstract

**Background:**

Bone marrow mesenchymal stem cells (BMMSCs) are suitable cell sources for dental pulp regeneration, but the mechanism of BMMSCs differentiation into odontogenic lineage remains unknown. The aim of the present study was to reveal the role of magnesium transporter protein 1 (MagT1) and MAPK pathways in the odontogenic differentiation of BMMSCs.

**Methods:**

The RNA sequencing (RNA-seq) was performed to explore the altered transcriptome of BMMSCs undergoing odontogenic differentiation induced by tooth germ cell-condition medium (TGC-CM). Pathway analysis was conducted to explore enriched pathways of the differential expression signature. Automated western blot, real-time PCR, shRNA lentivirus, and flow cytometry were used to detect the function of MagTl and MAPK pathway in odontogenic differentiation of BMMSCs.

**Results:**

RNA-seq identified 622 differentially expressed genes associated with odontogenic differentiation of BMMSCs induced by TGC-CM, some of which were responsible for MAPK pathway. Consistently, we verified that TGC-CM induced odontogenic differentiation of BMMSCs through activating ERK/MAPK pathway, and the inactivation of ERK/MAPK pathway inhibited the odontogenic differentiation induced by TGC-CM. We also found MagT1 protein was significantly increased during odontogenic differentiation of BMMSCs induced by TGC-CMM, in accordance, MagT1 knockdown significantly decreased the extent of mineralized nodules and the protein levels of alkaline phosphatase (ALP), dentin matrix protein 1 (DMP-1), and dentin sialophosphoprotein (DSP). Flow cytometry showed that intracellular Mg^2+^ was significantly reduced in MagT1-knockdown BMMSCs, indicating the suppression of MagT1 inhibited odontogenic differentiation of BMMSCs by decreasing intracellular Mg^2+^. Finally, we performed RNA-seq to explore the altered transcriptome of MagT1-knockdown BMMSCs undergoing odontogenic differentiation and identified 281 differentially expressed genes, some of which were involved in MAPK pathway. Consistently, automated western blot analysis found the ERK/MAPK pathway was inhibited in MagT1-knockdown BMMSCs during odontogenic differentiation, indicating that suppression of MagT1 inhibited odontogenic differentiation of BMMSCs via ERK/MAPK pathway.

**Conclusions:**

This study identified the significant alteration of transcriptome in BMMSCs undergoing odontogenic differentiation induced by TGC-CM. We clarified the pivotal role of MagT1 and ERK/MAPK pathway in odontogenic differentiation of BMMSCs, and suppression of MagT1 inhibited the odontogenic differentiation of BMMSCs by decreasing the intracellular Mg^2+^ and inactivating ERK/MAPK pathway.

**Electronic supplementary material:**

The online version of this article (10.1186/s13287-019-1148-6) contains supplementary material, which is available to authorized users.

## Background

Regenerative endodontic procedures (REPs) have been defined as biologically based procedures designed to replace damaged structures, including dentin and root structures, as well as cells of the pulp-dentin complex. REPs are the most potential treatment of endodontic disease. Stem cells are implanted into the biological stent by tissue engineering technique, then proliferate, migrate, and differentiate into different cell types in pulp induced by scaffolds and growth factors [1, 2]. There are two basically categories of stem cells used in REPs: dental stem cells and non-dental stem cells. Dental stem cells are isolated and characterized as follows: dental pulp stem cells (DPSCs) from pulp of permanent teeth, immature dental stem cells from primary teeth, periodontal ligament stem cells (PDLSCs), and stem cells from apical papilla (SCAP), etc.; Non-dental stem cell sources including embryonic stem cells, stem cells from human umbilical cord tissue, stem cells from hair follicle dermal papilla, adipose tissue derived mesenchymal stem cells, and bone marrow mesenchymal stem cells (BMMSCs) [3].

Although stem cells have been identified in most oral tissues, it is the stem cells surrounding the periapical region that are most likely to be involved in REPs, including stem cells of SCAP, periodontal PDLSCs, DPSCs, and BMMSCs [4]. In 2015, a clinical study was performed to evaluate the presence of mesenchymal stem cells (MSCs) following the evoked-bleeding step in regenerative endodontic procedures in mature teeth with pulp necrosis and apical lesions [5]. It was found that there is a substantial influx of MSCs into root canals during regenerative procedures, resulting in an increase greater than 31-fold in the expression of MSC markers. Since the dental pulp and apical papilla is no longer present in mature teeth with pulp necrosis and apical lesions, other sources of non-dental MSCs must be presented in the evoked blood, for example, BMMSCs. BMMSCs were relatively easy to obtain in clinically standardized isolation procedures compared to other stem cell sources [6, 7]. As reported, BMMSCs had the potential of differentiation into odontogenic lineage induced by enamel matrix proteins, natural dentine matrix, and tooth germ cell-conditioned medium (TGC-CM) [8–10]. Obeid et al. [11] clarified the potential of autologous BMMSCs to promote hard-tissue formation in direct pulp capping procedures. In accordance, Ishizaka et al. [12] found that BMMSC transplantation yielded significant regenerated pulp-like tissue in pulpectomized root canals in dogs, which had similar qualitative and quantitative patterns of mRNA expression compared with dental pulp tissue by microarray analysis. Thus, BMMSCs are suitable alternative cell sources for REPs; however, the mechanism of odontogenic differentiation of BMMSCs has not yet been well known.

Magnesium transporter protein 1 (MagT1) was identified as a selective Mg^2+^ transporter protein, which possesses five predicted transmembrane regions with a putative signaling sequence and a number of COOH-terminal phosphorylation consensus sites [13]. Published studies found that Mg^2+^ was associated with the biomineralization of the bone and tooth, in addition, Mg^2+^ directly affected the crystallization processes and pattern formation of the inorganic mineral phase [14–16]. The sustained release of Mg^2+^ significantly enhanced the odontogenic differentiation and biomineralization of human dental pulp stem cells [17]. It was found that the mutation of Mg^2+^ transporter CNNM4 and TRPM7 resulted in mineralization defects of dentin, indicating that a disrupted Mg^2+^ transport was involved in the development of the dental abnormalities [18, 19]. Thus, we could not disprove the hypothesis that MagTl, as a selective Mg^2+^ transporter, was involved in the odontogenic differentiation of BMMSCs.

Mitogen-activated protein kinases (MAPK) were an important signal transduction systems involved in the regulation of proliferation, migration, and differentiation of stem cells [20–22]. Published studies found that Mg^2+^ was involved in the regulation of ERK cascade in MDCK cells, Mg^2+^ deprivation decreased levels of phosphorylated ERK1/2 (p-ERK1/2), in accordance, re-addition of Mg^2+^ rescued the p-ERK1/2 levels inhibited by U0126 [23, 24]. Suppression of Mg^2+^ transporter TRPM7 decreased the intracellular basal Mg^2+^ concentration and inhibited the phosphorylation of ERK and JNK [25, 26]. Thus, we speculate that MagTl, as a selective Mg^2+^ transporter implicated in Mg^2+^ homeostasis, may have an effect on odontogenic differentiation through MAPK signaling pathways.

To test these hypotheses, we established in vitro system of odontogenic differentiation of BMMSCs induced by TGC-CM and determined the alteration of transcriptome using RNA sequencing (RNA-seq). Then, we explored whether MagTl and MAPK signaling pathways were involved in the odontogenic differentiation of BMMSCs.

## Methods

### Isolation and culture of BMMSCs

All animal experimental protocols were approved by the Ethics Committee of Sun Yat-sen University. Primary BMMSCs were harvested from 4-week-old Sprague-Dawley rats referred to previous study [27]. Briefly, BMMSCs were isolated by flushing femur and tibia bones with Dulbecco’s modified Eagle’s medium (DMEM) (GIBCO, USA). Cells were cultured at 37 °C, in a 5% CO_2_ incubator, and the growth medium was DMEM supplemented with 10% FBS (GIBCO, USA), 10 mg/mL streptomycin, and 10 U/mL penicillin (Sigma, USA). Experiments were performed with BMMSCs from passages 3 to 5.

### Investigation of BMMSC surface markers’ expression

After 3 passages, the isolated BMMSCs were prepared for examination, 100 μL BMMSCs at a concentration of 1.0 × 10^6^ cells/mL were stained by 20 μL of each of the following rat antibodies: CD45-FITC monoclonal antibody (mAb), CD90-PE mAb, CD29-DAPI mAb, and CD11b/c-APC mAb (Ebioscience, USA). Negative control staining was performed using the corresponding isotype control antibodies. The samples were incubated at 37 °C for 30 min, centrifuged, washed twice with PBS, and examined by flow cytometry (BD, USA).

### Determination of BMMSC differentiation capacity

We determined the multi-potential differentiation of BMMSCs into osteoblasts and adipocytes in vitro. To explore the potential of differentiation into osteoblasts, BMMSCs were induced for 21 days in osteogenic medium, which consisted of DMEM, 10% FBS, 100 nmol dexamethasone, 10 mmol β-glycerophosphate, and 0.2 mmol ascorbic acid (Sigma, USA), and osteogenic differentiation was measured by Alizarin Red S staining. To verify the adipogenic differentiation potential, BMMSCs were induced for 21 days in adipogenic medium supplemented with 0.5 휇M isobutyl-methylxanthine, 50 휇M indomethacin, 0.5 휇M dexamethasone, and 5 μg/mL insulin (Sigma, USA), and adipogenic differentiation was determined by Oil Red O staining.

### Odontogenic differentiation of BMMSCs induced by TGC-CM

TGC-CM was prepared and used to induce the odontogenic differentiation of BMMSCs as previously described [8, 28]. DMEM supplemented with 10% FBS was used as control medium. BMMSCs were seeded into 6-well plates (Costar, USA) at an initial density of 1 × 10^5^ cells/well and cultured in either TGC-CM or control medium. Odontogenic differentiation was measured by Alizarin red S staining of the mineralization nodules and determined by automated western blot to explore the protein expression of ALP, odontoblast-specific marker DSP, and DMP-1.

### Alizarin red S staining

Alizarin Red S (Sigma, USA) staining was performed to evaluate the mineralization nodules in the extracellular matrix of BMMSCs. Cells were washed three times with ultrapure water and were destained in 10% cetylpyridinium chloride monohydrate buffer for 30 min, and the absorbance was read at 575 nm. Values are expressed as the relative extracellular matrix mineralization.

### Automated western blot analysis

Total proteins of BMMSCs were isolated using RIPA (Cell Signaling Technology, USA). The automated western blot was performed using Simple Wes (Protein Simple, USA) following the manufacturer’s protocol. Briefly, 2.5 μg of protein from the cell lysates was added to the standard fluorescent mastermix, then was loaded into corresponding wells of the prefilled Wes assay plate, along with antibody diluent (Protein Simple, USA), anti-MagT1 (Proteintech, USA), anti-DSP (Santa Cruz, USA), anti-DMP-1 (Genetex, USA), anti-ALP (Affinity, USA), anti-ERK (Cell Signaling Technology, USA), anti-p-ERK (Cell Signaling Technology, USA), anti-JNK (Cell Signaling Technology, USA), anti-p-JNK (Cell Signaling Technology, USA), anti-p38 (Cell Signaling Technology, USA), anti-p-p38(Cell Signaling Technology, USA), anti-β-Tublin (Affinity, USA), anti-rabbit secondary antibody (Protein Simple, USA), and Streptavidin-HRP, followed by luminal peroxide mix. The imaging and analysis were done with compass software (Protein Simple).

### RNA sequencing (RNA-seq) and data analysis

RNA-seq was performed referred to published study [29] as follows: (1) total RNA purification: DNA in total RNA was digested with DNase I enzyme, and the digested product was purified using magnetic beads. (2) mRNA enrichment: mRNA was enriched with Oligo (dT) magnetic beads. (3) mRNA fragmentation: The mRNA was mixed with the interrupting reagent and was interrupted in high temperature. (4) cDNA synthesis: First-strand cDNA was synthesized using random-hexamer primers, with the short mRNA fragments as templates. Buffer containing dNTPs, RNaseH, and DNA polymerase I was used to synthesize second-strand cDNA according to the second-strand cDNA synthesis kit instructions (Beyotime, Shanghai, China). Short fragments were purified using the QiaQuick PCR extraction kit (Qiagen, Venlo, Limburg, Netherlands), using Ethidium bromide buffer for end repair and addition of poly(A). Sequencing adapters were then added to the short fragments. (5) PCR amplification: Suitable fragments were selected for the PCR amplification as templates. The cDNA library was sequenced using an Illumina HiSeq™ 2000 (Illumina Inc., USA).

Reads Per Kilobase of transcript per Million mapped reads (RPKM) was used to calculate gene expression levels based on RPKM = 10^9^*C*/*NL*, where *C* is the number of reads solely aligned to one expressed sequence, *N* is the total number of reads simultaneously aligned to all expressed sequences, and *L* is the basic number in the coding sequence of the corresponding expressed sequence. Filtering was then performed to select for a false discovery rate (FDR) adjusted *p* value < 0.05 using the Benjamini-Hochberg method.

Gene ontology (GO, http://www.geneontology.org) analysis and Kyoto Encyclopedia of Genes and Genomes (KEGG, http://www.genome.jp/kegg/pathway.html) pathway analysis were performed to detect molecular functions, biological processes, and pathways associated with the differential expression signature.

### Real-time PCR

Total RNA was extracted from cells by RNA extraction kit (Qiagen, China). qPCR was performed by SYBR-Green PCR kit (Qiagen, China) according to the manufacturer’s instructions in a LightCycler system (ABI, USA). PCR reaction conditions for all assays were 94 °C for 30 s, followed by 40 cycles of amplification (94 °C for 5 s, 58 °C for 30 s, and 72 °C for 30 s). GAPDH mRNA was used to normalize RNA. Primer sequences were DSP, forward 5′-CAGGTAGCCGGAAGCAAGAA and reverse 5′-CTTCTCTCTGCGGTGTCGTT; DMP-1, forward 5′-CGCCCATGGCAAATAGTGAC and reverse 5′-CTCCTTATCGGCGTCCATCC; ALP, forward 5′-TCGATGGCTTTGGTACGGAG and reverse 5′-TGCGGGACATAAGCGAGTTT; Runx2, forward 5′-CAGACCAGCAGCACTCCATA and reverse 5′-GCTTCCATCAGCGTCAACAC; MagT1, forward 5′-GGGCTTTTGCAGCATTGTGT and reverse 5′-AAACTGTGCTTGGCTGCTTC; GAPDH, forward 5′-AACGGCACAGTCAAGGCTGA and reverse 5′-ACGCCAGTAGACTCCACGACAT.

### Determination and inhibition of MAPK signaling pathway

For evaluation of MAPK signaling pathway involvement, BMMSCs were seeded in 6-well plates (Costar, USA). After 24 h incubation, cells were serum starved for 24 h prior to exposure to 2 mL TGC-CM for 0, 15, 30, and 60 min, respectively. BMMSCs were also induced by TGC-CM for 7 and 14 days to determine the MAPK signaling pathway involvement. ERK/MAPK signaling pathway was inhibited by 50 μM ERK inhibitor U0126 (AbMole BioScience, USA) for 2 h.

### Immunocytochemistry

Protein expression of MagT1 in BMMSCs was measured by immunocytochemistry using confocal microscopy. BMMSCs were fixed in 4% paraformaldehyde for 15 min at room temperature and permeabilized in 0.25% TritonX-100 in PBS for 15 min, then incubated in 5% BSA in PBS for 30 min and washed three times in PBS and incubated overnight with primary anti-MagT1 antibody (1:100, Proteintech, USA) at 4 °C. Subsequently, cells were incubated with goat anti-rabbit secondary antibody (1:1000) for 1 h at 37 °C. At last, BMMSCs were washed three times in PBS and then with DAPI to identify nuclei.

### Construction of MagT1 shRNA lentivirus and cell infection

BMMSCs were transfected with lentiviral vectors encoding short hairpin RNA targeting rat MagT1 for MagT1 knockdown (MagT1-shRNA) or a scrambled shRNA as control (Control-shRNA) (GeneChem, Shanghai, China). Then, BMMSCs were infected with MagT1-shRNA or Control-shRNA lentiviruses at a multiplicity of infection (MOI) of 50. The effect of MagT1 shRNA lentivirus was measured by automated western blot analysis and qPCR.

### Measurement of intracellular magnesium by flow cytometry

Intracellular magnesium was measured by indicator Mag-Fluo-4-AM (Invitrogen, USA) referred to the manufacturer’s instructions: (1) preincubated BMMSCs in physiological medium with 120 mM NaCl, 20 mM HEPES, 4.7 mM KCl, 1.2 mM KH_2_PO_4_, 1.2 mM MgSO_4_, 1.25 mM CaCl_2_, and 10 mM glucose for 10 min at temperature 37 °C to allow stabilization and equilibration of ion gradients; (2) Prepared indicator solution of Mag-Fluo-4-AM at 1 mM in high-quality anhydrous DMSO; (3) Prepared a dispersion of the AM ester by vigorously mixing 4 μL of indicator solution and 5 μL of 20% (*w*/*v*) Pluronic F-127 (Invitrogen, USA) in 1 mL of medium; (4) Added 0.25 mL of this dispersion per 0.75 mL of cell containing medium with final concentration for the indictor of 1 μM; (5) Continued incubation 30 min, then washed the cells three times in the final incubation medium and then incubated for a further 30 min to allow complete de-esterification of intracellular AM esters. The cells were analyzed by flow cytometry (BD, USA) at an excitation wavelength of 480 nm and an emission wavelength of 520 nm. The fluorescence intensity of all labeled cells was measured, and the data are reported as the mean fluorescence intensity obtained by averaging 3 separate experiments.

### Statistical analysis

Each experiment was repeated three times. All values were expressed as the mean ± SD and was evaluated by the independent samples *t* test using SPSS 17.0 (SPSS Inc., USA). *p* < 0.05 was considered statistically significant.

## Results

### TGC-CM induced odontogenic differentiation of BMMSCs

**Fig. 1:**
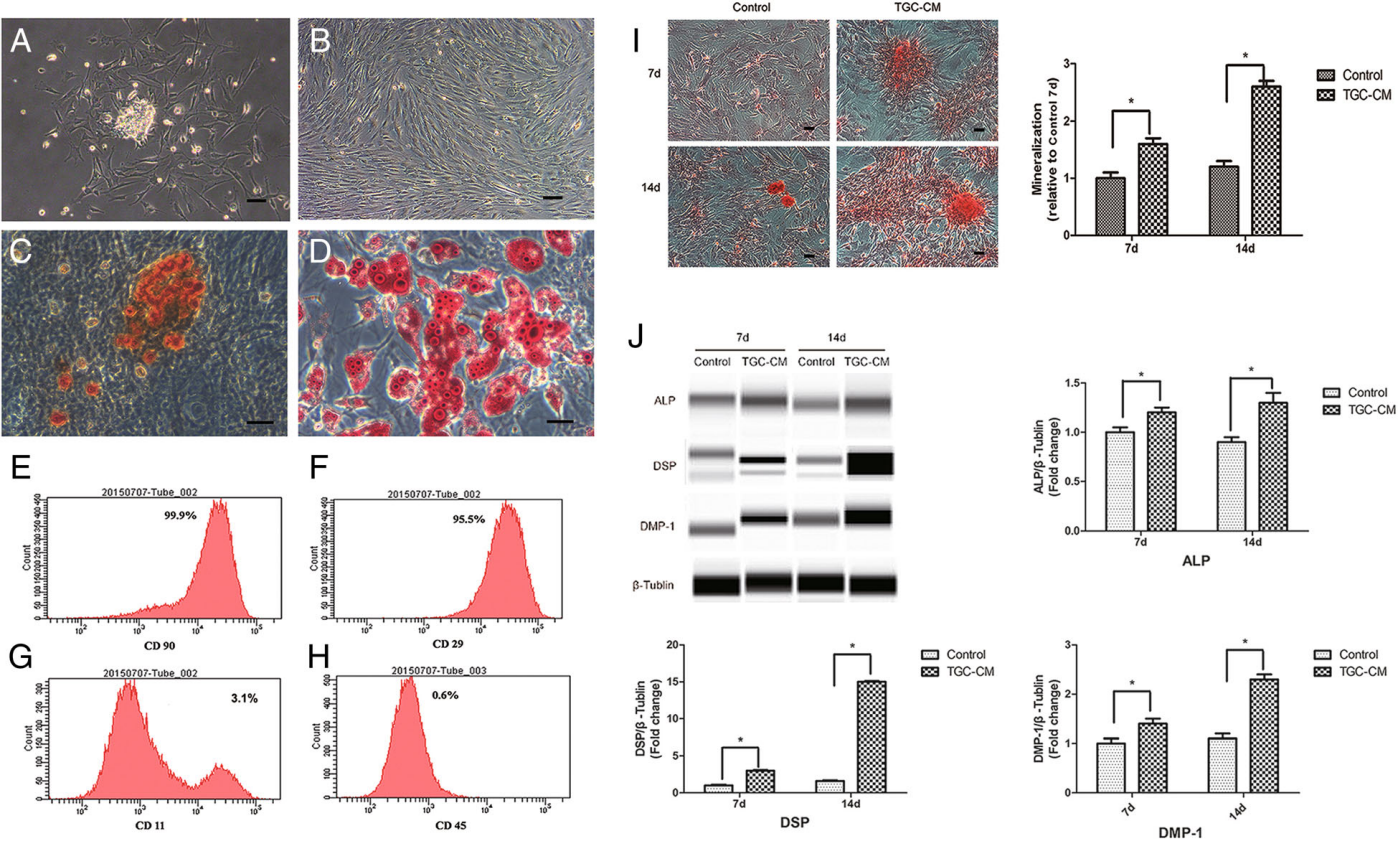
Odontogenic differentiation of BMMSCs induced by TGC-CM. **a** BMMSCs showed colony formation after 48 h in culture. **b** BMMSCs reached 100% confluence after 7 days in culture. **c** Mineral deposits stained by Alizarin Red S in BMMSCs after osteogenic differentiation for 3 weeks. **d** Adipocytes stained by Oil Red O after adipo-induction for 3 weeks. **e**, **f**, **g**, and **h** The expression rate of CD11, CD90, CD29, and CD45. **i** Mineralization nodules and (**j**) ALP, DMP-1, and odontoblast-specific marker DSP protein levels of BMMSCs were significantly increased after incubated by TGC-CM for 7 and 14 days. Scale bar = 100 μm

BMMSCs showed colony formation after 2 days in culture (Fig. 1a) and reached 100% confluence after 7 days (Fig. 1b). After 3 passages, the isolated cells were prepared for examination and analysis by flow cytometry. The results showed that BMMSCs had the potential of differentiation into osteoblasts (Fig. 1c) and adipocytes (Fig. 1d), indicating the multi-lineage differentiation potential of BMMSCs. BMMSCs expressed low levels of the negative marker CD45 and CD11b/c, but expressed high levels of the mesenchymal stem cell marker CD29 and CD90 (Fig. 1e, f, g, and h and Additional file 1: Figure S1).

After BMMSCs were induced by TGC-CM for 7 and 14 days, the protein levels of ALP, DMP-1, and odontoblast-specific marker DSP were significantly increased (Fig. 1j). In accordance, the extent of mineralization nodules was also significantly elevated (Fig. 1i). These results showed that the BMMSCs had the potential of differentiation into odontoblast.

### RNA-seq of BMMSCs during odontogenic differentiation induced by TGC-CM

**Fig. 2:**
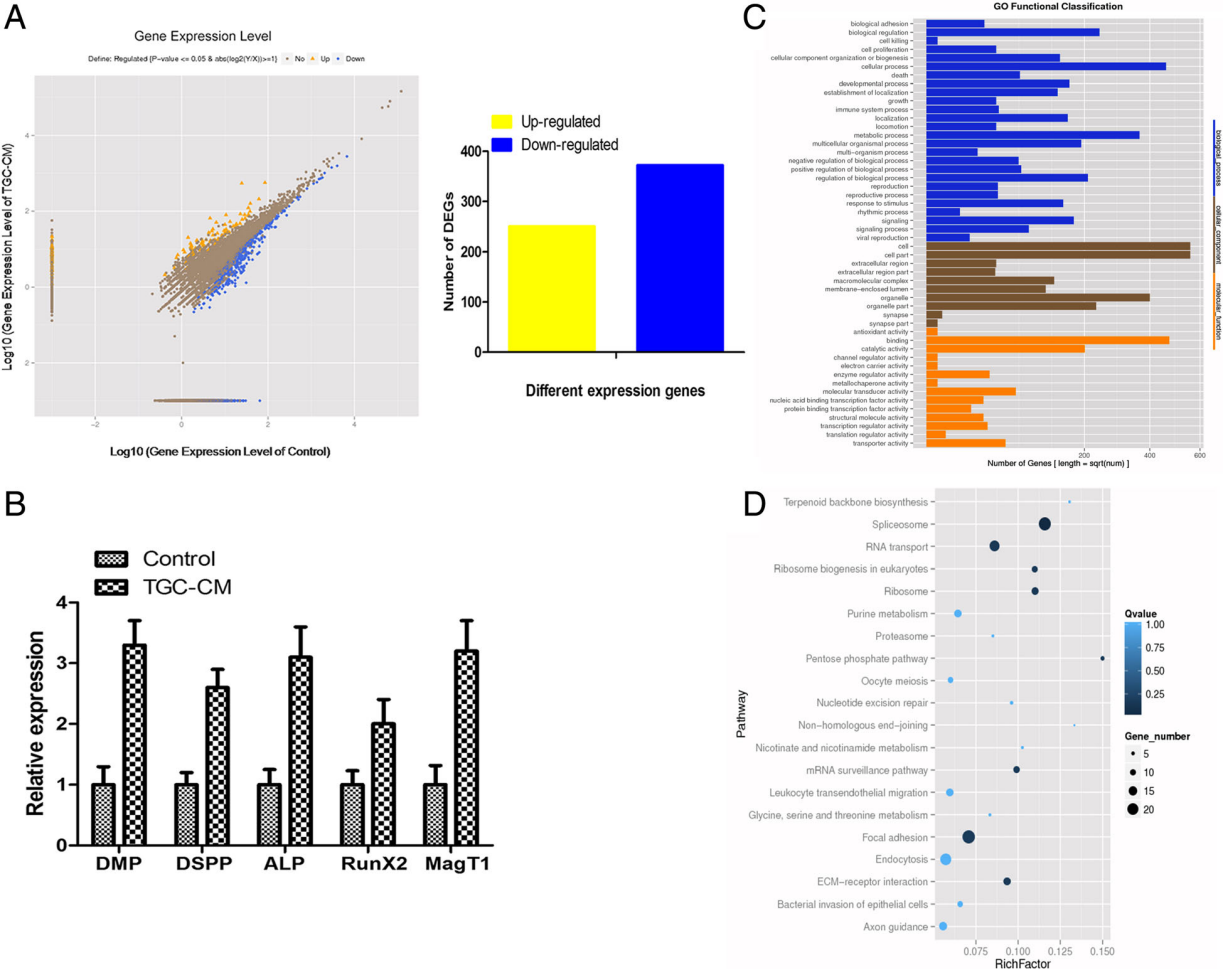
RNA-seq of BMMSCs during odontogenic differentiation. **a** There were 622 genes significantly changed during odontogenic differentiation of BMMSCs for 14 days, with 250 increased and 372 decreased. **b** In consistency with the RNA-seq, the mRNA levels of ALP, DSP, DMP-1, Runx2, and MagT1 were increased by qPCR analysis. **c** GO analysis of differentially expressed genes. **d** Pathway analysis showed differentially expressed genes involved in multiple signal transduction

The RNA-seq was performed to explore the altered transcriptome of BMMSCs undergoing odontogenic differentiation. Compare with control group, there were 622 genes significantly changed after BMMSCs were induced by TGC-CM for 14 days, with 250 increased and 372 decreased (Fig. 2a). qPCR analysis showed that the mRNA levels of ALP, DSP, DMP-1, Runx2, and MagT1 were increased, which was in consistency with the RNA-Seq data (Fig. 2b). GO analysis showed that the most significant biological processes consisted of biological adhesion, cell proliferation, cell killing, cellular metabolic process, regulation of developmental process, and response to stimulus (Fig. 2c). Pathway analysis showed differentially expressed genes involved in multiple signal transduction, including MAPK signaling pathway (Fig. 2d, Additional file 2: Table S1).

### ERK/MAPK signaling pathway regulated the odontogenic differentiation of BMMSCs induced by TGC-CM

**Fig. 3:**
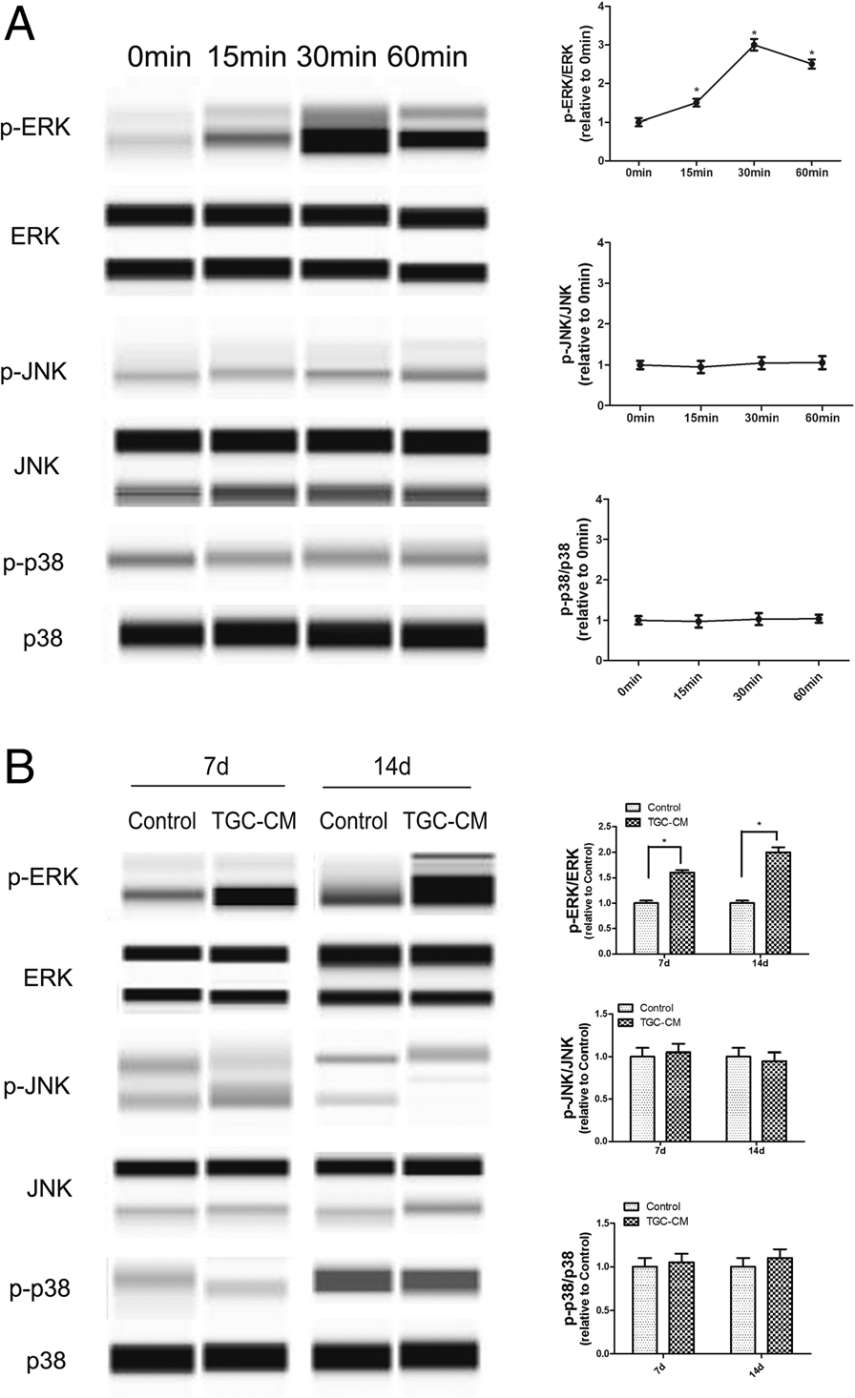
ERK/MAPK signaling pathway was activated by TGC-CM during odontogenic differentiation of BMMSCs. **a** TGC-CM induced significantly high phosphorylated level of ERK in BMMSCs after incubated in TGC-CM for 0 min, 15 min, 30 min, and 60 min. But the phosphorylated level of p38 and JNK did not change. **b** The phosphorylated level of ERK was also increased in BMMSCs during odontogenic differentiation for 7 and 14 days

**Fig. 4:**
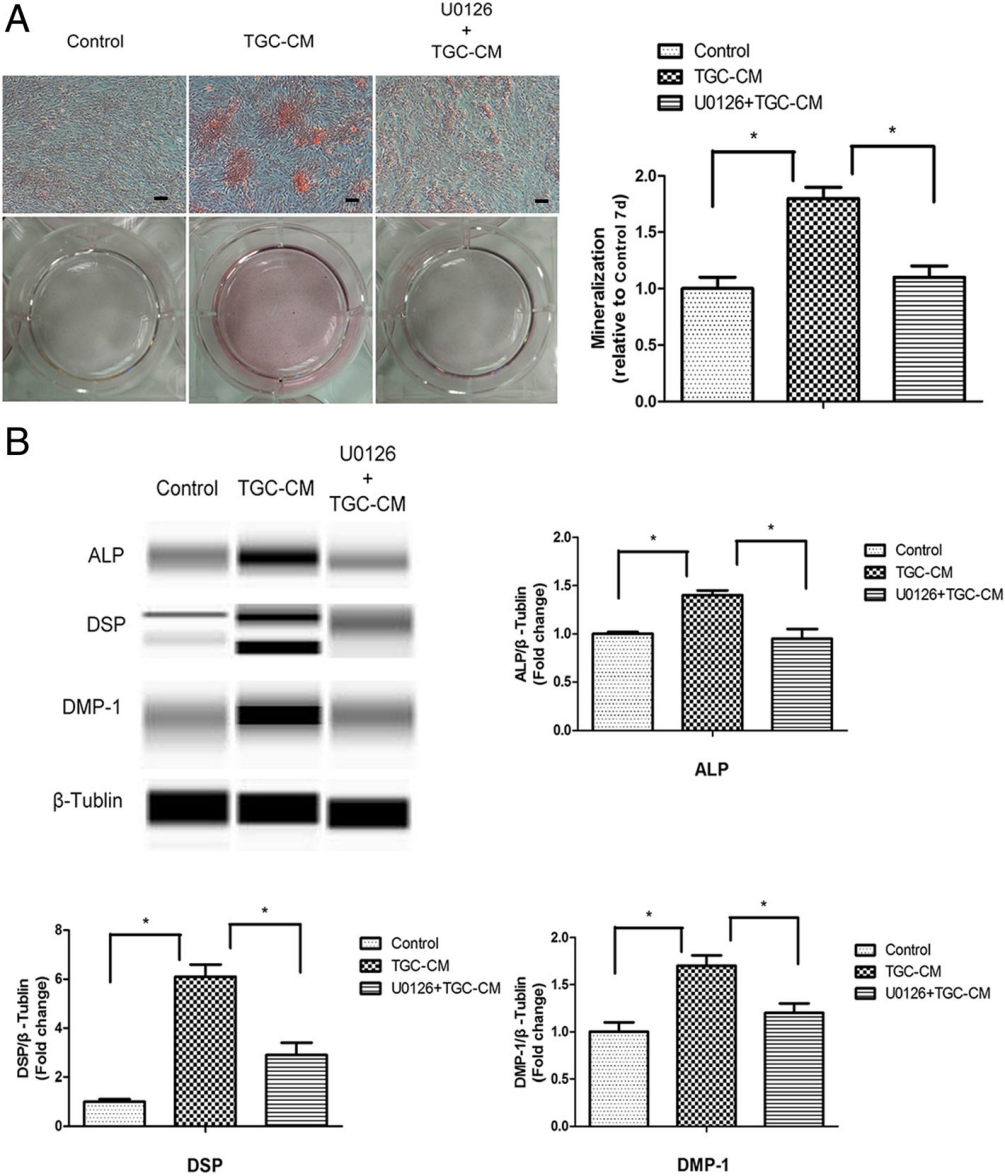
Inactivation of ERK/MAPK signaling pathway inhibited the odontogenic differentiation of BMMSCs induced by TGC-CM. **a** ERK inhibitor U0126 significantly inhibited the mineralization and **b** reduced ALP, DSP, and DMP-1 protein levels of BMMSCs during odontogenic differentiation induced by TGC-CM for 7 days. Scale bar = 100 μm

To confirm the results of pathway analysis whether MAPK signaling pathway was involved in the odontogenic differentiation of BMMSCs induced by TGC-CM, we incubated BMMSCs in TGC-CM for 0 min, 15 min, 30 min, and 60 min and found that the phosphorylated level of ERK was significantly increased, but the phosphorylated level of p38 and JNK did not change (Fig. 3a). ERK/MAPK signaling pathway was also activated in BMMSCs during odontogenic differentiation after 7 and 14 days (Fig. 3b). In accordance, we inhibited the ERK/MAPK signaling pathways by U0126 (Additional file 3: Figure S3) and found the extent of mineralization nodules, and protein levels of ALP, DSP, and DMP-1 were significantly reduced in BMMSCs during odontogenic differentiation for 7 days (Fig. 4a, b).

### MagT1 was involved in the odontogenic differentiation of BMMSCs

**Fig. 5:**
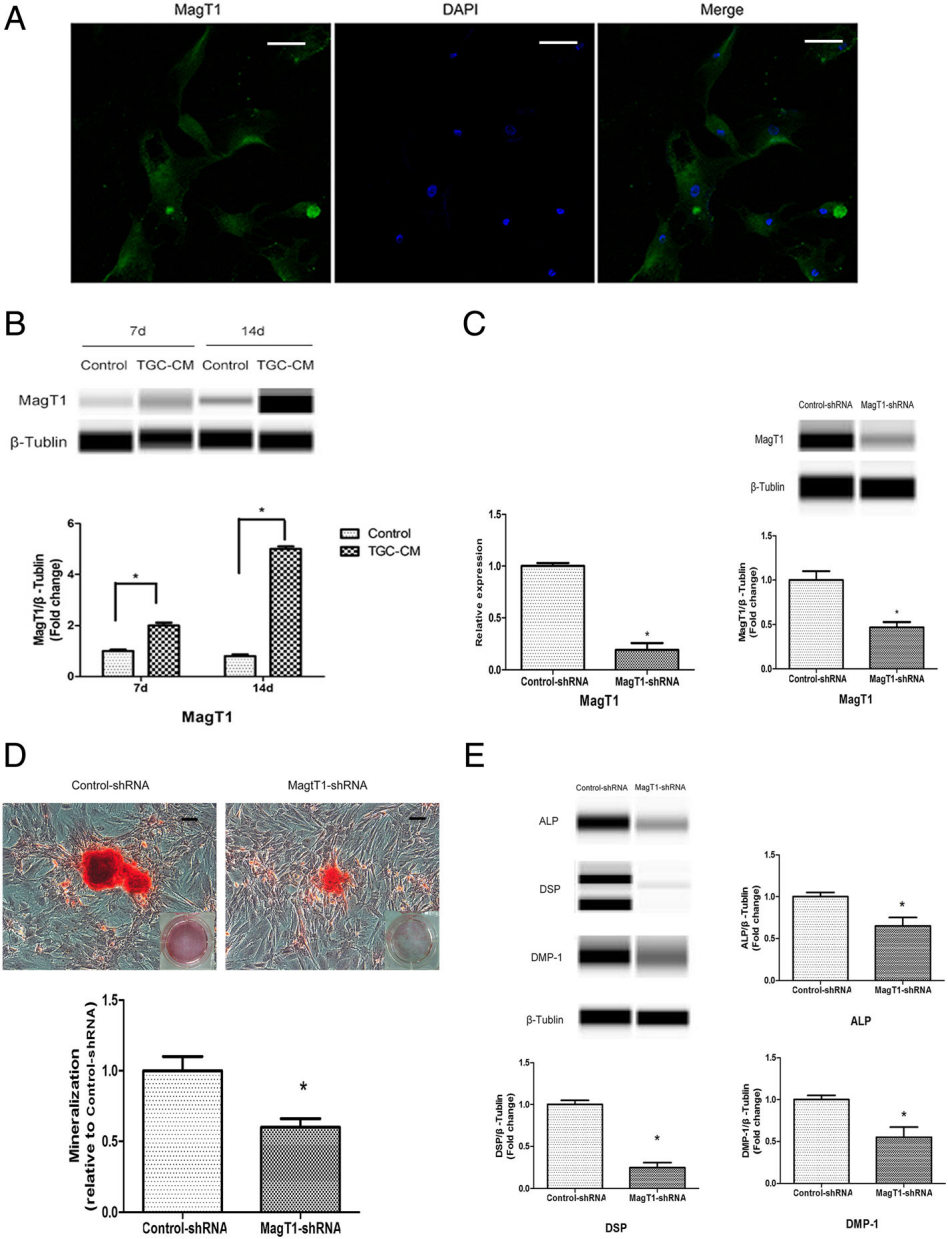
MagT1 was involved in the odontogenic differentiation of BMMSCs. **a** Immunocytochemistry showed protein expression of MagT1 in BMMSCs. **b** The protein expression of MagT1 was increased in BMMSCs during odontogenic differentiation. **c** MagT1-shRNA lentivirus inhibited the mRNA and protein expression of MagT1 in BMMSCs. **d** The protein levels of ALP, DSP, and DMP-1 were decreased in MagT1-shRNA BMMSCs during odontogenic differentiation after 14d. **e** In accordance, the extent of mineralization nodules was also significantly decreased. Scale bar = 100 μm

The immunocytochemistry showed protein expression of MagT1 in BMMSCs by using confocal microscopy (Fig. 5a). Consistent with the RNA-Seq of BMMSCs during odontogenic differentiation, the protein expression of MagT1 in BMMSCs was significantly increased during odontogenic differentiation induced by TGC-CM after 7 and 14 days (Fig. 5b). To explore the role of MagT1 in odontogenic differentiation, BMMSCs were transfected MagT1-shRNA lentivirus and expressed significantly lower levels of MagT1 mRNA and protein than control group (Fig. 5c). The extent of mineralization nodules were decreased in MagT1-shRNA BMMSCs in comparison with Control-shRNA BMMSCs during odontogenic differentiation after 14 days (Fig. 5d). In accordance, the protein levels of ALP, DSP, and DMP-1 were also significantly decreased in MagT1-shRNA BMMSCs (Fig. 5e).

**Fig. 6:**
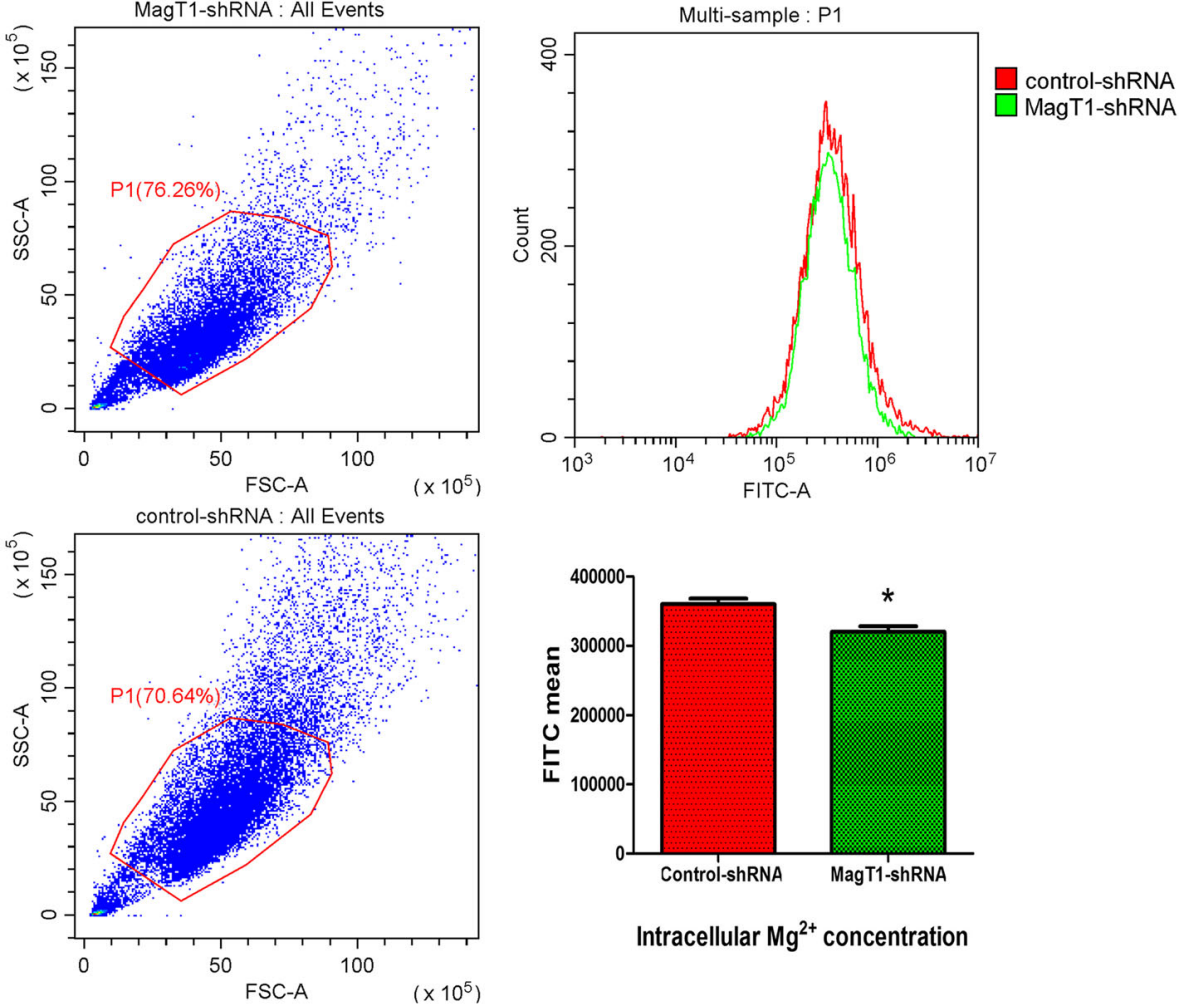
MagT1 knockdown reduced intracellular Mg^2+^ level in BMMSCs. Intracellular Mg^2+^ level was significantly reduced in MagT1 knockdown BMMSCs measured by indicator Mag-Fluo-4-AM using flow cytometry

Intracellular Mg^2+^ concentration was measured by indicator Mag-Fluo-4-AM using flow cytometry, and the results showed that intracellular Mg^2+^ was significantly reduced in MagT1-knockdown BMMSCs (Fig. 6), indicating that suppression of MagT1 inhibited odontogenic differentiation of BMMSCs by decreasing the intracellular Mg^2+^ concentration.

### Suppression of MagT1 inhibited odontogenic differentiation of BMMSCs via ERK/MAPK signaling pathway

**Fig. 7:**
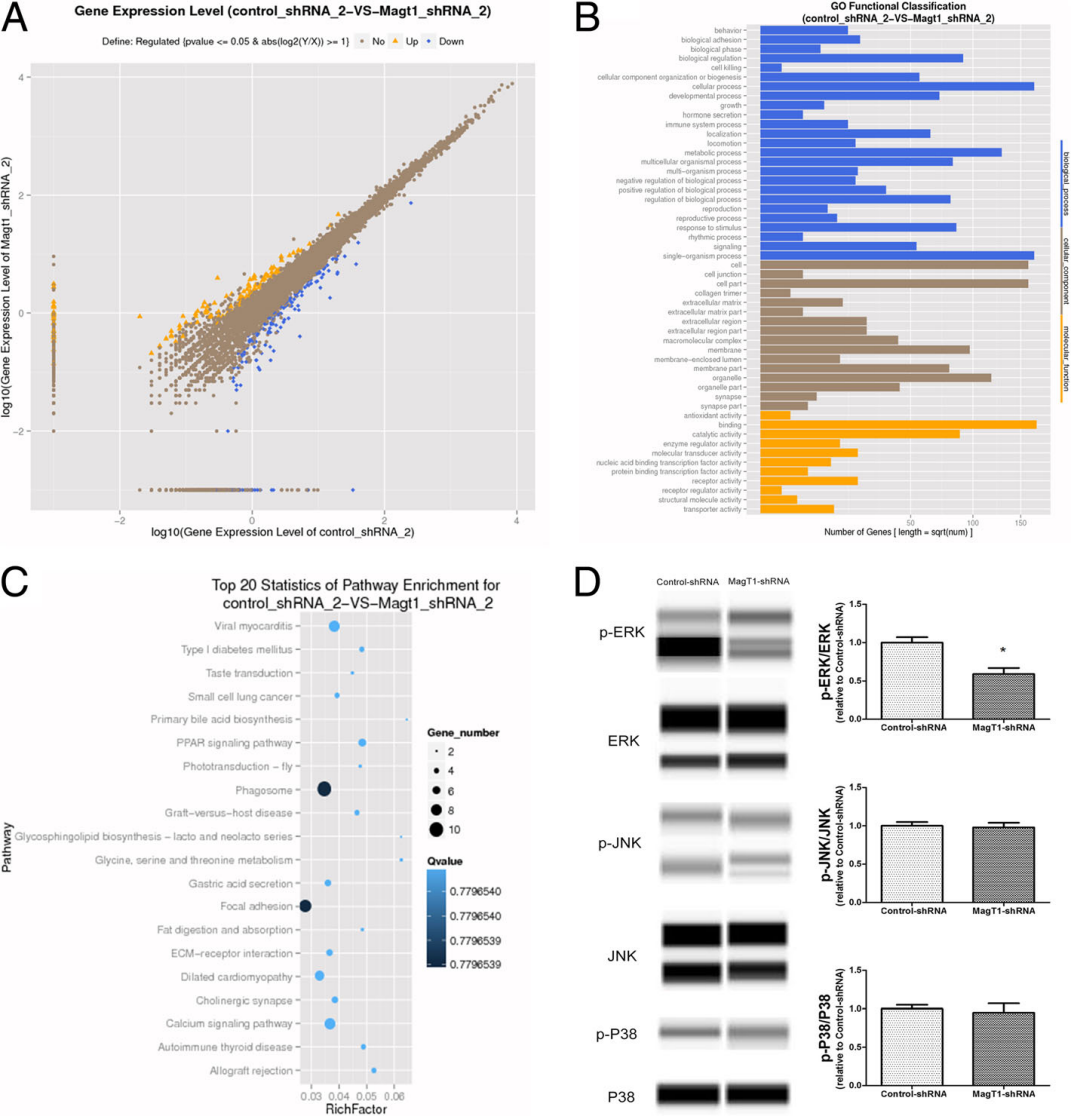
Suppression of MagT1 inhibited odontogenic differentiation of BMMSCs through ERK/MAPK signaling pathway. **a** Scatter plot analysis of RNA-Seq between MagT1-shRNA BMMSCs and Control-shRNA BMMSCs, 281 genes significantly changed. **b** GO analysis of differentially expressed genes. **c** Pathway analysis found differentially expressed genes involved in MAPK signaling pathway. **d** Compared with Control-shRNA BMMSCs, the phosphorylated level of ERK was decreased in MagT1-shRNA BMMSCs during odontogenic differentiation for 14 days

The RNA-seq was conducted to explore the altered transcriptome of MagT1-shRNA BMMSCs undergoing odontogenic differentiation. Compare with Control-shRNA BMMSCs, there were 281 genes significantly changed in MagT1-shRNA BMMSCs undergoing odontogenic differentiation for 14 days, with 126 increased and 155 decreased (Fig. 7a). GO analysis showed that the most significant biological processes consisted of biological adhesion, cell killing, cellular metabolic process, and regulation of developmental process (Fig. 7b).

**Fig. 8:**
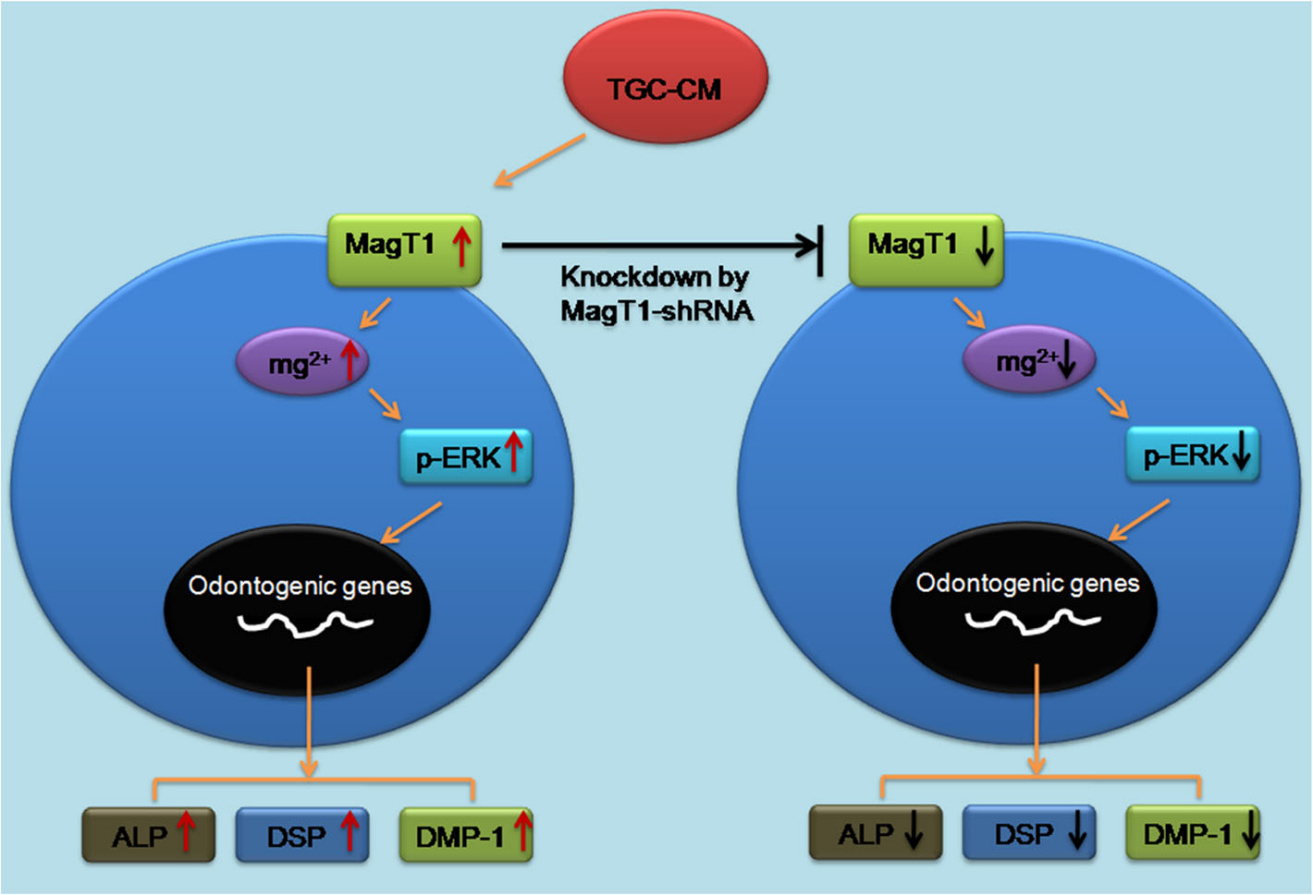
Summary of the function of MagT1 and ERK/MAPK signaling pathway in odontogenic differentiation of BMMSCs induced by TGC-CM

Pathway analysis of differentially expressed genes showed that the most significant pathways consisted of many biochemical metabolism and signal transduction pathway, including MAPK signaling pathway (Fig. 7c, Additional file 4: Table S2). Consistency with pathway analysis, we found the ERK/MAPK signaling pathway was inhibited in MagT1-shRNA BMMSCs in comparison with Control-shRNA BMMSCs undergoing odontogenic differentiation (Fig. 7d), indicating that suppression of MagT1 which disrupted Mg^2+^ transport inhibited odontogenic differentiation of BMMSCs via inactivating ERK/MAPK signaling pathway (Fig. 8).

## Discussion

The present study successfully established in vitro system of odontogenic differentiation of BMMSCs induced by TGC-CM and confirmed that TGC-CM promoted ALP, DMP-1, and odontoblast-specific marker DSP protein expression and mineralization of BMMSCs. In consistent with previous studies, TGC-CM provided the microenvironment and induced stem cell differentiation into odontogenic lineage, such as dermal stem cells [28], stem cells from human umbilical cord tissue [30], stem cells from hair follicle dermal papilla [31], and adipose tissue-derived mesenchymal stem cells [8]. However, the mechanism of odontogenic differentiation of non-dental stem cells induced by TGC-CM remained unclear. Therefore, we performed the high-throughput RNA-seq to explore the alteration of transcriptome in BMMSCs undergoing odontogenic differentiation, after that, GO analysis and pathway analysis were conducted to explore enriched biological processes and signaling pathways of the differential expression signature. The results of RNA-seq identified 622 differentially expressed genes associated with odontogenic differentiation of BMMSCs, many of which were responsible for cell proliferation, cellular metabolic process, regulation of developmental process, and MAPK signaling pathway.

As reported, MAPK signaling pathway, as an important signal transduction systems, was involved in the differentiation of stem cells [32–34]. Yu et al. [35] found that dentine matrix promoted the osteogenic/odontogenic differentiation of BMMSCs by activating ERK and P38 MAPK pathways. Huang et al. [36] clarified the potential of exosomes derived from dental pulp cells to induce odontogenic differentiation of BMMSCs through triggering the P38 MAPK pathways. In this study, to verify the results of pathway analysis that MAPK pathway was involved in the odontogenic differentiation of BMMSCs induced by TGC-CM, we cultured BMMSCs in TGC-CM for 0 min, 15 min, 30 min, and 60 min and confirmed that ERK/MAPK signaling pathway was activated by TGC-CM. Moreover, we verified that TGC-CM induced odontogenic differentiation of BMMSCs through activation of ERK/MAPK signaling pathway and found the inactivation of ERK inhibited by U0126 resulted in significant reduction of ALP, DSP, and DMP-1 protein levels and mineralization nodules.

The results of RNA-seq, qPCR, and automated western blot showed that MagT1 was significantly increased in BMMSCs during odontogenic differentiation. Therefore, we constructed a MagT1-shRNA lentivirus for MagT1 knockdown to explore the function of MagT1in odontogenic differentiation of BMMSCs. As reported, MagT1 identified as a selective Mg^2+^ transporter was implicated in Mg^2+^ homeostasis [13]. Some studies found Mg^2+^ and its transporter TRPM7 regulated the odontogenic differentiation of dental pulp stem cells and were involved in mineralization of dentin [17, 37]. Luder et al. [18] revealed that mutation of Mg^2+^ transporter CNNM4 resulted in mineralization defects of both enamel and dentin, which were associated with significantly abnormal magnesium concentrations, indicating that a disrupted Mg^2+^ transport was involved in the development of the dental abnormalities. Nakano et al. [19] also found TRPM7 knockdown in mice led to significantly hypomineralized in teeth, suggested that TRPM7 is essential for biomineralization of enamel and dentin by providing sufficient Mg^2+^ for the ALPL activity, underlining the key importance of Mg^2+^ transporter for biomineralization. In consistent with previous studies, we found MagT1 protein was significantly increased during odontogenic differentiation of BMMSCs induced by TGC-CMM. In accordance, MagT1 knockdown significantly decreased the ALP, DSP, and DMP-1 protein levels and the extent of mineralized nodules, indicating the pivotal role of MagT1 in odontogenic differentiation of BMMSCs. The results of flow cytometry showed that intracellular Mg^2+^ level was significantly reduced in MagT1-knockdown BMMSCs, indicating that suppression of MagT1 inhibited odontogenic differentiation of BMMSCs by decreasing the intracellular Mg^2+^ concentration.

Mg^2+^ is the most abundant divalent cation in mammalian cells and is an essential cofactor for ATP, polyphosphates such as DNA and RNA, and metabolic enzymes. In 2011, Li et al. [38] revealed a role for Mg^2+^ as an intracellular second messenger coupling cell-surface receptor activation to intracellular effectors, and MagT1 deficiency abrogates the Mg^2+^ influx, leading to impaired responses to antigen receptor engagement, including defective activation of phospholipase Cγ1 in T cells. It revealed that Mg^2+^ plays as second messenger in intracellular signaling. In accordance, published studies found that Mg^2+^ deprivation decreased levels of p-ERK1/2 in MDCK cells, and re-addition of Mg^2+^ rescued the p-ERK1/2 levels, indicating the pivotal role of Mg^2+^ in ERK cascade [23, 24]. Suppression of Mg^2+^ transporter TRPM7 inhibited the phosphorylation of ERK by decreasing the intracellular basal Mg^2+^ concentration [25, 26]. By now, we have discovered the important role of Mg^2+^ transporter MagT1 and ERK/MAPK signaling pathway in odontogenic differentiation of BMMSCs. According to the findings of previous studies that suppression of Mg^2+^ transporter and Mg^2+^ deprivation inhibited the activation of ERK/MAPK signaling pathway [23–26], we speculated that suppression of Mg^2+^ transporter MagTl might have an effect on odontogenic differentiation of BMMSCs through ERK/MAPK signaling pathways. To test this hypothesis, we performed RNA-Seq to explore the altered transcriptome of MagT1-shRNA BMMSCs undergoing odontogenic differentiation and identified 281 differentially expressed genes, some of which were involved in MAPK signaling pathway. In consistent with the results of RNA-Seq, automated western blot analysis found the ERK/MAPK signaling pathway was inhibited in MagT1-shRNA BMMSCs during odontogenic differentiation, indicating that suppression of MagT1 which disrupted Mg^2+^ transport inhibited odontogenic differentiation of BMMSCs via ERK/MAPK signaling pathway.

Finally, we need to point out the limitation of this study. Some animal experiments such as heterotopia regeneration experiment in nude mice should be performed to improve the significance of our study. But there is an uncontrollable factor, which is that the TGC-CM cannot be used continuously to stimulate BMMSCs in vivo. However, in the present study, we mainly focused on the unrevealed mechanism of odontogenic differentiation of BMMSCs induced by TGC-CM in vitro. Although the results of in vitro study verified by several assays, including RNA-seq, pathway analysis, automated western blot, qPCR, shRNA lentivirus, and flow cytometry may not be optimal, but it should be sufficient to draw a conclusion that suppression of MagT1 inhibited the odontogenic differentiation of BMMSCs induced by TGC-CM through decreasing the intracellular Mg^2+^ and inactivating ERK/MAPK pathway in vitro. Further study should be performed to clarify the role of MagT1 in tooth development and odontogenic differentiation in vivo (Additional file 5).

## Conclusions

In conclusions, this study identified the significant alteration of transcriptome in BMMSCs undergoing odontogenic differentiation induced by TGC-CM. We clarified the pivotal role of MagT1 and ERK/MAPK signaling pathway in odontogenic differentiation of BMMSCs, and suppression of MagT1 inhibited the odontogenic differentiation of BMMSCs by decreasing the intracellular Mg^2+^ and inactivating ERK/MAPK signaling pathway in vitro.

## Additional file

Additional file 1: **Figure S1.** The expression rate of CD11b/c was only 3.1%, when compared to negative control group staining by isotype control antibodies. (PDF 23 kb)

Additional file 2: **Table S1.** Pathway analysis of differentially expressed genes (DEGs) in BMMSCs during odontogenic differentiation. All pathways are ordered according to the *p* value, and MAPK pathway is ranked 63. (DOCX 55 kb)

Additional file 3: **Figure S3.** The effect of U0126 in p-ERK/ERK during TGC-CM mediated odontogenic differentiation of BMMSCs for 7 days. U0126 significantly reduced the phosphorylated level of ERK. (PDF 245 kb)

Additional file 4: **Table S2.** Pathway analysis of differentially expressed genes (DEGs) in MagT1-knockdown BMMSCs during odontogenic differentiation. All pathways are ordered according to the *p* value, and MAPK pathway is ranked 123. (DOCX 46 kb)

Additional file 5: **Figure S2.** A Original non-edited western blotting bands performed by Protein Simple Western of Fig. 1j in this article. B(a) Original non-edited western blotting bands performed by Protein Simple Western of Fig. 3a in this article. B(b) Original non-edited western blotting bands performed by Protein Simple Western of Fig. 3b in this article. C Original non-edited western blotting bands performed by Protein Simple Western of Fig. 4 in this article. D(a, b, c) Original non-edited western blotting bands performed by Protein Simple Western of Fig. 5b/C/E in this article. E Original non-edited western blotting bands performed by Protein Simple Western of Fig. 7d in this article. (PDF 706 kb)

## Abbreviations

ALP: Alkaline phosphatase
BMMSCs: Bone marrow mesenchymal stem cells
DMP-1: Dentin matrix protein 1
DSP: Dentin sialophosphoprotein
ERK: Extracellular signal-related
GO: Gene ontology
JNK: c-Jun NH2-terminal kinase
MagT1: Magnesium transporter protein 1
MAPK: Mitogen-activated protein kinases
qPCR: Quantitative polymerase chain reaction
REPs: Regenerative endodontic procedures
RNA-seq: RNA sequencing
shRNA: Short hairpin RNA
TGC-CM: Tooth germ cell-condition medium
