# Supplementary material for: MagT1 regulated the odontogenic differentiation of BMMSCs induced byTGC-CM via ERK signaling pathway
Source: Stem Cell Res Ther. 2019 Jan 31;10:48. doi: 10.1186/s13287-019-1148-6 (PMC6357492; doi:10.1186/s13287-019-1148-6)
Supplement: Supplementary file 1 — Figure S1. The expression rate of CD11b/c was only 3.1%, when compared to negative control group staining by isotype control antibodies. (PDF 23 kb) [file 13287_2019_1148_MOESM1_ESM.pdf]

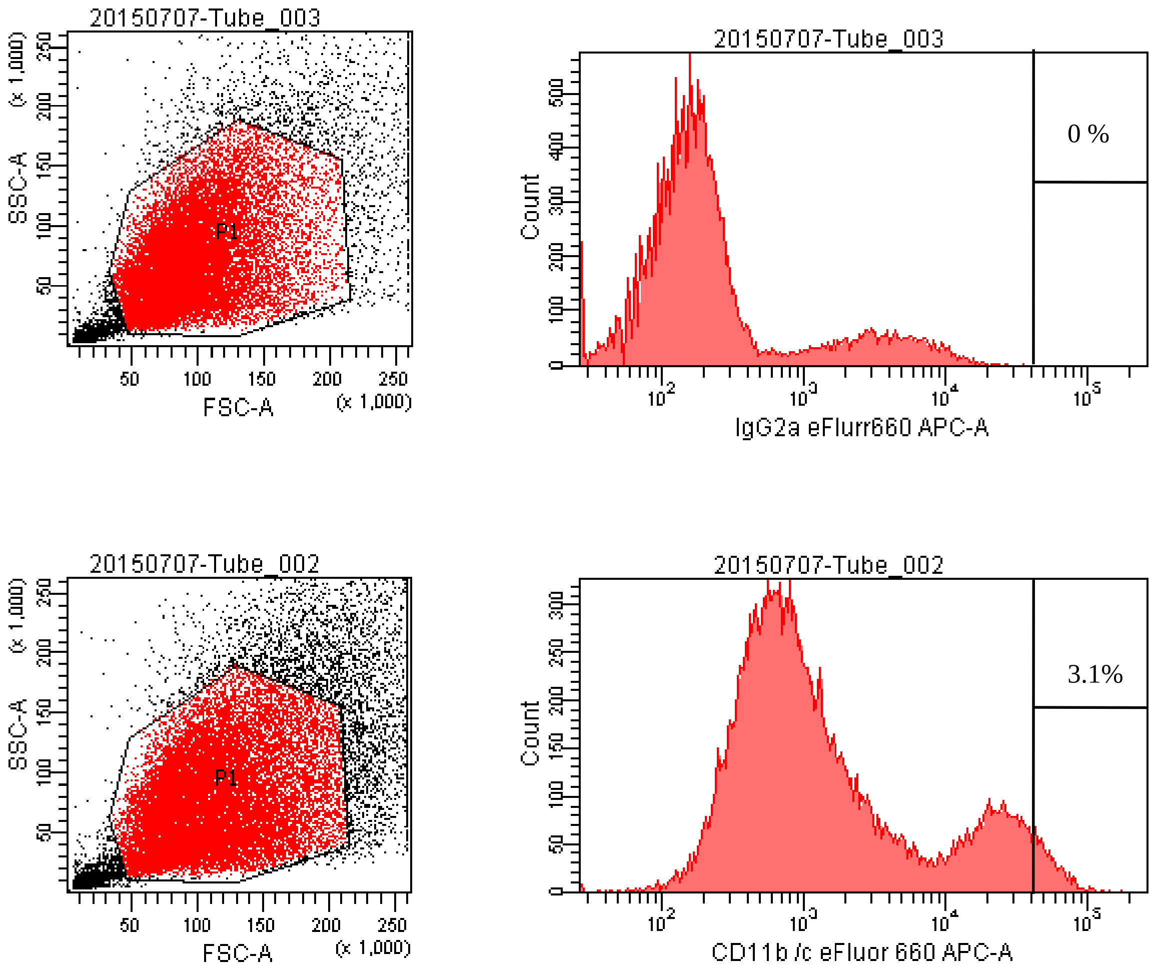

Figure S1 the expression rate of CD11b/c was only 3.1%, when compared to negative control group staining by isotype control antibodies
