# Supplementary material for: MagT1 regulated the odontogenic differentiation of BMMSCs induced byTGC-CM via ERK signaling pathway
Source: Stem Cell Res Ther. 2019 Jan 31;10:48. doi: 10.1186/s13287-019-1148-6 (PMC6357492; doi:10.1186/s13287-019-1148-6)
Supplement: Supplementary file 2 — Table S1. Pathway analysis of differentially expressed genes (DEGs) in BMMSCs during odontogenic differentiation. All pathways are ordered according to the p value, and MAPK pathway is ranked 63. (DOCX 55 kb) [file 13287_2019_1148_MOESM2_ESM.docx]

**Table S1. Pathway analysis of differentially expressed genes (DEGs) in BMMSCs during odontogenic differentiation. All pathways are ordered according to the p value, and MAPK pathway is ranked 63.**

| **#** | **Pathway** | **Number of DEGs (percent of total DEGs)** | **P value** | **Q value** | **Pathway ID** |
| --- | --- | --- | --- | --- | --- |
| 1 | [Spliceosome](file:///E:\01%20%E5%8D%9A%E5%A3%AB%E8%AE%BA%E6%96%87%E6%92%B0%E5%86%99%202016-2-1\1%20%E7%AC%AC%E4%B8%80%E9%83%A8%E5%88%86%20%EF%BC%88%E4%B8%89%EF%BC%89BMSCs%E4%B8%8E%E7%89%99%E8%83%9A%E7%BB%86%E8%83%9E%E4%BD%93%E5%A4%96%E9%97%B4%E6%8E%A5%E5%85%B1%E5%9F%B9%E5%85%BB%E6%A8%A1%E5%9E%8B%E4%B8%AD%E7%9A%84%E8%BD%AC%E5%BD%95%E7%BB%84%E8%A1%A8%E8%BE%BE%E8%B0%B1\pathway%E6%95%B0%E6%8D%AE%20Control_1-VS-Osteogenic_Medium_Treat_1.htm#gene1) | 22 (4.04%) | 6.812228e-06 | 0.001423756 | ko03040 |
| 2 | [Ribosome](file:///E:\01%20%E5%8D%9A%E5%A3%AB%E8%AE%BA%E6%96%87%E6%92%B0%E5%86%99%202016-2-1\1%20%E7%AC%AC%E4%B8%80%E9%83%A8%E5%88%86%20%EF%BC%88%E4%B8%89%EF%BC%89BMSCs%E4%B8%8E%E7%89%99%E8%83%9A%E7%BB%86%E8%83%9E%E4%BD%93%E5%A4%96%E9%97%B4%E6%8E%A5%E5%85%B1%E5%9F%B9%E5%85%BB%E6%A8%A1%E5%9E%8B%E4%B8%AD%E7%9A%84%E8%BD%AC%E5%BD%95%E7%BB%84%E8%A1%A8%E8%BE%BE%E8%B0%B1\pathway%E6%95%B0%E6%8D%AE%20Control_1-VS-Osteogenic_Medium_Treat_1.htm#gene2) | 12 (2.2%) | 0.001302082 | 0.121787017 | ko03010 |
| 3 | [RNA transport](file:///E:\01%20%E5%8D%9A%E5%A3%AB%E8%AE%BA%E6%96%87%E6%92%B0%E5%86%99%202016-2-1\1%20%E7%AC%AC%E4%B8%80%E9%83%A8%E5%88%86%20%EF%BC%88%E4%B8%89%EF%BC%89BMSCs%E4%B8%8E%E7%89%99%E8%83%9A%E7%BB%86%E8%83%9E%E4%BD%93%E5%A4%96%E9%97%B4%E6%8E%A5%E5%85%B1%E5%9F%B9%E5%85%BB%E6%A8%A1%E5%9E%8B%E4%B8%AD%E7%9A%84%E8%BD%AC%E5%BD%95%E7%BB%84%E8%A1%A8%E8%BE%BE%E8%B0%B1\pathway%E6%95%B0%E6%8D%AE%20Control_1-VS-Osteogenic_Medium_Treat_1.htm#gene3) | 18 (3.3%) | 0.001748139 | 0.121787017 | ko03013 |
| 4 | [Ribosome biogenesis in eukaryotes](file:///E:\01%20%E5%8D%9A%E5%A3%AB%E8%AE%BA%E6%96%87%E6%92%B0%E5%86%99%202016-2-1\1%20%E7%AC%AC%E4%B8%80%E9%83%A8%E5%88%86%20%EF%BC%88%E4%B8%89%EF%BC%89BMSCs%E4%B8%8E%E7%89%99%E8%83%9A%E7%BB%86%E8%83%9E%E4%BD%93%E5%A4%96%E9%97%B4%E6%8E%A5%E5%85%B1%E5%9F%B9%E5%85%BB%E6%A8%A1%E5%9E%8B%E4%B8%AD%E7%9A%84%E8%BD%AC%E5%BD%95%E7%BB%84%E8%A1%A8%E8%BE%BE%E8%B0%B1\pathway%E6%95%B0%E6%8D%AE%20Control_1-VS-Osteogenic_Medium_Treat_1.htm#gene4) | 10 (1.83%) | 0.003260792 | 0.139811894 | ko03008 |
| 5 | [ECM-receptor interaction](file:///E:\01%20%E5%8D%9A%E5%A3%AB%E8%AE%BA%E6%96%87%E6%92%B0%E5%86%99%202016-2-1\1%20%E7%AC%AC%E4%B8%80%E9%83%A8%E5%88%86%20%EF%BC%88%E4%B8%89%EF%BC%89BMSCs%E4%B8%8E%E7%89%99%E8%83%9A%E7%BB%86%E8%83%9E%E4%BD%93%E5%A4%96%E9%97%B4%E6%8E%A5%E5%85%B1%E5%9F%B9%E5%85%BB%E6%A8%A1%E5%9E%8B%E4%B8%AD%E7%9A%84%E8%BD%AC%E5%BD%95%E7%BB%84%E8%A1%A8%E8%BE%BE%E8%B0%B1\pathway%E6%95%B0%E6%8D%AE%20Control_1-VS-Osteogenic_Medium_Treat_1.htm#gene5) | 13 (2.39%) | 0.00363084 | 0.139811894 | ko04512 |
| 6 | [mRNA surveillance pathway](file:///E:\01%20%E5%8D%9A%E5%A3%AB%E8%AE%BA%E6%96%87%E6%92%B0%E5%86%99%202016-2-1\1%20%E7%AC%AC%E4%B8%80%E9%83%A8%E5%88%86%20%EF%BC%88%E4%B8%89%EF%BC%89BMSCs%E4%B8%8E%E7%89%99%E8%83%9A%E7%BB%86%E8%83%9E%E4%BD%93%E5%A4%96%E9%97%B4%E6%8E%A5%E5%85%B1%E5%9F%B9%E5%85%BB%E6%A8%A1%E5%9E%8B%E4%B8%AD%E7%9A%84%E8%BD%AC%E5%BD%95%E7%BB%84%E8%A1%A8%E8%BE%BE%E8%B0%B1\pathway%E6%95%B0%E6%8D%AE%20Control_1-VS-Osteogenic_Medium_Treat_1.htm#gene6) | 11 (2.02%) | 0.004653711 | 0.139811894 | ko03015 |
| 7 | [Pentose phosphate pathway](file:///E:\01%20%E5%8D%9A%E5%A3%AB%E8%AE%BA%E6%96%87%E6%92%B0%E5%86%99%202016-2-1\1%20%E7%AC%AC%E4%B8%80%E9%83%A8%E5%88%86%20%EF%BC%88%E4%B8%89%EF%BC%89BMSCs%E4%B8%8E%E7%89%99%E8%83%9A%E7%BB%86%E8%83%9E%E4%BD%93%E5%A4%96%E9%97%B4%E6%8E%A5%E5%85%B1%E5%9F%B9%E5%85%BB%E6%A8%A1%E5%9E%8B%E4%B8%AD%E7%9A%84%E8%BD%AC%E5%BD%95%E7%BB%84%E8%A1%A8%E8%BE%BE%E8%B0%B1\pathway%E6%95%B0%E6%8D%AE%20Control_1-VS-Osteogenic_Medium_Treat_1.htm#gene7) | 6 (1.1%) | 0.004682695 | 0.139811894 | ko00030 |
| 8 | [Focal adhesion](file:///E:\01%20%E5%8D%9A%E5%A3%AB%E8%AE%BA%E6%96%87%E6%92%B0%E5%86%99%202016-2-1\1%20%E7%AC%AC%E4%B8%80%E9%83%A8%E5%88%86%20%EF%BC%88%E4%B8%89%EF%BC%89BMSCs%E4%B8%8E%E7%89%99%E8%83%9A%E7%BB%86%E8%83%9E%E4%BD%93%E5%A4%96%E9%97%B4%E6%8E%A5%E5%85%B1%E5%9F%B9%E5%85%BB%E6%A8%A1%E5%9E%8B%E4%B8%AD%E7%9A%84%E8%BD%AC%E5%BD%95%E7%BB%84%E8%A1%A8%E8%BE%BE%E8%B0%B1\pathway%E6%95%B0%E6%8D%AE%20Control_1-VS-Osteogenic_Medium_Treat_1.htm#gene8) | 23 (4.22%) | 0.005540595 | 0.144748044 | ko04510 |
| 9 | [Nucleotide excision repair](file:///E:\01%20%E5%8D%9A%E5%A3%AB%E8%AE%BA%E6%96%87%E6%92%B0%E5%86%99%202016-2-1\1%20%E7%AC%AC%E4%B8%80%E9%83%A8%E5%88%86%20%EF%BC%88%E4%B8%89%EF%BC%89BMSCs%E4%B8%8E%E7%89%99%E8%83%9A%E7%BB%86%E8%83%9E%E4%BD%93%E5%A4%96%E9%97%B4%E6%8E%A5%E5%85%B1%E5%9F%B9%E5%85%BB%E6%A8%A1%E5%9E%8B%E4%B8%AD%E7%9A%84%E8%BD%AC%E5%BD%95%E7%BB%84%E8%A1%A8%E8%BE%BE%E8%B0%B1\pathway%E6%95%B0%E6%8D%AE%20Control_1-VS-Osteogenic_Medium_Treat_1.htm#gene9) | 5 (0.92%) | 0.05496503 | 1.000000000 | ko03420 |
| 10 | [Purine metabolism](file:///E:\01%20%E5%8D%9A%E5%A3%AB%E8%AE%BA%E6%96%87%E6%92%B0%E5%86%99%202016-2-1\1%20%E7%AC%AC%E4%B8%80%E9%83%A8%E5%88%86%20%EF%BC%88%E4%B8%89%EF%BC%89BMSCs%E4%B8%8E%E7%89%99%E8%83%9A%E7%BB%86%E8%83%9E%E4%BD%93%E5%A4%96%E9%97%B4%E6%8E%A5%E5%85%B1%E5%9F%B9%E5%85%BB%E6%A8%A1%E5%9E%8B%E4%B8%AD%E7%9A%84%E8%BD%AC%E5%BD%95%E7%BB%84%E8%A1%A8%E8%BE%BE%E8%B0%B1\pathway%E6%95%B0%E6%8D%AE%20Control_1-VS-Osteogenic_Medium_Treat_1.htm#gene10) | 13 (2.39%) | 0.06028883 | 1.000000000 | ko00230 |
| 11 | [Terpenoid backbone biosynthesis](file:///E:\01%20%E5%8D%9A%E5%A3%AB%E8%AE%BA%E6%96%87%E6%92%B0%E5%86%99%202016-2-1\1%20%E7%AC%AC%E4%B8%80%E9%83%A8%E5%88%86%20%EF%BC%88%E4%B8%89%EF%BC%89BMSCs%E4%B8%8E%E7%89%99%E8%83%9A%E7%BB%86%E8%83%9E%E4%BD%93%E5%A4%96%E9%97%B4%E6%8E%A5%E5%85%B1%E5%9F%B9%E5%85%BB%E6%A8%A1%E5%9E%8B%E4%B8%AD%E7%9A%84%E8%BD%AC%E5%BD%95%E7%BB%84%E8%A1%A8%E8%BE%BE%E8%B0%B1\pathway%E6%95%B0%E6%8D%AE%20Control_1-VS-Osteogenic_Medium_Treat_1.htm#gene11) | 3 (0.55%) | 0.06147624 | 1.000000000 | ko00900 |
| 12 | [Endocytosis](file:///E:\01%20%E5%8D%9A%E5%A3%AB%E8%AE%BA%E6%96%87%E6%92%B0%E5%86%99%202016-2-1\1%20%E7%AC%AC%E4%B8%80%E9%83%A8%E5%88%86%20%EF%BC%88%E4%B8%89%EF%BC%89BMSCs%E4%B8%8E%E7%89%99%E8%83%9A%E7%BB%86%E8%83%9E%E4%BD%93%E5%A4%96%E9%97%B4%E6%8E%A5%E5%85%B1%E5%9F%B9%E5%85%BB%E6%A8%A1%E5%9E%8B%E4%B8%AD%E7%9A%84%E8%BD%AC%E5%BD%95%E7%BB%84%E8%A1%A8%E8%BE%BE%E8%B0%B1\pathway%E6%95%B0%E6%8D%AE%20Control_1-VS-Osteogenic_Medium_Treat_1.htm#gene12) | 20 (3.67%) | 0.0669102 | 1.000000000 | ko04144 |
| 13 | [Nicotinate and nicotinamide metabolism](file:///E:\01%20%E5%8D%9A%E5%A3%AB%E8%AE%BA%E6%96%87%E6%92%B0%E5%86%99%202016-2-1\1%20%E7%AC%AC%E4%B8%80%E9%83%A8%E5%88%86%20%EF%BC%88%E4%B8%89%EF%BC%89BMSCs%E4%B8%8E%E7%89%99%E8%83%9A%E7%BB%86%E8%83%9E%E4%BD%93%E5%A4%96%E9%97%B4%E6%8E%A5%E5%85%B1%E5%9F%B9%E5%85%BB%E6%A8%A1%E5%9E%8B%E4%B8%AD%E7%9A%84%E8%BD%AC%E5%BD%95%E7%BB%84%E8%A1%A8%E8%BE%BE%E8%B0%B1\pathway%E6%95%B0%E6%8D%AE%20Control_1-VS-Osteogenic_Medium_Treat_1.htm#gene13) | 4 (0.73%) | 0.06812693 | 1.000000000 | ko00760 |
| 14 | [Leukocyte transendothelial migration](file:///E:\01%20%E5%8D%9A%E5%A3%AB%E8%AE%BA%E6%96%87%E6%92%B0%E5%86%99%202016-2-1\1%20%E7%AC%AC%E4%B8%80%E9%83%A8%E5%88%86%20%EF%BC%88%E4%B8%89%EF%BC%89BMSCs%E4%B8%8E%E7%89%99%E8%83%9A%E7%BB%86%E8%83%9E%E4%BD%93%E5%A4%96%E9%97%B4%E6%8E%A5%E5%85%B1%E5%9F%B9%E5%85%BB%E6%A8%A1%E5%9E%8B%E4%B8%AD%E7%9A%84%E8%BD%AC%E5%BD%95%E7%BB%84%E8%A1%A8%E8%BE%BE%E8%B0%B1\pathway%E6%95%B0%E6%8D%AE%20Control_1-VS-Osteogenic_Medium_Treat_1.htm#gene14) | 13 (2.39%) | 0.09571719 | 1.000000000 | ko04670 |
| 15 | [Bacterial invasion of epithelial cells](file:///E:\01%20%E5%8D%9A%E5%A3%AB%E8%AE%BA%E6%96%87%E6%92%B0%E5%86%99%202016-2-1\1%20%E7%AC%AC%E4%B8%80%E9%83%A8%E5%88%86%20%EF%BC%88%E4%B8%89%EF%BC%89BMSCs%E4%B8%8E%E7%89%99%E8%83%9A%E7%BB%86%E8%83%9E%E4%BD%93%E5%A4%96%E9%97%B4%E6%8E%A5%E5%85%B1%E5%9F%B9%E5%85%BB%E6%A8%A1%E5%9E%8B%E4%B8%AD%E7%9A%84%E8%BD%AC%E5%BD%95%E7%BB%84%E8%A1%A8%E8%BE%BE%E8%B0%B1\pathway%E6%95%B0%E6%8D%AE%20Control_1-VS-Osteogenic_Medium_Treat_1.htm#gene15) | 9 (1.65%) | 0.09617413 | 1.000000000 | ko05100 |
| 16 | [Proteasome](file:///E:\01%20%E5%8D%9A%E5%A3%AB%E8%AE%BA%E6%96%87%E6%92%B0%E5%86%99%202016-2-1\1%20%E7%AC%AC%E4%B8%80%E9%83%A8%E5%88%86%20%EF%BC%88%E4%B8%89%EF%BC%89BMSCs%E4%B8%8E%E7%89%99%E8%83%9A%E7%BB%86%E8%83%9E%E4%BD%93%E5%A4%96%E9%97%B4%E6%8E%A5%E5%85%B1%E5%9F%B9%E5%85%BB%E6%A8%A1%E5%9E%8B%E4%B8%AD%E7%9A%84%E8%BD%AC%E5%BD%95%E7%BB%84%E8%A1%A8%E8%BE%BE%E8%B0%B1\pathway%E6%95%B0%E6%8D%AE%20Control_1-VS-Osteogenic_Medium_Treat_1.htm#gene16) | 4 (0.73%) | 0.1159868 | 1.000000000 | ko03050 |
| 17 | [Non-homologous end-joining](file:///E:\01%20%E5%8D%9A%E5%A3%AB%E8%AE%BA%E6%96%87%E6%92%B0%E5%86%99%202016-2-1\1%20%E7%AC%AC%E4%B8%80%E9%83%A8%E5%88%86%20%EF%BC%88%E4%B8%89%EF%BC%89BMSCs%E4%B8%8E%E7%89%99%E8%83%9A%E7%BB%86%E8%83%9E%E4%BD%93%E5%A4%96%E9%97%B4%E6%8E%A5%E5%85%B1%E5%9F%B9%E5%85%BB%E6%A8%A1%E5%9E%8B%E4%B8%AD%E7%9A%84%E8%BD%AC%E5%BD%95%E7%BB%84%E8%A1%A8%E8%BE%BE%E8%B0%B1\pathway%E6%95%B0%E6%8D%AE%20Control_1-VS-Osteogenic_Medium_Treat_1.htm#gene17) | 2 (0.37%) | 0.1179922 | 1.000000000 | ko03450 |
| 18 | [Glycine, serine and threonine metabolism](file:///E:\01%20%E5%8D%9A%E5%A3%AB%E8%AE%BA%E6%96%87%E6%92%B0%E5%86%99%202016-2-1\1%20%E7%AC%AC%E4%B8%80%E9%83%A8%E5%88%86%20%EF%BC%88%E4%B8%89%EF%BC%89BMSCs%E4%B8%8E%E7%89%99%E8%83%9A%E7%BB%86%E8%83%9E%E4%BD%93%E5%A4%96%E9%97%B4%E6%8E%A5%E5%85%B1%E5%9F%B9%E5%85%BB%E6%A8%A1%E5%9E%8B%E4%B8%AD%E7%9A%84%E8%BD%AC%E5%BD%95%E7%BB%84%E8%A1%A8%E8%BE%BE%E8%B0%B1\pathway%E6%95%B0%E6%8D%AE%20Control_1-VS-Osteogenic_Medium_Treat_1.htm#gene18) | 4 (0.73%) | 0.1227923 | 1.000000000 | ko00260 |
| 19 | [Axon guidance](file:///E:\01%20%E5%8D%9A%E5%A3%AB%E8%AE%BA%E6%96%87%E6%92%B0%E5%86%99%202016-2-1\1%20%E7%AC%AC%E4%B8%80%E9%83%A8%E5%88%86%20%EF%BC%88%E4%B8%89%EF%BC%89BMSCs%E4%B8%8E%E7%89%99%E8%83%9A%E7%BB%86%E8%83%9E%E4%BD%93%E5%A4%96%E9%97%B4%E6%8E%A5%E5%85%B1%E5%9F%B9%E5%85%BB%E6%A8%A1%E5%9E%8B%E4%B8%AD%E7%9A%84%E8%BD%AC%E5%BD%95%E7%BB%84%E8%A1%A8%E8%BE%BE%E8%B0%B1\pathway%E6%95%B0%E6%8D%AE%20Control_1-VS-Osteogenic_Medium_Treat_1.htm#gene19) | 14 (2.57%) | 0.1310037 | 1.000000000 | ko04360 |
| 20 | [Oocyte meiosis](file:///E:\01%20%E5%8D%9A%E5%A3%AB%E8%AE%BA%E6%96%87%E6%92%B0%E5%86%99%202016-2-1\1%20%E7%AC%AC%E4%B8%80%E9%83%A8%E5%88%86%20%EF%BC%88%E4%B8%89%EF%BC%89BMSCs%E4%B8%8E%E7%89%99%E8%83%9A%E7%BB%86%E8%83%9E%E4%BD%93%E5%A4%96%E9%97%B4%E6%8E%A5%E5%85%B1%E5%9F%B9%E5%85%BB%E6%A8%A1%E5%9E%8B%E4%B8%AD%E7%9A%84%E8%BD%AC%E5%BD%95%E7%BB%84%E8%A1%A8%E8%BE%BE%E8%B0%B1\pathway%E6%95%B0%E6%8D%AE%20Control_1-VS-Osteogenic_Medium_Treat_1.htm#gene20) | 9 (1.65%) | 0.1441067 | 1.000000000 | ko04114 |
| 21 | [Citrate cycle (TCA cycle)](file:///E:\01%20%E5%8D%9A%E5%A3%AB%E8%AE%BA%E6%96%87%E6%92%B0%E5%86%99%202016-2-1\1%20%E7%AC%AC%E4%B8%80%E9%83%A8%E5%88%86%20%EF%BC%88%E4%B8%89%EF%BC%89BMSCs%E4%B8%8E%E7%89%99%E8%83%9A%E7%BB%86%E8%83%9E%E4%BD%93%E5%A4%96%E9%97%B4%E6%8E%A5%E5%85%B1%E5%9F%B9%E5%85%BB%E6%A8%A1%E5%9E%8B%E4%B8%AD%E7%9A%84%E8%BD%AC%E5%BD%95%E7%BB%84%E8%A1%A8%E8%BE%BE%E8%B0%B1\pathway%E6%95%B0%E6%8D%AE%20Control_1-VS-Osteogenic_Medium_Treat_1.htm#gene21) | 3 (0.55%) | 0.1518406 | 1.000000000 | ko00020 |
| 22 | [Mineral absorption](file:///E:\01%20%E5%8D%9A%E5%A3%AB%E8%AE%BA%E6%96%87%E6%92%B0%E5%86%99%202016-2-1\1%20%E7%AC%AC%E4%B8%80%E9%83%A8%E5%88%86%20%EF%BC%88%E4%B8%89%EF%BC%89BMSCs%E4%B8%8E%E7%89%99%E8%83%9A%E7%BB%86%E8%83%9E%E4%BD%93%E5%A4%96%E9%97%B4%E6%8E%A5%E5%85%B1%E5%9F%B9%E5%85%BB%E6%A8%A1%E5%9E%8B%E4%B8%AD%E7%9A%84%E8%BD%AC%E5%BD%95%E7%BB%84%E8%A1%A8%E8%BE%BE%E8%B0%B1\pathway%E6%95%B0%E6%8D%AE%20Control_1-VS-Osteogenic_Medium_Treat_1.htm#gene22) | 5 (0.92%) | 0.1648132 | 1.000000000 | ko04978 |
| 23 | [RNA degradation](file:///E:\01%20%E5%8D%9A%E5%A3%AB%E8%AE%BA%E6%96%87%E6%92%B0%E5%86%99%202016-2-1\1%20%E7%AC%AC%E4%B8%80%E9%83%A8%E5%88%86%20%EF%BC%88%E4%B8%89%EF%BC%89BMSCs%E4%B8%8E%E7%89%99%E8%83%9A%E7%BB%86%E8%83%9E%E4%BD%93%E5%A4%96%E9%97%B4%E6%8E%A5%E5%85%B1%E5%9F%B9%E5%85%BB%E6%A8%A1%E5%9E%8B%E4%B8%AD%E7%9A%84%E8%BD%AC%E5%BD%95%E7%BB%84%E8%A1%A8%E8%BE%BE%E8%B0%B1\pathway%E6%95%B0%E6%8D%AE%20Control_1-VS-Osteogenic_Medium_Treat_1.htm#gene23) | 6 (1.1%) | 0.1654982 | 1.000000000 | ko03018 |
| 24 | [Arrhythmogenic right ventricular cardiomyopathy (ARVC)](file:///E:\01%20%E5%8D%9A%E5%A3%AB%E8%AE%BA%E6%96%87%E6%92%B0%E5%86%99%202016-2-1\1%20%E7%AC%AC%E4%B8%80%E9%83%A8%E5%88%86%20%EF%BC%88%E4%B8%89%EF%BC%89BMSCs%E4%B8%8E%E7%89%99%E8%83%9A%E7%BB%86%E8%83%9E%E4%BD%93%E5%A4%96%E9%97%B4%E6%8E%A5%E5%85%B1%E5%9F%B9%E5%85%BB%E6%A8%A1%E5%9E%8B%E4%B8%AD%E7%9A%84%E8%BD%AC%E5%BD%95%E7%BB%84%E8%A1%A8%E8%BE%BE%E8%B0%B1\pathway%E6%95%B0%E6%8D%AE%20Control_1-VS-Osteogenic_Medium_Treat_1.htm#gene24) | 7 (1.28%) | 0.1688721 | 1.000000000 | ko05412 |
| 25 | [Vibrio cholerae infection](file:///E:\01%20%E5%8D%9A%E5%A3%AB%E8%AE%BA%E6%96%87%E6%92%B0%E5%86%99%202016-2-1\1%20%E7%AC%AC%E4%B8%80%E9%83%A8%E5%88%86%20%EF%BC%88%E4%B8%89%EF%BC%89BMSCs%E4%B8%8E%E7%89%99%E8%83%9A%E7%BB%86%E8%83%9E%E4%BD%93%E5%A4%96%E9%97%B4%E6%8E%A5%E5%85%B1%E5%9F%B9%E5%85%BB%E6%A8%A1%E5%9E%8B%E4%B8%AD%E7%9A%84%E8%BD%AC%E5%BD%95%E7%BB%84%E8%A1%A8%E8%BE%BE%E8%B0%B1\pathway%E6%95%B0%E6%8D%AE%20Control_1-VS-Osteogenic_Medium_Treat_1.htm#gene25) | 6 (1.1%) | 0.1712906 | 1.000000000 | ko05110 |
| 26 | [Lysine degradation](file:///E:\01%20%E5%8D%9A%E5%A3%AB%E8%AE%BA%E6%96%87%E6%92%B0%E5%86%99%202016-2-1\1%20%E7%AC%AC%E4%B8%80%E9%83%A8%E5%88%86%20%EF%BC%88%E4%B8%89%EF%BC%89BMSCs%E4%B8%8E%E7%89%99%E8%83%9A%E7%BB%86%E8%83%9E%E4%BD%93%E5%A4%96%E9%97%B4%E6%8E%A5%E5%85%B1%E5%9F%B9%E5%85%BB%E6%A8%A1%E5%9E%8B%E4%B8%AD%E7%9A%84%E8%BD%AC%E5%BD%95%E7%BB%84%E8%A1%A8%E8%BE%BE%E8%B0%B1\pathway%E6%95%B0%E6%8D%AE%20Control_1-VS-Osteogenic_Medium_Treat_1.htm#gene26) | 5 (0.92%) | 0.171407 | 1.000000000 | ko00310 |
| 27 | [SNARE interactions in vesicular transport](file:///E:\01%20%E5%8D%9A%E5%A3%AB%E8%AE%BA%E6%96%87%E6%92%B0%E5%86%99%202016-2-1\1%20%E7%AC%AC%E4%B8%80%E9%83%A8%E5%88%86%20%EF%BC%88%E4%B8%89%EF%BC%89BMSCs%E4%B8%8E%E7%89%99%E8%83%9A%E7%BB%86%E8%83%9E%E4%BD%93%E5%A4%96%E9%97%B4%E6%8E%A5%E5%85%B1%E5%9F%B9%E5%85%BB%E6%A8%A1%E5%9E%8B%E4%B8%AD%E7%9A%84%E8%BD%AC%E5%BD%95%E7%BB%84%E8%A1%A8%E8%BE%BE%E8%B0%B1\pathway%E6%95%B0%E6%8D%AE%20Control_1-VS-Osteogenic_Medium_Treat_1.htm#gene27) | 3 (0.55%) | 0.1812842 | 1.000000000 | ko04130 |
| 28 | [Phagosome](file:///E:\01%20%E5%8D%9A%E5%A3%AB%E8%AE%BA%E6%96%87%E6%92%B0%E5%86%99%202016-2-1\1%20%E7%AC%AC%E4%B8%80%E9%83%A8%E5%88%86%20%EF%BC%88%E4%B8%89%EF%BC%89BMSCs%E4%B8%8E%E7%89%99%E8%83%9A%E7%BB%86%E8%83%9E%E4%BD%93%E5%A4%96%E9%97%B4%E6%8E%A5%E5%85%B1%E5%9F%B9%E5%85%BB%E6%A8%A1%E5%9E%8B%E4%B8%AD%E7%9A%84%E8%BD%AC%E5%BD%95%E7%BB%84%E8%A1%A8%E8%BE%BE%E8%B0%B1\pathway%E6%95%B0%E6%8D%AE%20Control_1-VS-Osteogenic_Medium_Treat_1.htm#gene28) | 15 (2.75%) | 0.1882802 | 1.000000000 | ko04145 |
| 29 | [Tight junction](file:///E:\01%20%E5%8D%9A%E5%A3%AB%E8%AE%BA%E6%96%87%E6%92%B0%E5%86%99%202016-2-1\1%20%E7%AC%AC%E4%B8%80%E9%83%A8%E5%88%86%20%EF%BC%88%E4%B8%89%EF%BC%89BMSCs%E4%B8%8E%E7%89%99%E8%83%9A%E7%BB%86%E8%83%9E%E4%BD%93%E5%A4%96%E9%97%B4%E6%8E%A5%E5%85%B1%E5%9F%B9%E5%85%BB%E6%A8%A1%E5%9E%8B%E4%B8%AD%E7%9A%84%E8%BD%AC%E5%BD%95%E7%BB%84%E8%A1%A8%E8%BE%BE%E8%B0%B1\pathway%E6%95%B0%E6%8D%AE%20Control_1-VS-Osteogenic_Medium_Treat_1.htm#gene29) | 16 (2.94%) | 0.193494 | 1.000000000 | ko04530 |
| 30 | [Wnt signaling pathway](file:///E:\01%20%E5%8D%9A%E5%A3%AB%E8%AE%BA%E6%96%87%E6%92%B0%E5%86%99%202016-2-1\1%20%E7%AC%AC%E4%B8%80%E9%83%A8%E5%88%86%20%EF%BC%88%E4%B8%89%EF%BC%89BMSCs%E4%B8%8E%E7%89%99%E8%83%9A%E7%BB%86%E8%83%9E%E4%BD%93%E5%A4%96%E9%97%B4%E6%8E%A5%E5%85%B1%E5%9F%B9%E5%85%BB%E6%A8%A1%E5%9E%8B%E4%B8%AD%E7%9A%84%E8%BD%AC%E5%BD%95%E7%BB%84%E8%A1%A8%E8%BE%BE%E8%B0%B1\pathway%E6%95%B0%E6%8D%AE%20Control_1-VS-Osteogenic_Medium_Treat_1.htm#gene30) | 11 (2.02%) | 0.2030046 | 1.000000000 | ko04310 |
| 31 | [D-Arginine and D-ornithine metabolism](file:///E:\01%20%E5%8D%9A%E5%A3%AB%E8%AE%BA%E6%96%87%E6%92%B0%E5%86%99%202016-2-1\1%20%E7%AC%AC%E4%B8%80%E9%83%A8%E5%88%86%20%EF%BC%88%E4%B8%89%EF%BC%89BMSCs%E4%B8%8E%E7%89%99%E8%83%9A%E7%BB%86%E8%83%9E%E4%BD%93%E5%A4%96%E9%97%B4%E6%8E%A5%E5%85%B1%E5%9F%B9%E5%85%BB%E6%A8%A1%E5%9E%8B%E4%B8%AD%E7%9A%84%E8%BD%AC%E5%BD%95%E7%BB%84%E8%A1%A8%E8%BE%BE%E8%B0%B1\pathway%E6%95%B0%E6%8D%AE%20Control_1-VS-Osteogenic_Medium_Treat_1.htm#gene31) | 1 (0.18%) | 0.2162486 | 1.000000000 | ko00472 |
| 32 | [Protein digestion and absorption](file:///E:\01%20%E5%8D%9A%E5%A3%AB%E8%AE%BA%E6%96%87%E6%92%B0%E5%86%99%202016-2-1\1%20%E7%AC%AC%E4%B8%80%E9%83%A8%E5%88%86%20%EF%BC%88%E4%B8%89%EF%BC%89BMSCs%E4%B8%8E%E7%89%99%E8%83%9A%E7%BB%86%E8%83%9E%E4%BD%93%E5%A4%96%E9%97%B4%E6%8E%A5%E5%85%B1%E5%9F%B9%E5%85%BB%E6%A8%A1%E5%9E%8B%E4%B8%AD%E7%9A%84%E8%BD%AC%E5%BD%95%E7%BB%84%E8%A1%A8%E8%BE%BE%E8%B0%B1\pathway%E6%95%B0%E6%8D%AE%20Control_1-VS-Osteogenic_Medium_Treat_1.htm#gene32) | 6 (1.1%) | 0.2268652 | 1.000000000 | ko04974 |
| 33 | [Pathogenic Escherichia coli infection](file:///E:\01%20%E5%8D%9A%E5%A3%AB%E8%AE%BA%E6%96%87%E6%92%B0%E5%86%99%202016-2-1\1%20%E7%AC%AC%E4%B8%80%E9%83%A8%E5%88%86%20%EF%BC%88%E4%B8%89%EF%BC%89BMSCs%E4%B8%8E%E7%89%99%E8%83%9A%E7%BB%86%E8%83%9E%E4%BD%93%E5%A4%96%E9%97%B4%E6%8E%A5%E5%85%B1%E5%9F%B9%E5%85%BB%E6%A8%A1%E5%9E%8B%E4%B8%AD%E7%9A%84%E8%BD%AC%E5%BD%95%E7%BB%84%E8%A1%A8%E8%BE%BE%E8%B0%B1\pathway%E6%95%B0%E6%8D%AE%20Control_1-VS-Osteogenic_Medium_Treat_1.htm#gene33) | 9 (1.65%) | 0.231451 | 1.000000000 | ko05130 |
| 34 | [Fanconi anemia pathway](file:///E:\01%20%E5%8D%9A%E5%A3%AB%E8%AE%BA%E6%96%87%E6%92%B0%E5%86%99%202016-2-1\1%20%E7%AC%AC%E4%B8%80%E9%83%A8%E5%88%86%20%EF%BC%88%E4%B8%89%EF%BC%89BMSCs%E4%B8%8E%E7%89%99%E8%83%9A%E7%BB%86%E8%83%9E%E4%BD%93%E5%A4%96%E9%97%B4%E6%8E%A5%E5%85%B1%E5%9F%B9%E5%85%BB%E6%A8%A1%E5%9E%8B%E4%B8%AD%E7%9A%84%E8%BD%AC%E5%BD%95%E7%BB%84%E8%A1%A8%E8%BE%BE%E8%B0%B1\pathway%E6%95%B0%E6%8D%AE%20Control_1-VS-Osteogenic_Medium_Treat_1.htm#gene34) | 3 (0.55%) | 0.2332209 | 1.000000000 | ko03460 |
| 35 | [Phototransduction - fly](file:///E:\01%20%E5%8D%9A%E5%A3%AB%E8%AE%BA%E6%96%87%E6%92%B0%E5%86%99%202016-2-1\1%20%E7%AC%AC%E4%B8%80%E9%83%A8%E5%88%86%20%EF%BC%88%E4%B8%89%EF%BC%89BMSCs%E4%B8%8E%E7%89%99%E8%83%9A%E7%BB%86%E8%83%9E%E4%BD%93%E5%A4%96%E9%97%B4%E6%8E%A5%E5%85%B1%E5%9F%B9%E5%85%BB%E6%A8%A1%E5%9E%8B%E4%B8%AD%E7%9A%84%E8%BD%AC%E5%BD%95%E7%BB%84%E8%A1%A8%E8%BE%BE%E8%B0%B1\pathway%E6%95%B0%E6%8D%AE%20Control_1-VS-Osteogenic_Medium_Treat_1.htm#gene35) | 4 (0.73%) | 0.2414011 | 1.000000000 | ko04745 |
| 36 | [Cell cycle](file:///E:\01%20%E5%8D%9A%E5%A3%AB%E8%AE%BA%E6%96%87%E6%92%B0%E5%86%99%202016-2-1\1%20%E7%AC%AC%E4%B8%80%E9%83%A8%E5%88%86%20%EF%BC%88%E4%B8%89%EF%BC%89BMSCs%E4%B8%8E%E7%89%99%E8%83%9A%E7%BB%86%E8%83%9E%E4%BD%93%E5%A4%96%E9%97%B4%E6%8E%A5%E5%85%B1%E5%9F%B9%E5%85%BB%E6%A8%A1%E5%9E%8B%E4%B8%AD%E7%9A%84%E8%BD%AC%E5%BD%95%E7%BB%84%E8%A1%A8%E8%BE%BE%E8%B0%B1\pathway%E6%95%B0%E6%8D%AE%20Control_1-VS-Osteogenic_Medium_Treat_1.htm#gene36) | 8 (1.47%) | 0.2479521 | 1.000000000 | ko04110 |
| 37 | [Systemic lupus erythematosus](file:///E:\01%20%E5%8D%9A%E5%A3%AB%E8%AE%BA%E6%96%87%E6%92%B0%E5%86%99%202016-2-1\1%20%E7%AC%AC%E4%B8%80%E9%83%A8%E5%88%86%20%EF%BC%88%E4%B8%89%EF%BC%89BMSCs%E4%B8%8E%E7%89%99%E8%83%9A%E7%BB%86%E8%83%9E%E4%BD%93%E5%A4%96%E9%97%B4%E6%8E%A5%E5%85%B1%E5%9F%B9%E5%85%BB%E6%A8%A1%E5%9E%8B%E4%B8%AD%E7%9A%84%E8%BD%AC%E5%BD%95%E7%BB%84%E8%A1%A8%E8%BE%BE%E8%B0%B1\pathway%E6%95%B0%E6%8D%AE%20Control_1-VS-Osteogenic_Medium_Treat_1.htm#gene37) | 6 (1.1%) | 0.253245 | 1.000000000 | ko05322 |
| 38 | [Alzheimer's disease](file:///E:\01%20%E5%8D%9A%E5%A3%AB%E8%AE%BA%E6%96%87%E6%92%B0%E5%86%99%202016-2-1\1%20%E7%AC%AC%E4%B8%80%E9%83%A8%E5%88%86%20%EF%BC%88%E4%B8%89%EF%BC%89BMSCs%E4%B8%8E%E7%89%99%E8%83%9A%E7%BB%86%E8%83%9E%E4%BD%93%E5%A4%96%E9%97%B4%E6%8E%A5%E5%85%B1%E5%9F%B9%E5%85%BB%E6%A8%A1%E5%9E%8B%E4%B8%AD%E7%9A%84%E8%BD%AC%E5%BD%95%E7%BB%84%E8%A1%A8%E8%BE%BE%E8%B0%B1\pathway%E6%95%B0%E6%8D%AE%20Control_1-VS-Osteogenic_Medium_Treat_1.htm#gene38) | 10 (1.83%) | 0.2645268 | 1.000000000 | ko05010 |
| 39 | [Gap junction](file:///E:\01%20%E5%8D%9A%E5%A3%AB%E8%AE%BA%E6%96%87%E6%92%B0%E5%86%99%202016-2-1\1%20%E7%AC%AC%E4%B8%80%E9%83%A8%E5%88%86%20%EF%BC%88%E4%B8%89%EF%BC%89BMSCs%E4%B8%8E%E7%89%99%E8%83%9A%E7%BB%86%E8%83%9E%E4%BD%93%E5%A4%96%E9%97%B4%E6%8E%A5%E5%85%B1%E5%9F%B9%E5%85%BB%E6%A8%A1%E5%9E%8B%E4%B8%AD%E7%9A%84%E8%BD%AC%E5%BD%95%E7%BB%84%E8%A1%A8%E8%BE%BE%E8%B0%B1\pathway%E6%95%B0%E6%8D%AE%20Control_1-VS-Osteogenic_Medium_Treat_1.htm#gene39) | 6 (1.1%) | 0.2667427 | 1.000000000 | ko04540 |
| 40 | [Basal transcription factors](file:///E:\01%20%E5%8D%9A%E5%A3%AB%E8%AE%BA%E6%96%87%E6%92%B0%E5%86%99%202016-2-1\1%20%E7%AC%AC%E4%B8%80%E9%83%A8%E5%88%86%20%EF%BC%88%E4%B8%89%EF%BC%89BMSCs%E4%B8%8E%E7%89%99%E8%83%9A%E7%BB%86%E8%83%9E%E4%BD%93%E5%A4%96%E9%97%B4%E6%8E%A5%E5%85%B1%E5%9F%B9%E5%85%BB%E6%A8%A1%E5%9E%8B%E4%B8%AD%E7%9A%84%E8%BD%AC%E5%BD%95%E7%BB%84%E8%A1%A8%E8%BE%BE%E8%B0%B1\pathway%E6%95%B0%E6%8D%AE%20Control_1-VS-Osteogenic_Medium_Treat_1.htm#gene40) | 3 (0.55%) | 0.2764412 | 1.000000000 | ko03022 |
| 41 | [Notch signaling pathway](file:///E:\01%20%E5%8D%9A%E5%A3%AB%E8%AE%BA%E6%96%87%E6%92%B0%E5%86%99%202016-2-1\1%20%E7%AC%AC%E4%B8%80%E9%83%A8%E5%88%86%20%EF%BC%88%E4%B8%89%EF%BC%89BMSCs%E4%B8%8E%E7%89%99%E8%83%9A%E7%BB%86%E8%83%9E%E4%BD%93%E5%A4%96%E9%97%B4%E6%8E%A5%E5%85%B1%E5%9F%B9%E5%85%BB%E6%A8%A1%E5%9E%8B%E4%B8%AD%E7%9A%84%E8%BD%AC%E5%BD%95%E7%BB%84%E8%A1%A8%E8%BE%BE%E8%B0%B1\pathway%E6%95%B0%E6%8D%AE%20Control_1-VS-Osteogenic_Medium_Treat_1.htm#gene41) | 4 (0.73%) | 0.2766635 | 1.000000000 | ko04330 |
| 42 | [Lipoic acid metabolism](file:///E:\01%20%E5%8D%9A%E5%A3%AB%E8%AE%BA%E6%96%87%E6%92%B0%E5%86%99%202016-2-1\1%20%E7%AC%AC%E4%B8%80%E9%83%A8%E5%88%86%20%EF%BC%88%E4%B8%89%EF%BC%89BMSCs%E4%B8%8E%E7%89%99%E8%83%9A%E7%BB%86%E8%83%9E%E4%BD%93%E5%A4%96%E9%97%B4%E6%8E%A5%E5%85%B1%E5%9F%B9%E5%85%BB%E6%A8%A1%E5%9E%8B%E4%B8%AD%E7%9A%84%E8%BD%AC%E5%BD%95%E7%BB%84%E8%A1%A8%E8%BE%BE%E8%B0%B1\pathway%E6%95%B0%E6%8D%AE%20Control_1-VS-Osteogenic_Medium_Treat_1.htm#gene42) | 1 (0.18%) | 0.2774067 | 1.000000000 | ko00785 |
| 43 | [Renin-angiotensin system](file:///E:\01%20%E5%8D%9A%E5%A3%AB%E8%AE%BA%E6%96%87%E6%92%B0%E5%86%99%202016-2-1\1%20%E7%AC%AC%E4%B8%80%E9%83%A8%E5%88%86%20%EF%BC%88%E4%B8%89%EF%BC%89BMSCs%E4%B8%8E%E7%89%99%E8%83%9A%E7%BB%86%E8%83%9E%E4%BD%93%E5%A4%96%E9%97%B4%E6%8E%A5%E5%85%B1%E5%9F%B9%E5%85%BB%E6%A8%A1%E5%9E%8B%E4%B8%AD%E7%9A%84%E8%BD%AC%E5%BD%95%E7%BB%84%E8%A1%A8%E8%BE%BE%E8%B0%B1\pathway%E6%95%B0%E6%8D%AE%20Control_1-VS-Osteogenic_Medium_Treat_1.htm#gene43) | 2 (0.37%) | 0.2920837 | 1.000000000 | ko04614 |
| 44 | [Adherens junction](file:///E:\01%20%E5%8D%9A%E5%A3%AB%E8%AE%BA%E6%96%87%E6%92%B0%E5%86%99%202016-2-1\1%20%E7%AC%AC%E4%B8%80%E9%83%A8%E5%88%86%20%EF%BC%88%E4%B8%89%EF%BC%89BMSCs%E4%B8%8E%E7%89%99%E8%83%9A%E7%BB%86%E8%83%9E%E4%BD%93%E5%A4%96%E9%97%B4%E6%8E%A5%E5%85%B1%E5%9F%B9%E5%85%BB%E6%A8%A1%E5%9E%8B%E4%B8%AD%E7%9A%84%E8%BD%AC%E5%BD%95%E7%BB%84%E8%A1%A8%E8%BE%BE%E8%B0%B1\pathway%E6%95%B0%E6%8D%AE%20Control_1-VS-Osteogenic_Medium_Treat_1.htm#gene44) | 7 (1.28%) | 0.2977014 | 1.000000000 | ko04520 |
| 45 | [Caffeine metabolism](file:///E:\01%20%E5%8D%9A%E5%A3%AB%E8%AE%BA%E6%96%87%E6%92%B0%E5%86%99%202016-2-1\1%20%E7%AC%AC%E4%B8%80%E9%83%A8%E5%88%86%20%EF%BC%88%E4%B8%89%EF%BC%89BMSCs%E4%B8%8E%E7%89%99%E8%83%9A%E7%BB%86%E8%83%9E%E4%BD%93%E5%A4%96%E9%97%B4%E6%8E%A5%E5%85%B1%E5%9F%B9%E5%85%BB%E6%A8%A1%E5%9E%8B%E4%B8%AD%E7%9A%84%E8%BD%AC%E5%BD%95%E7%BB%84%E8%A1%A8%E8%BE%BE%E8%B0%B1\pathway%E6%95%B0%E6%8D%AE%20Control_1-VS-Osteogenic_Medium_Treat_1.htm#gene45) | 1 (0.18%) | 0.3061753 | 1.000000000 | ko00232 |
| 46 | [Peroxisome](file:///E:\01%20%E5%8D%9A%E5%A3%AB%E8%AE%BA%E6%96%87%E6%92%B0%E5%86%99%202016-2-1\1%20%E7%AC%AC%E4%B8%80%E9%83%A8%E5%88%86%20%EF%BC%88%E4%B8%89%EF%BC%89BMSCs%E4%B8%8E%E7%89%99%E8%83%9A%E7%BB%86%E8%83%9E%E4%BD%93%E5%A4%96%E9%97%B4%E6%8E%A5%E5%85%B1%E5%9F%B9%E5%85%BB%E6%A8%A1%E5%9E%8B%E4%B8%AD%E7%9A%84%E8%BD%AC%E5%BD%95%E7%BB%84%E8%A1%A8%E8%BE%BE%E8%B0%B1\pathway%E6%95%B0%E6%8D%AE%20Control_1-VS-Osteogenic_Medium_Treat_1.htm#gene46) | 6 (1.1%) | 0.3222771 | 1.000000000 | ko04146 |
| 47 | [Amyotrophic lateral sclerosis (ALS)](file:///E:\01%20%E5%8D%9A%E5%A3%AB%E8%AE%BA%E6%96%87%E6%92%B0%E5%86%99%202016-2-1\1%20%E7%AC%AC%E4%B8%80%E9%83%A8%E5%88%86%20%EF%BC%88%E4%B8%89%EF%BC%89BMSCs%E4%B8%8E%E7%89%99%E8%83%9A%E7%BB%86%E8%83%9E%E4%BD%93%E5%A4%96%E9%97%B4%E6%8E%A5%E5%85%B1%E5%9F%B9%E5%85%BB%E6%A8%A1%E5%9E%8B%E4%B8%AD%E7%9A%84%E8%BD%AC%E5%BD%95%E7%BB%84%E8%A1%A8%E8%BE%BE%E8%B0%B1\pathway%E6%95%B0%E6%8D%AE%20Control_1-VS-Osteogenic_Medium_Treat_1.htm#gene47) | 5 (0.92%) | 0.3271921 | 1.000000000 | ko05014 |
| 48 | [Glycolysis / Gluconeogenesis](file:///E:\01%20%E5%8D%9A%E5%A3%AB%E8%AE%BA%E6%96%87%E6%92%B0%E5%86%99%202016-2-1\1%20%E7%AC%AC%E4%B8%80%E9%83%A8%E5%88%86%20%EF%BC%88%E4%B8%89%EF%BC%89BMSCs%E4%B8%8E%E7%89%99%E8%83%9A%E7%BB%86%E8%83%9E%E4%BD%93%E5%A4%96%E9%97%B4%E6%8E%A5%E5%85%B1%E5%9F%B9%E5%85%BB%E6%A8%A1%E5%9E%8B%E4%B8%AD%E7%9A%84%E8%BD%AC%E5%BD%95%E7%BB%84%E8%A1%A8%E8%BE%BE%E8%B0%B1\pathway%E6%95%B0%E6%8D%AE%20Control_1-VS-Osteogenic_Medium_Treat_1.htm#gene48) | 4 (0.73%) | 0.3308646 | 1.000000000 | ko00010 |
| 49 | [Herpes simplex infection](file:///E:\01%20%E5%8D%9A%E5%A3%AB%E8%AE%BA%E6%96%87%E6%92%B0%E5%86%99%202016-2-1\1%20%E7%AC%AC%E4%B8%80%E9%83%A8%E5%88%86%20%EF%BC%88%E4%B8%89%EF%BC%89BMSCs%E4%B8%8E%E7%89%99%E8%83%9A%E7%BB%86%E8%83%9E%E4%BD%93%E5%A4%96%E9%97%B4%E6%8E%A5%E5%85%B1%E5%9F%B9%E5%85%BB%E6%A8%A1%E5%9E%8B%E4%B8%AD%E7%9A%84%E8%BD%AC%E5%BD%95%E7%BB%84%E8%A1%A8%E8%BE%BE%E8%B0%B1\pathway%E6%95%B0%E6%8D%AE%20Control_1-VS-Osteogenic_Medium_Treat_1.htm#gene49) | 12 (2.2%) | 0.3311675 | 1.000000000 | ko05168 |
| 50 | [Homologous recombination](file:///E:\01%20%E5%8D%9A%E5%A3%AB%E8%AE%BA%E6%96%87%E6%92%B0%E5%86%99%202016-2-1\1%20%E7%AC%AC%E4%B8%80%E9%83%A8%E5%88%86%20%EF%BC%88%E4%B8%89%EF%BC%89BMSCs%E4%B8%8E%E7%89%99%E8%83%9A%E7%BB%86%E8%83%9E%E4%BD%93%E5%A4%96%E9%97%B4%E6%8E%A5%E5%85%B1%E5%9F%B9%E5%85%BB%E6%A8%A1%E5%9E%8B%E4%B8%AD%E7%9A%84%E8%BD%AC%E5%BD%95%E7%BB%84%E8%A1%A8%E8%BE%BE%E8%B0%B1\pathway%E6%95%B0%E6%8D%AE%20Control_1-VS-Osteogenic_Medium_Treat_1.htm#gene50) | 2 (0.37%) | 0.3365485 | 1.000000000 | ko03440 |
| 51 | [Alcoholism](file:///E:\01%20%E5%8D%9A%E5%A3%AB%E8%AE%BA%E6%96%87%E6%92%B0%E5%86%99%202016-2-1\1%20%E7%AC%AC%E4%B8%80%E9%83%A8%E5%88%86%20%EF%BC%88%E4%B8%89%EF%BC%89BMSCs%E4%B8%8E%E7%89%99%E8%83%9A%E7%BB%86%E8%83%9E%E4%BD%93%E5%A4%96%E9%97%B4%E6%8E%A5%E5%85%B1%E5%9F%B9%E5%85%BB%E6%A8%A1%E5%9E%8B%E4%B8%AD%E7%9A%84%E8%BD%AC%E5%BD%95%E7%BB%84%E8%A1%A8%E8%BE%BE%E8%B0%B1\pathway%E6%95%B0%E6%8D%AE%20Control_1-VS-Osteogenic_Medium_Treat_1.htm#gene51) | 8 (1.47%) | 0.347072 | 1.000000000 | ko05034 |
| 52 | [Oxidative phosphorylation](file:///E:\01%20%E5%8D%9A%E5%A3%AB%E8%AE%BA%E6%96%87%E6%92%B0%E5%86%99%202016-2-1\1%20%E7%AC%AC%E4%B8%80%E9%83%A8%E5%88%86%20%EF%BC%88%E4%B8%89%EF%BC%89BMSCs%E4%B8%8E%E7%89%99%E8%83%9A%E7%BB%86%E8%83%9E%E4%BD%93%E5%A4%96%E9%97%B4%E6%8E%A5%E5%85%B1%E5%9F%B9%E5%85%BB%E6%A8%A1%E5%9E%8B%E4%B8%AD%E7%9A%84%E8%BD%AC%E5%BD%95%E7%BB%84%E8%A1%A8%E8%BE%BE%E8%B0%B1\pathway%E6%95%B0%E6%8D%AE%20Control_1-VS-Osteogenic_Medium_Treat_1.htm#gene52) | 6 (1.1%) | 0.3506686 | 1.000000000 | ko00190 |
| 53 | [Legionellosis](file:///E:\01%20%E5%8D%9A%E5%A3%AB%E8%AE%BA%E6%96%87%E6%92%B0%E5%86%99%202016-2-1\1%20%E7%AC%AC%E4%B8%80%E9%83%A8%E5%88%86%20%EF%BC%88%E4%B8%89%EF%BC%89BMSCs%E4%B8%8E%E7%89%99%E8%83%9A%E7%BB%86%E8%83%9E%E4%BD%93%E5%A4%96%E9%97%B4%E6%8E%A5%E5%85%B1%E5%9F%B9%E5%85%BB%E6%A8%A1%E5%9E%8B%E4%B8%AD%E7%9A%84%E8%BD%AC%E5%BD%95%E7%BB%84%E8%A1%A8%E8%BE%BE%E8%B0%B1\pathway%E6%95%B0%E6%8D%AE%20Control_1-VS-Osteogenic_Medium_Treat_1.htm#gene53) | 4 (0.73%) | 0.3673387 | 1.000000000 | ko05134 |
| 54 | [mTOR signaling pathway](file:///E:\01%20%E5%8D%9A%E5%A3%AB%E8%AE%BA%E6%96%87%E6%92%B0%E5%86%99%202016-2-1\1%20%E7%AC%AC%E4%B8%80%E9%83%A8%E5%88%86%20%EF%BC%88%E4%B8%89%EF%BC%89BMSCs%E4%B8%8E%E7%89%99%E8%83%9A%E7%BB%86%E8%83%9E%E4%BD%93%E5%A4%96%E9%97%B4%E6%8E%A5%E5%85%B1%E5%9F%B9%E5%85%BB%E6%A8%A1%E5%9E%8B%E4%B8%AD%E7%9A%84%E8%BD%AC%E5%BD%95%E7%BB%84%E8%A1%A8%E8%BE%BE%E8%B0%B1\pathway%E6%95%B0%E6%8D%AE%20Control_1-VS-Osteogenic_Medium_Treat_1.htm#gene54) | 4 (0.73%) | 0.3764517 | 1.000000000 | ko04150 |
| 55 | [Synthesis and degradation of ketone bodies](file:///E:\01%20%E5%8D%9A%E5%A3%AB%E8%AE%BA%E6%96%87%E6%92%B0%E5%86%99%202016-2-1\1%20%E7%AC%AC%E4%B8%80%E9%83%A8%E5%88%86%20%EF%BC%88%E4%B8%89%EF%BC%89BMSCs%E4%B8%8E%E7%89%99%E8%83%9A%E7%BB%86%E8%83%9E%E4%BD%93%E5%A4%96%E9%97%B4%E6%8E%A5%E5%85%B1%E5%9F%B9%E5%85%BB%E6%A8%A1%E5%9E%8B%E4%B8%AD%E7%9A%84%E8%BD%AC%E5%BD%95%E7%BB%84%E8%A1%A8%E8%BE%BE%E8%B0%B1\pathway%E6%95%B0%E6%8D%AE%20Control_1-VS-Osteogenic_Medium_Treat_1.htm#gene55) | 1 (0.18%) | 0.3858006 | 1.000000000 | ko00072 |
| 56 | [Shigellosis](file:///E:\01%20%E5%8D%9A%E5%A3%AB%E8%AE%BA%E6%96%87%E6%92%B0%E5%86%99%202016-2-1\1%20%E7%AC%AC%E4%B8%80%E9%83%A8%E5%88%86%20%EF%BC%88%E4%B8%89%EF%BC%89BMSCs%E4%B8%8E%E7%89%99%E8%83%9A%E7%BB%86%E8%83%9E%E4%BD%93%E5%A4%96%E9%97%B4%E6%8E%A5%E5%85%B1%E5%9F%B9%E5%85%BB%E6%A8%A1%E5%9E%8B%E4%B8%AD%E7%9A%84%E8%BD%AC%E5%BD%95%E7%BB%84%E8%A1%A8%E8%BE%BE%E8%B0%B1\pathway%E6%95%B0%E6%8D%AE%20Control_1-VS-Osteogenic_Medium_Treat_1.htm#gene56) | 6 (1.1%) | 0.3864109 | 1.000000000 | ko05131 |
| 57 | [Biosynthesis of unsaturated fatty acids](file:///E:\01%20%E5%8D%9A%E5%A3%AB%E8%AE%BA%E6%96%87%E6%92%B0%E5%86%99%202016-2-1\1%20%E7%AC%AC%E4%B8%80%E9%83%A8%E5%88%86%20%EF%BC%88%E4%B8%89%EF%BC%89BMSCs%E4%B8%8E%E7%89%99%E8%83%9A%E7%BB%86%E8%83%9E%E4%BD%93%E5%A4%96%E9%97%B4%E6%8E%A5%E5%85%B1%E5%9F%B9%E5%85%BB%E6%A8%A1%E5%9E%8B%E4%B8%AD%E7%9A%84%E8%BD%AC%E5%BD%95%E7%BB%84%E8%A1%A8%E8%BE%BE%E8%B0%B1\pathway%E6%95%B0%E6%8D%AE%20Control_1-VS-Osteogenic_Medium_Treat_1.htm#gene57) | 2 (0.37%) | 0.4085299 | 1.000000000 | ko01040 |
| 58 | [Basal cell carcinoma](file:///E:\01%20%E5%8D%9A%E5%A3%AB%E8%AE%BA%E6%96%87%E6%92%B0%E5%86%99%202016-2-1\1%20%E7%AC%AC%E4%B8%80%E9%83%A8%E5%88%86%20%EF%BC%88%E4%B8%89%EF%BC%89BMSCs%E4%B8%8E%E7%89%99%E8%83%9A%E7%BB%86%E8%83%9E%E4%BD%93%E5%A4%96%E9%97%B4%E6%8E%A5%E5%85%B1%E5%9F%B9%E5%85%BB%E6%A8%A1%E5%9E%8B%E4%B8%AD%E7%9A%84%E8%BD%AC%E5%BD%95%E7%BB%84%E8%A1%A8%E8%BE%BE%E8%B0%B1\pathway%E6%95%B0%E6%8D%AE%20Control_1-VS-Osteogenic_Medium_Treat_1.htm#gene58) | 3 (0.55%) | 0.4185657 | 1.000000000 | ko05217 |
| 59 | [Amphetamine addiction](file:///E:\01%20%E5%8D%9A%E5%A3%AB%E8%AE%BA%E6%96%87%E6%92%B0%E5%86%99%202016-2-1\1%20%E7%AC%AC%E4%B8%80%E9%83%A8%E5%88%86%20%EF%BC%88%E4%B8%89%EF%BC%89BMSCs%E4%B8%8E%E7%89%99%E8%83%9A%E7%BB%86%E8%83%9E%E4%BD%93%E5%A4%96%E9%97%B4%E6%8E%A5%E5%85%B1%E5%9F%B9%E5%85%BB%E6%A8%A1%E5%9E%8B%E4%B8%AD%E7%9A%84%E8%BD%AC%E5%BD%95%E7%BB%84%E8%A1%A8%E8%BE%BE%E8%B0%B1\pathway%E6%95%B0%E6%8D%AE%20Control_1-VS-Osteogenic_Medium_Treat_1.htm#gene59) | 5 (0.92%) | 0.4303926 | 1.000000000 | ko05031 |
| 60 | [Riboflavin metabolism](file:///E:\01%20%E5%8D%9A%E5%A3%AB%E8%AE%BA%E6%96%87%E6%92%B0%E5%86%99%202016-2-1\1%20%E7%AC%AC%E4%B8%80%E9%83%A8%E5%88%86%20%EF%BC%88%E4%B8%89%EF%BC%89BMSCs%E4%B8%8E%E7%89%99%E8%83%9A%E7%BB%86%E8%83%9E%E4%BD%93%E5%A4%96%E9%97%B4%E6%8E%A5%E5%85%B1%E5%9F%B9%E5%85%BB%E6%A8%A1%E5%9E%8B%E4%B8%AD%E7%9A%84%E8%BD%AC%E5%BD%95%E7%BB%84%E8%A1%A8%E8%BE%BE%E8%B0%B1\pathway%E6%95%B0%E6%8D%AE%20Control_1-VS-Osteogenic_Medium_Treat_1.htm#gene60) | 1 (0.18%) | 0.4337487 | 1.000000000 | ko00740 |
| 61 | [Parkinson's disease](file:///E:\01%20%E5%8D%9A%E5%A3%AB%E8%AE%BA%E6%96%87%E6%92%B0%E5%86%99%202016-2-1\1%20%E7%AC%AC%E4%B8%80%E9%83%A8%E5%88%86%20%EF%BC%88%E4%B8%89%EF%BC%89BMSCs%E4%B8%8E%E7%89%99%E8%83%9A%E7%BB%86%E8%83%9E%E4%BD%93%E5%A4%96%E9%97%B4%E6%8E%A5%E5%85%B1%E5%9F%B9%E5%85%BB%E6%A8%A1%E5%9E%8B%E4%B8%AD%E7%9A%84%E8%BD%AC%E5%BD%95%E7%BB%84%E8%A1%A8%E8%BE%BE%E8%B0%B1\pathway%E6%95%B0%E6%8D%AE%20Control_1-VS-Osteogenic_Medium_Treat_1.htm#gene61) | 6 (1.1%) | 0.4363781 | 1.000000000 | ko05012 |
| 62 | [Galactose metabolism](file:///E:\01%20%E5%8D%9A%E5%A3%AB%E8%AE%BA%E6%96%87%E6%92%B0%E5%86%99%202016-2-1\1%20%E7%AC%AC%E4%B8%80%E9%83%A8%E5%88%86%20%EF%BC%88%E4%B8%89%EF%BC%89BMSCs%E4%B8%8E%E7%89%99%E8%83%9A%E7%BB%86%E8%83%9E%E4%BD%93%E5%A4%96%E9%97%B4%E6%8E%A5%E5%85%B1%E5%9F%B9%E5%85%BB%E6%A8%A1%E5%9E%8B%E4%B8%AD%E7%9A%84%E8%BD%AC%E5%BD%95%E7%BB%84%E8%A1%A8%E8%BE%BE%E8%B0%B1\pathway%E6%95%B0%E6%8D%AE%20Control_1-VS-Osteogenic_Medium_Treat_1.htm#gene62) | 2 (0.37%) | 0.4498702 | 1.000000000 | ko00052 |
| 63 | [MAPK signaling pathway](file:///E:\01%20%E5%8D%9A%E5%A3%AB%E8%AE%BA%E6%96%87%E6%92%B0%E5%86%99%202016-2-1\1%20%E7%AC%AC%E4%B8%80%E9%83%A8%E5%88%86%20%EF%BC%88%E4%B8%89%EF%BC%89BMSCs%E4%B8%8E%E7%89%99%E8%83%9A%E7%BB%86%E8%83%9E%E4%BD%93%E5%A4%96%E9%97%B4%E6%8E%A5%E5%85%B1%E5%9F%B9%E5%85%BB%E6%A8%A1%E5%9E%8B%E4%B8%AD%E7%9A%84%E8%BD%AC%E5%BD%95%E7%BB%84%E8%A1%A8%E8%BE%BE%E8%B0%B1\pathway%E6%95%B0%E6%8D%AE%20Control_1-VS-Osteogenic_Medium_Treat_1.htm#gene63) | 15 (2.75%) | 0.4519353 | 1.000000000 | ko04010 |
| 64 | [Epithelial cell signaling in Helicobacter pylori infection](file:///E:\01%20%E5%8D%9A%E5%A3%AB%E8%AE%BA%E6%96%87%E6%92%B0%E5%86%99%202016-2-1\1%20%E7%AC%AC%E4%B8%80%E9%83%A8%E5%88%86%20%EF%BC%88%E4%B8%89%EF%BC%89BMSCs%E4%B8%8E%E7%89%99%E8%83%9A%E7%BB%86%E8%83%9E%E4%BD%93%E5%A4%96%E9%97%B4%E6%8E%A5%E5%85%B1%E5%9F%B9%E5%85%BB%E6%A8%A1%E5%9E%8B%E4%B8%AD%E7%9A%84%E8%BD%AC%E5%BD%95%E7%BB%84%E8%A1%A8%E8%BE%BE%E8%B0%B1\pathway%E6%95%B0%E6%8D%AE%20Control_1-VS-Osteogenic_Medium_Treat_1.htm#gene64) | 4 (0.73%) | 0.4574061 | 1.000000000 | ko05120 |
| 65 | [Aminoacyl-tRNA biosynthesis](file:///E:\01%20%E5%8D%9A%E5%A3%AB%E8%AE%BA%E6%96%87%E6%92%B0%E5%86%99%202016-2-1\1%20%E7%AC%AC%E4%B8%80%E9%83%A8%E5%88%86%20%EF%BC%88%E4%B8%89%EF%BC%89BMSCs%E4%B8%8E%E7%89%99%E8%83%9A%E7%BB%86%E8%83%9E%E4%BD%93%E5%A4%96%E9%97%B4%E6%8E%A5%E5%85%B1%E5%9F%B9%E5%85%BB%E6%A8%A1%E5%9E%8B%E4%B8%AD%E7%9A%84%E8%BD%AC%E5%BD%95%E7%BB%84%E8%A1%A8%E8%BE%BE%E8%B0%B1\pathway%E6%95%B0%E6%8D%AE%20Control_1-VS-Osteogenic_Medium_Treat_1.htm#gene65) | 3 (0.55%) | 0.4608297 | 1.000000000 | ko00970 |
| 66 | [Regulation of actin cytoskeleton](file:///E:\01%20%E5%8D%9A%E5%A3%AB%E8%AE%BA%E6%96%87%E6%92%B0%E5%86%99%202016-2-1\1%20%E7%AC%AC%E4%B8%80%E9%83%A8%E5%88%86%20%EF%BC%88%E4%B8%89%EF%BC%89BMSCs%E4%B8%8E%E7%89%99%E8%83%9A%E7%BB%86%E8%83%9E%E4%BD%93%E5%A4%96%E9%97%B4%E6%8E%A5%E5%85%B1%E5%9F%B9%E5%85%BB%E6%A8%A1%E5%9E%8B%E4%B8%AD%E7%9A%84%E8%BD%AC%E5%BD%95%E7%BB%84%E8%A1%A8%E8%BE%BE%E8%B0%B1\pathway%E6%95%B0%E6%8D%AE%20Control_1-VS-Osteogenic_Medium_Treat_1.htm#gene66) | 17 (3.12%) | 0.4620995 | 1.000000000 | ko04810 |
| 67 | [p53 signaling pathway](file:///E:\01%20%E5%8D%9A%E5%A3%AB%E8%AE%BA%E6%96%87%E6%92%B0%E5%86%99%202016-2-1\1%20%E7%AC%AC%E4%B8%80%E9%83%A8%E5%88%86%20%EF%BC%88%E4%B8%89%EF%BC%89BMSCs%E4%B8%8E%E7%89%99%E8%83%9A%E7%BB%86%E8%83%9E%E4%BD%93%E5%A4%96%E9%97%B4%E6%8E%A5%E5%85%B1%E5%9F%B9%E5%85%BB%E6%A8%A1%E5%9E%8B%E4%B8%AD%E7%9A%84%E8%BD%AC%E5%BD%95%E7%BB%84%E8%A1%A8%E8%BE%BE%E8%B0%B1\pathway%E6%95%B0%E6%8D%AE%20Control_1-VS-Osteogenic_Medium_Treat_1.htm#gene67) | 5 (0.92%) | 0.4694568 | 1.000000000 | ko04115 |
| 68 | [GnRH signaling pathway](file:///E:\01%20%E5%8D%9A%E5%A3%AB%E8%AE%BA%E6%96%87%E6%92%B0%E5%86%99%202016-2-1\1%20%E7%AC%AC%E4%B8%80%E9%83%A8%E5%88%86%20%EF%BC%88%E4%B8%89%EF%BC%89BMSCs%E4%B8%8E%E7%89%99%E8%83%9A%E7%BB%86%E8%83%9E%E4%BD%93%E5%A4%96%E9%97%B4%E6%8E%A5%E5%85%B1%E5%9F%B9%E5%85%BB%E6%A8%A1%E5%9E%8B%E4%B8%AD%E7%9A%84%E8%BD%AC%E5%BD%95%E7%BB%84%E8%A1%A8%E8%BE%BE%E8%B0%B1\pathway%E6%95%B0%E6%8D%AE%20Control_1-VS-Osteogenic_Medium_Treat_1.htm#gene68) | 6 (1.1%) | 0.4716398 | 1.000000000 | ko04912 |
| 69 | [Epstein-Barr virus infection](file:///E:\01%20%E5%8D%9A%E5%A3%AB%E8%AE%BA%E6%96%87%E6%92%B0%E5%86%99%202016-2-1\1%20%E7%AC%AC%E4%B8%80%E9%83%A8%E5%88%86%20%EF%BC%88%E4%B8%89%EF%BC%89BMSCs%E4%B8%8E%E7%89%99%E8%83%9A%E7%BB%86%E8%83%9E%E4%BD%93%E5%A4%96%E9%97%B4%E6%8E%A5%E5%85%B1%E5%9F%B9%E5%85%BB%E6%A8%A1%E5%9E%8B%E4%B8%AD%E7%9A%84%E8%BD%AC%E5%BD%95%E7%BB%84%E8%A1%A8%E8%BE%BE%E8%B0%B1\pathway%E6%95%B0%E6%8D%AE%20Control_1-VS-Osteogenic_Medium_Treat_1.htm#gene69) | 13 (2.39%) | 0.4774277 | 1.000000000 | ko05169 |
| 70 | [Progesterone-mediated oocyte maturation](file:///E:\01%20%E5%8D%9A%E5%A3%AB%E8%AE%BA%E6%96%87%E6%92%B0%E5%86%99%202016-2-1\1%20%E7%AC%AC%E4%B8%80%E9%83%A8%E5%88%86%20%EF%BC%88%E4%B8%89%EF%BC%89BMSCs%E4%B8%8E%E7%89%99%E8%83%9A%E7%BB%86%E8%83%9E%E4%BD%93%E5%A4%96%E9%97%B4%E6%8E%A5%E5%85%B1%E5%9F%B9%E5%85%BB%E6%A8%A1%E5%9E%8B%E4%B8%AD%E7%9A%84%E8%BD%AC%E5%BD%95%E7%BB%84%E8%A1%A8%E8%BE%BE%E8%B0%B1\pathway%E6%95%B0%E6%8D%AE%20Control_1-VS-Osteogenic_Medium_Treat_1.htm#gene70) | 5 (0.92%) | 0.4848549 | 1.000000000 | ko04914 |
| 71 | [Gastric acid secretion](file:///E:\01%20%E5%8D%9A%E5%A3%AB%E8%AE%BA%E6%96%87%E6%92%B0%E5%86%99%202016-2-1\1%20%E7%AC%AC%E4%B8%80%E9%83%A8%E5%88%86%20%EF%BC%88%E4%B8%89%EF%BC%89BMSCs%E4%B8%8E%E7%89%99%E8%83%9A%E7%BB%86%E8%83%9E%E4%BD%93%E5%A4%96%E9%97%B4%E6%8E%A5%E5%85%B1%E5%9F%B9%E5%85%BB%E6%A8%A1%E5%9E%8B%E4%B8%AD%E7%9A%84%E8%BD%AC%E5%BD%95%E7%BB%84%E8%A1%A8%E8%BE%BE%E8%B0%B1\pathway%E6%95%B0%E6%8D%AE%20Control_1-VS-Osteogenic_Medium_Treat_1.htm#gene71) | 6 (1.1%) | 0.4855786 | 1.000000000 | ko04971 |
| 72 | [Proximal tubule bicarbonate reclamation](file:///E:\01%20%E5%8D%9A%E5%A3%AB%E8%AE%BA%E6%96%87%E6%92%B0%E5%86%99%202016-2-1\1%20%E7%AC%AC%E4%B8%80%E9%83%A8%E5%88%86%20%EF%BC%88%E4%B8%89%EF%BC%89BMSCs%E4%B8%8E%E7%89%99%E8%83%9A%E7%BB%86%E8%83%9E%E4%BD%93%E5%A4%96%E9%97%B4%E6%8E%A5%E5%85%B1%E5%9F%B9%E5%85%BB%E6%A8%A1%E5%9E%8B%E4%B8%AD%E7%9A%84%E8%BD%AC%E5%BD%95%E7%BB%84%E8%A1%A8%E8%BE%BE%E8%B0%B1\pathway%E6%95%B0%E6%8D%AE%20Control_1-VS-Osteogenic_Medium_Treat_1.htm#gene72) | 2 (0.37%) | 0.489526 | 1.000000000 | ko04964 |
| 73 | [Dilated cardiomyopathy](file:///E:\01%20%E5%8D%9A%E5%A3%AB%E8%AE%BA%E6%96%87%E6%92%B0%E5%86%99%202016-2-1\1%20%E7%AC%AC%E4%B8%80%E9%83%A8%E5%88%86%20%EF%BC%88%E4%B8%89%EF%BC%89BMSCs%E4%B8%8E%E7%89%99%E8%83%9A%E7%BB%86%E8%83%9E%E4%BD%93%E5%A4%96%E9%97%B4%E6%8E%A5%E5%85%B1%E5%9F%B9%E5%85%BB%E6%A8%A1%E5%9E%8B%E4%B8%AD%E7%9A%84%E8%BD%AC%E5%BD%95%E7%BB%84%E8%A1%A8%E8%BE%BE%E8%B0%B1\pathway%E6%95%B0%E6%8D%AE%20Control_1-VS-Osteogenic_Medium_Treat_1.htm#gene73) | 9 (1.65%) | 0.4977085 | 1.000000000 | ko05414 |
| 74 | [Neurotrophin signaling pathway](file:///E:\01%20%E5%8D%9A%E5%A3%AB%E8%AE%BA%E6%96%87%E6%92%B0%E5%86%99%202016-2-1\1%20%E7%AC%AC%E4%B8%80%E9%83%A8%E5%88%86%20%EF%BC%88%E4%B8%89%EF%BC%89BMSCs%E4%B8%8E%E7%89%99%E8%83%9A%E7%BB%86%E8%83%9E%E4%BD%93%E5%A4%96%E9%97%B4%E6%8E%A5%E5%85%B1%E5%9F%B9%E5%85%BB%E6%A8%A1%E5%9E%8B%E4%B8%AD%E7%9A%84%E8%BD%AC%E5%BD%95%E7%BB%84%E8%A1%A8%E8%BE%BE%E8%B0%B1\pathway%E6%95%B0%E6%8D%AE%20Control_1-VS-Osteogenic_Medium_Treat_1.htm#gene74) | 8 (1.47%) | 0.4982202 | 1.000000000 | ko04722 |
| 75 | [Sulfur metabolism](file:///E:\01%20%E5%8D%9A%E5%A3%AB%E8%AE%BA%E6%96%87%E6%92%B0%E5%86%99%202016-2-1\1%20%E7%AC%AC%E4%B8%80%E9%83%A8%E5%88%86%20%EF%BC%88%E4%B8%89%EF%BC%89BMSCs%E4%B8%8E%E7%89%99%E8%83%9A%E7%BB%86%E8%83%9E%E4%BD%93%E5%A4%96%E9%97%B4%E6%8E%A5%E5%85%B1%E5%9F%B9%E5%85%BB%E6%A8%A1%E5%9E%8B%E4%B8%AD%E7%9A%84%E8%BD%AC%E5%BD%95%E7%BB%84%E8%A1%A8%E8%BE%BE%E8%B0%B1\pathway%E6%95%B0%E6%8D%AE%20Control_1-VS-Osteogenic_Medium_Treat_1.htm#gene75) | 1 (0.18%) | 0.4987562 | 1.000000000 | ko00920 |
| 76 | [Primary immunodeficiency](file:///E:\01%20%E5%8D%9A%E5%A3%AB%E8%AE%BA%E6%96%87%E6%92%B0%E5%86%99%202016-2-1\1%20%E7%AC%AC%E4%B8%80%E9%83%A8%E5%88%86%20%EF%BC%88%E4%B8%89%EF%BC%89BMSCs%E4%B8%8E%E7%89%99%E8%83%9A%E7%BB%86%E8%83%9E%E4%BD%93%E5%A4%96%E9%97%B4%E6%8E%A5%E5%85%B1%E5%9F%B9%E5%85%BB%E6%A8%A1%E5%9E%8B%E4%B8%AD%E7%9A%84%E8%BD%AC%E5%BD%95%E7%BB%84%E8%A1%A8%E8%BE%BE%E8%B0%B1\pathway%E6%95%B0%E6%8D%AE%20Control_1-VS-Osteogenic_Medium_Treat_1.htm#gene76) | 2 (0.37%) | 0.5023413 | 1.000000000 | ko05340 |
| 77 | [Calcium signaling pathway](file:///E:\01%20%E5%8D%9A%E5%A3%AB%E8%AE%BA%E6%96%87%E6%92%B0%E5%86%99%202016-2-1\1%20%E7%AC%AC%E4%B8%80%E9%83%A8%E5%88%86%20%EF%BC%88%E4%B8%89%EF%BC%89BMSCs%E4%B8%8E%E7%89%99%E8%83%9A%E7%BB%86%E8%83%9E%E4%BD%93%E5%A4%96%E9%97%B4%E6%8E%A5%E5%85%B1%E5%9F%B9%E5%85%BB%E6%A8%A1%E5%9E%8B%E4%B8%AD%E7%9A%84%E8%BD%AC%E5%BD%95%E7%BB%84%E8%A1%A8%E8%BE%BE%E8%B0%B1\pathway%E6%95%B0%E6%8D%AE%20Control_1-VS-Osteogenic_Medium_Treat_1.htm#gene77) | 9 (1.65%) | 0.5032744 | 1.000000000 | ko04020 |
| 78 | [Valine, leucine and isoleucine degradation](file:///E:\01%20%E5%8D%9A%E5%A3%AB%E8%AE%BA%E6%96%87%E6%92%B0%E5%86%99%202016-2-1\1%20%E7%AC%AC%E4%B8%80%E9%83%A8%E5%88%86%20%EF%BC%88%E4%B8%89%EF%BC%89BMSCs%E4%B8%8E%E7%89%99%E8%83%9A%E7%BB%86%E8%83%9E%E4%BD%93%E5%A4%96%E9%97%B4%E6%8E%A5%E5%85%B1%E5%9F%B9%E5%85%BB%E6%A8%A1%E5%9E%8B%E4%B8%AD%E7%9A%84%E8%BD%AC%E5%BD%95%E7%BB%84%E8%A1%A8%E8%BE%BE%E8%B0%B1\pathway%E6%95%B0%E6%8D%AE%20Control_1-VS-Osteogenic_Medium_Treat_1.htm#gene78) | 3 (0.55%) | 0.5116912 | 1.000000000 | ko00280 |
| 79 | [Propanoate metabolism](file:///E:\01%20%E5%8D%9A%E5%A3%AB%E8%AE%BA%E6%96%87%E6%92%B0%E5%86%99%202016-2-1\1%20%E7%AC%AC%E4%B8%80%E9%83%A8%E5%88%86%20%EF%BC%88%E4%B8%89%EF%BC%89BMSCs%E4%B8%8E%E7%89%99%E8%83%9A%E7%BB%86%E8%83%9E%E4%BD%93%E5%A4%96%E9%97%B4%E6%8E%A5%E5%85%B1%E5%9F%B9%E5%85%BB%E6%A8%A1%E5%9E%8B%E4%B8%AD%E7%9A%84%E8%BD%AC%E5%BD%95%E7%BB%84%E8%A1%A8%E8%BE%BE%E8%B0%B1\pathway%E6%95%B0%E6%8D%AE%20Control_1-VS-Osteogenic_Medium_Treat_1.htm#gene79) | 2 (0.37%) | 0.5149471 | 1.000000000 | ko00640 |
| 80 | [Selenocompound metabolism](file:///E:\01%20%E5%8D%9A%E5%A3%AB%E8%AE%BA%E6%96%87%E6%92%B0%E5%86%99%202016-2-1\1%20%E7%AC%AC%E4%B8%80%E9%83%A8%E5%88%86%20%EF%BC%88%E4%B8%89%EF%BC%89BMSCs%E4%B8%8E%E7%89%99%E8%83%9A%E7%BB%86%E8%83%9E%E4%BD%93%E5%A4%96%E9%97%B4%E6%8E%A5%E5%85%B1%E5%9F%B9%E5%85%BB%E6%A8%A1%E5%9E%8B%E4%B8%AD%E7%9A%84%E8%BD%AC%E5%BD%95%E7%BB%84%E8%A1%A8%E8%BE%BE%E8%B0%B1\pathway%E6%95%B0%E6%8D%AE%20Control_1-VS-Osteogenic_Medium_Treat_1.htm#gene80) | 1 (0.18%) | 0.5187253 | 1.000000000 | ko00450 |
| 81 | [One carbon pool by folate](file:///E:\01%20%E5%8D%9A%E5%A3%AB%E8%AE%BA%E6%96%87%E6%92%B0%E5%86%99%202016-2-1\1%20%E7%AC%AC%E4%B8%80%E9%83%A8%E5%88%86%20%EF%BC%88%E4%B8%89%EF%BC%89BMSCs%E4%B8%8E%E7%89%99%E8%83%9A%E7%BB%86%E8%83%9E%E4%BD%93%E5%A4%96%E9%97%B4%E6%8E%A5%E5%85%B1%E5%9F%B9%E5%85%BB%E6%A8%A1%E5%9E%8B%E4%B8%AD%E7%9A%84%E8%BD%AC%E5%BD%95%E7%BB%84%E8%A1%A8%E8%BE%BE%E8%B0%B1\pathway%E6%95%B0%E6%8D%AE%20Control_1-VS-Osteogenic_Medium_Treat_1.htm#gene81) | 1 (0.18%) | 0.5187253 | 1.000000000 | ko00670 |
| 82 | [Insulin signaling pathway](file:///E:\01%20%E5%8D%9A%E5%A3%AB%E8%AE%BA%E6%96%87%E6%92%B0%E5%86%99%202016-2-1\1%20%E7%AC%AC%E4%B8%80%E9%83%A8%E5%88%86%20%EF%BC%88%E4%B8%89%EF%BC%89BMSCs%E4%B8%8E%E7%89%99%E8%83%9A%E7%BB%86%E8%83%9E%E4%BD%93%E5%A4%96%E9%97%B4%E6%8E%A5%E5%85%B1%E5%9F%B9%E5%85%BB%E6%A8%A1%E5%9E%8B%E4%B8%AD%E7%9A%84%E8%BD%AC%E5%BD%95%E7%BB%84%E8%A1%A8%E8%BE%BE%E8%B0%B1\pathway%E6%95%B0%E6%8D%AE%20Control_1-VS-Osteogenic_Medium_Treat_1.htm#gene82) | 8 (1.47%) | 0.5217353 | 1.000000000 | ko04910 |
| 83 | [Nicotine addiction](file:///E:\01%20%E5%8D%9A%E5%A3%AB%E8%AE%BA%E6%96%87%E6%92%B0%E5%86%99%202016-2-1\1%20%E7%AC%AC%E4%B8%80%E9%83%A8%E5%88%86%20%EF%BC%88%E4%B8%89%EF%BC%89BMSCs%E4%B8%8E%E7%89%99%E8%83%9A%E7%BB%86%E8%83%9E%E4%BD%93%E5%A4%96%E9%97%B4%E6%8E%A5%E5%85%B1%E5%9F%B9%E5%85%BB%E6%A8%A1%E5%9E%8B%E4%B8%AD%E7%9A%84%E8%BD%AC%E5%BD%95%E7%BB%84%E8%A1%A8%E8%BE%BE%E8%B0%B1\pathway%E6%95%B0%E6%8D%AE%20Control_1-VS-Osteogenic_Medium_Treat_1.htm#gene83) | 2 (0.37%) | 0.5395163 | 1.000000000 | ko05033 |
| 84 | [Vitamin digestion and absorption](file:///E:\01%20%E5%8D%9A%E5%A3%AB%E8%AE%BA%E6%96%87%E6%92%B0%E5%86%99%202016-2-1\1%20%E7%AC%AC%E4%B8%80%E9%83%A8%E5%88%86%20%EF%BC%88%E4%B8%89%EF%BC%89BMSCs%E4%B8%8E%E7%89%99%E8%83%9A%E7%BB%86%E8%83%9E%E4%BD%93%E5%A4%96%E9%97%B4%E6%8E%A5%E5%85%B1%E5%9F%B9%E5%85%BB%E6%A8%A1%E5%9E%8B%E4%B8%AD%E7%9A%84%E8%BD%AC%E5%BD%95%E7%BB%84%E8%A1%A8%E8%BE%BE%E8%B0%B1\pathway%E6%95%B0%E6%8D%AE%20Control_1-VS-Osteogenic_Medium_Treat_1.htm#gene84) | 2 (0.37%) | 0.5395163 | 1.000000000 | ko04977 |
| 85 | [Base excision repair](file:///E:\01%20%E5%8D%9A%E5%A3%AB%E8%AE%BA%E6%96%87%E6%92%B0%E5%86%99%202016-2-1\1%20%E7%AC%AC%E4%B8%80%E9%83%A8%E5%88%86%20%EF%BC%88%E4%B8%89%EF%BC%89BMSCs%E4%B8%8E%E7%89%99%E8%83%9A%E7%BB%86%E8%83%9E%E4%BD%93%E5%A4%96%E9%97%B4%E6%8E%A5%E5%85%B1%E5%9F%B9%E5%85%BB%E6%A8%A1%E5%9E%8B%E4%B8%AD%E7%9A%84%E8%BD%AC%E5%BD%95%E7%BB%84%E8%A1%A8%E8%BE%BE%E8%B0%B1\pathway%E6%95%B0%E6%8D%AE%20Control_1-VS-Osteogenic_Medium_Treat_1.htm#gene85) | 2 (0.37%) | 0.5395163 | 1.000000000 | ko03410 |
| 86 | [Butanoate metabolism](file:///E:\01%20%E5%8D%9A%E5%A3%AB%E8%AE%BA%E6%96%87%E6%92%B0%E5%86%99%202016-2-1\1%20%E7%AC%AC%E4%B8%80%E9%83%A8%E5%88%86%20%EF%BC%88%E4%B8%89%EF%BC%89BMSCs%E4%B8%8E%E7%89%99%E8%83%9A%E7%BB%86%E8%83%9E%E4%BD%93%E5%A4%96%E9%97%B4%E6%8E%A5%E5%85%B1%E5%9F%B9%E5%85%BB%E6%A8%A1%E5%9E%8B%E4%B8%AD%E7%9A%84%E8%BD%AC%E5%BD%95%E7%BB%84%E8%A1%A8%E8%BE%BE%E8%B0%B1\pathway%E6%95%B0%E6%8D%AE%20Control_1-VS-Osteogenic_Medium_Treat_1.htm#gene86) | 2 (0.37%) | 0.5395163 | 1.000000000 | ko00650 |
| 87 | [Cocaine addiction](file:///E:\01%20%E5%8D%9A%E5%A3%AB%E8%AE%BA%E6%96%87%E6%92%B0%E5%86%99%202016-2-1\1%20%E7%AC%AC%E4%B8%80%E9%83%A8%E5%88%86%20%EF%BC%88%E4%B8%89%EF%BC%89BMSCs%E4%B8%8E%E7%89%99%E8%83%9A%E7%BB%86%E8%83%9E%E4%BD%93%E5%A4%96%E9%97%B4%E6%8E%A5%E5%85%B1%E5%9F%B9%E5%85%BB%E6%A8%A1%E5%9E%8B%E4%B8%AD%E7%9A%84%E8%BD%AC%E5%BD%95%E7%BB%84%E8%A1%A8%E8%BE%BE%E8%B0%B1\pathway%E6%95%B0%E6%8D%AE%20Control_1-VS-Osteogenic_Medium_Treat_1.htm#gene87) | 3 (0.55%) | 0.5409623 | 1.000000000 | ko05030 |
| 88 | [Osteoclast differentiation](file:///E:\01%20%E5%8D%9A%E5%A3%AB%E8%AE%BA%E6%96%87%E6%92%B0%E5%86%99%202016-2-1\1%20%E7%AC%AC%E4%B8%80%E9%83%A8%E5%88%86%20%EF%BC%88%E4%B8%89%EF%BC%89BMSCs%E4%B8%8E%E7%89%99%E8%83%9A%E7%BB%86%E8%83%9E%E4%BD%93%E5%A4%96%E9%97%B4%E6%8E%A5%E5%85%B1%E5%9F%B9%E5%85%BB%E6%A8%A1%E5%9E%8B%E4%B8%AD%E7%9A%84%E8%BD%AC%E5%BD%95%E7%BB%84%E8%A1%A8%E8%BE%BE%E8%B0%B1\pathway%E6%95%B0%E6%8D%AE%20Control_1-VS-Osteogenic_Medium_Treat_1.htm#gene88) | 7 (1.28%) | 0.5425141 | 1.000000000 | ko04380 |
| 89 | [Melanogenesis](file:///E:\01%20%E5%8D%9A%E5%A3%AB%E8%AE%BA%E6%96%87%E6%92%B0%E5%86%99%202016-2-1\1%20%E7%AC%AC%E4%B8%80%E9%83%A8%E5%88%86%20%EF%BC%88%E4%B8%89%EF%BC%89BMSCs%E4%B8%8E%E7%89%99%E8%83%9A%E7%BB%86%E8%83%9E%E4%BD%93%E5%A4%96%E9%97%B4%E6%8E%A5%E5%85%B1%E5%9F%B9%E5%85%BB%E6%A8%A1%E5%9E%8B%E4%B8%AD%E7%9A%84%E8%BD%AC%E5%BD%95%E7%BB%84%E8%A1%A8%E8%BE%BE%E8%B0%B1\pathway%E6%95%B0%E6%8D%AE%20Control_1-VS-Osteogenic_Medium_Treat_1.htm#gene89) | 5 (0.92%) | 0.5446996 | 1.000000000 | ko04916 |
| 90 | [Glycosphingolipid biosynthesis - ganglio series](file:///E:\01%20%E5%8D%9A%E5%A3%AB%E8%AE%BA%E6%96%87%E6%92%B0%E5%86%99%202016-2-1\1%20%E7%AC%AC%E4%B8%80%E9%83%A8%E5%88%86%20%EF%BC%88%E4%B8%89%EF%BC%89BMSCs%E4%B8%8E%E7%89%99%E8%83%9A%E7%BB%86%E8%83%9E%E4%BD%93%E5%A4%96%E9%97%B4%E6%8E%A5%E5%85%B1%E5%9F%B9%E5%85%BB%E6%A8%A1%E5%9E%8B%E4%B8%AD%E7%9A%84%E8%BD%AC%E5%BD%95%E7%BB%84%E8%A1%A8%E8%BE%BE%E8%B0%B1\pathway%E6%95%B0%E6%8D%AE%20Control_1-VS-Osteogenic_Medium_Treat_1.htm#gene90) | 1 (0.18%) | 0.5563127 | 1.000000000 | ko00604 |
| 91 | [Pantothenate and CoA biosynthesis](file:///E:\01%20%E5%8D%9A%E5%A3%AB%E8%AE%BA%E6%96%87%E6%92%B0%E5%86%99%202016-2-1\1%20%E7%AC%AC%E4%B8%80%E9%83%A8%E5%88%86%20%EF%BC%88%E4%B8%89%EF%BC%89BMSCs%E4%B8%8E%E7%89%99%E8%83%9A%E7%BB%86%E8%83%9E%E4%BD%93%E5%A4%96%E9%97%B4%E6%8E%A5%E5%85%B1%E5%9F%B9%E5%85%BB%E6%A8%A1%E5%9E%8B%E4%B8%AD%E7%9A%84%E8%BD%AC%E5%BD%95%E7%BB%84%E8%A1%A8%E8%BE%BE%E8%B0%B1\pathway%E6%95%B0%E6%8D%AE%20Control_1-VS-Osteogenic_Medium_Treat_1.htm#gene91) | 1 (0.18%) | 0.5563127 | 1.000000000 | ko00770 |
| 92 | [Bile secretion](file:///E:\01%20%E5%8D%9A%E5%A3%AB%E8%AE%BA%E6%96%87%E6%92%B0%E5%86%99%202016-2-1\1%20%E7%AC%AC%E4%B8%80%E9%83%A8%E5%88%86%20%EF%BC%88%E4%B8%89%EF%BC%89BMSCs%E4%B8%8E%E7%89%99%E8%83%9A%E7%BB%86%E8%83%9E%E4%BD%93%E5%A4%96%E9%97%B4%E6%8E%A5%E5%85%B1%E5%9F%B9%E5%85%BB%E6%A8%A1%E5%9E%8B%E4%B8%AD%E7%9A%84%E8%BD%AC%E5%BD%95%E7%BB%84%E8%A1%A8%E8%BE%BE%E8%B0%B1\pathway%E6%95%B0%E6%8D%AE%20Control_1-VS-Osteogenic_Medium_Treat_1.htm#gene92) | 5 (0.92%) | 0.5591476 | 1.000000000 | ko04976 |
| 93 | [Dorso-ventral axis formation](file:///E:\01%20%E5%8D%9A%E5%A3%AB%E8%AE%BA%E6%96%87%E6%92%B0%E5%86%99%202016-2-1\1%20%E7%AC%AC%E4%B8%80%E9%83%A8%E5%88%86%20%EF%BC%88%E4%B8%89%EF%BC%89BMSCs%E4%B8%8E%E7%89%99%E8%83%9A%E7%BB%86%E8%83%9E%E4%BD%93%E5%A4%96%E9%97%B4%E6%8E%A5%E5%85%B1%E5%9F%B9%E5%85%BB%E6%A8%A1%E5%9E%8B%E4%B8%AD%E7%9A%84%E8%BD%AC%E5%BD%95%E7%BB%84%E8%A1%A8%E8%BE%BE%E8%B0%B1\pathway%E6%95%B0%E6%8D%AE%20Control_1-VS-Osteogenic_Medium_Treat_1.htm#gene93) | 2 (0.37%) | 0.5632117 | 1.000000000 | ko04320 |
| 94 | [Glioma](file:///E:\01%20%E5%8D%9A%E5%A3%AB%E8%AE%BA%E6%96%87%E6%92%B0%E5%86%99%202016-2-1\1%20%E7%AC%AC%E4%B8%80%E9%83%A8%E5%88%86%20%EF%BC%88%E4%B8%89%EF%BC%89BMSCs%E4%B8%8E%E7%89%99%E8%83%9A%E7%BB%86%E8%83%9E%E4%BD%93%E5%A4%96%E9%97%B4%E6%8E%A5%E5%85%B1%E5%9F%B9%E5%85%BB%E6%A8%A1%E5%9E%8B%E4%B8%AD%E7%9A%84%E8%BD%AC%E5%BD%95%E7%BB%84%E8%A1%A8%E8%BE%BE%E8%B0%B1\pathway%E6%95%B0%E6%8D%AE%20Control_1-VS-Osteogenic_Medium_Treat_1.htm#gene94) | 4 (0.73%) | 0.5670109 | 1.000000000 | ko05214 |
| 95 | [Arginine and proline metabolism](file:///E:\01%20%E5%8D%9A%E5%A3%AB%E8%AE%BA%E6%96%87%E6%92%B0%E5%86%99%202016-2-1\1%20%E7%AC%AC%E4%B8%80%E9%83%A8%E5%88%86%20%EF%BC%88%E4%B8%89%EF%BC%89BMSCs%E4%B8%8E%E7%89%99%E8%83%9A%E7%BB%86%E8%83%9E%E4%BD%93%E5%A4%96%E9%97%B4%E6%8E%A5%E5%85%B1%E5%9F%B9%E5%85%BB%E6%A8%A1%E5%9E%8B%E4%B8%AD%E7%9A%84%E8%BD%AC%E5%BD%95%E7%BB%84%E8%A1%A8%E8%BE%BE%E8%B0%B1\pathway%E6%95%B0%E6%8D%AE%20Control_1-VS-Osteogenic_Medium_Treat_1.htm#gene95) | 3 (0.55%) | 0.569198 | 1.000000000 | ko00330 |
| 96 | [Long-term potentiation](file:///E:\01%20%E5%8D%9A%E5%A3%AB%E8%AE%BA%E6%96%87%E6%92%B0%E5%86%99%202016-2-1\1%20%E7%AC%AC%E4%B8%80%E9%83%A8%E5%88%86%20%EF%BC%88%E4%B8%89%EF%BC%89BMSCs%E4%B8%8E%E7%89%99%E8%83%9A%E7%BB%86%E8%83%9E%E4%BD%93%E5%A4%96%E9%97%B4%E6%8E%A5%E5%85%B1%E5%9F%B9%E5%85%BB%E6%A8%A1%E5%9E%8B%E4%B8%AD%E7%9A%84%E8%BD%AC%E5%BD%95%E7%BB%84%E8%A1%A8%E8%BE%BE%E8%B0%B1\pathway%E6%95%B0%E6%8D%AE%20Control_1-VS-Osteogenic_Medium_Treat_1.htm#gene96) | 4 (0.73%) | 0.5749346 | 1.000000000 | ko04720 |
| 97 | [Influenza A](file:///E:\01%20%E5%8D%9A%E5%A3%AB%E8%AE%BA%E6%96%87%E6%92%B0%E5%86%99%202016-2-1\1%20%E7%AC%AC%E4%B8%80%E9%83%A8%E5%88%86%20%EF%BC%88%E4%B8%89%EF%BC%89BMSCs%E4%B8%8E%E7%89%99%E8%83%9A%E7%BB%86%E8%83%9E%E4%BD%93%E5%A4%96%E9%97%B4%E6%8E%A5%E5%85%B1%E5%9F%B9%E5%85%BB%E6%A8%A1%E5%9E%8B%E4%B8%AD%E7%9A%84%E8%BD%AC%E5%BD%95%E7%BB%84%E8%A1%A8%E8%BE%BE%E8%B0%B1\pathway%E6%95%B0%E6%8D%AE%20Control_1-VS-Osteogenic_Medium_Treat_1.htm#gene97) | 10 (1.83%) | 0.5794245 | 1.000000000 | ko05164 |
| 98 | [ABC transporters](file:///E:\01%20%E5%8D%9A%E5%A3%AB%E8%AE%BA%E6%96%87%E6%92%B0%E5%86%99%202016-2-1\1%20%E7%AC%AC%E4%B8%80%E9%83%A8%E5%88%86%20%EF%BC%88%E4%B8%89%EF%BC%89BMSCs%E4%B8%8E%E7%89%99%E8%83%9A%E7%BB%86%E8%83%9E%E4%BD%93%E5%A4%96%E9%97%B4%E6%8E%A5%E5%85%B1%E5%9F%B9%E5%85%BB%E6%A8%A1%E5%9E%8B%E4%B8%AD%E7%9A%84%E8%BD%AC%E5%BD%95%E7%BB%84%E8%A1%A8%E8%BE%BE%E8%B0%B1\pathway%E6%95%B0%E6%8D%AE%20Control_1-VS-Osteogenic_Medium_Treat_1.htm#gene98) | 2 (0.37%) | 0.58602 | 1.000000000 | ko02010 |
| 99 | [Prion diseases](file:///E:\01%20%E5%8D%9A%E5%A3%AB%E8%AE%BA%E6%96%87%E6%92%B0%E5%86%99%202016-2-1\1%20%E7%AC%AC%E4%B8%80%E9%83%A8%E5%88%86%20%EF%BC%88%E4%B8%89%EF%BC%89BMSCs%E4%B8%8E%E7%89%99%E8%83%9A%E7%BB%86%E8%83%9E%E4%BD%93%E5%A4%96%E9%97%B4%E6%8E%A5%E5%85%B1%E5%9F%B9%E5%85%BB%E6%A8%A1%E5%9E%8B%E4%B8%AD%E7%9A%84%E8%BD%AC%E5%BD%95%E7%BB%84%E8%A1%A8%E8%BE%BE%E8%B0%B1\pathway%E6%95%B0%E6%8D%AE%20Control_1-VS-Osteogenic_Medium_Treat_1.htm#gene99) | 3 (0.55%) | 0.6051337 | 1.000000000 | ko05020 |
| 100 | [Steroid biosynthesis](file:///E:\01%20%E5%8D%9A%E5%A3%AB%E8%AE%BA%E6%96%87%E6%92%B0%E5%86%99%202016-2-1\1%20%E7%AC%AC%E4%B8%80%E9%83%A8%E5%88%86%20%EF%BC%88%E4%B8%89%EF%BC%89BMSCs%E4%B8%8E%E7%89%99%E8%83%9A%E7%BB%86%E8%83%9E%E4%BD%93%E5%A4%96%E9%97%B4%E6%8E%A5%E5%85%B1%E5%9F%B9%E5%85%BB%E6%A8%A1%E5%9E%8B%E4%B8%AD%E7%9A%84%E8%BD%AC%E5%BD%95%E7%BB%84%E8%A1%A8%E8%BE%BE%E8%B0%B1\pathway%E6%95%B0%E6%8D%AE%20Control_1-VS-Osteogenic_Medium_Treat_1.htm#gene100) | 1 (0.18%) | 0.6072709 | 1.000000000 | ko00100 |
| 101 | [Ubiquitin mediated proteolysis](file:///E:\01%20%E5%8D%9A%E5%A3%AB%E8%AE%BA%E6%96%87%E6%92%B0%E5%86%99%202016-2-1\1%20%E7%AC%AC%E4%B8%80%E9%83%A8%E5%88%86%20%EF%BC%88%E4%B8%89%EF%BC%89BMSCs%E4%B8%8E%E7%89%99%E8%83%9A%E7%BB%86%E8%83%9E%E4%BD%93%E5%A4%96%E9%97%B4%E6%8E%A5%E5%85%B1%E5%9F%B9%E5%85%BB%E6%A8%A1%E5%9E%8B%E4%B8%AD%E7%9A%84%E8%BD%AC%E5%BD%95%E7%BB%84%E8%A1%A8%E8%BE%BE%E8%B0%B1\pathway%E6%95%B0%E6%8D%AE%20Control_1-VS-Osteogenic_Medium_Treat_1.htm#gene101) | 7 (1.28%) | 0.6191031 | 1.000000000 | ko04120 |
| 102 | [Circadian rhythm - mammal](file:///E:\01%20%E5%8D%9A%E5%A3%AB%E8%AE%BA%E6%96%87%E6%92%B0%E5%86%99%202016-2-1\1%20%E7%AC%AC%E4%B8%80%E9%83%A8%E5%88%86%20%EF%BC%88%E4%B8%89%EF%BC%89BMSCs%E4%B8%8E%E7%89%99%E8%83%9A%E7%BB%86%E8%83%9E%E4%BD%93%E5%A4%96%E9%97%B4%E6%8E%A5%E5%85%B1%E5%9F%B9%E5%85%BB%E6%A8%A1%E5%9E%8B%E4%B8%AD%E7%9A%84%E8%BD%AC%E5%BD%95%E7%BB%84%E8%A1%A8%E8%BE%BE%E8%B0%B1\pathway%E6%95%B0%E6%8D%AE%20Control_1-VS-Osteogenic_Medium_Treat_1.htm#gene102) | 1 (0.18%) | 0.6229237 | 1.000000000 | ko04710 |
| 103 | [Hypertrophic cardiomyopathy (HCM)](file:///E:\01%20%E5%8D%9A%E5%A3%AB%E8%AE%BA%E6%96%87%E6%92%B0%E5%86%99%202016-2-1\1%20%E7%AC%AC%E4%B8%80%E9%83%A8%E5%88%86%20%EF%BC%88%E4%B8%89%EF%BC%89BMSCs%E4%B8%8E%E7%89%99%E8%83%9A%E7%BB%86%E8%83%9E%E4%BD%93%E5%A4%96%E9%97%B4%E6%8E%A5%E5%85%B1%E5%9F%B9%E5%85%BB%E6%A8%A1%E5%9E%8B%E4%B8%AD%E7%9A%84%E8%BD%AC%E5%BD%95%E7%BB%84%E8%A1%A8%E8%BE%BE%E8%B0%B1\pathway%E6%95%B0%E6%8D%AE%20Control_1-VS-Osteogenic_Medium_Treat_1.htm#gene103) | 8 (1.47%) | 0.6270836 | 1.000000000 | ko05410 |
| 104 | [Morphine addiction](file:///E:\01%20%E5%8D%9A%E5%A3%AB%E8%AE%BA%E6%96%87%E6%92%B0%E5%86%99%202016-2-1\1%20%E7%AC%AC%E4%B8%80%E9%83%A8%E5%88%86%20%EF%BC%88%E4%B8%89%EF%BC%89BMSCs%E4%B8%8E%E7%89%99%E8%83%9A%E7%BB%86%E8%83%9E%E4%BD%93%E5%A4%96%E9%97%B4%E6%8E%A5%E5%85%B1%E5%9F%B9%E5%85%BB%E6%A8%A1%E5%9E%8B%E4%B8%AD%E7%9A%84%E8%BD%AC%E5%BD%95%E7%BB%84%E8%A1%A8%E8%BE%BE%E8%B0%B1\pathway%E6%95%B0%E6%8D%AE%20Control_1-VS-Osteogenic_Medium_Treat_1.htm#gene104) | 4 (0.73%) | 0.6280179 | 1.000000000 | ko05032 |
| 105 | [Type I diabetes mellitus](file:///E:\01%20%E5%8D%9A%E5%A3%AB%E8%AE%BA%E6%96%87%E6%92%B0%E5%86%99%202016-2-1\1%20%E7%AC%AC%E4%B8%80%E9%83%A8%E5%88%86%20%EF%BC%88%E4%B8%89%EF%BC%89BMSCs%E4%B8%8E%E7%89%99%E8%83%9A%E7%BB%86%E8%83%9E%E4%BD%93%E5%A4%96%E9%97%B4%E6%8E%A5%E5%85%B1%E5%9F%B9%E5%85%BB%E6%A8%A1%E5%9E%8B%E4%B8%AD%E7%9A%84%E8%BD%AC%E5%BD%95%E7%BB%84%E8%A1%A8%E8%BE%BE%E8%B0%B1\pathway%E6%95%B0%E6%8D%AE%20Control_1-VS-Osteogenic_Medium_Treat_1.htm#gene105) | 3 (0.55%) | 0.6471765 | 1.000000000 | ko04940 |
| 106 | [Glyoxylate and dicarboxylate metabolism](file:///E:\01%20%E5%8D%9A%E5%A3%AB%E8%AE%BA%E6%96%87%E6%92%B0%E5%86%99%202016-2-1\1%20%E7%AC%AC%E4%B8%80%E9%83%A8%E5%88%86%20%EF%BC%88%E4%B8%89%EF%BC%89BMSCs%E4%B8%8E%E7%89%99%E8%83%9A%E7%BB%86%E8%83%9E%E4%BD%93%E5%A4%96%E9%97%B4%E6%8E%A5%E5%85%B1%E5%9F%B9%E5%85%BB%E6%A8%A1%E5%9E%8B%E4%B8%AD%E7%9A%84%E8%BD%AC%E5%BD%95%E7%BB%84%E8%A1%A8%E8%BE%BE%E8%B0%B1\pathway%E6%95%B0%E6%8D%AE%20Control_1-VS-Osteogenic_Medium_Treat_1.htm#gene106) | 1 (0.18%) | 0.6523859 | 1.000000000 | ko00630 |
| 107 | [Hedgehog signaling pathway](file:///E:\01%20%E5%8D%9A%E5%A3%AB%E8%AE%BA%E6%96%87%E6%92%B0%E5%86%99%202016-2-1\1%20%E7%AC%AC%E4%B8%80%E9%83%A8%E5%88%86%20%EF%BC%88%E4%B8%89%EF%BC%89BMSCs%E4%B8%8E%E7%89%99%E8%83%9A%E7%BB%86%E8%83%9E%E4%BD%93%E5%A4%96%E9%97%B4%E6%8E%A5%E5%85%B1%E5%9F%B9%E5%85%BB%E6%A8%A1%E5%9E%8B%E4%B8%AD%E7%9A%84%E8%BD%AC%E5%BD%95%E7%BB%84%E8%A1%A8%E8%BE%BE%E8%B0%B1\pathway%E6%95%B0%E6%8D%AE%20Control_1-VS-Osteogenic_Medium_Treat_1.htm#gene107) | 2 (0.37%) | 0.6588305 | 1.000000000 | ko04340 |
| 108 | [Pyruvate metabolism](file:///E:\01%20%E5%8D%9A%E5%A3%AB%E8%AE%BA%E6%96%87%E6%92%B0%E5%86%99%202016-2-1\1%20%E7%AC%AC%E4%B8%80%E9%83%A8%E5%88%86%20%EF%BC%88%E4%B8%89%EF%BC%89BMSCs%E4%B8%8E%E7%89%99%E8%83%9A%E7%BB%86%E8%83%9E%E4%BD%93%E5%A4%96%E9%97%B4%E6%8E%A5%E5%85%B1%E5%9F%B9%E5%85%BB%E6%A8%A1%E5%9E%8B%E4%B8%AD%E7%9A%84%E8%BD%AC%E5%BD%95%E7%BB%84%E8%A1%A8%E8%BE%BE%E8%B0%B1\pathway%E6%95%B0%E6%8D%AE%20Control_1-VS-Osteogenic_Medium_Treat_1.htm#gene108) | 2 (0.37%) | 0.6588305 | 1.000000000 | ko00620 |
| 109 | [Vasopressin-regulated water reabsorption](file:///E:\01%20%E5%8D%9A%E5%A3%AB%E8%AE%BA%E6%96%87%E6%92%B0%E5%86%99%202016-2-1\1%20%E7%AC%AC%E4%B8%80%E9%83%A8%E5%88%86%20%EF%BC%88%E4%B8%89%EF%BC%89BMSCs%E4%B8%8E%E7%89%99%E8%83%9A%E7%BB%86%E8%83%9E%E4%BD%93%E5%A4%96%E9%97%B4%E6%8E%A5%E5%85%B1%E5%9F%B9%E5%85%BB%E6%A8%A1%E5%9E%8B%E4%B8%AD%E7%9A%84%E8%BD%AC%E5%BD%95%E7%BB%84%E8%A1%A8%E8%BE%BE%E8%B0%B1\pathway%E6%95%B0%E6%8D%AE%20Control_1-VS-Osteogenic_Medium_Treat_1.htm#gene109) | 2 (0.37%) | 0.6588305 | 1.000000000 | ko04962 |
| 110 | [Tryptophan metabolism](file:///E:\01%20%E5%8D%9A%E5%A3%AB%E8%AE%BA%E6%96%87%E6%92%B0%E5%86%99%202016-2-1\1%20%E7%AC%AC%E4%B8%80%E9%83%A8%E5%88%86%20%EF%BC%88%E4%B8%89%EF%BC%89BMSCs%E4%B8%8E%E7%89%99%E8%83%9A%E7%BB%86%E8%83%9E%E4%BD%93%E5%A4%96%E9%97%B4%E6%8E%A5%E5%85%B1%E5%9F%B9%E5%85%BB%E6%A8%A1%E5%9E%8B%E4%B8%AD%E7%9A%84%E8%BD%AC%E5%BD%95%E7%BB%84%E8%A1%A8%E8%BE%BE%E8%B0%B1\pathway%E6%95%B0%E6%8D%AE%20Control_1-VS-Osteogenic_Medium_Treat_1.htm#gene110) | 2 (0.37%) | 0.6683508 | 1.000000000 | ko00380 |
| 111 | [Starch and sucrose metabolism](file:///E:\01%20%E5%8D%9A%E5%A3%AB%E8%AE%BA%E6%96%87%E6%92%B0%E5%86%99%202016-2-1\1%20%E7%AC%AC%E4%B8%80%E9%83%A8%E5%88%86%20%EF%BC%88%E4%B8%89%EF%BC%89BMSCs%E4%B8%8E%E7%89%99%E8%83%9A%E7%BB%86%E8%83%9E%E4%BD%93%E5%A4%96%E9%97%B4%E6%8E%A5%E5%85%B1%E5%9F%B9%E5%85%BB%E6%A8%A1%E5%9E%8B%E4%B8%AD%E7%9A%84%E8%BD%AC%E5%BD%95%E7%BB%84%E8%A1%A8%E8%BE%BE%E8%B0%B1\pathway%E6%95%B0%E6%8D%AE%20Control_1-VS-Osteogenic_Medium_Treat_1.htm#gene111) | 2 (0.37%) | 0.6683508 | 1.000000000 | ko00500 |
| 112 | [Bladder cancer](file:///E:\01%20%E5%8D%9A%E5%A3%AB%E8%AE%BA%E6%96%87%E6%92%B0%E5%86%99%202016-2-1\1%20%E7%AC%AC%E4%B8%80%E9%83%A8%E5%88%86%20%EF%BC%88%E4%B8%89%EF%BC%89BMSCs%E4%B8%8E%E7%89%99%E8%83%9A%E7%BB%86%E8%83%9E%E4%BD%93%E5%A4%96%E9%97%B4%E6%8E%A5%E5%85%B1%E5%9F%B9%E5%85%BB%E6%A8%A1%E5%9E%8B%E4%B8%AD%E7%9A%84%E8%BD%AC%E5%BD%95%E7%BB%84%E8%A1%A8%E8%BE%BE%E8%B0%B1\pathway%E6%95%B0%E6%8D%AE%20Control_1-VS-Osteogenic_Medium_Treat_1.htm#gene112) | 2 (0.37%) | 0.6683508 | 1.000000000 | ko05219 |
| 113 | [Metabolic pathways](file:///E:\01%20%E5%8D%9A%E5%A3%AB%E8%AE%BA%E6%96%87%E6%92%B0%E5%86%99%202016-2-1\1%20%E7%AC%AC%E4%B8%80%E9%83%A8%E5%88%86%20%EF%BC%88%E4%B8%89%EF%BC%89BMSCs%E4%B8%8E%E7%89%99%E8%83%9A%E7%BB%86%E8%83%9E%E4%BD%93%E5%A4%96%E9%97%B4%E6%8E%A5%E5%85%B1%E5%9F%B9%E5%85%BB%E6%A8%A1%E5%9E%8B%E4%B8%AD%E7%9A%84%E8%BD%AC%E5%BD%95%E7%BB%84%E8%A1%A8%E8%BE%BE%E8%B0%B1\pathway%E6%95%B0%E6%8D%AE%20Control_1-VS-Osteogenic_Medium_Treat_1.htm#gene113) | 53 (9.72%) | 0.6690058 | 1.000000000 | ko01100 |
| 114 | [Toll-like receptor signaling pathway](file:///E:\01%20%E5%8D%9A%E5%A3%AB%E8%AE%BA%E6%96%87%E6%92%B0%E5%86%99%202016-2-1\1%20%E7%AC%AC%E4%B8%80%E9%83%A8%E5%88%86%20%EF%BC%88%E4%B8%89%EF%BC%89BMSCs%E4%B8%8E%E7%89%99%E8%83%9A%E7%BB%86%E8%83%9E%E4%BD%93%E5%A4%96%E9%97%B4%E6%8E%A5%E5%85%B1%E5%9F%B9%E5%85%BB%E6%A8%A1%E5%9E%8B%E4%B8%AD%E7%9A%84%E8%BD%AC%E5%BD%95%E7%BB%84%E8%A1%A8%E8%BE%BE%E8%B0%B1\pathway%E6%95%B0%E6%8D%AE%20Control_1-VS-Osteogenic_Medium_Treat_1.htm#gene114) | 4 (0.73%) | 0.6766804 | 1.000000000 | ko04620 |
| 115 | [Sphingolipid metabolism](file:///E:\01%20%E5%8D%9A%E5%A3%AB%E8%AE%BA%E6%96%87%E6%92%B0%E5%86%99%202016-2-1\1%20%E7%AC%AC%E4%B8%80%E9%83%A8%E5%88%86%20%EF%BC%88%E4%B8%89%EF%BC%89BMSCs%E4%B8%8E%E7%89%99%E8%83%9A%E7%BB%86%E8%83%9E%E4%BD%93%E5%A4%96%E9%97%B4%E6%8E%A5%E5%85%B1%E5%9F%B9%E5%85%BB%E6%A8%A1%E5%9E%8B%E4%B8%AD%E7%9A%84%E8%BD%AC%E5%BD%95%E7%BB%84%E8%A1%A8%E8%BE%BE%E8%B0%B1\pathway%E6%95%B0%E6%8D%AE%20Control_1-VS-Osteogenic_Medium_Treat_1.htm#gene115) | 2 (0.37%) | 0.6776554 | 1.000000000 | ko00600 |
| 116 | [NF-kappa B signaling pathway](file:///E:\01%20%E5%8D%9A%E5%A3%AB%E8%AE%BA%E6%96%87%E6%92%B0%E5%86%99%202016-2-1\1%20%E7%AC%AC%E4%B8%80%E9%83%A8%E5%88%86%20%EF%BC%88%E4%B8%89%EF%BC%89BMSCs%E4%B8%8E%E7%89%99%E8%83%9A%E7%BB%86%E8%83%9E%E4%BD%93%E5%A4%96%E9%97%B4%E6%8E%A5%E5%85%B1%E5%9F%B9%E5%85%BB%E6%A8%A1%E5%9E%8B%E4%B8%AD%E7%9A%84%E8%BD%AC%E5%BD%95%E7%BB%84%E8%A1%A8%E8%BE%BE%E8%B0%B1\pathway%E6%95%B0%E6%8D%AE%20Control_1-VS-Osteogenic_Medium_Treat_1.htm#gene116) | 5 (0.92%) | 0.683591 | 1.000000000 | ko04064 |
| 117 | [Ether lipid metabolism](file:///E:\01%20%E5%8D%9A%E5%A3%AB%E8%AE%BA%E6%96%87%E6%92%B0%E5%86%99%202016-2-1\1%20%E7%AC%AC%E4%B8%80%E9%83%A8%E5%88%86%20%EF%BC%88%E4%B8%89%EF%BC%89BMSCs%E4%B8%8E%E7%89%99%E8%83%9A%E7%BB%86%E8%83%9E%E4%BD%93%E5%A4%96%E9%97%B4%E6%8E%A5%E5%85%B1%E5%9F%B9%E5%85%BB%E6%A8%A1%E5%9E%8B%E4%B8%AD%E7%9A%84%E8%BD%AC%E5%BD%95%E7%BB%84%E8%A1%A8%E8%BE%BE%E8%B0%B1\pathway%E6%95%B0%E6%8D%AE%20Control_1-VS-Osteogenic_Medium_Treat_1.htm#gene117) | 2 (0.37%) | 0.6867462 | 1.000000000 | ko00565 |
| 118 | [Hematopoietic cell lineage](file:///E:\01%20%E5%8D%9A%E5%A3%AB%E8%AE%BA%E6%96%87%E6%92%B0%E5%86%99%202016-2-1\1%20%E7%AC%AC%E4%B8%80%E9%83%A8%E5%88%86%20%EF%BC%88%E4%B8%89%EF%BC%89BMSCs%E4%B8%8E%E7%89%99%E8%83%9A%E7%BB%86%E8%83%9E%E4%BD%93%E5%A4%96%E9%97%B4%E6%8E%A5%E5%85%B1%E5%9F%B9%E5%85%BB%E6%A8%A1%E5%9E%8B%E4%B8%AD%E7%9A%84%E8%BD%AC%E5%BD%95%E7%BB%84%E8%A1%A8%E8%BE%BE%E8%B0%B1\pathway%E6%95%B0%E6%8D%AE%20Control_1-VS-Osteogenic_Medium_Treat_1.htm#gene118) | 4 (0.73%) | 0.6897421 | 1.000000000 | ko04640 |
| 119 | [MAPK signaling pathway - fly](file:///E:\01%20%E5%8D%9A%E5%A3%AB%E8%AE%BA%E6%96%87%E6%92%B0%E5%86%99%202016-2-1\1%20%E7%AC%AC%E4%B8%80%E9%83%A8%E5%88%86%20%EF%BC%88%E4%B8%89%EF%BC%89BMSCs%E4%B8%8E%E7%89%99%E8%83%9A%E7%BB%86%E8%83%9E%E4%BD%93%E5%A4%96%E9%97%B4%E6%8E%A5%E5%85%B1%E5%9F%B9%E5%85%BB%E6%A8%A1%E5%9E%8B%E4%B8%AD%E7%9A%84%E8%BD%AC%E5%BD%95%E7%BB%84%E8%A1%A8%E8%BE%BE%E8%B0%B1\pathway%E6%95%B0%E6%8D%AE%20Control_1-VS-Osteogenic_Medium_Treat_1.htm#gene119) | 1 (0.18%) | 0.7045949 | 1.000000000 | ko04013 |
| 120 | [Tuberculosis](file:///E:\01%20%E5%8D%9A%E5%A3%AB%E8%AE%BA%E6%96%87%E6%92%B0%E5%86%99%202016-2-1\1%20%E7%AC%AC%E4%B8%80%E9%83%A8%E5%88%86%20%EF%BC%88%E4%B8%89%EF%BC%89BMSCs%E4%B8%8E%E7%89%99%E8%83%9A%E7%BB%86%E8%83%9E%E4%BD%93%E5%A4%96%E9%97%B4%E6%8E%A5%E5%85%B1%E5%9F%B9%E5%85%BB%E6%A8%A1%E5%9E%8B%E4%B8%AD%E7%9A%84%E8%BD%AC%E5%BD%95%E7%BB%84%E8%A1%A8%E8%BE%BE%E8%B0%B1\pathway%E6%95%B0%E6%8D%AE%20Control_1-VS-Osteogenic_Medium_Treat_1.htm#gene120) | 9 (1.65%) | 0.7120283 | 1.000000000 | ko05152 |
| 121 | [Fat digestion and absorption](file:///E:\01%20%E5%8D%9A%E5%A3%AB%E8%AE%BA%E6%96%87%E6%92%B0%E5%86%99%202016-2-1\1%20%E7%AC%AC%E4%B8%80%E9%83%A8%E5%88%86%20%EF%BC%88%E4%B8%89%EF%BC%89BMSCs%E4%B8%8E%E7%89%99%E8%83%9A%E7%BB%86%E8%83%9E%E4%BD%93%E5%A4%96%E9%97%B4%E6%8E%A5%E5%85%B1%E5%9F%B9%E5%85%BB%E6%A8%A1%E5%9E%8B%E4%B8%AD%E7%9A%84%E8%BD%AC%E5%BD%95%E7%BB%84%E8%A1%A8%E8%BE%BE%E8%B0%B1\pathway%E6%95%B0%E6%8D%AE%20Control_1-VS-Osteogenic_Medium_Treat_1.htm#gene121) | 2 (0.37%) | 0.7127598 | 1.000000000 | ko04975 |
| 122 | [Primary bile acid biosynthesis](file:///E:\01%20%E5%8D%9A%E5%A3%AB%E8%AE%BA%E6%96%87%E6%92%B0%E5%86%99%202016-2-1\1%20%E7%AC%AC%E4%B8%80%E9%83%A8%E5%88%86%20%EF%BC%88%E4%B8%89%EF%BC%89BMSCs%E4%B8%8E%E7%89%99%E8%83%9A%E7%BB%86%E8%83%9E%E4%BD%93%E5%A4%96%E9%97%B4%E6%8E%A5%E5%85%B1%E5%9F%B9%E5%85%BB%E6%A8%A1%E5%9E%8B%E4%B8%AD%E7%9A%84%E8%BD%AC%E5%BD%95%E7%BB%84%E8%A1%A8%E8%BE%BE%E8%B0%B1\pathway%E6%95%B0%E6%8D%AE%20Control_1-VS-Osteogenic_Medium_Treat_1.htm#gene122) | 1 (0.18%) | 0.7163748 | 1.000000000 | ko00120 |
| 123 | [Lysosome](file:///E:\01%20%E5%8D%9A%E5%A3%AB%E8%AE%BA%E6%96%87%E6%92%B0%E5%86%99%202016-2-1\1%20%E7%AC%AC%E4%B8%80%E9%83%A8%E5%88%86%20%EF%BC%88%E4%B8%89%EF%BC%89BMSCs%E4%B8%8E%E7%89%99%E8%83%9A%E7%BB%86%E8%83%9E%E4%BD%93%E5%A4%96%E9%97%B4%E6%8E%A5%E5%85%B1%E5%9F%B9%E5%85%BB%E6%A8%A1%E5%9E%8B%E4%B8%AD%E7%9A%84%E8%BD%AC%E5%BD%95%E7%BB%84%E8%A1%A8%E8%BE%BE%E8%B0%B1\pathway%E6%95%B0%E6%8D%AE%20Control_1-VS-Osteogenic_Medium_Treat_1.htm#gene123) | 6 (1.1%) | 0.7216689 | 1.000000000 | ko04142 |
| 124 | [alpha-Linolenic acid metabolism](file:///E:\01%20%E5%8D%9A%E5%A3%AB%E8%AE%BA%E6%96%87%E6%92%B0%E5%86%99%202016-2-1\1%20%E7%AC%AC%E4%B8%80%E9%83%A8%E5%88%86%20%EF%BC%88%E4%B8%89%EF%BC%89BMSCs%E4%B8%8E%E7%89%99%E8%83%9A%E7%BB%86%E8%83%9E%E4%BD%93%E5%A4%96%E9%97%B4%E6%8E%A5%E5%85%B1%E5%9F%B9%E5%85%BB%E6%A8%A1%E5%9E%8B%E4%B8%AD%E7%9A%84%E8%BD%AC%E5%BD%95%E7%BB%84%E8%A1%A8%E8%BE%BE%E8%B0%B1\pathway%E6%95%B0%E6%8D%AE%20Control_1-VS-Osteogenic_Medium_Treat_1.htm#gene124) | 1 (0.18%) | 0.7276858 | 1.000000000 | ko00592 |
| 125 | [RNA polymerase](file:///E:\01%20%E5%8D%9A%E5%A3%AB%E8%AE%BA%E6%96%87%E6%92%B0%E5%86%99%202016-2-1\1%20%E7%AC%AC%E4%B8%80%E9%83%A8%E5%88%86%20%EF%BC%88%E4%B8%89%EF%BC%89BMSCs%E4%B8%8E%E7%89%99%E8%83%9A%E7%BB%86%E8%83%9E%E4%BD%93%E5%A4%96%E9%97%B4%E6%8E%A5%E5%85%B1%E5%9F%B9%E5%85%BB%E6%A8%A1%E5%9E%8B%E4%B8%AD%E7%9A%84%E8%BD%AC%E5%BD%95%E7%BB%84%E8%A1%A8%E8%BE%BE%E8%B0%B1\pathway%E6%95%B0%E6%8D%AE%20Control_1-VS-Osteogenic_Medium_Treat_1.htm#gene125) | 1 (0.18%) | 0.7276858 | 1.000000000 | ko03020 |
| 126 | [PPAR signaling pathway](file:///E:\01%20%E5%8D%9A%E5%A3%AB%E8%AE%BA%E6%96%87%E6%92%B0%E5%86%99%202016-2-1\1%20%E7%AC%AC%E4%B8%80%E9%83%A8%E5%88%86%20%EF%BC%88%E4%B8%89%EF%BC%89BMSCs%E4%B8%8E%E7%89%99%E8%83%9A%E7%BB%86%E8%83%9E%E4%BD%93%E5%A4%96%E9%97%B4%E6%8E%A5%E5%85%B1%E5%9F%B9%E5%85%BB%E6%A8%A1%E5%9E%8B%E4%B8%AD%E7%9A%84%E8%BD%AC%E5%BD%95%E7%BB%84%E8%A1%A8%E8%BE%BE%E8%B0%B1\pathway%E6%95%B0%E6%8D%AE%20Control_1-VS-Osteogenic_Medium_Treat_1.htm#gene126) | 4 (0.73%) | 0.7324845 | 1.000000000 | ko03320 |
| 127 | [Viral myocarditis](file:///E:\01%20%E5%8D%9A%E5%A3%AB%E8%AE%BA%E6%96%87%E6%92%B0%E5%86%99%202016-2-1\1%20%E7%AC%AC%E4%B8%80%E9%83%A8%E5%88%86%20%EF%BC%88%E4%B8%89%EF%BC%89BMSCs%E4%B8%8E%E7%89%99%E8%83%9A%E7%BB%86%E8%83%9E%E4%BD%93%E5%A4%96%E9%97%B4%E6%8E%A5%E5%85%B1%E5%9F%B9%E5%85%BB%E6%A8%A1%E5%9E%8B%E4%B8%AD%E7%9A%84%E8%BD%AC%E5%BD%95%E7%BB%84%E8%A1%A8%E8%BE%BE%E8%B0%B1\pathway%E6%95%B0%E6%8D%AE%20Control_1-VS-Osteogenic_Medium_Treat_1.htm#gene127) | 7 (1.28%) | 0.7399051 | 1.000000000 | ko05416 |
| 128 | [Salivary secretion](file:///E:\01%20%E5%8D%9A%E5%A3%AB%E8%AE%BA%E6%96%87%E6%92%B0%E5%86%99%202016-2-1\1%20%E7%AC%AC%E4%B8%80%E9%83%A8%E5%88%86%20%EF%BC%88%E4%B8%89%EF%BC%89BMSCs%E4%B8%8E%E7%89%99%E8%83%9A%E7%BB%86%E8%83%9E%E4%BD%93%E5%A4%96%E9%97%B4%E6%8E%A5%E5%85%B1%E5%9F%B9%E5%85%BB%E6%A8%A1%E5%9E%8B%E4%B8%AD%E7%9A%84%E8%BD%AC%E5%BD%95%E7%BB%84%E8%A1%A8%E8%BE%BE%E8%B0%B1\pathway%E6%95%B0%E6%8D%AE%20Control_1-VS-Osteogenic_Medium_Treat_1.htm#gene128) | 4 (0.73%) | 0.7438517 | 1.000000000 | ko04970 |
| 129 | [Carbohydrate digestion and absorption](file:///E:\01%20%E5%8D%9A%E5%A3%AB%E8%AE%BA%E6%96%87%E6%92%B0%E5%86%99%202016-2-1\1%20%E7%AC%AC%E4%B8%80%E9%83%A8%E5%88%86%20%EF%BC%88%E4%B8%89%EF%BC%89BMSCs%E4%B8%8E%E7%89%99%E8%83%9A%E7%BB%86%E8%83%9E%E4%BD%93%E5%A4%96%E9%97%B4%E6%8E%A5%E5%85%B1%E5%9F%B9%E5%85%BB%E6%A8%A1%E5%9E%8B%E4%B8%AD%E7%9A%84%E8%BD%AC%E5%BD%95%E7%BB%84%E8%A1%A8%E8%BE%BE%E8%B0%B1\pathway%E6%95%B0%E6%8D%AE%20Control_1-VS-Osteogenic_Medium_Treat_1.htm#gene129) | 2 (0.37%) | 0.7446052 | 1.000000000 | ko04973 |
| 130 | [TGF-beta signaling pathway](file:///E:\01%20%E5%8D%9A%E5%A3%AB%E8%AE%BA%E6%96%87%E6%92%B0%E5%86%99%202016-2-1\1%20%E7%AC%AC%E4%B8%80%E9%83%A8%E5%88%86%20%EF%BC%88%E4%B8%89%EF%BC%89BMSCs%E4%B8%8E%E7%89%99%E8%83%9A%E7%BB%86%E8%83%9E%E4%BD%93%E5%A4%96%E9%97%B4%E6%8E%A5%E5%85%B1%E5%9F%B9%E5%85%BB%E6%A8%A1%E5%9E%8B%E4%B8%AD%E7%9A%84%E8%BD%AC%E5%BD%95%E7%BB%84%E8%A1%A8%E8%BE%BE%E8%B0%B1\pathway%E6%95%B0%E6%8D%AE%20Control_1-VS-Osteogenic_Medium_Treat_1.htm#gene130) | 4 (0.73%) | 0.7602084 | 1.000000000 | ko04350 |
| 131 | [Drug metabolism - other enzymes](file:///E:\01%20%E5%8D%9A%E5%A3%AB%E8%AE%BA%E6%96%87%E6%92%B0%E5%86%99%202016-2-1\1%20%E7%AC%AC%E4%B8%80%E9%83%A8%E5%88%86%20%EF%BC%88%E4%B8%89%EF%BC%89BMSCs%E4%B8%8E%E7%89%99%E8%83%9A%E7%BB%86%E8%83%9E%E4%BD%93%E5%A4%96%E9%97%B4%E6%8E%A5%E5%85%B1%E5%9F%B9%E5%85%BB%E6%A8%A1%E5%9E%8B%E4%B8%AD%E7%9A%84%E8%BD%AC%E5%BD%95%E7%BB%84%E8%A1%A8%E8%BE%BE%E8%B0%B1\pathway%E6%95%B0%E6%8D%AE%20Control_1-VS-Osteogenic_Medium_Treat_1.htm#gene131) | 2 (0.37%) | 0.7664618 | 1.000000000 | ko00983 |
| 132 | [Cholinergic synapse](file:///E:\01%20%E5%8D%9A%E5%A3%AB%E8%AE%BA%E6%96%87%E6%92%B0%E5%86%99%202016-2-1\1%20%E7%AC%AC%E4%B8%80%E9%83%A8%E5%88%86%20%EF%BC%88%E4%B8%89%EF%BC%89BMSCs%E4%B8%8E%E7%89%99%E8%83%9A%E7%BB%86%E8%83%9E%E4%BD%93%E5%A4%96%E9%97%B4%E6%8E%A5%E5%85%B1%E5%9F%B9%E5%85%BB%E6%A8%A1%E5%9E%8B%E4%B8%AD%E7%9A%84%E8%BD%AC%E5%BD%95%E7%BB%84%E8%A1%A8%E8%BE%BE%E8%B0%B1\pathway%E6%95%B0%E6%8D%AE%20Control_1-VS-Osteogenic_Medium_Treat_1.htm#gene132) | 4 (0.73%) | 0.7706561 | 1.000000000 | ko04725 |
| 133 | [Glutathione metabolism](file:///E:\01%20%E5%8D%9A%E5%A3%AB%E8%AE%BA%E6%96%87%E6%92%B0%E5%86%99%202016-2-1\1%20%E7%AC%AC%E4%B8%80%E9%83%A8%E5%88%86%20%EF%BC%88%E4%B8%89%EF%BC%89BMSCs%E4%B8%8E%E7%89%99%E8%83%9A%E7%BB%86%E8%83%9E%E4%BD%93%E5%A4%96%E9%97%B4%E6%8E%A5%E5%85%B1%E5%9F%B9%E5%85%BB%E6%A8%A1%E5%9E%8B%E4%B8%AD%E7%9A%84%E8%BD%AC%E5%BD%95%E7%BB%84%E8%A1%A8%E8%BE%BE%E8%B0%B1\pathway%E6%95%B0%E6%8D%AE%20Control_1-VS-Osteogenic_Medium_Treat_1.htm#gene133) | 2 (0.37%) | 0.7733778 | 1.000000000 | ko00480 |
| 134 | [Small cell lung cancer](file:///E:\01%20%E5%8D%9A%E5%A3%AB%E8%AE%BA%E6%96%87%E6%92%B0%E5%86%99%202016-2-1\1%20%E7%AC%AC%E4%B8%80%E9%83%A8%E5%88%86%20%EF%BC%88%E4%B8%89%EF%BC%89BMSCs%E4%B8%8E%E7%89%99%E8%83%9A%E7%BB%86%E8%83%9E%E4%BD%93%E5%A4%96%E9%97%B4%E6%8E%A5%E5%85%B1%E5%9F%B9%E5%85%BB%E6%A8%A1%E5%9E%8B%E4%B8%AD%E7%9A%84%E8%BD%AC%E5%BD%95%E7%BB%84%E8%A1%A8%E8%BE%BE%E8%B0%B1\pathway%E6%95%B0%E6%8D%AE%20Control_1-VS-Osteogenic_Medium_Treat_1.htm#gene134) | 3 (0.55%) | 0.7773564 | 1.000000000 | ko05222 |
| 135 | [Pentose and glucuronate interconversions](file:///E:\01%20%E5%8D%9A%E5%A3%AB%E8%AE%BA%E6%96%87%E6%92%B0%E5%86%99%202016-2-1\1%20%E7%AC%AC%E4%B8%80%E9%83%A8%E5%88%86%20%EF%BC%88%E4%B8%89%EF%BC%89BMSCs%E4%B8%8E%E7%89%99%E8%83%9A%E7%BB%86%E8%83%9E%E4%BD%93%E5%A4%96%E9%97%B4%E6%8E%A5%E5%85%B1%E5%9F%B9%E5%85%BB%E6%A8%A1%E5%9E%8B%E4%B8%AD%E7%9A%84%E8%BD%AC%E5%BD%95%E7%BB%84%E8%A1%A8%E8%BE%BE%E8%B0%B1\pathway%E6%95%B0%E6%8D%AE%20Control_1-VS-Osteogenic_Medium_Treat_1.htm#gene135) | 1 (0.18%) | 0.7778337 | 1.000000000 | ko00040 |
| 136 | [Hepatitis C](file:///E:\01%20%E5%8D%9A%E5%A3%AB%E8%AE%BA%E6%96%87%E6%92%B0%E5%86%99%202016-2-1\1%20%E7%AC%AC%E4%B8%80%E9%83%A8%E5%88%86%20%EF%BC%88%E4%B8%89%EF%BC%89BMSCs%E4%B8%8E%E7%89%99%E8%83%9A%E7%BB%86%E8%83%9E%E4%BD%93%E5%A4%96%E9%97%B4%E6%8E%A5%E5%85%B1%E5%9F%B9%E5%85%BB%E6%A8%A1%E5%9E%8B%E4%B8%AD%E7%9A%84%E8%BD%AC%E5%BD%95%E7%BB%84%E8%A1%A8%E8%BE%BE%E8%B0%B1\pathway%E6%95%B0%E6%8D%AE%20Control_1-VS-Osteogenic_Medium_Treat_1.htm#gene136) | 5 (0.92%) | 0.7820462 | 1.000000000 | ko05160 |
| 137 | [Serotonergic synapse](file:///E:\01%20%E5%8D%9A%E5%A3%AB%E8%AE%BA%E6%96%87%E6%92%B0%E5%86%99%202016-2-1\1%20%E7%AC%AC%E4%B8%80%E9%83%A8%E5%88%86%20%EF%BC%88%E4%B8%89%EF%BC%89BMSCs%E4%B8%8E%E7%89%99%E8%83%9A%E7%BB%86%E8%83%9E%E4%BD%93%E5%A4%96%E9%97%B4%E6%8E%A5%E5%85%B1%E5%9F%B9%E5%85%BB%E6%A8%A1%E5%9E%8B%E4%B8%AD%E7%9A%84%E8%BD%AC%E5%BD%95%E7%BB%84%E8%A1%A8%E8%BE%BE%E8%B0%B1\pathway%E6%95%B0%E6%8D%AE%20Control_1-VS-Osteogenic_Medium_Treat_1.htm#gene137) | 5 (0.92%) | 0.7864986 | 1.000000000 | ko04726 |
| 138 | [Chronic myeloid leukemia](file:///E:\01%20%E5%8D%9A%E5%A3%AB%E8%AE%BA%E6%96%87%E6%92%B0%E5%86%99%202016-2-1\1%20%E7%AC%AC%E4%B8%80%E9%83%A8%E5%88%86%20%EF%BC%88%E4%B8%89%EF%BC%89BMSCs%E4%B8%8E%E7%89%99%E8%83%9A%E7%BB%86%E8%83%9E%E4%BD%93%E5%A4%96%E9%97%B4%E6%8E%A5%E5%85%B1%E5%9F%B9%E5%85%BB%E6%A8%A1%E5%9E%8B%E4%B8%AD%E7%9A%84%E8%BD%AC%E5%BD%95%E7%BB%84%E8%A1%A8%E8%BE%BE%E8%B0%B1\pathway%E6%95%B0%E6%8D%AE%20Control_1-VS-Osteogenic_Medium_Treat_1.htm#gene138) | 3 (0.55%) | 0.7938212 | 1.000000000 | ko05220 |
| 139 | [Fc gamma R-mediated phagocytosis](file:///E:\01%20%E5%8D%9A%E5%A3%AB%E8%AE%BA%E6%96%87%E6%92%B0%E5%86%99%202016-2-1\1%20%E7%AC%AC%E4%B8%80%E9%83%A8%E5%88%86%20%EF%BC%88%E4%B8%89%EF%BC%89BMSCs%E4%B8%8E%E7%89%99%E8%83%9A%E7%BB%86%E8%83%9E%E4%BD%93%E5%A4%96%E9%97%B4%E6%8E%A5%E5%85%B1%E5%9F%B9%E5%85%BB%E6%A8%A1%E5%9E%8B%E4%B8%AD%E7%9A%84%E8%BD%AC%E5%BD%95%E7%BB%84%E8%A1%A8%E8%BE%BE%E8%B0%B1\pathway%E6%95%B0%E6%8D%AE%20Control_1-VS-Osteogenic_Medium_Treat_1.htm#gene139) | 5 (0.92%) | 0.7951914 | 1.000000000 | ko04666 |
| 140 | [Cardiac muscle contraction](file:///E:\01%20%E5%8D%9A%E5%A3%AB%E8%AE%BA%E6%96%87%E6%92%B0%E5%86%99%202016-2-1\1%20%E7%AC%AC%E4%B8%80%E9%83%A8%E5%88%86%20%EF%BC%88%E4%B8%89%EF%BC%89BMSCs%E4%B8%8E%E7%89%99%E8%83%9A%E7%BB%86%E8%83%9E%E4%BD%93%E5%A4%96%E9%97%B4%E6%8E%A5%E5%85%B1%E5%9F%B9%E5%85%BB%E6%A8%A1%E5%9E%8B%E4%B8%AD%E7%9A%84%E8%BD%AC%E5%BD%95%E7%BB%84%E8%A1%A8%E8%BE%BE%E8%B0%B1\pathway%E6%95%B0%E6%8D%AE%20Control_1-VS-Osteogenic_Medium_Treat_1.htm#gene140) | 5 (0.92%) | 0.807708 | 1.000000000 | ko04260 |
| 141 | [Apoptosis](file:///E:\01%20%E5%8D%9A%E5%A3%AB%E8%AE%BA%E6%96%87%E6%92%B0%E5%86%99%202016-2-1\1%20%E7%AC%AC%E4%B8%80%E9%83%A8%E5%88%86%20%EF%BC%88%E4%B8%89%EF%BC%89BMSCs%E4%B8%8E%E7%89%99%E8%83%9A%E7%BB%86%E8%83%9E%E4%BD%93%E5%A4%96%E9%97%B4%E6%8E%A5%E5%85%B1%E5%9F%B9%E5%85%BB%E6%A8%A1%E5%9E%8B%E4%B8%AD%E7%9A%84%E8%BD%AC%E5%BD%95%E7%BB%84%E8%A1%A8%E8%BE%BE%E8%B0%B1\pathway%E6%95%B0%E6%8D%AE%20Control_1-VS-Osteogenic_Medium_Treat_1.htm#gene141) | 3 (0.55%) | 0.8092595 | 1.000000000 | ko04210 |
| 142 | [Thyroid cancer](file:///E:\01%20%E5%8D%9A%E5%A3%AB%E8%AE%BA%E6%96%87%E6%92%B0%E5%86%99%202016-2-1\1%20%E7%AC%AC%E4%B8%80%E9%83%A8%E5%88%86%20%EF%BC%88%E4%B8%89%EF%BC%89BMSCs%E4%B8%8E%E7%89%99%E8%83%9A%E7%BB%86%E8%83%9E%E4%BD%93%E5%A4%96%E9%97%B4%E6%8E%A5%E5%85%B1%E5%9F%B9%E5%85%BB%E6%A8%A1%E5%9E%8B%E4%B8%AD%E7%9A%84%E8%BD%AC%E5%BD%95%E7%BB%84%E8%A1%A8%E8%BE%BE%E8%B0%B1\pathway%E6%95%B0%E6%8D%AE%20Control_1-VS-Osteogenic_Medium_Treat_1.htm#gene142) | 1 (0.18%) | 0.8112267 | 1.000000000 | ko05216 |
| 143 | [Endocrine and other factor-regulated calcium reabsorption](file:///E:\01%20%E5%8D%9A%E5%A3%AB%E8%AE%BA%E6%96%87%E6%92%B0%E5%86%99%202016-2-1\1%20%E7%AC%AC%E4%B8%80%E9%83%A8%E5%88%86%20%EF%BC%88%E4%B8%89%EF%BC%89BMSCs%E4%B8%8E%E7%89%99%E8%83%9A%E7%BB%86%E8%83%9E%E4%BD%93%E5%A4%96%E9%97%B4%E6%8E%A5%E5%85%B1%E5%9F%B9%E5%85%BB%E6%A8%A1%E5%9E%8B%E4%B8%AD%E7%9A%84%E8%BD%AC%E5%BD%95%E7%BB%84%E8%A1%A8%E8%BE%BE%E8%B0%B1\pathway%E6%95%B0%E6%8D%AE%20Control_1-VS-Osteogenic_Medium_Treat_1.htm#gene143) | 2 (0.37%) | 0.8169538 | 1.000000000 | ko04961 |
| 144 | [Huntington's disease](file:///E:\01%20%E5%8D%9A%E5%A3%AB%E8%AE%BA%E6%96%87%E6%92%B0%E5%86%99%202016-2-1\1%20%E7%AC%AC%E4%B8%80%E9%83%A8%E5%88%86%20%EF%BC%88%E4%B8%89%EF%BC%89BMSCs%E4%B8%8E%E7%89%99%E8%83%9A%E7%BB%86%E8%83%9E%E4%BD%93%E5%A4%96%E9%97%B4%E6%8E%A5%E5%85%B1%E5%9F%B9%E5%85%BB%E6%A8%A1%E5%9E%8B%E4%B8%AD%E7%9A%84%E8%BD%AC%E5%BD%95%E7%BB%84%E8%A1%A8%E8%BE%BE%E8%B0%B1\pathway%E6%95%B0%E6%8D%AE%20Control_1-VS-Osteogenic_Medium_Treat_1.htm#gene144) | 6 (1.1%) | 0.8232993 | 1.000000000 | ko05016 |
| 145 | [Cysteine and methionine metabolism](file:///E:\01%20%E5%8D%9A%E5%A3%AB%E8%AE%BA%E6%96%87%E6%92%B0%E5%86%99%202016-2-1\1%20%E7%AC%AC%E4%B8%80%E9%83%A8%E5%88%86%20%EF%BC%88%E4%B8%89%EF%BC%89BMSCs%E4%B8%8E%E7%89%99%E8%83%9A%E7%BB%86%E8%83%9E%E4%BD%93%E5%A4%96%E9%97%B4%E6%8E%A5%E5%85%B1%E5%9F%B9%E5%85%BB%E6%A8%A1%E5%9E%8B%E4%B8%AD%E7%9A%84%E8%BD%AC%E5%BD%95%E7%BB%84%E8%A1%A8%E8%BE%BE%E8%B0%B1\pathway%E6%95%B0%E6%8D%AE%20Control_1-VS-Osteogenic_Medium_Treat_1.htm#gene145) | 1 (0.18%) | 0.8259942 | 1.000000000 | ko00270 |
| 146 | [HTLV-I infection](file:///E:\01%20%E5%8D%9A%E5%A3%AB%E8%AE%BA%E6%96%87%E6%92%B0%E5%86%99%202016-2-1\1%20%E7%AC%AC%E4%B8%80%E9%83%A8%E5%88%86%20%EF%BC%88%E4%B8%89%EF%BC%89BMSCs%E4%B8%8E%E7%89%99%E8%83%9A%E7%BB%86%E8%83%9E%E4%BD%93%E5%A4%96%E9%97%B4%E6%8E%A5%E5%85%B1%E5%9F%B9%E5%85%BB%E6%A8%A1%E5%9E%8B%E4%B8%AD%E7%9A%84%E8%BD%AC%E5%BD%95%E7%BB%84%E8%A1%A8%E8%BE%BE%E8%B0%B1\pathway%E6%95%B0%E6%8D%AE%20Control_1-VS-Osteogenic_Medium_Treat_1.htm#gene146) | 11 (2.02%) | 0.8281743 | 1.000000000 | ko05166 |
| 147 | [Adipocytokine signaling pathway](file:///E:\01%20%E5%8D%9A%E5%A3%AB%E8%AE%BA%E6%96%87%E6%92%B0%E5%86%99%202016-2-1\1%20%E7%AC%AC%E4%B8%80%E9%83%A8%E5%88%86%20%EF%BC%88%E4%B8%89%EF%BC%89BMSCs%E4%B8%8E%E7%89%99%E8%83%9A%E7%BB%86%E8%83%9E%E4%BD%93%E5%A4%96%E9%97%B4%E6%8E%A5%E5%85%B1%E5%9F%B9%E5%85%BB%E6%A8%A1%E5%9E%8B%E4%B8%AD%E7%9A%84%E8%BD%AC%E5%BD%95%E7%BB%84%E8%A1%A8%E8%BE%BE%E8%B0%B1\pathway%E6%95%B0%E6%8D%AE%20Control_1-VS-Osteogenic_Medium_Treat_1.htm#gene147) | 3 (0.55%) | 0.8283169 | 1.000000000 | ko04920 |
| 148 | [Pyrimidine metabolism](file:///E:\01%20%E5%8D%9A%E5%A3%AB%E8%AE%BA%E6%96%87%E6%92%B0%E5%86%99%202016-2-1\1%20%E7%AC%AC%E4%B8%80%E9%83%A8%E5%88%86%20%EF%BC%88%E4%B8%89%EF%BC%89BMSCs%E4%B8%8E%E7%89%99%E8%83%9A%E7%BB%86%E8%83%9E%E4%BD%93%E5%A4%96%E9%97%B4%E6%8E%A5%E5%85%B1%E5%9F%B9%E5%85%BB%E6%A8%A1%E5%9E%8B%E4%B8%AD%E7%9A%84%E8%BD%AC%E5%BD%95%E7%BB%84%E8%A1%A8%E8%BE%BE%E8%B0%B1\pathway%E6%95%B0%E6%8D%AE%20Control_1-VS-Osteogenic_Medium_Treat_1.htm#gene148) | 3 (0.55%) | 0.8372193 | 1.000000000 | ko00240 |
| 149 | [Inositol phosphate metabolism](file:///E:\01%20%E5%8D%9A%E5%A3%AB%E8%AE%BA%E6%96%87%E6%92%B0%E5%86%99%202016-2-1\1%20%E7%AC%AC%E4%B8%80%E9%83%A8%E5%88%86%20%EF%BC%88%E4%B8%89%EF%BC%89BMSCs%E4%B8%8E%E7%89%99%E8%83%9A%E7%BB%86%E8%83%9E%E4%BD%93%E5%A4%96%E9%97%B4%E6%8E%A5%E5%85%B1%E5%9F%B9%E5%85%BB%E6%A8%A1%E5%9E%8B%E4%B8%AD%E7%9A%84%E8%BD%AC%E5%BD%95%E7%BB%84%E8%A1%A8%E8%BE%BE%E8%B0%B1\pathway%E6%95%B0%E6%8D%AE%20Control_1-VS-Osteogenic_Medium_Treat_1.htm#gene149) | 2 (0.37%) | 0.8433303 | 1.000000000 | ko00562 |
| 150 | [Salmonella infection](file:///E:\01%20%E5%8D%9A%E5%A3%AB%E8%AE%BA%E6%96%87%E6%92%B0%E5%86%99%202016-2-1\1%20%E7%AC%AC%E4%B8%80%E9%83%A8%E5%88%86%20%EF%BC%88%E4%B8%89%EF%BC%89BMSCs%E4%B8%8E%E7%89%99%E8%83%9A%E7%BB%86%E8%83%9E%E4%BD%93%E5%A4%96%E9%97%B4%E6%8E%A5%E5%85%B1%E5%9F%B9%E5%85%BB%E6%A8%A1%E5%9E%8B%E4%B8%AD%E7%9A%84%E8%BD%AC%E5%BD%95%E7%BB%84%E8%A1%A8%E8%BE%BE%E8%B0%B1\pathway%E6%95%B0%E6%8D%AE%20Control_1-VS-Osteogenic_Medium_Treat_1.htm#gene150) | 6 (1.1%) | 0.8466735 | 1.000000000 | ko05132 |
| 151 | [Arachidonic acid metabolism](file:///E:\01%20%E5%8D%9A%E5%A3%AB%E8%AE%BA%E6%96%87%E6%92%B0%E5%86%99%202016-2-1\1%20%E7%AC%AC%E4%B8%80%E9%83%A8%E5%88%86%20%EF%BC%88%E4%B8%89%EF%BC%89BMSCs%E4%B8%8E%E7%89%99%E8%83%9A%E7%BB%86%E8%83%9E%E4%BD%93%E5%A4%96%E9%97%B4%E6%8E%A5%E5%85%B1%E5%9F%B9%E5%85%BB%E6%A8%A1%E5%9E%8B%E4%B8%AD%E7%9A%84%E8%BD%AC%E5%BD%95%E7%BB%84%E8%A1%A8%E8%BE%BE%E8%B0%B1\pathway%E6%95%B0%E6%8D%AE%20Control_1-VS-Osteogenic_Medium_Treat_1.htm#gene151) | 3 (0.55%) | 0.8498257 | 1.000000000 | ko00590 |
| 152 | [Collecting duct acid secretion](file:///E:\01%20%E5%8D%9A%E5%A3%AB%E8%AE%BA%E6%96%87%E6%92%B0%E5%86%99%202016-2-1\1%20%E7%AC%AC%E4%B8%80%E9%83%A8%E5%88%86%20%EF%BC%88%E4%B8%89%EF%BC%89BMSCs%E4%B8%8E%E7%89%99%E8%83%9A%E7%BB%86%E8%83%9E%E4%BD%93%E5%A4%96%E9%97%B4%E6%8E%A5%E5%85%B1%E5%9F%B9%E5%85%BB%E6%A8%A1%E5%9E%8B%E4%B8%AD%E7%9A%84%E8%BD%AC%E5%BD%95%E7%BB%84%E8%A1%A8%E8%BE%BE%E8%B0%B1\pathway%E6%95%B0%E6%8D%AE%20Control_1-VS-Osteogenic_Medium_Treat_1.htm#gene152) | 1 (0.18%) | 0.8521591 | 1.000000000 | ko04966 |
| 153 | [VEGF signaling pathway](file:///E:\01%20%E5%8D%9A%E5%A3%AB%E8%AE%BA%E6%96%87%E6%92%B0%E5%86%99%202016-2-1\1%20%E7%AC%AC%E4%B8%80%E9%83%A8%E5%88%86%20%EF%BC%88%E4%B8%89%EF%BC%89BMSCs%E4%B8%8E%E7%89%99%E8%83%9A%E7%BB%86%E8%83%9E%E4%BD%93%E5%A4%96%E9%97%B4%E6%8E%A5%E5%85%B1%E5%9F%B9%E5%85%BB%E6%A8%A1%E5%9E%8B%E4%B8%AD%E7%9A%84%E8%BD%AC%E5%BD%95%E7%BB%84%E8%A1%A8%E8%BE%BE%E8%B0%B1\pathway%E6%95%B0%E6%8D%AE%20Control_1-VS-Osteogenic_Medium_Treat_1.htm#gene153) | 3 (0.55%) | 0.8653079 | 1.000000000 | ko04370 |
| 154 | [Linoleic acid metabolism](file:///E:\01%20%E5%8D%9A%E5%A3%AB%E8%AE%BA%E6%96%87%E6%92%B0%E5%86%99%202016-2-1\1%20%E7%AC%AC%E4%B8%80%E9%83%A8%E5%88%86%20%EF%BC%88%E4%B8%89%EF%BC%89BMSCs%E4%B8%8E%E7%89%99%E8%83%9A%E7%BB%86%E8%83%9E%E4%BD%93%E5%A4%96%E9%97%B4%E6%8E%A5%E5%85%B1%E5%9F%B9%E5%85%BB%E6%A8%A1%E5%9E%8B%E4%B8%AD%E7%9A%84%E8%BD%AC%E5%BD%95%E7%BB%84%E8%A1%A8%E8%BE%BE%E8%B0%B1\pathway%E6%95%B0%E6%8D%AE%20Control_1-VS-Osteogenic_Medium_Treat_1.htm#gene154) | 1 (0.18%) | 0.8691711 | 1.000000000 | ko00591 |
| 155 | [African trypanosomiasis](file:///E:\01%20%E5%8D%9A%E5%A3%AB%E8%AE%BA%E6%96%87%E6%92%B0%E5%86%99%202016-2-1\1%20%E7%AC%AC%E4%B8%80%E9%83%A8%E5%88%86%20%EF%BC%88%E4%B8%89%EF%BC%89BMSCs%E4%B8%8E%E7%89%99%E8%83%9A%E7%BB%86%E8%83%9E%E4%BD%93%E5%A4%96%E9%97%B4%E6%8E%A5%E5%85%B1%E5%9F%B9%E5%85%BB%E6%A8%A1%E5%9E%8B%E4%B8%AD%E7%9A%84%E8%BD%AC%E5%BD%95%E7%BB%84%E8%A1%A8%E8%BE%BE%E8%B0%B1\pathway%E6%95%B0%E6%8D%AE%20Control_1-VS-Osteogenic_Medium_Treat_1.htm#gene155) | 1 (0.18%) | 0.8691711 | 1.000000000 | ko05143 |
| 156 | [Glutamatergic synapse](file:///E:\01%20%E5%8D%9A%E5%A3%AB%E8%AE%BA%E6%96%87%E6%92%B0%E5%86%99%202016-2-1\1%20%E7%AC%AC%E4%B8%80%E9%83%A8%E5%88%86%20%EF%BC%88%E4%B8%89%EF%BC%89BMSCs%E4%B8%8E%E7%89%99%E8%83%9A%E7%BB%86%E8%83%9E%E4%BD%93%E5%A4%96%E9%97%B4%E6%8E%A5%E5%85%B1%E5%9F%B9%E5%85%BB%E6%A8%A1%E5%9E%8B%E4%B8%AD%E7%9A%84%E8%BD%AC%E5%BD%95%E7%BB%84%E8%A1%A8%E8%BE%BE%E8%B0%B1\pathway%E6%95%B0%E6%8D%AE%20Control_1-VS-Osteogenic_Medium_Treat_1.htm#gene156) | 4 (0.73%) | 0.8697953 | 1.000000000 | ko04724 |
| 157 | [ErbB signaling pathway](file:///E:\01%20%E5%8D%9A%E5%A3%AB%E8%AE%BA%E6%96%87%E6%92%B0%E5%86%99%202016-2-1\1%20%E7%AC%AC%E4%B8%80%E9%83%A8%E5%88%86%20%EF%BC%88%E4%B8%89%EF%BC%89BMSCs%E4%B8%8E%E7%89%99%E8%83%9A%E7%BB%86%E8%83%9E%E4%BD%93%E5%A4%96%E9%97%B4%E6%8E%A5%E5%85%B1%E5%9F%B9%E5%85%BB%E6%A8%A1%E5%9E%8B%E4%B8%AD%E7%9A%84%E8%BD%AC%E5%BD%95%E7%BB%84%E8%A1%A8%E8%BE%BE%E8%B0%B1\pathway%E6%95%B0%E6%8D%AE%20Control_1-VS-Osteogenic_Medium_Treat_1.htm#gene157) | 3 (0.55%) | 0.8793691 | 1.000000000 | ko04012 |
| 158 | [Phototransduction](file:///E:\01%20%E5%8D%9A%E5%A3%AB%E8%AE%BA%E6%96%87%E6%92%B0%E5%86%99%202016-2-1\1%20%E7%AC%AC%E4%B8%80%E9%83%A8%E5%88%86%20%EF%BC%88%E4%B8%89%EF%BC%89BMSCs%E4%B8%8E%E7%89%99%E8%83%9A%E7%BB%86%E8%83%9E%E4%BD%93%E5%A4%96%E9%97%B4%E6%8E%A5%E5%85%B1%E5%9F%B9%E5%85%BB%E6%A8%A1%E5%9E%8B%E4%B8%AD%E7%9A%84%E8%BD%AC%E5%BD%95%E7%BB%84%E8%A1%A8%E8%BE%BE%E8%B0%B1\pathway%E6%95%B0%E6%8D%AE%20Control_1-VS-Osteogenic_Medium_Treat_1.htm#gene158) | 1 (0.18%) | 0.8794122 | 1.000000000 | ko04744 |
| 159 | [Phosphatidylinositol signaling system](file:///E:\01%20%E5%8D%9A%E5%A3%AB%E8%AE%BA%E6%96%87%E6%92%B0%E5%86%99%202016-2-1\1%20%E7%AC%AC%E4%B8%80%E9%83%A8%E5%88%86%20%EF%BC%88%E4%B8%89%EF%BC%89BMSCs%E4%B8%8E%E7%89%99%E8%83%9A%E7%BB%86%E8%83%9E%E4%BD%93%E5%A4%96%E9%97%B4%E6%8E%A5%E5%85%B1%E5%9F%B9%E5%85%BB%E6%A8%A1%E5%9E%8B%E4%B8%AD%E7%9A%84%E8%BD%AC%E5%BD%95%E7%BB%84%E8%A1%A8%E8%BE%BE%E8%B0%B1\pathway%E6%95%B0%E6%8D%AE%20Control_1-VS-Osteogenic_Medium_Treat_1.htm#gene159) | 3 (0.55%) | 0.8921116 | 1.000000000 | ko04070 |
| 160 | [Rheumatoid arthritis](file:///E:\01%20%E5%8D%9A%E5%A3%AB%E8%AE%BA%E6%96%87%E6%92%B0%E5%86%99%202016-2-1\1%20%E7%AC%AC%E4%B8%80%E9%83%A8%E5%88%86%20%EF%BC%88%E4%B8%89%EF%BC%89BMSCs%E4%B8%8E%E7%89%99%E8%83%9A%E7%BB%86%E8%83%9E%E4%BD%93%E5%A4%96%E9%97%B4%E6%8E%A5%E5%85%B1%E5%9F%B9%E5%85%BB%E6%A8%A1%E5%9E%8B%E4%B8%AD%E7%9A%84%E8%BD%AC%E5%BD%95%E7%BB%84%E8%A1%A8%E8%BE%BE%E8%B0%B1\pathway%E6%95%B0%E6%8D%AE%20Control_1-VS-Osteogenic_Medium_Treat_1.htm#gene160) | 2 (0.37%) | 0.8965268 | 1.000000000 | ko05323 |
| 161 | [Cell adhesion molecules (CAMs)](file:///E:\01%20%E5%8D%9A%E5%A3%AB%E8%AE%BA%E6%96%87%E6%92%B0%E5%86%99%202016-2-1\1%20%E7%AC%AC%E4%B8%80%E9%83%A8%E5%88%86%20%EF%BC%88%E4%B8%89%EF%BC%89BMSCs%E4%B8%8E%E7%89%99%E8%83%9A%E7%BB%86%E8%83%9E%E4%BD%93%E5%A4%96%E9%97%B4%E6%8E%A5%E5%85%B1%E5%9F%B9%E5%85%BB%E6%A8%A1%E5%9E%8B%E4%B8%AD%E7%9A%84%E8%BD%AC%E5%BD%95%E7%BB%84%E8%A1%A8%E8%BE%BE%E8%B0%B1\pathway%E6%95%B0%E6%8D%AE%20Control_1-VS-Osteogenic_Medium_Treat_1.htm#gene161) | 5 (0.92%) | 0.8972071 | 1.000000000 | ko04514 |
| 162 | [Toxoplasmosis](file:///E:\01%20%E5%8D%9A%E5%A3%AB%E8%AE%BA%E6%96%87%E6%92%B0%E5%86%99%202016-2-1\1%20%E7%AC%AC%E4%B8%80%E9%83%A8%E5%88%86%20%EF%BC%88%E4%B8%89%EF%BC%89BMSCs%E4%B8%8E%E7%89%99%E8%83%9A%E7%BB%86%E8%83%9E%E4%BD%93%E5%A4%96%E9%97%B4%E6%8E%A5%E5%85%B1%E5%9F%B9%E5%85%BB%E6%A8%A1%E5%9E%8B%E4%B8%AD%E7%9A%84%E8%BD%AC%E5%BD%95%E7%BB%84%E8%A1%A8%E8%BE%BE%E8%B0%B1\pathway%E6%95%B0%E6%8D%AE%20Control_1-VS-Osteogenic_Medium_Treat_1.htm#gene162) | 4 (0.73%) | 0.9012731 | 1.000000000 | ko05145 |
| 163 | [Measles](file:///E:\01%20%E5%8D%9A%E5%A3%AB%E8%AE%BA%E6%96%87%E6%92%B0%E5%86%99%202016-2-1\1%20%E7%AC%AC%E4%B8%80%E9%83%A8%E5%88%86%20%EF%BC%88%E4%B8%89%EF%BC%89BMSCs%E4%B8%8E%E7%89%99%E8%83%9A%E7%BB%86%E8%83%9E%E4%BD%93%E5%A4%96%E9%97%B4%E6%8E%A5%E5%85%B1%E5%9F%B9%E5%85%BB%E6%A8%A1%E5%9E%8B%E4%B8%AD%E7%9A%84%E8%BD%AC%E5%BD%95%E7%BB%84%E8%A1%A8%E8%BE%BE%E8%B0%B1\pathway%E6%95%B0%E6%8D%AE%20Control_1-VS-Osteogenic_Medium_Treat_1.htm#gene163) | 4 (0.73%) | 0.9012731 | 1.000000000 | ko05162 |
| 164 | [Leishmaniasis](file:///E:\01%20%E5%8D%9A%E5%A3%AB%E8%AE%BA%E6%96%87%E6%92%B0%E5%86%99%202016-2-1\1%20%E7%AC%AC%E4%B8%80%E9%83%A8%E5%88%86%20%EF%BC%88%E4%B8%89%EF%BC%89BMSCs%E4%B8%8E%E7%89%99%E8%83%9A%E7%BB%86%E8%83%9E%E4%BD%93%E5%A4%96%E9%97%B4%E6%8E%A5%E5%85%B1%E5%9F%B9%E5%85%BB%E6%A8%A1%E5%9E%8B%E4%B8%AD%E7%9A%84%E8%BD%AC%E5%BD%95%E7%BB%84%E8%A1%A8%E8%BE%BE%E8%B0%B1\pathway%E6%95%B0%E6%8D%AE%20Control_1-VS-Osteogenic_Medium_Treat_1.htm#gene164) | 2 (0.37%) | 0.9030379 | 1.000000000 | ko05140 |
| 165 | [Vascular smooth muscle contraction](file:///E:\01%20%E5%8D%9A%E5%A3%AB%E8%AE%BA%E6%96%87%E6%92%B0%E5%86%99%202016-2-1\1%20%E7%AC%AC%E4%B8%80%E9%83%A8%E5%88%86%20%EF%BC%88%E4%B8%89%EF%BC%89BMSCs%E4%B8%8E%E7%89%99%E8%83%9A%E7%BB%86%E8%83%9E%E4%BD%93%E5%A4%96%E9%97%B4%E6%8E%A5%E5%85%B1%E5%9F%B9%E5%85%BB%E6%A8%A1%E5%9E%8B%E4%B8%AD%E7%9A%84%E8%BD%AC%E5%BD%95%E7%BB%84%E8%A1%A8%E8%BE%BE%E8%B0%B1\pathway%E6%95%B0%E6%8D%AE%20Control_1-VS-Osteogenic_Medium_Treat_1.htm#gene165) | 7 (1.28%) | 0.9054058 | 1.000000000 | ko04270 |
| 166 | [N-Glycan biosynthesis](file:///E:\01%20%E5%8D%9A%E5%A3%AB%E8%AE%BA%E6%96%87%E6%92%B0%E5%86%99%202016-2-1\1%20%E7%AC%AC%E4%B8%80%E9%83%A8%E5%88%86%20%EF%BC%88%E4%B8%89%EF%BC%89BMSCs%E4%B8%8E%E7%89%99%E8%83%9A%E7%BB%86%E8%83%9E%E4%BD%93%E5%A4%96%E9%97%B4%E6%8E%A5%E5%85%B1%E5%9F%B9%E5%85%BB%E6%A8%A1%E5%9E%8B%E4%B8%AD%E7%9A%84%E8%BD%AC%E5%BD%95%E7%BB%84%E8%A1%A8%E8%BE%BE%E8%B0%B1\pathway%E6%95%B0%E6%8D%AE%20Control_1-VS-Osteogenic_Medium_Treat_1.htm#gene166) | 1 (0.18%) | 0.9093517 | 1.000000000 | ko00510 |
| 167 | [Dopaminergic synapse](file:///E:\01%20%E5%8D%9A%E5%A3%AB%E8%AE%BA%E6%96%87%E6%92%B0%E5%86%99%202016-2-1\1%20%E7%AC%AC%E4%B8%80%E9%83%A8%E5%88%86%20%EF%BC%88%E4%B8%89%EF%BC%89BMSCs%E4%B8%8E%E7%89%99%E8%83%9A%E7%BB%86%E8%83%9E%E4%BD%93%E5%A4%96%E9%97%B4%E6%8E%A5%E5%85%B1%E5%9F%B9%E5%85%BB%E6%A8%A1%E5%9E%8B%E4%B8%AD%E7%9A%84%E8%BD%AC%E5%BD%95%E7%BB%84%E8%A1%A8%E8%BE%BE%E8%B0%B1\pathway%E6%95%B0%E6%8D%AE%20Control_1-VS-Osteogenic_Medium_Treat_1.htm#gene167) | 4 (0.73%) | 0.9132183 | 1.000000000 | ko04728 |
| 168 | [Other types of O-glycan biosynthesis](file:///E:\01%20%E5%8D%9A%E5%A3%AB%E8%AE%BA%E6%96%87%E6%92%B0%E5%86%99%202016-2-1\1%20%E7%AC%AC%E4%B8%80%E9%83%A8%E5%88%86%20%EF%BC%88%E4%B8%89%EF%BC%89BMSCs%E4%B8%8E%E7%89%99%E8%83%9A%E7%BB%86%E8%83%9E%E4%BD%93%E5%A4%96%E9%97%B4%E6%8E%A5%E5%85%B1%E5%9F%B9%E5%85%BB%E6%A8%A1%E5%9E%8B%E4%B8%AD%E7%9A%84%E8%BD%AC%E5%BD%95%E7%BB%84%E8%A1%A8%E8%BE%BE%E8%B0%B1\pathway%E6%95%B0%E6%8D%AE%20Control_1-VS-Osteogenic_Medium_Treat_1.htm#gene168) | 1 (0.18%) | 0.9164521 | 1.000000000 | ko00514 |
| 169 | [Fatty acid metabolism](file:///E:\01%20%E5%8D%9A%E5%A3%AB%E8%AE%BA%E6%96%87%E6%92%B0%E5%86%99%202016-2-1\1%20%E7%AC%AC%E4%B8%80%E9%83%A8%E5%88%86%20%EF%BC%88%E4%B8%89%EF%BC%89BMSCs%E4%B8%8E%E7%89%99%E8%83%9A%E7%BB%86%E8%83%9E%E4%BD%93%E5%A4%96%E9%97%B4%E6%8E%A5%E5%85%B1%E5%9F%B9%E5%85%BB%E6%A8%A1%E5%9E%8B%E4%B8%AD%E7%9A%84%E8%BD%AC%E5%BD%95%E7%BB%84%E8%A1%A8%E8%BE%BE%E8%B0%B1\pathway%E6%95%B0%E6%8D%AE%20Control_1-VS-Osteogenic_Medium_Treat_1.htm#gene169) | 1 (0.18%) | 0.9197914 | 1.000000000 | ko00071 |
| 170 | [NOD-like receptor signaling pathway](file:///E:\01%20%E5%8D%9A%E5%A3%AB%E8%AE%BA%E6%96%87%E6%92%B0%E5%86%99%202016-2-1\1%20%E7%AC%AC%E4%B8%80%E9%83%A8%E5%88%86%20%EF%BC%88%E4%B8%89%EF%BC%89BMSCs%E4%B8%8E%E7%89%99%E8%83%9A%E7%BB%86%E8%83%9E%E4%BD%93%E5%A4%96%E9%97%B4%E6%8E%A5%E5%85%B1%E5%9F%B9%E5%85%BB%E6%A8%A1%E5%9E%8B%E4%B8%AD%E7%9A%84%E8%BD%AC%E5%BD%95%E7%BB%84%E8%A1%A8%E8%BE%BE%E8%B0%B1\pathway%E6%95%B0%E6%8D%AE%20Control_1-VS-Osteogenic_Medium_Treat_1.htm#gene170) | 2 (0.37%) | 0.9229341 | 1.000000000 | ko04621 |
| 171 | [Synaptic vesicle cycle](file:///E:\01%20%E5%8D%9A%E5%A3%AB%E8%AE%BA%E6%96%87%E6%92%B0%E5%86%99%202016-2-1\1%20%E7%AC%AC%E4%B8%80%E9%83%A8%E5%88%86%20%EF%BC%88%E4%B8%89%EF%BC%89BMSCs%E4%B8%8E%E7%89%99%E8%83%9A%E7%BB%86%E8%83%9E%E4%BD%93%E5%A4%96%E9%97%B4%E6%8E%A5%E5%85%B1%E5%9F%B9%E5%85%BB%E6%A8%A1%E5%9E%8B%E4%B8%AD%E7%9A%84%E8%BD%AC%E5%BD%95%E7%BB%84%E8%A1%A8%E8%BE%BE%E8%B0%B1\pathway%E6%95%B0%E6%8D%AE%20Control_1-VS-Osteogenic_Medium_Treat_1.htm#gene171) | 2 (0.37%) | 0.9229341 | 1.000000000 | ko04721 |
| 172 | [Fructose and mannose metabolism](file:///E:\01%20%E5%8D%9A%E5%A3%AB%E8%AE%BA%E6%96%87%E6%92%B0%E5%86%99%202016-2-1\1%20%E7%AC%AC%E4%B8%80%E9%83%A8%E5%88%86%20%EF%BC%88%E4%B8%89%EF%BC%89BMSCs%E4%B8%8E%E7%89%99%E8%83%9A%E7%BB%86%E8%83%9E%E4%BD%93%E5%A4%96%E9%97%B4%E6%8E%A5%E5%85%B1%E5%9F%B9%E5%85%BB%E6%A8%A1%E5%9E%8B%E4%B8%AD%E7%9A%84%E8%BD%AC%E5%BD%95%E7%BB%84%E8%A1%A8%E8%BE%BE%E8%B0%B1\pathway%E6%95%B0%E6%8D%AE%20Control_1-VS-Osteogenic_Medium_Treat_1.htm#gene172) | 1 (0.18%) | 0.9290307 | 1.000000000 | ko00051 |
| 173 | [Amino sugar and nucleotide sugar metabolism](file:///E:\01%20%E5%8D%9A%E5%A3%AB%E8%AE%BA%E6%96%87%E6%92%B0%E5%86%99%202016-2-1\1%20%E7%AC%AC%E4%B8%80%E9%83%A8%E5%88%86%20%EF%BC%88%E4%B8%89%EF%BC%89BMSCs%E4%B8%8E%E7%89%99%E8%83%9A%E7%BB%86%E8%83%9E%E4%BD%93%E5%A4%96%E9%97%B4%E6%8E%A5%E5%85%B1%E5%9F%B9%E5%85%BB%E6%A8%A1%E5%9E%8B%E4%B8%AD%E7%9A%84%E8%BD%AC%E5%BD%95%E7%BB%84%E8%A1%A8%E8%BE%BE%E8%B0%B1\pathway%E6%95%B0%E6%8D%AE%20Control_1-VS-Osteogenic_Medium_Treat_1.htm#gene173) | 1 (0.18%) | 0.9345921 | 1.000000000 | ko00520 |
| 174 | [Taste transduction](file:///E:\01%20%E5%8D%9A%E5%A3%AB%E8%AE%BA%E6%96%87%E6%92%B0%E5%86%99%202016-2-1\1%20%E7%AC%AC%E4%B8%80%E9%83%A8%E5%88%86%20%EF%BC%88%E4%B8%89%EF%BC%89BMSCs%E4%B8%8E%E7%89%99%E8%83%9A%E7%BB%86%E8%83%9E%E4%BD%93%E5%A4%96%E9%97%B4%E6%8E%A5%E5%85%B1%E5%9F%B9%E5%85%BB%E6%A8%A1%E5%9E%8B%E4%B8%AD%E7%9A%84%E8%BD%AC%E5%BD%95%E7%BB%84%E8%A1%A8%E8%BE%BE%E8%B0%B1\pathway%E6%95%B0%E6%8D%AE%20Control_1-VS-Osteogenic_Medium_Treat_1.htm#gene174) | 1 (0.18%) | 0.9345921 | 1.000000000 | ko04742 |
| 175 | [RIG-I-like receptor signaling pathway](file:///E:\01%20%E5%8D%9A%E5%A3%AB%E8%AE%BA%E6%96%87%E6%92%B0%E5%86%99%202016-2-1\1%20%E7%AC%AC%E4%B8%80%E9%83%A8%E5%88%86%20%EF%BC%88%E4%B8%89%EF%BC%89BMSCs%E4%B8%8E%E7%89%99%E8%83%9A%E7%BB%86%E8%83%9E%E4%BD%93%E5%A4%96%E9%97%B4%E6%8E%A5%E5%85%B1%E5%9F%B9%E5%85%BB%E6%A8%A1%E5%9E%8B%E4%B8%AD%E7%9A%84%E8%BD%AC%E5%BD%95%E7%BB%84%E8%A1%A8%E8%BE%BE%E8%B0%B1\pathway%E6%95%B0%E6%8D%AE%20Control_1-VS-Osteogenic_Medium_Treat_1.htm#gene175) | 1 (0.18%) | 0.9397184 | 1.000000000 | ko04622 |
| 176 | [Amoebiasis](file:///E:\01%20%E5%8D%9A%E5%A3%AB%E8%AE%BA%E6%96%87%E6%92%B0%E5%86%99%202016-2-1\1%20%E7%AC%AC%E4%B8%80%E9%83%A8%E5%88%86%20%EF%BC%88%E4%B8%89%EF%BC%89BMSCs%E4%B8%8E%E7%89%99%E8%83%9A%E7%BB%86%E8%83%9E%E4%BD%93%E5%A4%96%E9%97%B4%E6%8E%A5%E5%85%B1%E5%9F%B9%E5%85%BB%E6%A8%A1%E5%9E%8B%E4%B8%AD%E7%9A%84%E8%BD%AC%E5%BD%95%E7%BB%84%E8%A1%A8%E8%BE%BE%E8%B0%B1\pathway%E6%95%B0%E6%8D%AE%20Control_1-VS-Osteogenic_Medium_Treat_1.htm#gene176) | 3 (0.55%) | 0.9428882 | 1.000000000 | ko05146 |
| 177 | [Endometrial cancer](file:///E:\01%20%E5%8D%9A%E5%A3%AB%E8%AE%BA%E6%96%87%E6%92%B0%E5%86%99%202016-2-1\1%20%E7%AC%AC%E4%B8%80%E9%83%A8%E5%88%86%20%EF%BC%88%E4%B8%89%EF%BC%89BMSCs%E4%B8%8E%E7%89%99%E8%83%9A%E7%BB%86%E8%83%9E%E4%BD%93%E5%A4%96%E9%97%B4%E6%8E%A5%E5%85%B1%E5%9F%B9%E5%85%BB%E6%A8%A1%E5%9E%8B%E4%B8%AD%E7%9A%84%E8%BD%AC%E5%BD%95%E7%BB%84%E8%A1%A8%E8%BE%BE%E8%B0%B1\pathway%E6%95%B0%E6%8D%AE%20Control_1-VS-Osteogenic_Medium_Treat_1.htm#gene177) | 1 (0.18%) | 0.9487991 | 1.000000000 | ko05213 |
| 178 | [Fc epsilon RI signaling pathway](file:///E:\01%20%E5%8D%9A%E5%A3%AB%E8%AE%BA%E6%96%87%E6%92%B0%E5%86%99%202016-2-1\1%20%E7%AC%AC%E4%B8%80%E9%83%A8%E5%88%86%20%EF%BC%88%E4%B8%89%EF%BC%89BMSCs%E4%B8%8E%E7%89%99%E8%83%9A%E7%BB%86%E8%83%9E%E4%BD%93%E5%A4%96%E9%97%B4%E6%8E%A5%E5%85%B1%E5%9F%B9%E5%85%BB%E6%A8%A1%E5%9E%8B%E4%B8%AD%E7%9A%84%E8%BD%AC%E5%BD%95%E7%BB%84%E8%A1%A8%E8%BE%BE%E8%B0%B1\pathway%E6%95%B0%E6%8D%AE%20Control_1-VS-Osteogenic_Medium_Treat_1.htm#gene178) | 2 (0.37%) | 0.9500981 | 1.000000000 | ko04664 |
| 179 | [Transcriptional misregulation in cancer](file:///E:\01%20%E5%8D%9A%E5%A3%AB%E8%AE%BA%E6%96%87%E6%92%B0%E5%86%99%202016-2-1\1%20%E7%AC%AC%E4%B8%80%E9%83%A8%E5%88%86%20%EF%BC%88%E4%B8%89%EF%BC%89BMSCs%E4%B8%8E%E7%89%99%E8%83%9A%E7%BB%86%E8%83%9E%E4%BD%93%E5%A4%96%E9%97%B4%E6%8E%A5%E5%85%B1%E5%9F%B9%E5%85%BB%E6%A8%A1%E5%9E%8B%E4%B8%AD%E7%9A%84%E8%BD%AC%E5%BD%95%E7%BB%84%E8%A1%A8%E8%BE%BE%E8%B0%B1\pathway%E6%95%B0%E6%8D%AE%20Control_1-VS-Osteogenic_Medium_Treat_1.htm#gene179) | 6 (1.1%) | 0.952187 | 1.000000000 | ko05202 |
| 180 | [Malaria](file:///E:\01%20%E5%8D%9A%E5%A3%AB%E8%AE%BA%E6%96%87%E6%92%B0%E5%86%99%202016-2-1\1%20%E7%AC%AC%E4%B8%80%E9%83%A8%E5%88%86%20%EF%BC%88%E4%B8%89%EF%BC%89BMSCs%E4%B8%8E%E7%89%99%E8%83%9A%E7%BB%86%E8%83%9E%E4%BD%93%E5%A4%96%E9%97%B4%E6%8E%A5%E5%85%B1%E5%9F%B9%E5%85%BB%E6%A8%A1%E5%9E%8B%E4%B8%AD%E7%9A%84%E8%BD%AC%E5%BD%95%E7%BB%84%E8%A1%A8%E8%BE%BE%E8%B0%B1\pathway%E6%95%B0%E6%8D%AE%20Control_1-VS-Osteogenic_Medium_Treat_1.htm#gene180) | 1 (0.18%) | 0.9528137 | 1.000000000 | ko05144 |
| 181 | [Steroid hormone biosynthesis](file:///E:\01%20%E5%8D%9A%E5%A3%AB%E8%AE%BA%E6%96%87%E6%92%B0%E5%86%99%202016-2-1\1%20%E7%AC%AC%E4%B8%80%E9%83%A8%E5%88%86%20%EF%BC%88%E4%B8%89%EF%BC%89BMSCs%E4%B8%8E%E7%89%99%E8%83%9A%E7%BB%86%E8%83%9E%E4%BD%93%E5%A4%96%E9%97%B4%E6%8E%A5%E5%85%B1%E5%9F%B9%E5%85%BB%E6%A8%A1%E5%9E%8B%E4%B8%AD%E7%9A%84%E8%BD%AC%E5%BD%95%E7%BB%84%E8%A1%A8%E8%BE%BE%E8%B0%B1\pathway%E6%95%B0%E6%8D%AE%20Control_1-VS-Osteogenic_Medium_Treat_1.htm#gene181) | 1 (0.18%) | 0.9528137 | 1.000000000 | ko00140 |
| 182 | [Allograft rejection](file:///E:\01%20%E5%8D%9A%E5%A3%AB%E8%AE%BA%E6%96%87%E6%92%B0%E5%86%99%202016-2-1\1%20%E7%AC%AC%E4%B8%80%E9%83%A8%E5%88%86%20%EF%BC%88%E4%B8%89%EF%BC%89BMSCs%E4%B8%8E%E7%89%99%E8%83%9A%E7%BB%86%E8%83%9E%E4%BD%93%E5%A4%96%E9%97%B4%E6%8E%A5%E5%85%B1%E5%9F%B9%E5%85%BB%E6%A8%A1%E5%9E%8B%E4%B8%AD%E7%9A%84%E8%BD%AC%E5%BD%95%E7%BB%84%E8%A1%A8%E8%BE%BE%E8%B0%B1\pathway%E6%95%B0%E6%8D%AE%20Control_1-VS-Osteogenic_Medium_Treat_1.htm#gene182) | 1 (0.18%) | 0.9547015 | 1.000000000 | ko05330 |
| 183 | [Glycerolipid metabolism](file:///E:\01%20%E5%8D%9A%E5%A3%AB%E8%AE%BA%E6%96%87%E6%92%B0%E5%86%99%202016-2-1\1%20%E7%AC%AC%E4%B8%80%E9%83%A8%E5%88%86%20%EF%BC%88%E4%B8%89%EF%BC%89BMSCs%E4%B8%8E%E7%89%99%E8%83%9A%E7%BB%86%E8%83%9E%E4%BD%93%E5%A4%96%E9%97%B4%E6%8E%A5%E5%85%B1%E5%9F%B9%E5%85%BB%E6%A8%A1%E5%9E%8B%E4%B8%AD%E7%9A%84%E8%BD%AC%E5%BD%95%E7%BB%84%E8%A1%A8%E8%BE%BE%E8%B0%B1\pathway%E6%95%B0%E6%8D%AE%20Control_1-VS-Osteogenic_Medium_Treat_1.htm#gene183) | 1 (0.18%) | 0.9582541 | 1.000000000 | ko00561 |
| 184 | [Retrograde endocannabinoid signaling](file:///E:\01%20%E5%8D%9A%E5%A3%AB%E8%AE%BA%E6%96%87%E6%92%B0%E5%86%99%202016-2-1\1%20%E7%AC%AC%E4%B8%80%E9%83%A8%E5%88%86%20%EF%BC%88%E4%B8%89%EF%BC%89BMSCs%E4%B8%8E%E7%89%99%E8%83%9A%E7%BB%86%E8%83%9E%E4%BD%93%E5%A4%96%E9%97%B4%E6%8E%A5%E5%85%B1%E5%9F%B9%E5%85%BB%E6%A8%A1%E5%9E%8B%E4%B8%AD%E7%9A%84%E8%BD%AC%E5%BD%95%E7%BB%84%E8%A1%A8%E8%BE%BE%E8%B0%B1\pathway%E6%95%B0%E6%8D%AE%20Control_1-VS-Osteogenic_Medium_Treat_1.htm#gene184) | 2 (0.37%) | 0.9592957 | 1.000000000 | ko04723 |
| 185 | [Colorectal cancer](file:///E:\01%20%E5%8D%9A%E5%A3%AB%E8%AE%BA%E6%96%87%E6%92%B0%E5%86%99%202016-2-1\1%20%E7%AC%AC%E4%B8%80%E9%83%A8%E5%88%86%20%EF%BC%88%E4%B8%89%EF%BC%89BMSCs%E4%B8%8E%E7%89%99%E8%83%9A%E7%BB%86%E8%83%9E%E4%BD%93%E5%A4%96%E9%97%B4%E6%8E%A5%E5%85%B1%E5%9F%B9%E5%85%BB%E6%A8%A1%E5%9E%8B%E4%B8%AD%E7%9A%84%E8%BD%AC%E5%BD%95%E7%BB%84%E8%A1%A8%E8%BE%BE%E8%B0%B1\pathway%E6%95%B0%E6%8D%AE%20Control_1-VS-Osteogenic_Medium_Treat_1.htm#gene185) | 1 (0.18%) | 0.9599247 | 1.000000000 | ko05210 |
| 186 | [Natural killer cell mediated cytotoxicity](file:///E:\01%20%E5%8D%9A%E5%A3%AB%E8%AE%BA%E6%96%87%E6%92%B0%E5%86%99%202016-2-1\1%20%E7%AC%AC%E4%B8%80%E9%83%A8%E5%88%86%20%EF%BC%88%E4%B8%89%EF%BC%89BMSCs%E4%B8%8E%E7%89%99%E8%83%9A%E7%BB%86%E8%83%9E%E4%BD%93%E5%A4%96%E9%97%B4%E6%8E%A5%E5%85%B1%E5%9F%B9%E5%85%BB%E6%A8%A1%E5%9E%8B%E4%B8%AD%E7%9A%84%E8%BD%AC%E5%BD%95%E7%BB%84%E8%A1%A8%E8%BE%BE%E8%B0%B1\pathway%E6%95%B0%E6%8D%AE%20Control_1-VS-Osteogenic_Medium_Treat_1.htm#gene186) | 4 (0.73%) | 0.9624679 | 1.000000000 | ko04650 |
| 187 | [Antigen processing and presentation](file:///E:\01%20%E5%8D%9A%E5%A3%AB%E8%AE%BA%E6%96%87%E6%92%B0%E5%86%99%202016-2-1\1%20%E7%AC%AC%E4%B8%80%E9%83%A8%E5%88%86%20%EF%BC%88%E4%B8%89%EF%BC%89BMSCs%E4%B8%8E%E7%89%99%E8%83%9A%E7%BB%86%E8%83%9E%E4%BD%93%E5%A4%96%E9%97%B4%E6%8E%A5%E5%85%B1%E5%9F%B9%E5%85%BB%E6%A8%A1%E5%9E%8B%E4%B8%AD%E7%9A%84%E8%BD%AC%E5%BD%95%E7%BB%84%E8%A1%A8%E8%BE%BE%E8%B0%B1\pathway%E6%95%B0%E6%8D%AE%20Control_1-VS-Osteogenic_Medium_Treat_1.htm#gene187) | 2 (0.37%) | 0.9632625 | 1.000000000 | ko04612 |
| 188 | [Pathways in cancer](file:///E:\01%20%E5%8D%9A%E5%A3%AB%E8%AE%BA%E6%96%87%E6%92%B0%E5%86%99%202016-2-1\1%20%E7%AC%AC%E4%B8%80%E9%83%A8%E5%88%86%20%EF%BC%88%E4%B8%89%EF%BC%89BMSCs%E4%B8%8E%E7%89%99%E8%83%9A%E7%BB%86%E8%83%9E%E4%BD%93%E5%A4%96%E9%97%B4%E6%8E%A5%E5%85%B1%E5%9F%B9%E5%85%BB%E6%A8%A1%E5%9E%8B%E4%B8%AD%E7%9A%84%E8%BD%AC%E5%BD%95%E7%BB%84%E8%A1%A8%E8%BE%BE%E8%B0%B1\pathway%E6%95%B0%E6%8D%AE%20Control_1-VS-Osteogenic_Medium_Treat_1.htm#gene188) | 11 (2.02%) | 0.9634571 | 1.000000000 | ko05200 |
| 189 | [Autoimmune thyroid disease](file:///E:\01%20%E5%8D%9A%E5%A3%AB%E8%AE%BA%E6%96%87%E6%92%B0%E5%86%99%202016-2-1\1%20%E7%AC%AC%E4%B8%80%E9%83%A8%E5%88%86%20%EF%BC%88%E4%B8%89%EF%BC%89BMSCs%E4%B8%8E%E7%89%99%E8%83%9A%E7%BB%86%E8%83%9E%E4%BD%93%E5%A4%96%E9%97%B4%E6%8E%A5%E5%85%B1%E5%9F%B9%E5%85%BB%E6%A8%A1%E5%9E%8B%E4%B8%AD%E7%9A%84%E8%BD%AC%E5%BD%95%E7%BB%84%E8%A1%A8%E8%BE%BE%E8%B0%B1\pathway%E6%95%B0%E6%8D%AE%20Control_1-VS-Osteogenic_Medium_Treat_1.htm#gene189) | 1 (0.18%) | 0.9645465 | 1.000000000 | ko05320 |
| 190 | [Acute myeloid leukemia](file:///E:\01%20%E5%8D%9A%E5%A3%AB%E8%AE%BA%E6%96%87%E6%92%B0%E5%86%99%202016-2-1\1%20%E7%AC%AC%E4%B8%80%E9%83%A8%E5%88%86%20%EF%BC%88%E4%B8%89%EF%BC%89BMSCs%E4%B8%8E%E7%89%99%E8%83%9A%E7%BB%86%E8%83%9E%E4%BD%93%E5%A4%96%E9%97%B4%E6%8E%A5%E5%85%B1%E5%9F%B9%E5%85%BB%E6%A8%A1%E5%9E%8B%E4%B8%AD%E7%9A%84%E8%BD%AC%E5%BD%95%E7%BB%84%E8%A1%A8%E8%BE%BE%E8%B0%B1\pathway%E6%95%B0%E6%8D%AE%20Control_1-VS-Osteogenic_Medium_Treat_1.htm#gene190) | 1 (0.18%) | 0.9659657 | 1.000000000 | ko05221 |
| 191 | [Melanoma](file:///E:\01%20%E5%8D%9A%E5%A3%AB%E8%AE%BA%E6%96%87%E6%92%B0%E5%86%99%202016-2-1\1%20%E7%AC%AC%E4%B8%80%E9%83%A8%E5%88%86%20%EF%BC%88%E4%B8%89%EF%BC%89BMSCs%E4%B8%8E%E7%89%99%E8%83%9A%E7%BB%86%E8%83%9E%E4%BD%93%E5%A4%96%E9%97%B4%E6%8E%A5%E5%85%B1%E5%9F%B9%E5%85%BB%E6%A8%A1%E5%9E%8B%E4%B8%AD%E7%9A%84%E8%BD%AC%E5%BD%95%E7%BB%84%E8%A1%A8%E8%BE%BE%E8%B0%B1\pathway%E6%95%B0%E6%8D%AE%20Control_1-VS-Osteogenic_Medium_Treat_1.htm#gene191) | 1 (0.18%) | 0.9698919 | 1.000000000 | ko05218 |
| 192 | [Graft-versus-host disease](file:///E:\01%20%E5%8D%9A%E5%A3%AB%E8%AE%BA%E6%96%87%E6%92%B0%E5%86%99%202016-2-1\1%20%E7%AC%AC%E4%B8%80%E9%83%A8%E5%88%86%20%EF%BC%88%E4%B8%89%EF%BC%89BMSCs%E4%B8%8E%E7%89%99%E8%83%9A%E7%BB%86%E8%83%9E%E4%BD%93%E5%A4%96%E9%97%B4%E6%8E%A5%E5%85%B1%E5%9F%B9%E5%85%BB%E6%A8%A1%E5%9E%8B%E4%B8%AD%E7%9A%84%E8%BD%AC%E5%BD%95%E7%BB%84%E8%A1%A8%E8%BE%BE%E8%B0%B1\pathway%E6%95%B0%E6%8D%AE%20Control_1-VS-Osteogenic_Medium_Treat_1.htm#gene192) | 1 (0.18%) | 0.9698919 | 1.000000000 | ko05332 |
| 193 | [Renal cell carcinoma](file:///E:\01%20%E5%8D%9A%E5%A3%AB%E8%AE%BA%E6%96%87%E6%92%B0%E5%86%99%202016-2-1\1%20%E7%AC%AC%E4%B8%80%E9%83%A8%E5%88%86%20%EF%BC%88%E4%B8%89%EF%BC%89BMSCs%E4%B8%8E%E7%89%99%E8%83%9A%E7%BB%86%E8%83%9E%E4%BD%93%E5%A4%96%E9%97%B4%E6%8E%A5%E5%85%B1%E5%9F%B9%E5%85%BB%E6%A8%A1%E5%9E%8B%E4%B8%AD%E7%9A%84%E8%BD%AC%E5%BD%95%E7%BB%84%E8%A1%A8%E8%BE%BE%E8%B0%B1\pathway%E6%95%B0%E6%8D%AE%20Control_1-VS-Osteogenic_Medium_Treat_1.htm#gene193) | 1 (0.18%) | 0.9710975 | 1.000000000 | ko05211 |
| 194 | [Complement and coagulation cascades](file:///E:\01%20%E5%8D%9A%E5%A3%AB%E8%AE%BA%E6%96%87%E6%92%B0%E5%86%99%202016-2-1\1%20%E7%AC%AC%E4%B8%80%E9%83%A8%E5%88%86%20%EF%BC%88%E4%B8%89%EF%BC%89BMSCs%E4%B8%8E%E7%89%99%E8%83%9A%E7%BB%86%E8%83%9E%E4%BD%93%E5%A4%96%E9%97%B4%E6%8E%A5%E5%85%B1%E5%9F%B9%E5%85%BB%E6%A8%A1%E5%9E%8B%E4%B8%AD%E7%9A%84%E8%BD%AC%E5%BD%95%E7%BB%84%E8%A1%A8%E8%BE%BE%E8%B0%B1\pathway%E6%95%B0%E6%8D%AE%20Control_1-VS-Osteogenic_Medium_Treat_1.htm#gene194) | 2 (0.37%) | 0.9711268 | 1.000000000 | ko04610 |
| 195 | [Long-term depression](file:///E:\01%20%E5%8D%9A%E5%A3%AB%E8%AE%BA%E6%96%87%E6%92%B0%E5%86%99%202016-2-1\1%20%E7%AC%AC%E4%B8%80%E9%83%A8%E5%88%86%20%EF%BC%88%E4%B8%89%EF%BC%89BMSCs%E4%B8%8E%E7%89%99%E8%83%9A%E7%BB%86%E8%83%9E%E4%BD%93%E5%A4%96%E9%97%B4%E6%8E%A5%E5%85%B1%E5%9F%B9%E5%85%BB%E6%A8%A1%E5%9E%8B%E4%B8%AD%E7%9A%84%E8%BD%AC%E5%BD%95%E7%BB%84%E8%A1%A8%E8%BE%BE%E8%B0%B1\pathway%E6%95%B0%E6%8D%AE%20Control_1-VS-Osteogenic_Medium_Treat_1.htm#gene195) | 1 (0.18%) | 0.9722549 | 1.000000000 | ko04730 |
| 196 | [Staphylococcus aureus infection](file:///E:\01%20%E5%8D%9A%E5%A3%AB%E8%AE%BA%E6%96%87%E6%92%B0%E5%86%99%202016-2-1\1%20%E7%AC%AC%E4%B8%80%E9%83%A8%E5%88%86%20%EF%BC%88%E4%B8%89%EF%BC%89BMSCs%E4%B8%8E%E7%89%99%E8%83%9A%E7%BB%86%E8%83%9E%E4%BD%93%E5%A4%96%E9%97%B4%E6%8E%A5%E5%85%B1%E5%9F%B9%E5%85%BB%E6%A8%A1%E5%9E%8B%E4%B8%AD%E7%9A%84%E8%BD%AC%E5%BD%95%E7%BB%84%E8%A1%A8%E8%BE%BE%E8%B0%B1\pathway%E6%95%B0%E6%8D%AE%20Control_1-VS-Osteogenic_Medium_Treat_1.htm#gene196) | 1 (0.18%) | 0.9799945 | 1.000000000 | ko05150 |
| 197 | [Neuroactive ligand-receptor interaction](file:///E:\01%20%E5%8D%9A%E5%A3%AB%E8%AE%BA%E6%96%87%E6%92%B0%E5%86%99%202016-2-1\1%20%E7%AC%AC%E4%B8%80%E9%83%A8%E5%88%86%20%EF%BC%88%E4%B8%89%EF%BC%89BMSCs%E4%B8%8E%E7%89%99%E8%83%9A%E7%BB%86%E8%83%9E%E4%BD%93%E5%A4%96%E9%97%B4%E6%8E%A5%E5%85%B1%E5%9F%B9%E5%85%BB%E6%A8%A1%E5%9E%8B%E4%B8%AD%E7%9A%84%E8%BD%AC%E5%BD%95%E7%BB%84%E8%A1%A8%E8%BE%BE%E8%B0%B1\pathway%E6%95%B0%E6%8D%AE%20Control_1-VS-Osteogenic_Medium_Treat_1.htm#gene197) | 7 (1.28%) | 0.9820255 | 1.000000000 | ko04080 |
| 198 | [Pancreatic secretion](file:///E:\01%20%E5%8D%9A%E5%A3%AB%E8%AE%BA%E6%96%87%E6%92%B0%E5%86%99%202016-2-1\1%20%E7%AC%AC%E4%B8%80%E9%83%A8%E5%88%86%20%EF%BC%88%E4%B8%89%EF%BC%89BMSCs%E4%B8%8E%E7%89%99%E8%83%9A%E7%BB%86%E8%83%9E%E4%BD%93%E5%A4%96%E9%97%B4%E6%8E%A5%E5%85%B1%E5%9F%B9%E5%85%BB%E6%A8%A1%E5%9E%8B%E4%B8%AD%E7%9A%84%E8%BD%AC%E5%BD%95%E7%BB%84%E8%A1%A8%E8%BE%BE%E8%B0%B1\pathway%E6%95%B0%E6%8D%AE%20Control_1-VS-Osteogenic_Medium_Treat_1.htm#gene198) | 2 (0.37%) | 0.9840519 | 1.000000000 | ko04972 |
| 199 | [Pertussis](file:///E:\01%20%E5%8D%9A%E5%A3%AB%E8%AE%BA%E6%96%87%E6%92%B0%E5%86%99%202016-2-1\1%20%E7%AC%AC%E4%B8%80%E9%83%A8%E5%88%86%20%EF%BC%88%E4%B8%89%EF%BC%89BMSCs%E4%B8%8E%E7%89%99%E8%83%9A%E7%BB%86%E8%83%9E%E4%BD%93%E5%A4%96%E9%97%B4%E6%8E%A5%E5%85%B1%E5%9F%B9%E5%85%BB%E6%A8%A1%E5%9E%8B%E4%B8%AD%E7%9A%84%E8%BD%AC%E5%BD%95%E7%BB%84%E8%A1%A8%E8%BE%BE%E8%B0%B1\pathway%E6%95%B0%E6%8D%AE%20Control_1-VS-Osteogenic_Medium_Treat_1.htm#gene199) | 1 (0.18%) | 0.9843482 | 1.000000000 | ko05133 |
| 200 | [Metabolism of xenobiotics by cytochrome P450](file:///E:\01%20%E5%8D%9A%E5%A3%AB%E8%AE%BA%E6%96%87%E6%92%B0%E5%86%99%202016-2-1\1%20%E7%AC%AC%E4%B8%80%E9%83%A8%E5%88%86%20%EF%BC%88%E4%B8%89%EF%BC%89BMSCs%E4%B8%8E%E7%89%99%E8%83%9A%E7%BB%86%E8%83%9E%E4%BD%93%E5%A4%96%E9%97%B4%E6%8E%A5%E5%85%B1%E5%9F%B9%E5%85%BB%E6%A8%A1%E5%9E%8B%E4%B8%AD%E7%9A%84%E8%BD%AC%E5%BD%95%E7%BB%84%E8%A1%A8%E8%BE%BE%E8%B0%B1\pathway%E6%95%B0%E6%8D%AE%20Control_1-VS-Osteogenic_Medium_Treat_1.htm#gene200) | 1 (0.18%) | 0.9882468 | 1.000000000 | ko00980 |
| 201 | [Chemokine signaling pathway](file:///E:\01%20%E5%8D%9A%E5%A3%AB%E8%AE%BA%E6%96%87%E6%92%B0%E5%86%99%202016-2-1\1%20%E7%AC%AC%E4%B8%80%E9%83%A8%E5%88%86%20%EF%BC%88%E4%B8%89%EF%BC%89BMSCs%E4%B8%8E%E7%89%99%E8%83%9A%E7%BB%86%E8%83%9E%E4%BD%93%E5%A4%96%E9%97%B4%E6%8E%A5%E5%85%B1%E5%9F%B9%E5%85%BB%E6%A8%A1%E5%9E%8B%E4%B8%AD%E7%9A%84%E8%BD%AC%E5%BD%95%E7%BB%84%E8%A1%A8%E8%BE%BE%E8%B0%B1\pathway%E6%95%B0%E6%8D%AE%20Control_1-VS-Osteogenic_Medium_Treat_1.htm#gene201) | 4 (0.73%) | 0.9882615 | 1.000000000 | ko04062 |
| 202 | [Glycerophospholipid metabolism](file:///E:\01%20%E5%8D%9A%E5%A3%AB%E8%AE%BA%E6%96%87%E6%92%B0%E5%86%99%202016-2-1\1%20%E7%AC%AC%E4%B8%80%E9%83%A8%E5%88%86%20%EF%BC%88%E4%B8%89%EF%BC%89BMSCs%E4%B8%8E%E7%89%99%E8%83%9A%E7%BB%86%E8%83%9E%E4%BD%93%E5%A4%96%E9%97%B4%E6%8E%A5%E5%85%B1%E5%9F%B9%E5%85%BB%E6%A8%A1%E5%9E%8B%E4%B8%AD%E7%9A%84%E8%BD%AC%E5%BD%95%E7%BB%84%E8%A1%A8%E8%BE%BE%E8%B0%B1\pathway%E6%95%B0%E6%8D%AE%20Control_1-VS-Osteogenic_Medium_Treat_1.htm#gene202) | 1 (0.18%) | 0.9887182 | 1.000000000 | ko00564 |
| 203 | [Protein processing in endoplasmic reticulum](file:///E:\01%20%E5%8D%9A%E5%A3%AB%E8%AE%BA%E6%96%87%E6%92%B0%E5%86%99%202016-2-1\1%20%E7%AC%AC%E4%B8%80%E9%83%A8%E5%88%86%20%EF%BC%88%E4%B8%89%EF%BC%89BMSCs%E4%B8%8E%E7%89%99%E8%83%9A%E7%BB%86%E8%83%9E%E4%BD%93%E5%A4%96%E9%97%B4%E6%8E%A5%E5%85%B1%E5%9F%B9%E5%85%BB%E6%A8%A1%E5%9E%8B%E4%B8%AD%E7%9A%84%E8%BD%AC%E5%BD%95%E7%BB%84%E8%A1%A8%E8%BE%BE%E8%B0%B1\pathway%E6%95%B0%E6%8D%AE%20Control_1-VS-Osteogenic_Medium_Treat_1.htm#gene203) | 3 (0.55%) | 0.9903742 | 1.000000000 | ko04141 |
| 204 | [GABAergic synapse](file:///E:\01%20%E5%8D%9A%E5%A3%AB%E8%AE%BA%E6%96%87%E6%92%B0%E5%86%99%202016-2-1\1%20%E7%AC%AC%E4%B8%80%E9%83%A8%E5%88%86%20%EF%BC%88%E4%B8%89%EF%BC%89BMSCs%E4%B8%8E%E7%89%99%E8%83%9A%E7%BB%86%E8%83%9E%E4%BD%93%E5%A4%96%E9%97%B4%E6%8E%A5%E5%85%B1%E5%9F%B9%E5%85%BB%E6%A8%A1%E5%9E%8B%E4%B8%AD%E7%9A%84%E8%BD%AC%E5%BD%95%E7%BB%84%E8%A1%A8%E8%BE%BE%E8%B0%B1\pathway%E6%95%B0%E6%8D%AE%20Control_1-VS-Osteogenic_Medium_Treat_1.htm#gene204) | 1 (0.18%) | 0.9904225 | 1.000000000 | ko04727 |
| 205 | [Jak-STAT signaling pathway](file:///E:\01%20%E5%8D%9A%E5%A3%AB%E8%AE%BA%E6%96%87%E6%92%B0%E5%86%99%202016-2-1\1%20%E7%AC%AC%E4%B8%80%E9%83%A8%E5%88%86%20%EF%BC%88%E4%B8%89%EF%BC%89BMSCs%E4%B8%8E%E7%89%99%E8%83%9A%E7%BB%86%E8%83%9E%E4%BD%93%E5%A4%96%E9%97%B4%E6%8E%A5%E5%85%B1%E5%9F%B9%E5%85%BB%E6%A8%A1%E5%9E%8B%E4%B8%AD%E7%9A%84%E8%BD%AC%E5%BD%95%E7%BB%84%E8%A1%A8%E8%BE%BE%E8%B0%B1\pathway%E6%95%B0%E6%8D%AE%20Control_1-VS-Osteogenic_Medium_Treat_1.htm#gene205) | 2 (0.37%) | 0.9943394 | 1.000000000 | ko04630 |
| 206 | [B cell receptor signaling pathway](file:///E:\01%20%E5%8D%9A%E5%A3%AB%E8%AE%BA%E6%96%87%E6%92%B0%E5%86%99%202016-2-1\1%20%E7%AC%AC%E4%B8%80%E9%83%A8%E5%88%86%20%EF%BC%88%E4%B8%89%EF%BC%89BMSCs%E4%B8%8E%E7%89%99%E8%83%9A%E7%BB%86%E8%83%9E%E4%BD%93%E5%A4%96%E9%97%B4%E6%8E%A5%E5%85%B1%E5%9F%B9%E5%85%BB%E6%A8%A1%E5%9E%8B%E4%B8%AD%E7%9A%84%E8%BD%AC%E5%BD%95%E7%BB%84%E8%A1%A8%E8%BE%BE%E8%B0%B1\pathway%E6%95%B0%E6%8D%AE%20Control_1-VS-Osteogenic_Medium_Treat_1.htm#gene206) | 1 (0.18%) | 0.9943773 | 1.000000000 | ko04662 |
| 207 | [Prostate cancer](file:///E:\01%20%E5%8D%9A%E5%A3%AB%E8%AE%BA%E6%96%87%E6%92%B0%E5%86%99%202016-2-1\1%20%E7%AC%AC%E4%B8%80%E9%83%A8%E5%88%86%20%EF%BC%88%E4%B8%89%EF%BC%89BMSCs%E4%B8%8E%E7%89%99%E8%83%9A%E7%BB%86%E8%83%9E%E4%BD%93%E5%A4%96%E9%97%B4%E6%8E%A5%E5%85%B1%E5%9F%B9%E5%85%BB%E6%A8%A1%E5%9E%8B%E4%B8%AD%E7%9A%84%E8%BD%AC%E5%BD%95%E7%BB%84%E8%A1%A8%E8%BE%BE%E8%B0%B1\pathway%E6%95%B0%E6%8D%AE%20Control_1-VS-Osteogenic_Medium_Treat_1.htm#gene207) | 1 (0.18%) | 0.9961124 | 1.000000000 | ko05215 |
| 208 | [Cytokine-cytokine receptor interaction](file:///E:\01%20%E5%8D%9A%E5%A3%AB%E8%AE%BA%E6%96%87%E6%92%B0%E5%86%99%202016-2-1\1%20%E7%AC%AC%E4%B8%80%E9%83%A8%E5%88%86%20%EF%BC%88%E4%B8%89%EF%BC%89BMSCs%E4%B8%8E%E7%89%99%E8%83%9A%E7%BB%86%E8%83%9E%E4%BD%93%E5%A4%96%E9%97%B4%E6%8E%A5%E5%85%B1%E5%9F%B9%E5%85%BB%E6%A8%A1%E5%9E%8B%E4%B8%AD%E7%9A%84%E8%BD%AC%E5%BD%95%E7%BB%84%E8%A1%A8%E8%BE%BE%E8%B0%B1\pathway%E6%95%B0%E6%8D%AE%20Control_1-VS-Osteogenic_Medium_Treat_1.htm#gene208) | 3 (0.55%) | 0.9985263 | 1.000000000 | ko04060 |
| 209 | [Olfactory transduction](file:///E:\01%20%E5%8D%9A%E5%A3%AB%E8%AE%BA%E6%96%87%E6%92%B0%E5%86%99%202016-2-1\1%20%E7%AC%AC%E4%B8%80%E9%83%A8%E5%88%86%20%EF%BC%88%E4%B8%89%EF%BC%89BMSCs%E4%B8%8E%E7%89%99%E8%83%9A%E7%BB%86%E8%83%9E%E4%BD%93%E5%A4%96%E9%97%B4%E6%8E%A5%E5%85%B1%E5%9F%B9%E5%85%BB%E6%A8%A1%E5%9E%8B%E4%B8%AD%E7%9A%84%E8%BD%AC%E5%BD%95%E7%BB%84%E8%A1%A8%E8%BE%BE%E8%B0%B1\pathway%E6%95%B0%E6%8D%AE%20Control_1-VS-Osteogenic_Medium_Treat_1.htm#gene209) | 3 (0.55%) | 1 | 1.000000000 | ko04740 |
