# Supplementary material for: MagT1 regulated the odontogenic differentiation of BMMSCs induced byTGC-CM via ERK signaling pathway
Source: Stem Cell Res Ther. 2019 Jan 31;10:48. doi: 10.1186/s13287-019-1148-6 (PMC6357492; doi:10.1186/s13287-019-1148-6)
Supplement: Supplementary file 3 — Figure S3. The effect of U0126 in p-ERK/ERK during TGC-CM mediated odontogenic differentiation of BMMSCs for 7 days. U0126 significantly reduced the phosphorylated level of ERK. (PDF 245 kb) [file 13287_2019_1148_MOESM3_ESM.pdf]

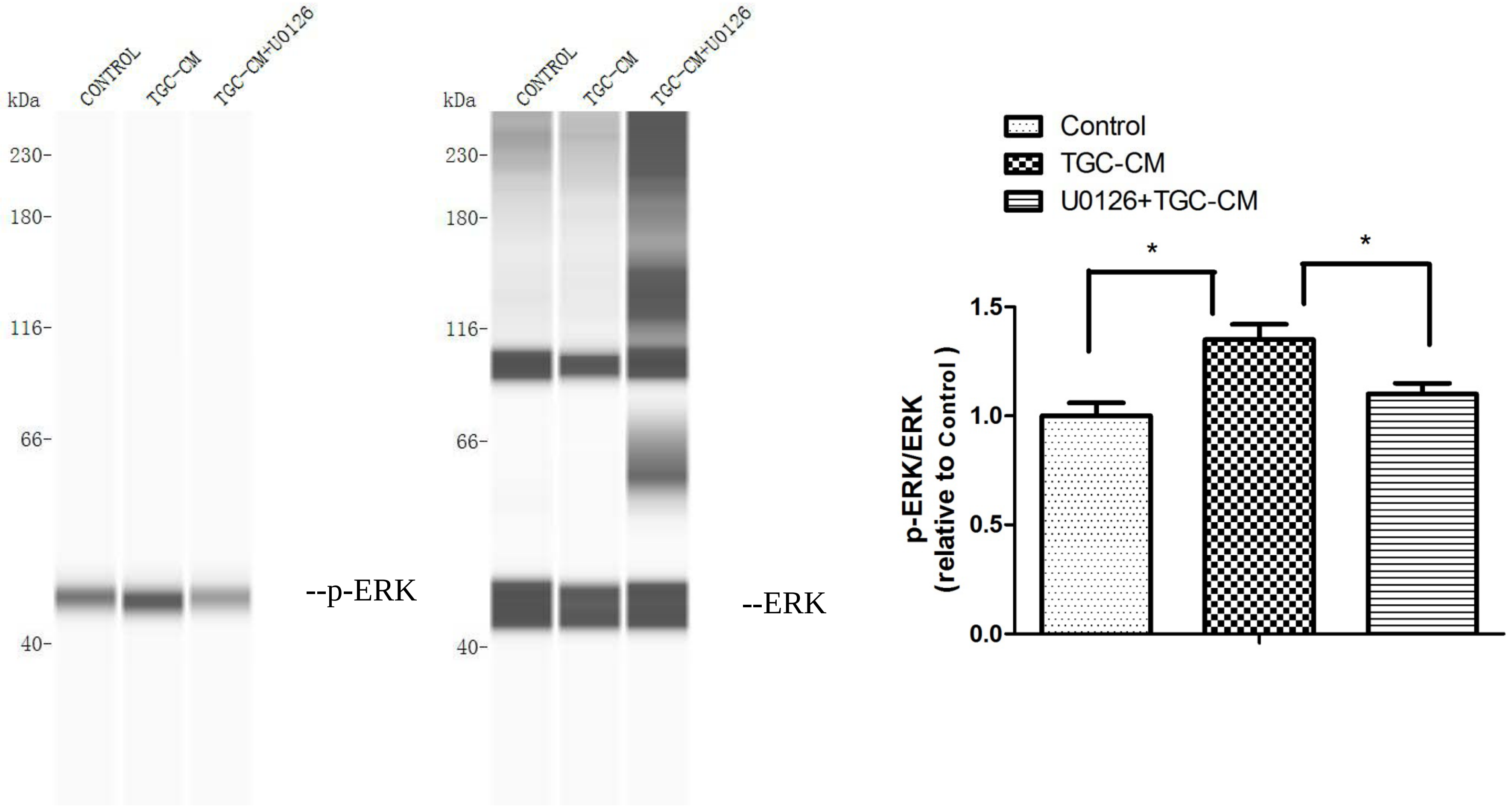

Figure S3. The effect of U0126 in p-ERK/ERK during TGC-CM mediated odontogenic differentiation of BMMSCs for 7 days. U0126 significantly reduced the phosphorylated level of ERK.
