# Supplementary material for: MagT1 regulated the odontogenic differentiation of BMMSCs induced byTGC-CM via ERK signaling pathway
Source: Stem Cell Res Ther. 2019 Jan 31;10:48. doi: 10.1186/s13287-019-1148-6 (PMC6357492; doi:10.1186/s13287-019-1148-6)
Supplement: Supplementary file 4 — Table S2. Pathway analysis of differentially expressed genes (DEGs) in MagT1-knockdown BMMSCs during odontogenic differentiation. All pathways are ordered according to the p value, and MAPK pathway is ranked 123. (DOCX 46 kb) [file 13287_2019_1148_MOESM4_ESM.docx]

**Table S2. Pathway analysis of differentially expressed genes (DEGs) in MagT1-konckdown BMMSCs during odontogenic differentiation.. All pathways are ordered according to the p value, and MAPK pathway is ranked 123.**

| **#** | **Pathway** | **Number of DEGs (percent of total DEGs)** | **P value** | **Q value** | **Pathway ID** |
| --- | --- | --- | --- | --- | --- |
| 1 | [PPAR signaling pathway](file:///E:\01%20%E5%8D%9A%E5%A3%AB%E8%AE%BA%E6%96%87%E6%92%B0%E5%86%99%202016-2-1\3%20%E7%AC%AC%E4%B8%89%E9%83%A8%E5%88%86%20%EF%BC%881%EF%BC%89%E8%BD%AC%E5%BD%95%E7%BB%84%E5%9F%BA%E5%9B%A0%E6%B5%8B%E5%BA%8F\control_shRNA_2-VS-Magt1_shRNA_2.htm#gene1) | 6 (2.61%) | 0.01851929 | 0.7796540 | ko03320 |
| 2 | [Phagosome](file:///E:\01%20%E5%8D%9A%E5%A3%AB%E8%AE%BA%E6%96%87%E6%92%B0%E5%86%99%202016-2-1\3%20%E7%AC%AC%E4%B8%89%E9%83%A8%E5%88%86%20%EF%BC%881%EF%BC%89%E8%BD%AC%E5%BD%95%E7%BB%84%E5%9F%BA%E5%9B%A0%E6%B5%8B%E5%BA%8F\control_shRNA_2-VS-Magt1_shRNA_2.htm#gene2) | 10 (4.35%) | 0.02489572 | 0.7796540 | ko04145 |
| 3 | [Viral myocarditis](file:///E:\01%20%E5%8D%9A%E5%A3%AB%E8%AE%BA%E6%96%87%E6%92%B0%E5%86%99%202016-2-1\3%20%E7%AC%AC%E4%B8%89%E9%83%A8%E5%88%86%20%EF%BC%881%EF%BC%89%E8%BD%AC%E5%BD%95%E7%BB%84%E5%9F%BA%E5%9B%A0%E6%B5%8B%E5%BA%8F\control_shRNA_2-VS-Magt1_shRNA_2.htm#gene3) | 8 (3.48%) | 0.02546931 | 0.7796540 | ko05416 |
| 4 | [Calcium signaling pathway](file:///E:\01%20%E5%8D%9A%E5%A3%AB%E8%AE%BA%E6%96%87%E6%92%B0%E5%86%99%202016-2-1\3%20%E7%AC%AC%E4%B8%89%E9%83%A8%E5%88%86%20%EF%BC%881%EF%BC%89%E8%BD%AC%E5%BD%95%E7%BB%84%E5%9F%BA%E5%9B%A0%E6%B5%8B%E5%BA%8F\control_shRNA_2-VS-Magt1_shRNA_2.htm#gene4) | 8 (3.48%) | 0.0316281 | 0.7796540 | ko04020 |
| 5 | [Allograft rejection](file:///E:\01%20%E5%8D%9A%E5%A3%AB%E8%AE%BA%E6%96%87%E6%92%B0%E5%86%99%202016-2-1\3%20%E7%AC%AC%E4%B8%89%E9%83%A8%E5%88%86%20%EF%BC%881%EF%BC%89%E8%BD%AC%E5%BD%95%E7%BB%84%E5%9F%BA%E5%9B%A0%E6%B5%8B%E5%BA%8F\control_shRNA_2-VS-Magt1_shRNA_2.htm#gene5) | 4 (1.74%) | 0.03936309 | 0.7796540 | ko05330 |
| 6 | [Glycine, serine and threonine metabolism](file:///E:\01%20%E5%8D%9A%E5%A3%AB%E8%AE%BA%E6%96%87%E6%92%B0%E5%86%99%202016-2-1\3%20%E7%AC%AC%E4%B8%89%E9%83%A8%E5%88%86%20%EF%BC%881%EF%BC%89%E8%BD%AC%E5%BD%95%E7%BB%84%E5%9F%BA%E5%9B%A0%E6%B5%8B%E5%BA%8F\control_shRNA_2-VS-Magt1_shRNA_2.htm#gene6) | 3 (1.3%) | 0.04696841 | 0.7796540 | ko00260 |
| 7 | [Autoimmune thyroid disease](file:///E:\01%20%E5%8D%9A%E5%A3%AB%E8%AE%BA%E6%96%87%E6%92%B0%E5%86%99%202016-2-1\3%20%E7%AC%AC%E4%B8%89%E9%83%A8%E5%88%86%20%EF%BC%881%EF%BC%89%E8%BD%AC%E5%BD%95%E7%BB%84%E5%9F%BA%E5%9B%A0%E6%B5%8B%E5%BA%8F\control_shRNA_2-VS-Magt1_shRNA_2.htm#gene7) | 4 (1.74%) | 0.04970277 | 0.7796540 | ko05320 |
| 8 | [Type I diabetes mellitus](file:///E:\01%20%E5%8D%9A%E5%A3%AB%E8%AE%BA%E6%96%87%E6%92%B0%E5%86%99%202016-2-1\3%20%E7%AC%AC%E4%B8%89%E9%83%A8%E5%88%86%20%EF%BC%881%EF%BC%89%E8%BD%AC%E5%BD%95%E7%BB%84%E5%9F%BA%E5%9B%A0%E6%B5%8B%E5%BA%8F\control_shRNA_2-VS-Magt1_shRNA_2.htm#gene8) | 4 (1.74%) | 0.05155793 | 0.7796540 | ko04940 |
| 9 | [Graft-versus-host disease](file:///E:\01%20%E5%8D%9A%E5%A3%AB%E8%AE%BA%E6%96%87%E6%92%B0%E5%86%99%202016-2-1\3%20%E7%AC%AC%E4%B8%89%E9%83%A8%E5%88%86%20%EF%BC%881%EF%BC%89%E8%BD%AC%E5%BD%95%E7%BB%84%E5%9F%BA%E5%9B%A0%E6%B5%8B%E5%BA%8F\control_shRNA_2-VS-Magt1_shRNA_2.htm#gene9) | 4 (1.74%) | 0.05734791 | 0.7796540 | ko05332 |
| 10 | [Cholinergic synapse](file:///E:\01%20%E5%8D%9A%E5%A3%AB%E8%AE%BA%E6%96%87%E6%92%B0%E5%86%99%202016-2-1\3%20%E7%AC%AC%E4%B8%89%E9%83%A8%E5%88%86%20%EF%BC%881%EF%BC%89%E8%BD%AC%E5%BD%95%E7%BB%84%E5%9F%BA%E5%9B%A0%E6%B5%8B%E5%BA%8F\control_shRNA_2-VS-Magt1_shRNA_2.htm#gene10) | 5 (2.17%) | 0.06900808 | 0.7796540 | ko04725 |
| 11 | [Dilated cardiomyopathy](file:///E:\01%20%E5%8D%9A%E5%A3%AB%E8%AE%BA%E6%96%87%E6%92%B0%E5%86%99%202016-2-1\3%20%E7%AC%AC%E4%B8%89%E9%83%A8%E5%88%86%20%EF%BC%881%EF%BC%89%E8%BD%AC%E5%BD%95%E7%BB%84%E5%9F%BA%E5%9B%A0%E6%B5%8B%E5%BA%8F\control_shRNA_2-VS-Magt1_shRNA_2.htm#gene11) | 7 (3.04%) | 0.06928858 | 0.7796540 | ko05414 |
| 12 | [ECM-receptor interaction](file:///E:\01%20%E5%8D%9A%E5%A3%AB%E8%AE%BA%E6%96%87%E6%92%B0%E5%86%99%202016-2-1\3%20%E7%AC%AC%E4%B8%89%E9%83%A8%E5%88%86%20%EF%BC%881%EF%BC%89%E8%BD%AC%E5%BD%95%E7%BB%84%E5%9F%BA%E5%9B%A0%E6%B5%8B%E5%BA%8F\control_shRNA_2-VS-Magt1_shRNA_2.htm#gene12) | 5 (2.17%) | 0.08217412 | 0.7796540 | ko04512 |
| 13 | [Gastric acid secretion](file:///E:\01%20%E5%8D%9A%E5%A3%AB%E8%AE%BA%E6%96%87%E6%92%B0%E5%86%99%202016-2-1\3%20%E7%AC%AC%E4%B8%89%E9%83%A8%E5%88%86%20%EF%BC%881%EF%BC%89%E8%BD%AC%E5%BD%95%E7%BB%84%E5%9F%BA%E5%9B%A0%E6%B5%8B%E5%BA%8F\control_shRNA_2-VS-Magt1_shRNA_2.htm#gene13) | 5 (2.17%) | 0.08616485 | 0.7796540 | ko04971 |
| 14 | [Fat digestion and absorption](file:///E:\01%20%E5%8D%9A%E5%A3%AB%E8%AE%BA%E6%96%87%E6%92%B0%E5%86%99%202016-2-1\3%20%E7%AC%AC%E4%B8%89%E9%83%A8%E5%88%86%20%EF%BC%881%EF%BC%89%E8%BD%AC%E5%BD%95%E7%BB%84%E5%9F%BA%E5%9B%A0%E6%B5%8B%E5%BA%8F\control_shRNA_2-VS-Magt1_shRNA_2.htm#gene14) | 3 (1.3%) | 0.08681175 | 0.7796540 | ko04975 |
| 15 | [Phototransduction - fly](file:///E:\01%20%E5%8D%9A%E5%A3%AB%E8%AE%BA%E6%96%87%E6%92%B0%E5%86%99%202016-2-1\3%20%E7%AC%AC%E4%B8%89%E9%83%A8%E5%88%86%20%EF%BC%881%EF%BC%89%E8%BD%AC%E5%BD%95%E7%BB%84%E5%9F%BA%E5%9B%A0%E6%B5%8B%E5%BA%8F\control_shRNA_2-VS-Magt1_shRNA_2.htm#gene15) | 3 (1.3%) | 0.09007231 | 0.7796540 | ko04745 |
| 16 | [Small cell lung cancer](file:///E:\01%20%E5%8D%9A%E5%A3%AB%E8%AE%BA%E6%96%87%E6%92%B0%E5%86%99%202016-2-1\3%20%E7%AC%AC%E4%B8%89%E9%83%A8%E5%88%86%20%EF%BC%881%EF%BC%89%E8%BD%AC%E5%BD%95%E7%BB%84%E5%9F%BA%E5%9B%A0%E6%B5%8B%E5%BA%8F\control_shRNA_2-VS-Magt1_shRNA_2.htm#gene16) | 4 (1.74%) | 0.09374077 | 0.7796540 | ko05222 |
| 17 | [Primary bile acid biosynthesis](file:///E:\01%20%E5%8D%9A%E5%A3%AB%E8%AE%BA%E6%96%87%E6%92%B0%E5%86%99%202016-2-1\3%20%E7%AC%AC%E4%B8%89%E9%83%A8%E5%88%86%20%EF%BC%881%EF%BC%89%E8%BD%AC%E5%BD%95%E7%BB%84%E5%9F%BA%E5%9B%A0%E6%B5%8B%E5%BA%8F\control_shRNA_2-VS-Magt1_shRNA_2.htm#gene17) | 2 (0.87%) | 0.09568672 | 0.7796540 | ko00120 |
| 18 | [Focal adhesion](file:///E:\01%20%E5%8D%9A%E5%A3%AB%E8%AE%BA%E6%96%87%E6%92%B0%E5%86%99%202016-2-1\3%20%E7%AC%AC%E4%B8%89%E9%83%A8%E5%88%86%20%EF%BC%881%EF%BC%89%E8%BD%AC%E5%BD%95%E7%BB%84%E5%9F%BA%E5%9B%A0%E6%B5%8B%E5%BA%8F\control_shRNA_2-VS-Magt1_shRNA_2.htm#gene18) | 9 (3.91%) | 0.09810502 | 0.7796540 | ko04510 |
| 19 | [Glycosphingolipid biosynthesis - lacto and neolacto series](file:///E:\01%20%E5%8D%9A%E5%A3%AB%E8%AE%BA%E6%96%87%E6%92%B0%E5%86%99%202016-2-1\3%20%E7%AC%AC%E4%B8%89%E9%83%A8%E5%88%86%20%EF%BC%881%EF%BC%89%E8%BD%AC%E5%BD%95%E7%BB%84%E5%9F%BA%E5%9B%A0%E6%B5%8B%E5%BA%8F\control_shRNA_2-VS-Magt1_shRNA_2.htm#gene19) | 2 (0.87%) | 0.1009747 | 0.7796540 | ko00601 |
| 20 | [Taste transduction](file:///E:\01%20%E5%8D%9A%E5%A3%AB%E8%AE%BA%E6%96%87%E6%92%B0%E5%86%99%202016-2-1\3%20%E7%AC%AC%E4%B8%89%E9%83%A8%E5%88%86%20%EF%BC%881%EF%BC%89%E8%BD%AC%E5%BD%95%E7%BB%84%E5%9F%BA%E5%9B%A0%E6%B5%8B%E5%BA%8F\control_shRNA_2-VS-Magt1_shRNA_2.htm#gene20) | 3 (1.3%) | 0.1036112 | 0.7796540 | ko04742 |
| 21 | [Amphetamine addiction](file:///E:\01%20%E5%8D%9A%E5%A3%AB%E8%AE%BA%E6%96%87%E6%92%B0%E5%86%99%202016-2-1\3%20%E7%AC%AC%E4%B8%89%E9%83%A8%E5%88%86%20%EF%BC%881%EF%BC%89%E8%BD%AC%E5%BD%95%E7%BB%84%E5%9F%BA%E5%9B%A0%E6%B5%8B%E5%BA%8F\control_shRNA_2-VS-Magt1_shRNA_2.htm#gene21) | 4 (1.74%) | 0.1096437 | 0.7796540 | ko05031 |
| 22 | [Amoebiasis](file:///E:\01%20%E5%8D%9A%E5%A3%AB%E8%AE%BA%E6%96%87%E6%92%B0%E5%86%99%202016-2-1\3%20%E7%AC%AC%E4%B8%89%E9%83%A8%E5%88%86%20%EF%BC%881%EF%BC%89%E8%BD%AC%E5%BD%95%E7%BB%84%E5%9F%BA%E5%9B%A0%E6%B5%8B%E5%BA%8F\control_shRNA_2-VS-Magt1_shRNA_2.htm#gene22) | 5 (2.17%) | 0.109875 | 0.7796540 | ko05146 |
| 23 | [Glutathione metabolism](file:///E:\01%20%E5%8D%9A%E5%A3%AB%E8%AE%BA%E6%96%87%E6%92%B0%E5%86%99%202016-2-1\3%20%E7%AC%AC%E4%B8%89%E9%83%A8%E5%88%86%20%EF%BC%881%EF%BC%89%E8%BD%AC%E5%BD%95%E7%BB%84%E5%9F%BA%E5%9B%A0%E6%B5%8B%E5%BA%8F\control_shRNA_2-VS-Magt1_shRNA_2.htm#gene23) | 3 (1.3%) | 0.1106653 | 0.7796540 | ko00480 |
| 24 | [Cyanoamino acid metabolism](file:///E:\01%20%E5%8D%9A%E5%A3%AB%E8%AE%BA%E6%96%87%E6%92%B0%E5%86%99%202016-2-1\3%20%E7%AC%AC%E4%B8%89%E9%83%A8%E5%88%86%20%EF%BC%881%EF%BC%89%E8%BD%AC%E5%BD%95%E7%BB%84%E5%9F%BA%E5%9B%A0%E6%B5%8B%E5%BA%8F\control_shRNA_2-VS-Magt1_shRNA_2.htm#gene24) | 1 (0.43%) | 0.1122292 | 0.7796540 | ko00460 |
| 25 | [Metabolism of xenobiotics by cytochrome P450](file:///E:\01%20%E5%8D%9A%E5%A3%AB%E8%AE%BA%E6%96%87%E6%92%B0%E5%86%99%202016-2-1\3%20%E7%AC%AC%E4%B8%89%E9%83%A8%E5%88%86%20%EF%BC%881%EF%BC%89%E8%BD%AC%E5%BD%95%E7%BB%84%E5%9F%BA%E5%9B%A0%E6%B5%8B%E5%BA%8F\control_shRNA_2-VS-Magt1_shRNA_2.htm#gene25) | 4 (1.74%) | 0.1124052 | 0.7796540 | ko00980 |
| 26 | [Cocaine addiction](file:///E:\01%20%E5%8D%9A%E5%A3%AB%E8%AE%BA%E6%96%87%E6%92%B0%E5%86%99%202016-2-1\3%20%E7%AC%AC%E4%B8%89%E9%83%A8%E5%88%86%20%EF%BC%881%EF%BC%89%E8%BD%AC%E5%BD%95%E7%BB%84%E5%9F%BA%E5%9B%A0%E6%B5%8B%E5%BA%8F\control_shRNA_2-VS-Magt1_shRNA_2.htm#gene26) | 3 (1.3%) | 0.1178987 | 0.7796540 | ko05030 |
| 27 | [mRNA surveillance pathway](file:///E:\01%20%E5%8D%9A%E5%A3%AB%E8%AE%BA%E6%96%87%E6%92%B0%E5%86%99%202016-2-1\3%20%E7%AC%AC%E4%B8%89%E9%83%A8%E5%88%86%20%EF%BC%881%EF%BC%89%E8%BD%AC%E5%BD%95%E7%BB%84%E5%9F%BA%E5%9B%A0%E6%B5%8B%E5%BA%8F\control_shRNA_2-VS-Magt1_shRNA_2.htm#gene27) | 4 (1.74%) | 0.1180201 | 0.7796540 | ko03015 |
| 28 | [Alzheimer's disease](file:///E:\01%20%E5%8D%9A%E5%A3%AB%E8%AE%BA%E6%96%87%E6%92%B0%E5%86%99%202016-2-1\3%20%E7%AC%AC%E4%B8%89%E9%83%A8%E5%88%86%20%EF%BC%881%EF%BC%89%E8%BD%AC%E5%BD%95%E7%BB%84%E5%9F%BA%E5%9B%A0%E6%B5%8B%E5%BA%8F\control_shRNA_2-VS-Magt1_shRNA_2.htm#gene28) | 6 (2.61%) | 0.118491 | 0.7796540 | ko05010 |
| 29 | [Progesterone-mediated oocyte maturation](file:///E:\01%20%E5%8D%9A%E5%A3%AB%E8%AE%BA%E6%96%87%E6%92%B0%E5%86%99%202016-2-1\3%20%E7%AC%AC%E4%B8%89%E9%83%A8%E5%88%86%20%EF%BC%881%EF%BC%89%E8%BD%AC%E5%BD%95%E7%BB%84%E5%9F%BA%E5%9B%A0%E6%B5%8B%E5%BA%8F\control_shRNA_2-VS-Magt1_shRNA_2.htm#gene29) | 4 (1.74%) | 0.126666 | 0.7796540 | ko04914 |
| 30 | [Purine metabolism](file:///E:\01%20%E5%8D%9A%E5%A3%AB%E8%AE%BA%E6%96%87%E6%92%B0%E5%86%99%202016-2-1\3%20%E7%AC%AC%E4%B8%89%E9%83%A8%E5%88%86%20%EF%BC%881%EF%BC%89%E8%BD%AC%E5%BD%95%E7%BB%84%E5%9F%BA%E5%9B%A0%E6%B5%8B%E5%BA%8F\control_shRNA_2-VS-Magt1_shRNA_2.htm#gene30) | 6 (2.61%) | 0.1290371 | 0.7796540 | ko00230 |
| 31 | [Nicotinate and nicotinamide metabolism](file:///E:\01%20%E5%8D%9A%E5%A3%AB%E8%AE%BA%E6%96%87%E6%92%B0%E5%86%99%202016-2-1\3%20%E7%AC%AC%E4%B8%89%E9%83%A8%E5%88%86%20%EF%BC%881%EF%BC%89%E8%BD%AC%E5%BD%95%E7%BB%84%E5%9F%BA%E5%9B%A0%E6%B5%8B%E5%BA%8F\control_shRNA_2-VS-Magt1_shRNA_2.htm#gene31) | 2 (0.87%) | 0.1399829 | 0.7796540 | ko00760 |
| 32 | [Toxoplasmosis](file:///E:\01%20%E5%8D%9A%E5%A3%AB%E8%AE%BA%E6%96%87%E6%92%B0%E5%86%99%202016-2-1\3%20%E7%AC%AC%E4%B8%89%E9%83%A8%E5%88%86%20%EF%BC%881%EF%BC%89%E8%BD%AC%E5%BD%95%E7%BB%84%E5%9F%BA%E5%9B%A0%E6%B5%8B%E5%BA%8F\control_shRNA_2-VS-Magt1_shRNA_2.htm#gene32) | 5 (2.17%) | 0.1467011 | 0.7796540 | ko05145 |
| 33 | [Hypertrophic cardiomyopathy (HCM)](file:///E:\01%20%E5%8D%9A%E5%A3%AB%E8%AE%BA%E6%96%87%E6%92%B0%E5%86%99%202016-2-1\3%20%E7%AC%AC%E4%B8%89%E9%83%A8%E5%88%86%20%EF%BC%881%EF%BC%89%E8%BD%AC%E5%BD%95%E7%BB%84%E5%9F%BA%E5%9B%A0%E6%B5%8B%E5%BA%8F\control_shRNA_2-VS-Magt1_shRNA_2.htm#gene33) | 6 (2.61%) | 0.1491117 | 0.7796540 | ko05410 |
| 34 | [Regulation of actin cytoskeleton](file:///E:\01%20%E5%8D%9A%E5%A3%AB%E8%AE%BA%E6%96%87%E6%92%B0%E5%86%99%202016-2-1\3%20%E7%AC%AC%E4%B8%89%E9%83%A8%E5%88%86%20%EF%BC%881%EF%BC%89%E8%BD%AC%E5%BD%95%E7%BB%84%E5%9F%BA%E5%9B%A0%E6%B5%8B%E5%BA%8F\control_shRNA_2-VS-Magt1_shRNA_2.htm#gene34) | 10 (4.35%) | 0.1491217 | 0.7796540 | ko04810 |
| 35 | [Measles](file:///E:\01%20%E5%8D%9A%E5%A3%AB%E8%AE%BA%E6%96%87%E6%92%B0%E5%86%99%202016-2-1\3%20%E7%AC%AC%E4%B8%89%E9%83%A8%E5%88%86%20%EF%BC%881%EF%BC%89%E8%BD%AC%E5%BD%95%E7%BB%84%E5%9F%BA%E5%9B%A0%E6%B5%8B%E5%BA%8F\control_shRNA_2-VS-Magt1_shRNA_2.htm#gene35) | 5 (2.17%) | 0.149326 | 0.7796540 | ko05162 |
| 36 | [Cysteine and methionine metabolism](file:///E:\01%20%E5%8D%9A%E5%A3%AB%E8%AE%BA%E6%96%87%E6%92%B0%E5%86%99%202016-2-1\3%20%E7%AC%AC%E4%B8%89%E9%83%A8%E5%88%86%20%EF%BC%881%EF%BC%89%E8%BD%AC%E5%BD%95%E7%BB%84%E5%9F%BA%E5%9B%A0%E6%B5%8B%E5%BA%8F\control_shRNA_2-VS-Magt1_shRNA_2.htm#gene36) | 2 (0.87%) | 0.1575614 | 0.7796540 | ko00270 |
| 37 | [Epstein-Barr virus infection](file:///E:\01%20%E5%8D%9A%E5%A3%AB%E8%AE%BA%E6%96%87%E6%92%B0%E5%86%99%202016-2-1\3%20%E7%AC%AC%E4%B8%89%E9%83%A8%E5%88%86%20%EF%BC%881%EF%BC%89%E8%BD%AC%E5%BD%95%E7%BB%84%E5%9F%BA%E5%9B%A0%E6%B5%8B%E5%BA%8F\control_shRNA_2-VS-Magt1_shRNA_2.htm#gene37) | 8 (3.48%) | 0.1597759 | 0.7796540 | ko05169 |
| 38 | [Antigen processing and presentation](file:///E:\01%20%E5%8D%9A%E5%A3%AB%E8%AE%BA%E6%96%87%E6%92%B0%E5%86%99%202016-2-1\3%20%E7%AC%AC%E4%B8%89%E9%83%A8%E5%88%86%20%EF%BC%881%EF%BC%89%E8%BD%AC%E5%BD%95%E7%BB%84%E5%9F%BA%E5%9B%A0%E6%B5%8B%E5%BA%8F\control_shRNA_2-VS-Magt1_shRNA_2.htm#gene38) | 4 (1.74%) | 0.1604924 | 0.7796540 | ko04612 |
| 39 | [Nicotine addiction](file:///E:\01%20%E5%8D%9A%E5%A3%AB%E8%AE%BA%E6%96%87%E6%92%B0%E5%86%99%202016-2-1\3%20%E7%AC%AC%E4%B8%89%E9%83%A8%E5%88%86%20%EF%BC%881%EF%BC%89%E8%BD%AC%E5%BD%95%E7%BB%84%E5%9F%BA%E5%9B%A0%E6%B5%8B%E5%BA%8F\control_shRNA_2-VS-Magt1_shRNA_2.htm#gene39) | 2 (0.87%) | 0.1694991 | 0.7796540 | ko05033 |
| 40 | [TGF-beta signaling pathway](file:///E:\01%20%E5%8D%9A%E5%A3%AB%E8%AE%BA%E6%96%87%E6%92%B0%E5%86%99%202016-2-1\3%20%E7%AC%AC%E4%B8%89%E9%83%A8%E5%88%86%20%EF%BC%881%EF%BC%89%E8%BD%AC%E5%BD%95%E7%BB%84%E5%9F%BA%E5%9B%A0%E6%B5%8B%E5%BA%8F\control_shRNA_2-VS-Magt1_shRNA_2.htm#gene40) | 4 (1.74%) | 0.1702449 | 0.7796540 | ko04350 |
| 41 | [Taurine and hypotaurine metabolism](file:///E:\01%20%E5%8D%9A%E5%A3%AB%E8%AE%BA%E6%96%87%E6%92%B0%E5%86%99%202016-2-1\3%20%E7%AC%AC%E4%B8%89%E9%83%A8%E5%88%86%20%EF%BC%881%EF%BC%89%E8%BD%AC%E5%BD%95%E7%BB%84%E5%9F%BA%E5%9B%A0%E6%B5%8B%E5%BA%8F\control_shRNA_2-VS-Magt1_shRNA_2.htm#gene41) | 1 (0.43%) | 0.1706336 | 0.7796540 | ko00430 |
| 42 | [Vitamin digestion and absorption](file:///E:\01%20%E5%8D%9A%E5%A3%AB%E8%AE%BA%E6%96%87%E6%92%B0%E5%86%99%202016-2-1\3%20%E7%AC%AC%E4%B8%89%E9%83%A8%E5%88%86%20%EF%BC%881%EF%BC%89%E8%BD%AC%E5%BD%95%E7%BB%84%E5%9F%BA%E5%9B%A0%E6%B5%8B%E5%BA%8F\control_shRNA_2-VS-Magt1_shRNA_2.htm#gene42) | 2 (0.87%) | 0.1755242 | 0.7796540 | ko04977 |
| 43 | [Base excision repair](file:///E:\01%20%E5%8D%9A%E5%A3%AB%E8%AE%BA%E6%96%87%E6%92%B0%E5%86%99%202016-2-1\3%20%E7%AC%AC%E4%B8%89%E9%83%A8%E5%88%86%20%EF%BC%881%EF%BC%89%E8%BD%AC%E5%BD%95%E7%BB%84%E5%9F%BA%E5%9B%A0%E6%B5%8B%E5%BA%8F\control_shRNA_2-VS-Magt1_shRNA_2.htm#gene43) | 2 (0.87%) | 0.1755242 | 0.7796540 | ko03410 |
| 44 | [Fatty acid biosynthesis](file:///E:\01%20%E5%8D%9A%E5%A3%AB%E8%AE%BA%E6%96%87%E6%92%B0%E5%86%99%202016-2-1\3%20%E7%AC%AC%E4%B8%89%E9%83%A8%E5%88%86%20%EF%BC%881%EF%BC%89%E8%BD%AC%E5%BD%95%E7%BB%84%E5%9F%BA%E5%9B%A0%E6%B5%8B%E5%BA%8F\control_shRNA_2-VS-Magt1_shRNA_2.htm#gene44) | 1 (0.43%) | 0.1846267 | 0.8014477 | ko00061 |
| 45 | [Linoleic acid metabolism](file:///E:\01%20%E5%8D%9A%E5%A3%AB%E8%AE%BA%E6%96%87%E6%92%B0%E5%86%99%202016-2-1\3%20%E7%AC%AC%E4%B8%89%E9%83%A8%E5%88%86%20%EF%BC%881%EF%BC%89%E8%BD%AC%E5%BD%95%E7%BB%84%E5%9F%BA%E5%9B%A0%E6%B5%8B%E5%BA%8F\control_shRNA_2-VS-Magt1_shRNA_2.htm#gene45) | 2 (0.87%) | 0.199933 | 0.8181931 | ko00591 |
| 46 | [Pathways in cancer](file:///E:\01%20%E5%8D%9A%E5%A3%AB%E8%AE%BA%E6%96%87%E6%92%B0%E5%86%99%202016-2-1\3%20%E7%AC%AC%E4%B8%89%E9%83%A8%E5%88%86%20%EF%BC%881%EF%BC%89%E8%BD%AC%E5%BD%95%E7%BB%84%E5%9F%BA%E5%9B%A0%E6%B5%8B%E5%BA%8F\control_shRNA_2-VS-Magt1_shRNA_2.htm#gene46) | 10 (4.35%) | 0.2021218 | 0.8181931 | ko05200 |
| 47 | [Vibrio cholerae infection](file:///E:\01%20%E5%8D%9A%E5%A3%AB%E8%AE%BA%E6%96%87%E6%92%B0%E5%86%99%202016-2-1\3%20%E7%AC%AC%E4%B8%89%E9%83%A8%E5%88%86%20%EF%BC%881%EF%BC%89%E8%BD%AC%E5%BD%95%E7%BB%84%E5%9F%BA%E5%9B%A0%E6%B5%8B%E5%BA%8F\control_shRNA_2-VS-Magt1_shRNA_2.htm#gene47) | 3 (1.3%) | 0.2070135 | 0.8181931 | ko05110 |
| 48 | [Amyotrophic lateral sclerosis (ALS)](file:///E:\01%20%E5%8D%9A%E5%A3%AB%E8%AE%BA%E6%96%87%E6%92%B0%E5%86%99%202016-2-1\3%20%E7%AC%AC%E4%B8%89%E9%83%A8%E5%88%86%20%EF%BC%881%EF%BC%89%E8%BD%AC%E5%BD%95%E7%BB%84%E5%9F%BA%E5%9B%A0%E6%B5%8B%E5%BA%8F\control_shRNA_2-VS-Magt1_shRNA_2.htm#gene48) | 3 (1.3%) | 0.2113851 | 0.8181931 | ko05014 |
| 49 | [Glycosaminoglycan biosynthesis - keratan sulfate](file:///E:\01%20%E5%8D%9A%E5%A3%AB%E8%AE%BA%E6%96%87%E6%92%B0%E5%86%99%202016-2-1\3%20%E7%AC%AC%E4%B8%89%E9%83%A8%E5%88%86%20%EF%BC%881%EF%BC%89%E8%BD%AC%E5%BD%95%E7%BB%84%E5%9F%BA%E5%9B%A0%E6%B5%8B%E5%BA%8F\control_shRNA_2-VS-Magt1_shRNA_2.htm#gene49) | 1 (0.43%) | 0.2119116 | 0.8181931 | ko00533 |
| 50 | [Phototransduction](file:///E:\01%20%E5%8D%9A%E5%A3%AB%E8%AE%BA%E6%96%87%E6%92%B0%E5%86%99%202016-2-1\3%20%E7%AC%AC%E4%B8%89%E9%83%A8%E5%88%86%20%EF%BC%881%EF%BC%89%E8%BD%AC%E5%BD%95%E7%BB%84%E5%9F%BA%E5%9B%A0%E6%B5%8B%E5%BA%8F\control_shRNA_2-VS-Magt1_shRNA_2.htm#gene50) | 2 (0.87%) | 0.2184887 | 0.8181931 | ko04744 |
| 51 | [Drug metabolism - cytochrome P450](file:///E:\01%20%E5%8D%9A%E5%A3%AB%E8%AE%BA%E6%96%87%E6%92%B0%E5%86%99%202016-2-1\3%20%E7%AC%AC%E4%B8%89%E9%83%A8%E5%88%86%20%EF%BC%881%EF%BC%89%E8%BD%AC%E5%BD%95%E7%BB%84%E5%9F%BA%E5%9B%A0%E6%B5%8B%E5%BA%8F\control_shRNA_2-VS-Magt1_shRNA_2.htm#gene51) | 3 (1.3%) | 0.2246174 | 0.8181931 | ko00982 |
| 52 | [Pyruvate metabolism](file:///E:\01%20%E5%8D%9A%E5%A3%AB%E8%AE%BA%E6%96%87%E6%92%B0%E5%86%99%202016-2-1\3%20%E7%AC%AC%E4%B8%89%E9%83%A8%E5%88%86%20%EF%BC%881%EF%BC%89%E8%BD%AC%E5%BD%95%E7%BB%84%E5%9F%BA%E5%9B%A0%E6%B5%8B%E5%BA%8F\control_shRNA_2-VS-Magt1_shRNA_2.htm#gene52) | 2 (0.87%) | 0.2371846 | 0.8181931 | ko00620 |
| 53 | [Sulfur metabolism](file:///E:\01%20%E5%8D%9A%E5%A3%AB%E8%AE%BA%E6%96%87%E6%92%B0%E5%86%99%202016-2-1\3%20%E7%AC%AC%E4%B8%89%E9%83%A8%E5%88%86%20%EF%BC%881%EF%BC%89%E8%BD%AC%E5%BD%95%E7%BB%84%E5%9F%BA%E5%9B%A0%E6%B5%8B%E5%BA%8F\control_shRNA_2-VS-Magt1_shRNA_2.htm#gene53) | 1 (0.43%) | 0.2382873 | 0.8181931 | ko00920 |
| 54 | [Oocyte meiosis](file:///E:\01%20%E5%8D%9A%E5%A3%AB%E8%AE%BA%E6%96%87%E6%92%B0%E5%86%99%202016-2-1\3%20%E7%AC%AC%E4%B8%89%E9%83%A8%E5%88%86%20%EF%BC%881%EF%BC%89%E8%BD%AC%E5%BD%95%E7%BB%84%E5%9F%BA%E5%9B%A0%E6%B5%8B%E5%BA%8F\control_shRNA_2-VS-Magt1_shRNA_2.htm#gene54) | 4 (1.74%) | 0.2434724 | 0.8181931 | ko04114 |
| 55 | [Synaptic vesicle cycle](file:///E:\01%20%E5%8D%9A%E5%A3%AB%E8%AE%BA%E6%96%87%E6%92%B0%E5%86%99%202016-2-1\3%20%E7%AC%AC%E4%B8%89%E9%83%A8%E5%88%86%20%EF%BC%881%EF%BC%89%E8%BD%AC%E5%BD%95%E7%BB%84%E5%9F%BA%E5%9B%A0%E6%B5%8B%E5%BA%8F\control_shRNA_2-VS-Magt1_shRNA_2.htm#gene55) | 3 (1.3%) | 0.2560629 | 0.8181931 | ko04721 |
| 56 | [Glutamatergic synapse](file:///E:\01%20%E5%8D%9A%E5%A3%AB%E8%AE%BA%E6%96%87%E6%92%B0%E5%86%99%202016-2-1\3%20%E7%AC%AC%E4%B8%89%E9%83%A8%E5%88%86%20%EF%BC%881%EF%BC%89%E8%BD%AC%E5%BD%95%E7%BB%84%E5%9F%BA%E5%9B%A0%E6%B5%8B%E5%BA%8F\control_shRNA_2-VS-Magt1_shRNA_2.htm#gene56) | 4 (1.74%) | 0.2618902 | 0.8181931 | ko04724 |
| 57 | [Systemic lupus erythematosus](file:///E:\01%20%E5%8D%9A%E5%A3%AB%E8%AE%BA%E6%96%87%E6%92%B0%E5%86%99%202016-2-1\3%20%E7%AC%AC%E4%B8%89%E9%83%A8%E5%88%86%20%EF%BC%881%EF%BC%89%E8%BD%AC%E5%BD%95%E7%BB%84%E5%9F%BA%E5%9B%A0%E6%B5%8B%E5%BA%8F\control_shRNA_2-VS-Magt1_shRNA_2.htm#gene57) | 3 (1.3%) | 0.2651626 | 0.8181931 | ko05322 |
| 58 | [Morphine addiction](file:///E:\01%20%E5%8D%9A%E5%A3%AB%E8%AE%BA%E6%96%87%E6%92%B0%E5%86%99%202016-2-1\3%20%E7%AC%AC%E4%B8%89%E9%83%A8%E5%88%86%20%EF%BC%881%EF%BC%89%E8%BD%AC%E5%BD%95%E7%BB%84%E5%9F%BA%E5%9B%A0%E6%B5%8B%E5%BA%8F\control_shRNA_2-VS-Magt1_shRNA_2.htm#gene58) | 3 (1.3%) | 0.2651626 | 0.8181931 | ko05032 |
| 59 | [Influenza A](file:///E:\01%20%E5%8D%9A%E5%A3%AB%E8%AE%BA%E6%96%87%E6%92%B0%E5%86%99%202016-2-1\3%20%E7%AC%AC%E4%B8%89%E9%83%A8%E5%88%86%20%EF%BC%881%EF%BC%89%E8%BD%AC%E5%BD%95%E7%BB%84%E5%9F%BA%E5%9B%A0%E6%B5%8B%E5%BA%8F\control_shRNA_2-VS-Magt1_shRNA_2.htm#gene59) | 6 (2.61%) | 0.2695758 | 0.8181931 | ko05164 |
| 60 | [Salmonella infection](file:///E:\01%20%E5%8D%9A%E5%A3%AB%E8%AE%BA%E6%96%87%E6%92%B0%E5%86%99%202016-2-1\3%20%E7%AC%AC%E4%B8%89%E9%83%A8%E5%88%86%20%EF%BC%881%EF%BC%89%E8%BD%AC%E5%BD%95%E7%BB%84%E5%9F%BA%E5%9B%A0%E6%B5%8B%E5%BA%8F\control_shRNA_2-VS-Magt1_shRNA_2.htm#gene60) | 5 (2.17%) | 0.2742403 | 0.8181931 | ko05132 |
| 61 | [Gap junction](file:///E:\01%20%E5%8D%9A%E5%A3%AB%E8%AE%BA%E6%96%87%E6%92%B0%E5%86%99%202016-2-1\3%20%E7%AC%AC%E4%B8%89%E9%83%A8%E5%88%86%20%EF%BC%881%EF%BC%89%E8%BD%AC%E5%BD%95%E7%BB%84%E5%9F%BA%E5%9B%A0%E6%B5%8B%E5%BA%8F\control_shRNA_2-VS-Magt1_shRNA_2.htm#gene61) | 3 (1.3%) | 0.2743013 | 0.8181931 | ko04540 |
| 62 | [Vascular smooth muscle contraction](file:///E:\01%20%E5%8D%9A%E5%A3%AB%E8%AE%BA%E6%96%87%E6%92%B0%E5%86%99%202016-2-1\3%20%E7%AC%AC%E4%B8%89%E9%83%A8%E5%88%86%20%EF%BC%881%EF%BC%89%E8%BD%AC%E5%BD%95%E7%BB%84%E5%9F%BA%E5%9B%A0%E6%B5%8B%E5%BA%8F\control_shRNA_2-VS-Magt1_shRNA_2.htm#gene62) | 6 (2.61%) | 0.2810991 | 0.8181931 | ko04270 |
| 63 | [Transcriptional misregulation in cancer](file:///E:\01%20%E5%8D%9A%E5%A3%AB%E8%AE%BA%E6%96%87%E6%92%B0%E5%86%99%202016-2-1\3%20%E7%AC%AC%E4%B8%89%E9%83%A8%E5%88%86%20%EF%BC%881%EF%BC%89%E8%BD%AC%E5%BD%95%E7%BB%84%E5%9F%BA%E5%9B%A0%E6%B5%8B%E5%BA%8F\control_shRNA_2-VS-Magt1_shRNA_2.htm#gene63) | 6 (2.61%) | 0.2839977 | 0.8181931 | ko05202 |
| 64 | [Glycosphingolipid biosynthesis - ganglio series](file:///E:\01%20%E5%8D%9A%E5%A3%AB%E8%AE%BA%E6%96%87%E6%92%B0%E5%86%99%202016-2-1\3%20%E7%AC%AC%E4%B8%89%E9%83%A8%E5%88%86%20%EF%BC%881%EF%BC%89%E8%BD%AC%E5%BD%95%E7%BB%84%E5%9F%BA%E5%9B%A0%E6%B5%8B%E5%BA%8F\control_shRNA_2-VS-Magt1_shRNA_2.htm#gene64) | 1 (0.43%) | 0.2884309 | 0.8181931 | ko00604 |
| 65 | [Adipocytokine signaling pathway](file:///E:\01%20%E5%8D%9A%E5%A3%AB%E8%AE%BA%E6%96%87%E6%92%B0%E5%86%99%202016-2-1\3%20%E7%AC%AC%E4%B8%89%E9%83%A8%E5%88%86%20%EF%BC%881%EF%BC%89%E8%BD%AC%E5%BD%95%E7%BB%84%E5%9F%BA%E5%9B%A0%E6%B5%8B%E5%BA%8F\control_shRNA_2-VS-Magt1_shRNA_2.htm#gene65) | 3 (1.3%) | 0.2926703 | 0.8181931 | ko04920 |
| 66 | [Fructose and mannose metabolism](file:///E:\01%20%E5%8D%9A%E5%A3%AB%E8%AE%BA%E6%96%87%E6%92%B0%E5%86%99%202016-2-1\3%20%E7%AC%AC%E4%B8%89%E9%83%A8%E5%88%86%20%EF%BC%881%EF%BC%89%E8%BD%AC%E5%BD%95%E7%BB%84%E5%9F%BA%E5%9B%A0%E6%B5%8B%E5%BA%8F\control_shRNA_2-VS-Magt1_shRNA_2.htm#gene66) | 2 (0.87%) | 0.2935456 | 0.8181931 | ko00051 |
| 67 | [Arrhythmogenic right ventricular cardiomyopathy (ARVC)](file:///E:\01%20%E5%8D%9A%E5%A3%AB%E8%AE%BA%E6%96%87%E6%92%B0%E5%86%99%202016-2-1\3%20%E7%AC%AC%E4%B8%89%E9%83%A8%E5%88%86%20%EF%BC%881%EF%BC%89%E8%BD%AC%E5%BD%95%E7%BB%84%E5%9F%BA%E5%9B%A0%E6%B5%8B%E5%BA%8F\control_shRNA_2-VS-Magt1_shRNA_2.htm#gene67) | 3 (1.3%) | 0.3018881 | 0.8181931 | ko05412 |
| 68 | [Aldosterone-regulated sodium reabsorption](file:///E:\01%20%E5%8D%9A%E5%A3%AB%E8%AE%BA%E6%96%87%E6%92%B0%E5%86%99%202016-2-1\3%20%E7%AC%AC%E4%B8%89%E9%83%A8%E5%88%86%20%EF%BC%881%EF%BC%89%E8%BD%AC%E5%BD%95%E7%BB%84%E5%9F%BA%E5%9B%A0%E6%B5%8B%E5%BA%8F\control_shRNA_2-VS-Magt1_shRNA_2.htm#gene68) | 2 (0.87%) | 0.3060319 | 0.8181931 | ko04960 |
| 69 | [Arachidonic acid metabolism](file:///E:\01%20%E5%8D%9A%E5%A3%AB%E8%AE%BA%E6%96%87%E6%92%B0%E5%86%99%202016-2-1\3%20%E7%AC%AC%E4%B8%89%E9%83%A8%E5%88%86%20%EF%BC%881%EF%BC%89%E8%BD%AC%E5%BD%95%E7%BB%84%E5%9F%BA%E5%9B%A0%E6%B5%8B%E5%BA%8F\control_shRNA_2-VS-Magt1_shRNA_2.htm#gene69) | 3 (1.3%) | 0.3065028 | 0.8181931 | ko00590 |
| 70 | [Alcoholism](file:///E:\01%20%E5%8D%9A%E5%A3%AB%E8%AE%BA%E6%96%87%E6%92%B0%E5%86%99%202016-2-1\3%20%E7%AC%AC%E4%B8%89%E9%83%A8%E5%88%86%20%EF%BC%881%EF%BC%89%E8%BD%AC%E5%BD%95%E7%BB%84%E5%9F%BA%E5%9B%A0%E6%B5%8B%E5%BA%8F\control_shRNA_2-VS-Magt1_shRNA_2.htm#gene70) | 4 (1.74%) | 0.307038 | 0.8181931 | ko05034 |
| 71 | [Glycosaminoglycan biosynthesis - chondroitin sulfate](file:///E:\01%20%E5%8D%9A%E5%A3%AB%E8%AE%BA%E6%96%87%E6%92%B0%E5%86%99%202016-2-1\3%20%E7%AC%AC%E4%B8%89%E9%83%A8%E5%88%86%20%EF%BC%881%EF%BC%89%E8%BD%AC%E5%BD%95%E7%BB%84%E5%9F%BA%E5%9B%A0%E6%B5%8B%E5%BA%8F\control_shRNA_2-VS-Magt1_shRNA_2.htm#gene71) | 1 (0.43%) | 0.3122561 | 0.8181931 | ko00532 |
| 72 | [Circadian rhythm - mammal](file:///E:\01%20%E5%8D%9A%E5%A3%AB%E8%AE%BA%E6%96%87%E6%92%B0%E5%86%99%202016-2-1\3%20%E7%AC%AC%E4%B8%89%E9%83%A8%E5%88%86%20%EF%BC%881%EF%BC%89%E8%BD%AC%E5%BD%95%E7%BB%84%E5%9F%BA%E5%9B%A0%E6%B5%8B%E5%BA%8F\control_shRNA_2-VS-Magt1_shRNA_2.htm#gene72) | 1 (0.43%) | 0.3238691 | 0.8181931 | ko04710 |
| 73 | [Steroid biosynthesis](file:///E:\01%20%E5%8D%9A%E5%A3%AB%E8%AE%BA%E6%96%87%E6%92%B0%E5%86%99%202016-2-1\3%20%E7%AC%AC%E4%B8%89%E9%83%A8%E5%88%86%20%EF%BC%881%EF%BC%89%E8%BD%AC%E5%BD%95%E7%BB%84%E5%9F%BA%E5%9B%A0%E6%B5%8B%E5%BA%8F\control_shRNA_2-VS-Magt1_shRNA_2.htm#gene73) | 1 (0.43%) | 0.3238691 | 0.8181931 | ko00100 |
| 74 | [Dopaminergic synapse](file:///E:\01%20%E5%8D%9A%E5%A3%AB%E8%AE%BA%E6%96%87%E6%92%B0%E5%86%99%202016-2-1\3%20%E7%AC%AC%E4%B8%89%E9%83%A8%E5%88%86%20%EF%BC%881%EF%BC%89%E8%BD%AC%E5%BD%95%E7%BB%84%E5%9F%BA%E5%9B%A0%E6%B5%8B%E5%BA%8F\control_shRNA_2-VS-Magt1_shRNA_2.htm#gene74) | 4 (1.74%) | 0.3261068 | 0.8181931 | ko04728 |
| 75 | [Neuroactive ligand-receptor interaction](file:///E:\01%20%E5%8D%9A%E5%A3%AB%E8%AE%BA%E6%96%87%E6%92%B0%E5%86%99%202016-2-1\3%20%E7%AC%AC%E4%B8%89%E9%83%A8%E5%88%86%20%EF%BC%881%EF%BC%89%E8%BD%AC%E5%BD%95%E7%BB%84%E5%9F%BA%E5%9B%A0%E6%B5%8B%E5%BA%8F\control_shRNA_2-VS-Magt1_shRNA_2.htm#gene75) | 7 (3.04%) | 0.3306044 | 0.8181931 | ko04080 |
| 76 | [Retrograde endocannabinoid signaling](file:///E:\01%20%E5%8D%9A%E5%A3%AB%E8%AE%BA%E6%96%87%E6%92%B0%E5%86%99%202016-2-1\3%20%E7%AC%AC%E4%B8%89%E9%83%A8%E5%88%86%20%EF%BC%881%EF%BC%89%E8%BD%AC%E5%BD%95%E7%BB%84%E5%9F%BA%E5%9B%A0%E6%B5%8B%E5%BA%8F\control_shRNA_2-VS-Magt1_shRNA_2.htm#gene76) | 3 (1.3%) | 0.3342269 | 0.8181931 | ko04723 |
| 77 | [Type II diabetes mellitus](file:///E:\01%20%E5%8D%9A%E5%A3%AB%E8%AE%BA%E6%96%87%E6%92%B0%E5%86%99%202016-2-1\3%20%E7%AC%AC%E4%B8%89%E9%83%A8%E5%88%86%20%EF%BC%881%EF%BC%89%E8%BD%AC%E5%BD%95%E7%BB%84%E5%9F%BA%E5%9B%A0%E6%B5%8B%E5%BA%8F\control_shRNA_2-VS-Magt1_shRNA_2.htm#gene77) | 2 (0.87%) | 0.343198 | 0.8181931 | ko04930 |
| 78 | [Glycosaminoglycan degradation](file:///E:\01%20%E5%8D%9A%E5%A3%AB%E8%AE%BA%E6%96%87%E6%92%B0%E5%86%99%202016-2-1\3%20%E7%AC%AC%E4%B8%89%E9%83%A8%E5%88%86%20%EF%BC%881%EF%BC%89%E8%BD%AC%E5%BD%95%E7%BB%84%E5%9F%BA%E5%9B%A0%E6%B5%8B%E5%BA%8F\control_shRNA_2-VS-Magt1_shRNA_2.htm#gene78) | 1 (0.43%) | 0.3465127 | 0.8181931 | ko00531 |
| 79 | [Salivary secretion](file:///E:\01%20%E5%8D%9A%E5%A3%AB%E8%AE%BA%E6%96%87%E6%92%B0%E5%86%99%202016-2-1\3%20%E7%AC%AC%E4%B8%89%E9%83%A8%E5%88%86%20%EF%BC%881%EF%BC%89%E8%BD%AC%E5%BD%95%E7%BB%84%E5%9F%BA%E5%9B%A0%E6%B5%8B%E5%BA%8F\control_shRNA_2-VS-Magt1_shRNA_2.htm#gene79) | 3 (1.3%) | 0.3526945 | 0.8181931 | ko04970 |
| 80 | [Shigellosis](file:///E:\01%20%E5%8D%9A%E5%A3%AB%E8%AE%BA%E6%96%87%E6%92%B0%E5%86%99%202016-2-1\3%20%E7%AC%AC%E4%B8%89%E9%83%A8%E5%88%86%20%EF%BC%881%EF%BC%89%E8%BD%AC%E5%BD%95%E7%BB%84%E5%9F%BA%E5%9B%A0%E6%B5%8B%E5%BA%8F\control_shRNA_2-VS-Magt1_shRNA_2.htm#gene80) | 3 (1.3%) | 0.3573036 | 0.8181931 | ko05131 |
| 81 | [Phenylalanine metabolism](file:///E:\01%20%E5%8D%9A%E5%A3%AB%E8%AE%BA%E6%96%87%E6%92%B0%E5%86%99%202016-2-1\3%20%E7%AC%AC%E4%B8%89%E9%83%A8%E5%88%86%20%EF%BC%881%EF%BC%89%E8%BD%AC%E5%BD%95%E7%BB%84%E5%9F%BA%E5%9B%A0%E6%B5%8B%E5%BA%8F\control_shRNA_2-VS-Magt1_shRNA_2.htm#gene81) | 1 (0.43%) | 0.3575497 | 0.8181931 | ko00360 |
| 82 | [Malaria](file:///E:\01%20%E5%8D%9A%E5%A3%AB%E8%AE%BA%E6%96%87%E6%92%B0%E5%86%99%202016-2-1\3%20%E7%AC%AC%E4%B8%89%E9%83%A8%E5%88%86%20%EF%BC%881%EF%BC%89%E8%BD%AC%E5%BD%95%E7%BB%84%E5%9F%BA%E5%9B%A0%E6%B5%8B%E5%BA%8F\control_shRNA_2-VS-Magt1_shRNA_2.htm#gene82) | 2 (0.87%) | 0.3615527 | 0.8181931 | ko05144 |
| 83 | [Endocytosis](file:///E:\01%20%E5%8D%9A%E5%A3%AB%E8%AE%BA%E6%96%87%E6%92%B0%E5%86%99%202016-2-1\3%20%E7%AC%AC%E4%B8%89%E9%83%A8%E5%88%86%20%EF%BC%881%EF%BC%89%E8%BD%AC%E5%BD%95%E7%BB%84%E5%9F%BA%E5%9B%A0%E6%B5%8B%E5%BA%8F\control_shRNA_2-VS-Magt1_shRNA_2.htm#gene83) | 7 (3.04%) | 0.3662735 | 0.8181931 | ko04144 |
| 84 | [Glycosylphosphatidylinositol(GPI)-anchor biosynthesis](file:///E:\01%20%E5%8D%9A%E5%A3%AB%E8%AE%BA%E6%96%87%E6%92%B0%E5%86%99%202016-2-1\3%20%E7%AC%AC%E4%B8%89%E9%83%A8%E5%88%86%20%EF%BC%881%EF%BC%89%E8%BD%AC%E5%BD%95%E7%BB%84%E5%9F%BA%E5%9B%A0%E6%B5%8B%E5%BA%8F\control_shRNA_2-VS-Magt1_shRNA_2.htm#gene84) | 1 (0.43%) | 0.3684011 | 0.8181931 | ko00563 |
| 85 | [Glycosaminoglycan biosynthesis - heparan sulfate](file:///E:\01%20%E5%8D%9A%E5%A3%AB%E8%AE%BA%E6%96%87%E6%92%B0%E5%86%99%202016-2-1\3%20%E7%AC%AC%E4%B8%89%E9%83%A8%E5%88%86%20%EF%BC%881%EF%BC%89%E8%BD%AC%E5%BD%95%E7%BB%84%E5%9F%BA%E5%9B%A0%E6%B5%8B%E5%BA%8F\control_shRNA_2-VS-Magt1_shRNA_2.htm#gene85) | 1 (0.43%) | 0.3684011 | 0.8181931 | ko00534 |
| 86 | [Renin-angiotensin system](file:///E:\01%20%E5%8D%9A%E5%A3%AB%E8%AE%BA%E6%96%87%E6%92%B0%E5%86%99%202016-2-1\3%20%E7%AC%AC%E4%B8%89%E9%83%A8%E5%88%86%20%EF%BC%881%EF%BC%89%E8%BD%AC%E5%BD%95%E7%BB%84%E5%9F%BA%E5%9B%A0%E6%B5%8B%E5%BA%8F\control_shRNA_2-VS-Magt1_shRNA_2.htm#gene86) | 1 (0.43%) | 0.3684011 | 0.8181931 | ko04614 |
| 87 | [Parkinson's disease](file:///E:\01%20%E5%8D%9A%E5%A3%AB%E8%AE%BA%E6%96%87%E6%92%B0%E5%86%99%202016-2-1\3%20%E7%AC%AC%E4%B8%89%E9%83%A8%E5%88%86%20%EF%BC%881%EF%BC%89%E8%BD%AC%E5%BD%95%E7%BB%84%E5%9F%BA%E5%9B%A0%E6%B5%8B%E5%BA%8F\control_shRNA_2-VS-Magt1_shRNA_2.htm#gene87) | 3 (1.3%) | 0.3894148 | 0.8245419 | ko05012 |
| 88 | [Neurotrophin signaling pathway](file:///E:\01%20%E5%8D%9A%E5%A3%AB%E8%AE%BA%E6%96%87%E6%92%B0%E5%86%99%202016-2-1\3%20%E7%AC%AC%E4%B8%89%E9%83%A8%E5%88%86%20%EF%BC%881%EF%BC%89%E8%BD%AC%E5%BD%95%E7%BB%84%E5%9F%BA%E5%9B%A0%E6%B5%8B%E5%BA%8F\control_shRNA_2-VS-Magt1_shRNA_2.htm#gene88) | 4 (1.74%) | 0.4064591 | 0.8245419 | ko04722 |
| 89 | [Prostate cancer](file:///E:\01%20%E5%8D%9A%E5%A3%AB%E8%AE%BA%E6%96%87%E6%92%B0%E5%86%99%202016-2-1\3%20%E7%AC%AC%E4%B8%89%E9%83%A8%E5%88%86%20%EF%BC%881%EF%BC%89%E8%BD%AC%E5%BD%95%E7%BB%84%E5%9F%BA%E5%9B%A0%E6%B5%8B%E5%BA%8F\control_shRNA_2-VS-Magt1_shRNA_2.htm#gene89) | 3 (1.3%) | 0.407596 | 0.8245419 | ko05215 |
| 90 | [GnRH signaling pathway](file:///E:\01%20%E5%8D%9A%E5%A3%AB%E8%AE%BA%E6%96%87%E6%92%B0%E5%86%99%202016-2-1\3%20%E7%AC%AC%E4%B8%89%E9%83%A8%E5%88%86%20%EF%BC%881%EF%BC%89%E8%BD%AC%E5%BD%95%E7%BB%84%E5%9F%BA%E5%9B%A0%E6%B5%8B%E5%BA%8F\control_shRNA_2-VS-Magt1_shRNA_2.htm#gene90) | 3 (1.3%) | 0.407596 | 0.8245419 | ko04912 |
| 91 | [Bacterial invasion of epithelial cells](file:///E:\01%20%E5%8D%9A%E5%A3%AB%E8%AE%BA%E6%96%87%E6%92%B0%E5%86%99%202016-2-1\3%20%E7%AC%AC%E4%B8%89%E9%83%A8%E5%88%86%20%EF%BC%881%EF%BC%89%E8%BD%AC%E5%BD%95%E7%BB%84%E5%9F%BA%E5%9B%A0%E6%B5%8B%E5%BA%8F\control_shRNA_2-VS-Magt1_shRNA_2.htm#gene91) | 3 (1.3%) | 0.4121171 | 0.8245419 | ko05100 |
| 92 | [Acute myeloid leukemia](file:///E:\01%20%E5%8D%9A%E5%A3%AB%E8%AE%BA%E6%96%87%E6%92%B0%E5%86%99%202016-2-1\3%20%E7%AC%AC%E4%B8%89%E9%83%A8%E5%88%86%20%EF%BC%881%EF%BC%89%E8%BD%AC%E5%BD%95%E7%BB%84%E5%9F%BA%E5%9B%A0%E6%B5%8B%E5%BA%8F\control_shRNA_2-VS-Magt1_shRNA_2.htm#gene92) | 2 (0.87%) | 0.4153578 | 0.8245419 | ko05221 |
| 93 | [Retinol metabolism](file:///E:\01%20%E5%8D%9A%E5%A3%AB%E8%AE%BA%E6%96%87%E6%92%B0%E5%86%99%202016-2-1\3%20%E7%AC%AC%E4%B8%89%E9%83%A8%E5%88%86%20%EF%BC%881%EF%BC%89%E8%BD%AC%E5%BD%95%E7%BB%84%E5%9F%BA%E5%9B%A0%E6%B5%8B%E5%BA%8F\control_shRNA_2-VS-Magt1_shRNA_2.htm#gene93) | 2 (0.87%) | 0.4153578 | 0.8245419 | ko00830 |
| 94 | [Insulin signaling pathway](file:///E:\01%20%E5%8D%9A%E5%A3%AB%E8%AE%BA%E6%96%87%E6%92%B0%E5%86%99%202016-2-1\3%20%E7%AC%AC%E4%B8%89%E9%83%A8%E5%88%86%20%EF%BC%881%EF%BC%89%E8%BD%AC%E5%BD%95%E7%BB%84%E5%9F%BA%E5%9B%A0%E6%B5%8B%E5%BA%8F\control_shRNA_2-VS-Magt1_shRNA_2.htm#gene94) | 4 (1.74%) | 0.4178471 | 0.8245419 | ko04910 |
| 95 | [alpha-Linolenic acid metabolism](file:///E:\01%20%E5%8D%9A%E5%A3%AB%E8%AE%BA%E6%96%87%E6%92%B0%E5%86%99%202016-2-1\3%20%E7%AC%AC%E4%B8%89%E9%83%A8%E5%88%86%20%EF%BC%881%EF%BC%89%E8%BD%AC%E5%BD%95%E7%BB%84%E5%9F%BA%E5%9B%A0%E6%B5%8B%E5%BA%8F\control_shRNA_2-VS-Magt1_shRNA_2.htm#gene95) | 1 (0.43%) | 0.4199809 | 0.8245419 | ko00592 |
| 96 | [Cell adhesion molecules (CAMs)](file:///E:\01%20%E5%8D%9A%E5%A3%AB%E8%AE%BA%E6%96%87%E6%92%B0%E5%86%99%202016-2-1\3%20%E7%AC%AC%E4%B8%89%E9%83%A8%E5%88%86%20%EF%BC%881%EF%BC%89%E8%BD%AC%E5%BD%95%E7%BB%84%E5%9F%BA%E5%9B%A0%E6%B5%8B%E5%BA%8F\control_shRNA_2-VS-Magt1_shRNA_2.htm#gene96) | 4 (1.74%) | 0.4216326 | 0.8245419 | ko04514 |
| 97 | [Melanoma](file:///E:\01%20%E5%8D%9A%E5%A3%AB%E8%AE%BA%E6%96%87%E6%92%B0%E5%86%99%202016-2-1\3%20%E7%AC%AC%E4%B8%89%E9%83%A8%E5%88%86%20%EF%BC%881%EF%BC%89%E8%BD%AC%E5%BD%95%E7%BB%84%E5%9F%BA%E5%9B%A0%E6%B5%8B%E5%BA%8F\control_shRNA_2-VS-Magt1_shRNA_2.htm#gene97) | 2 (0.87%) | 0.4270136 | 0.8245419 | ko05218 |
| 98 | [Renal cell carcinoma](file:///E:\01%20%E5%8D%9A%E5%A3%AB%E8%AE%BA%E6%96%87%E6%92%B0%E5%86%99%202016-2-1\3%20%E7%AC%AC%E4%B8%89%E9%83%A8%E5%88%86%20%EF%BC%881%EF%BC%89%E8%BD%AC%E5%BD%95%E7%BB%84%E5%9F%BA%E5%9B%A0%E6%B5%8B%E5%BA%8F\control_shRNA_2-VS-Magt1_shRNA_2.htm#gene98) | 2 (0.87%) | 0.432796 | 0.8245419 | ko05211 |
| 99 | [NF-kappa B signaling pathway](file:///E:\01%20%E5%8D%9A%E5%A3%AB%E8%AE%BA%E6%96%87%E6%92%B0%E5%86%99%202016-2-1\3%20%E7%AC%AC%E4%B8%89%E9%83%A8%E5%88%86%20%EF%BC%881%EF%BC%89%E8%BD%AC%E5%BD%95%E7%BB%84%E5%9F%BA%E5%9B%A0%E6%B5%8B%E5%BA%8F\control_shRNA_2-VS-Magt1_shRNA_2.htm#gene99) | 3 (1.3%) | 0.4390052 | 0.8245419 | ko04064 |
| 100 | [Herpes simplex infection](file:///E:\01%20%E5%8D%9A%E5%A3%AB%E8%AE%BA%E6%96%87%E6%92%B0%E5%86%99%202016-2-1\3%20%E7%AC%AC%E4%B8%89%E9%83%A8%E5%88%86%20%EF%BC%881%EF%BC%89%E8%BD%AC%E5%BD%95%E7%BB%84%E5%9F%BA%E5%9B%A0%E6%B5%8B%E5%BA%8F\control_shRNA_2-VS-Magt1_shRNA_2.htm#gene100) | 5 (2.17%) | 0.4435976 | 0.8245419 | ko05168 |
| 101 | [Natural killer cell mediated cytotoxicity](file:///E:\01%20%E5%8D%9A%E5%A3%AB%E8%AE%BA%E6%96%87%E6%92%B0%E5%86%99%202016-2-1\3%20%E7%AC%AC%E4%B8%89%E9%83%A8%E5%88%86%20%EF%BC%881%EF%BC%89%E8%BD%AC%E5%BD%95%E7%BB%84%E5%9F%BA%E5%9B%A0%E6%B5%8B%E5%BA%8F\control_shRNA_2-VS-Magt1_shRNA_2.htm#gene101) | 4 (1.74%) | 0.4479531 | 0.8245419 | ko04650 |
| 102 | [Biosynthesis of unsaturated fatty acids](file:///E:\01%20%E5%8D%9A%E5%A3%AB%E8%AE%BA%E6%96%87%E6%92%B0%E5%86%99%202016-2-1\3%20%E7%AC%AC%E4%B8%89%E9%83%A8%E5%88%86%20%EF%BC%881%EF%BC%89%E8%BD%AC%E5%BD%95%E7%BB%84%E5%9F%BA%E5%9B%A0%E6%B5%8B%E5%BA%8F\control_shRNA_2-VS-Magt1_shRNA_2.htm#gene102) | 1 (0.43%) | 0.4488926 | 0.8245419 | ko01040 |
| 103 | [Alanine, aspartate and glutamate metabolism](file:///E:\01%20%E5%8D%9A%E5%A3%AB%E8%AE%BA%E6%96%87%E6%92%B0%E5%86%99%202016-2-1\3%20%E7%AC%AC%E4%B8%89%E9%83%A8%E5%88%86%20%EF%BC%881%EF%BC%89%E8%BD%AC%E5%BD%95%E7%BB%84%E5%9F%BA%E5%9B%A0%E6%B5%8B%E5%BA%8F\control_shRNA_2-VS-Magt1_shRNA_2.htm#gene103) | 1 (0.43%) | 0.4488926 | 0.8245419 | ko00250 |
| 104 | [Asthma](file:///E:\01%20%E5%8D%9A%E5%A3%AB%E8%AE%BA%E6%96%87%E6%92%B0%E5%86%99%202016-2-1\3%20%E7%AC%AC%E4%B8%89%E9%83%A8%E5%88%86%20%EF%BC%881%EF%BC%89%E8%BD%AC%E5%BD%95%E7%BB%84%E5%9F%BA%E5%9B%A0%E6%B5%8B%E5%BA%8F\control_shRNA_2-VS-Magt1_shRNA_2.htm#gene104) | 1 (0.43%) | 0.4582073 | 0.8245419 | ko05310 |
| 105 | [T cell receptor signaling pathway](file:///E:\01%20%E5%8D%9A%E5%A3%AB%E8%AE%BA%E6%96%87%E6%92%B0%E5%86%99%202016-2-1\3%20%E7%AC%AC%E4%B8%89%E9%83%A8%E5%88%86%20%EF%BC%881%EF%BC%89%E8%BD%AC%E5%BD%95%E7%BB%84%E5%9F%BA%E5%9B%A0%E6%B5%8B%E5%BA%8F\control_shRNA_2-VS-Magt1_shRNA_2.htm#gene105) | 3 (1.3%) | 0.4610529 | 0.8245419 | ko04660 |
| 106 | [beta-Alanine metabolism](file:///E:\01%20%E5%8D%9A%E5%A3%AB%E8%AE%BA%E6%96%87%E6%92%B0%E5%86%99%202016-2-1\3%20%E7%AC%AC%E4%B8%89%E9%83%A8%E5%88%86%20%EF%BC%881%EF%BC%89%E8%BD%AC%E5%BD%95%E7%BB%84%E5%9F%BA%E5%9B%A0%E6%B5%8B%E5%BA%8F\control_shRNA_2-VS-Magt1_shRNA_2.htm#gene106) | 1 (0.43%) | 0.4763691 | 0.8245419 | ko00410 |
| 107 | [Proximal tubule bicarbonate reclamation](file:///E:\01%20%E5%8D%9A%E5%A3%AB%E8%AE%BA%E6%96%87%E6%92%B0%E5%86%99%202016-2-1\3%20%E7%AC%AC%E4%B8%89%E9%83%A8%E5%88%86%20%EF%BC%881%EF%BC%89%E8%BD%AC%E5%BD%95%E7%BB%84%E5%9F%BA%E5%9B%A0%E6%B5%8B%E5%BA%8F\control_shRNA_2-VS-Magt1_shRNA_2.htm#gene107) | 1 (0.43%) | 0.5024813 | 0.8245419 | ko04964 |
| 108 | [Glioma](file:///E:\01%20%E5%8D%9A%E5%A3%AB%E8%AE%BA%E6%96%87%E6%92%B0%E5%86%99%202016-2-1\3%20%E7%AC%AC%E4%B8%89%E9%83%A8%E5%88%86%20%EF%BC%881%EF%BC%89%E8%BD%AC%E5%BD%95%E7%BB%84%E5%9F%BA%E5%9B%A0%E6%B5%8B%E5%BA%8F\control_shRNA_2-VS-Magt1_shRNA_2.htm#gene108) | 2 (0.87%) | 0.5049435 | 0.8245419 | ko05214 |
| 109 | [Leukocyte transendothelial migration](file:///E:\01%20%E5%8D%9A%E5%A3%AB%E8%AE%BA%E6%96%87%E6%92%B0%E5%86%99%202016-2-1\3%20%E7%AC%AC%E4%B8%89%E9%83%A8%E5%88%86%20%EF%BC%881%EF%BC%89%E8%BD%AC%E5%BD%95%E7%BB%84%E5%9F%BA%E5%9B%A0%E6%B5%8B%E5%BA%8F\control_shRNA_2-VS-Magt1_shRNA_2.htm#gene109) | 4 (1.74%) | 0.5065586 | 0.8245419 | ko04670 |
| 110 | [Long-term potentiation](file:///E:\01%20%E5%8D%9A%E5%A3%AB%E8%AE%BA%E6%96%87%E6%92%B0%E5%86%99%202016-2-1\3%20%E7%AC%AC%E4%B8%89%E9%83%A8%E5%88%86%20%EF%BC%881%EF%BC%89%E8%BD%AC%E5%BD%95%E7%BB%84%E5%9F%BA%E5%9B%A0%E6%B5%8B%E5%BA%8F\control_shRNA_2-VS-Magt1_shRNA_2.htm#gene110) | 2 (0.87%) | 0.5102454 | 0.8245419 | ko04720 |
| 111 | [Pertussis](file:///E:\01%20%E5%8D%9A%E5%A3%AB%E8%AE%BA%E6%96%87%E6%92%B0%E5%86%99%202016-2-1\3%20%E7%AC%AC%E4%B8%89%E9%83%A8%E5%88%86%20%EF%BC%881%EF%BC%89%E8%BD%AC%E5%BD%95%E7%BB%84%E5%9F%BA%E5%9B%A0%E6%B5%8B%E5%BA%8F\control_shRNA_2-VS-Magt1_shRNA_2.htm#gene111) | 2 (0.87%) | 0.5155101 | 0.8245419 | ko05133 |
| 112 | [Propanoate metabolism](file:///E:\01%20%E5%8D%9A%E5%A3%AB%E8%AE%BA%E6%96%87%E6%92%B0%E5%86%99%202016-2-1\3%20%E7%AC%AC%E4%B8%89%E9%83%A8%E5%88%86%20%EF%BC%881%EF%BC%89%E8%BD%AC%E5%BD%95%E7%BB%84%E5%9F%BA%E5%9B%A0%E6%B5%8B%E5%BA%8F\control_shRNA_2-VS-Magt1_shRNA_2.htm#gene112) | 1 (0.43%) | 0.5191651 | 0.8245419 | ko00640 |
| 113 | [Protein digestion and absorption](file:///E:\01%20%E5%8D%9A%E5%A3%AB%E8%AE%BA%E6%96%87%E6%92%B0%E5%86%99%202016-2-1\3%20%E7%AC%AC%E4%B8%89%E9%83%A8%E5%88%86%20%EF%BC%881%EF%BC%89%E8%BD%AC%E5%BD%95%E7%BB%84%E5%9F%BA%E5%9B%A0%E6%B5%8B%E5%BA%8F\control_shRNA_2-VS-Magt1_shRNA_2.htm#gene113) | 2 (0.87%) | 0.5207374 | 0.8245419 | ko04974 |
| 114 | [NOD-like receptor signaling pathway](file:///E:\01%20%E5%8D%9A%E5%A3%AB%E8%AE%BA%E6%96%87%E6%92%B0%E5%86%99%202016-2-1\3%20%E7%AC%AC%E4%B8%89%E9%83%A8%E5%88%86%20%EF%BC%881%EF%BC%89%E8%BD%AC%E5%BD%95%E7%BB%84%E5%9F%BA%E5%9B%A0%E6%B5%8B%E5%BA%8F\control_shRNA_2-VS-Magt1_shRNA_2.htm#gene114) | 2 (0.87%) | 0.5259271 | 0.8245419 | ko04621 |
| 115 | [Chronic myeloid leukemia](file:///E:\01%20%E5%8D%9A%E5%A3%AB%E8%AE%BA%E6%96%87%E6%92%B0%E5%86%99%202016-2-1\3%20%E7%AC%AC%E4%B8%89%E9%83%A8%E5%88%86%20%EF%BC%881%EF%BC%89%E8%BD%AC%E5%BD%95%E7%BB%84%E5%9F%BA%E5%9B%A0%E6%B5%8B%E5%BA%8F\control_shRNA_2-VS-Magt1_shRNA_2.htm#gene115) | 2 (0.87%) | 0.5259271 | 0.8245419 | ko05220 |
| 116 | [Intestinal immune network for IgA production](file:///E:\01%20%E5%8D%9A%E5%A3%AB%E8%AE%BA%E6%96%87%E6%92%B0%E5%86%99%202016-2-1\3%20%E7%AC%AC%E4%B8%89%E9%83%A8%E5%88%86%20%EF%BC%881%EF%BC%89%E8%BD%AC%E5%BD%95%E7%BB%84%E5%9F%BA%E5%9B%A0%E6%B5%8B%E5%BA%8F\control_shRNA_2-VS-Magt1_shRNA_2.htm#gene116) | 1 (0.43%) | 0.5272968 | 0.8245419 | ko04672 |
| 117 | [Butanoate metabolism](file:///E:\01%20%E5%8D%9A%E5%A3%AB%E8%AE%BA%E6%96%87%E6%92%B0%E5%86%99%202016-2-1\3%20%E7%AC%AC%E4%B8%89%E9%83%A8%E5%88%86%20%EF%BC%881%EF%BC%89%E8%BD%AC%E5%BD%95%E7%BB%84%E5%9F%BA%E5%9B%A0%E6%B5%8B%E5%BA%8F\control_shRNA_2-VS-Magt1_shRNA_2.htm#gene117) | 1 (0.43%) | 0.5352917 | 0.8245419 | ko00650 |
| 118 | [HTLV-I infection](file:///E:\01%20%E5%8D%9A%E5%A3%AB%E8%AE%BA%E6%96%87%E6%92%B0%E5%86%99%202016-2-1\3%20%E7%AC%AC%E4%B8%89%E9%83%A8%E5%88%86%20%EF%BC%881%EF%BC%89%E8%BD%AC%E5%BD%95%E7%BB%84%E5%9F%BA%E5%9B%A0%E6%B5%8B%E5%BA%8F\control_shRNA_2-VS-Magt1_shRNA_2.htm#gene118) | 6 (2.61%) | 0.5385334 | 0.8245419 | ko05166 |
| 119 | [Proteasome](file:///E:\01%20%E5%8D%9A%E5%A3%AB%E8%AE%BA%E6%96%87%E6%92%B0%E5%86%99%202016-2-1\3%20%E7%AC%AC%E4%B8%89%E9%83%A8%E5%88%86%20%EF%BC%881%EF%BC%89%E8%BD%AC%E5%BD%95%E7%BB%84%E5%9F%BA%E5%9B%A0%E6%B5%8B%E5%BA%8F\control_shRNA_2-VS-Magt1_shRNA_2.htm#gene119) | 1 (0.43%) | 0.5431519 | 0.8245419 | ko03050 |
| 120 | [Pathogenic Escherichia coli infection](file:///E:\01%20%E5%8D%9A%E5%A3%AB%E8%AE%BA%E6%96%87%E6%92%B0%E5%86%99%202016-2-1\3%20%E7%AC%AC%E4%B8%89%E9%83%A8%E5%88%86%20%EF%BC%881%EF%BC%89%E8%BD%AC%E5%BD%95%E7%BB%84%E5%9F%BA%E5%9B%A0%E6%B5%8B%E5%BA%8F\control_shRNA_2-VS-Magt1_shRNA_2.htm#gene120) | 3 (1.3%) | 0.5450899 | 0.8245419 | ko05130 |
| 121 | [Apoptosis](file:///E:\01%20%E5%8D%9A%E5%A3%AB%E8%AE%BA%E6%96%87%E6%92%B0%E5%86%99%202016-2-1\3%20%E7%AC%AC%E4%B8%89%E9%83%A8%E5%88%86%20%EF%BC%881%EF%BC%89%E8%BD%AC%E5%BD%95%E7%BB%84%E5%9F%BA%E5%9B%A0%E6%B5%8B%E5%BA%8F\control_shRNA_2-VS-Magt1_shRNA_2.htm#gene121) | 2 (0.87%) | 0.5463054 | 0.8245419 | ko04210 |
| 122 | [Glycerophospholipid metabolism](file:///E:\01%20%E5%8D%9A%E5%A3%AB%E8%AE%BA%E6%96%87%E6%92%B0%E5%86%99%202016-2-1\3%20%E7%AC%AC%E4%B8%89%E9%83%A8%E5%88%86%20%EF%BC%881%EF%BC%89%E8%BD%AC%E5%BD%95%E7%BB%84%E5%9F%BA%E5%9B%A0%E6%B5%8B%E5%BA%8F\control_shRNA_2-VS-Magt1_shRNA_2.htm#gene122) | 2 (0.87%) | 0.5562637 | 0.8245419 | ko00564 |
| 123 | [MAPK signaling pathway](file:///E:\01%20%E5%8D%9A%E5%A3%AB%E8%AE%BA%E6%96%87%E6%92%B0%E5%86%99%202016-2-1\3%20%E7%AC%AC%E4%B8%89%E9%83%A8%E5%88%86%20%EF%BC%881%EF%BC%89%E8%BD%AC%E5%BD%95%E7%BB%84%E5%9F%BA%E5%9B%A0%E6%B5%8B%E5%BA%8F\control_shRNA_2-VS-Magt1_shRNA_2.htm#gene123) | 6 (2.61%) | 0.5580596 | 0.8245419 | ko04010 |
| 124 | [Toll-like receptor signaling pathway](file:///E:\01%20%E5%8D%9A%E5%A3%AB%E8%AE%BA%E6%96%87%E6%92%B0%E5%86%99%202016-2-1\3%20%E7%AC%AC%E4%B8%89%E9%83%A8%E5%88%86%20%EF%BC%881%EF%BC%89%E8%BD%AC%E5%BD%95%E7%BB%84%E5%9F%BA%E5%9B%A0%E6%B5%8B%E5%BA%8F\control_shRNA_2-VS-Magt1_shRNA_2.htm#gene124) | 2 (0.87%) | 0.5709099 | 0.8245419 | ko04620 |
| 125 | [ABC transporters](file:///E:\01%20%E5%8D%9A%E5%A3%AB%E8%AE%BA%E6%96%87%E6%92%B0%E5%86%99%202016-2-1\3%20%E7%AC%AC%E4%B8%89%E9%83%A8%E5%88%86%20%EF%BC%881%EF%BC%89%E8%BD%AC%E5%BD%95%E7%BB%84%E5%9F%BA%E5%9B%A0%E6%B5%8B%E5%BA%8F\control_shRNA_2-VS-Magt1_shRNA_2.htm#gene125) | 1 (0.43%) | 0.5732908 | 0.8245419 | ko02010 |
| 126 | [Metabolic pathways](file:///E:\01%20%E5%8D%9A%E5%A3%AB%E8%AE%BA%E6%96%87%E6%92%B0%E5%86%99%202016-2-1\3%20%E7%AC%AC%E4%B8%89%E9%83%A8%E5%88%86%20%EF%BC%881%EF%BC%89%E8%BD%AC%E5%BD%95%E7%BB%84%E5%9F%BA%E5%9B%A0%E6%B5%8B%E5%BA%8F\control_shRNA_2-VS-Magt1_shRNA_2.htm#gene126) | 23 (10%) | 0.5763382 | 0.8245419 | ko01100 |
| 127 | [Pyrimidine metabolism](file:///E:\01%20%E5%8D%9A%E5%A3%AB%E8%AE%BA%E6%96%87%E6%92%B0%E5%86%99%202016-2-1\3%20%E7%AC%AC%E4%B8%89%E9%83%A8%E5%88%86%20%EF%BC%881%EF%BC%89%E8%BD%AC%E5%BD%95%E7%BB%84%E5%9F%BA%E5%9B%A0%E6%B5%8B%E5%BA%8F\control_shRNA_2-VS-Magt1_shRNA_2.htm#gene127) | 2 (0.87%) | 0.5804787 | 0.8245419 | ko00240 |
| 128 | [Nucleotide excision repair](file:///E:\01%20%E5%8D%9A%E5%A3%AB%E8%AE%BA%E6%96%87%E6%92%B0%E5%86%99%202016-2-1\3%20%E7%AC%AC%E4%B8%89%E9%83%A8%E5%88%86%20%EF%BC%881%EF%BC%89%E8%BD%AC%E5%BD%95%E7%BB%84%E5%9F%BA%E5%9B%A0%E6%B5%8B%E5%BA%8F\control_shRNA_2-VS-Magt1_shRNA_2.htm#gene128) | 1 (0.43%) | 0.580511 | 0.8245419 | ko03420 |
| 129 | [Tyrosine metabolism](file:///E:\01%20%E5%8D%9A%E5%A3%AB%E8%AE%BA%E6%96%87%E6%92%B0%E5%86%99%202016-2-1\3%20%E7%AC%AC%E4%B8%89%E9%83%A8%E5%88%86%20%EF%BC%881%EF%BC%89%E8%BD%AC%E5%BD%95%E7%BB%84%E5%9F%BA%E5%9B%A0%E6%B5%8B%E5%BA%8F\control_shRNA_2-VS-Magt1_shRNA_2.htm#gene129) | 1 (0.43%) | 0.580511 | 0.8245419 | ko00350 |
| 130 | [Chemokine signaling pathway](file:///E:\01%20%E5%8D%9A%E5%A3%AB%E8%AE%BA%E6%96%87%E6%92%B0%E5%86%99%202016-2-1\3%20%E7%AC%AC%E4%B8%89%E9%83%A8%E5%88%86%20%EF%BC%881%EF%BC%89%E8%BD%AC%E5%BD%95%E7%BB%84%E5%9F%BA%E5%9B%A0%E6%B5%8B%E5%BA%8F\control_shRNA_2-VS-Magt1_shRNA_2.htm#gene130) | 4 (1.74%) | 0.5822657 | 0.8245419 | ko04062 |
| 131 | [Jak-STAT signaling pathway](file:///E:\01%20%E5%8D%9A%E5%A3%AB%E8%AE%BA%E6%96%87%E6%92%B0%E5%86%99%202016-2-1\3%20%E7%AC%AC%E4%B8%89%E9%83%A8%E5%88%86%20%EF%BC%881%EF%BC%89%E8%BD%AC%E5%BD%95%E7%BB%84%E5%9F%BA%E5%9B%A0%E6%B5%8B%E5%BA%8F\control_shRNA_2-VS-Magt1_shRNA_2.htm#gene131) | 3 (1.3%) | 0.5841686 | 0.8245419 | ko04630 |
| 132 | [Peroxisome](file:///E:\01%20%E5%8D%9A%E5%A3%AB%E8%AE%BA%E6%96%87%E6%92%B0%E5%86%99%202016-2-1\3%20%E7%AC%AC%E4%B8%89%E9%83%A8%E5%88%86%20%EF%BC%881%EF%BC%89%E8%BD%AC%E5%BD%95%E7%BB%84%E5%9F%BA%E5%9B%A0%E6%B5%8B%E5%BA%8F\control_shRNA_2-VS-Magt1_shRNA_2.htm#gene132) | 2 (0.87%) | 0.5852042 | 0.8245419 | ko04146 |
| 133 | [Fc epsilon RI signaling pathway](file:///E:\01%20%E5%8D%9A%E5%A3%AB%E8%AE%BA%E6%96%87%E6%92%B0%E5%86%99%202016-2-1\3%20%E7%AC%AC%E4%B8%89%E9%83%A8%E5%88%86%20%EF%BC%881%EF%BC%89%E8%BD%AC%E5%BD%95%E7%BB%84%E5%9F%BA%E5%9B%A0%E6%B5%8B%E5%BA%8F\control_shRNA_2-VS-Magt1_shRNA_2.htm#gene133) | 2 (0.87%) | 0.5898906 | 0.8245419 | ko04664 |
| 134 | [Hematopoietic cell lineage](file:///E:\01%20%E5%8D%9A%E5%A3%AB%E8%AE%BA%E6%96%87%E6%92%B0%E5%86%99%202016-2-1\3%20%E7%AC%AC%E4%B8%89%E9%83%A8%E5%88%86%20%EF%BC%881%EF%BC%89%E8%BD%AC%E5%BD%95%E7%BB%84%E5%9F%BA%E5%9B%A0%E6%B5%8B%E5%BA%8F\control_shRNA_2-VS-Magt1_shRNA_2.htm#gene134) | 2 (0.87%) | 0.5898906 | 0.8245419 | ko04640 |
| 135 | [Chagas disease (American trypanosomiasis)](file:///E:\01%20%E5%8D%9A%E5%A3%AB%E8%AE%BA%E6%96%87%E6%92%B0%E5%86%99%202016-2-1\3%20%E7%AC%AC%E4%B8%89%E9%83%A8%E5%88%86%20%EF%BC%881%EF%BC%89%E8%BD%AC%E5%BD%95%E7%BB%84%E5%9F%BA%E5%9B%A0%E6%B5%8B%E5%BA%8F\control_shRNA_2-VS-Magt1_shRNA_2.htm#gene135) | 2 (0.87%) | 0.5945376 | 0.8245419 | ko05142 |
| 136 | [Spliceosome](file:///E:\01%20%E5%8D%9A%E5%A3%AB%E8%AE%BA%E6%96%87%E6%92%B0%E5%86%99%202016-2-1\3%20%E7%AC%AC%E4%B8%89%E9%83%A8%E5%88%86%20%EF%BC%881%EF%BC%89%E8%BD%AC%E5%BD%95%E7%BB%84%E5%9F%BA%E5%9B%A0%E6%B5%8B%E5%BA%8F\control_shRNA_2-VS-Magt1_shRNA_2.htm#gene136) | 3 (1.3%) | 0.5991941 | 0.8245419 | ko03040 |
| 137 | [Oxidative phosphorylation](file:///E:\01%20%E5%8D%9A%E5%A3%AB%E8%AE%BA%E6%96%87%E6%92%B0%E5%86%99%202016-2-1\3%20%E7%AC%AC%E4%B8%89%E9%83%A8%E5%88%86%20%EF%BC%881%EF%BC%89%E8%BD%AC%E5%BD%95%E7%BB%84%E5%9F%BA%E5%9B%A0%E6%B5%8B%E5%BA%8F\control_shRNA_2-VS-Magt1_shRNA_2.htm#gene137) | 2 (0.87%) | 0.6082431 | 0.8245419 | ko00190 |
| 138 | [VEGF signaling pathway](file:///E:\01%20%E5%8D%9A%E5%A3%AB%E8%AE%BA%E6%96%87%E6%92%B0%E5%86%99%202016-2-1\3%20%E7%AC%AC%E4%B8%89%E9%83%A8%E5%88%86%20%EF%BC%881%EF%BC%89%E8%BD%AC%E5%BD%95%E7%BB%84%E5%9F%BA%E5%9B%A0%E6%B5%8B%E5%BA%8F\control_shRNA_2-VS-Magt1_shRNA_2.htm#gene138) | 2 (0.87%) | 0.6082431 | 0.8245419 | ko04370 |
| 139 | [Tight junction](file:///E:\01%20%E5%8D%9A%E5%A3%AB%E8%AE%BA%E6%96%87%E6%92%B0%E5%86%99%202016-2-1\3%20%E7%AC%AC%E4%B8%89%E9%83%A8%E5%88%86%20%EF%BC%881%EF%BC%89%E8%BD%AC%E5%BD%95%E7%BB%84%E5%9F%BA%E5%9B%A0%E6%B5%8B%E5%BA%8F\control_shRNA_2-VS-Magt1_shRNA_2.htm#gene139) | 5 (2.17%) | 0.6165098 | 0.8245419 | ko04530 |
| 140 | [Melanogenesis](file:///E:\01%20%E5%8D%9A%E5%A3%AB%E8%AE%BA%E6%96%87%E6%92%B0%E5%86%99%202016-2-1\3%20%E7%AC%AC%E4%B8%89%E9%83%A8%E5%88%86%20%EF%BC%881%EF%BC%89%E8%BD%AC%E5%BD%95%E7%BB%84%E5%9F%BA%E5%9B%A0%E6%B5%8B%E5%BA%8F\control_shRNA_2-VS-Magt1_shRNA_2.htm#gene140) | 2 (0.87%) | 0.6171837 | 0.8245419 | ko04916 |
| 141 | [Axon guidance](file:///E:\01%20%E5%8D%9A%E5%A3%AB%E8%AE%BA%E6%96%87%E6%92%B0%E5%86%99%202016-2-1\3%20%E7%AC%AC%E4%B8%89%E9%83%A8%E5%88%86%20%EF%BC%881%EF%BC%89%E8%BD%AC%E5%BD%95%E7%BB%84%E5%9F%BA%E5%9B%A0%E6%B5%8B%E5%BA%8F\control_shRNA_2-VS-Magt1_shRNA_2.htm#gene141) | 4 (1.74%) | 0.620651 | 0.8245419 | ko04360 |
| 142 | [Cytosolic DNA-sensing pathway](file:///E:\01%20%E5%8D%9A%E5%A3%AB%E8%AE%BA%E6%96%87%E6%92%B0%E5%86%99%202016-2-1\3%20%E7%AC%AC%E4%B8%89%E9%83%A8%E5%88%86%20%EF%BC%881%EF%BC%89%E8%BD%AC%E5%BD%95%E7%BB%84%E5%9F%BA%E5%9B%A0%E6%B5%8B%E5%BA%8F\control_shRNA_2-VS-Magt1_shRNA_2.htm#gene142) | 1 (0.43%) | 0.6213474 | 0.8245419 | ko04623 |
| 143 | [Bile secretion](file:///E:\01%20%E5%8D%9A%E5%A3%AB%E8%AE%BA%E6%96%87%E6%92%B0%E5%86%99%202016-2-1\3%20%E7%AC%AC%E4%B8%89%E9%83%A8%E5%88%86%20%EF%BC%881%EF%BC%89%E8%BD%AC%E5%BD%95%E7%BB%84%E5%9F%BA%E5%9B%A0%E6%B5%8B%E5%BA%8F\control_shRNA_2-VS-Magt1_shRNA_2.htm#gene143) | 2 (0.87%) | 0.6215952 | 0.8245419 | ko04976 |
| 144 | [Sphingolipid metabolism](file:///E:\01%20%E5%8D%9A%E5%A3%AB%E8%AE%BA%E6%96%87%E6%92%B0%E5%86%99%202016-2-1\3%20%E7%AC%AC%E4%B8%89%E9%83%A8%E5%88%86%20%EF%BC%881%EF%BC%89%E8%BD%AC%E5%BD%95%E7%BB%84%E5%9F%BA%E5%9B%A0%E6%B5%8B%E5%BA%8F\control_shRNA_2-VS-Magt1_shRNA_2.htm#gene144) | 1 (0.43%) | 0.6277577 | 0.8245419 | ko00600 |
| 145 | [Tuberculosis](file:///E:\01%20%E5%8D%9A%E5%A3%AB%E8%AE%BA%E6%96%87%E6%92%B0%E5%86%99%202016-2-1\3%20%E7%AC%AC%E4%B8%89%E9%83%A8%E5%88%86%20%EF%BC%881%EF%BC%89%E8%BD%AC%E5%BD%95%E7%BB%84%E5%9F%BA%E5%9B%A0%E6%B5%8B%E5%BA%8F\control_shRNA_2-VS-Magt1_shRNA_2.htm#gene145) | 4 (1.74%) | 0.6298968 | 0.8245419 | ko05152 |
| 146 | [Ether lipid metabolism](file:///E:\01%20%E5%8D%9A%E5%A3%AB%E8%AE%BA%E6%96%87%E6%92%B0%E5%86%99%202016-2-1\3%20%E7%AC%AC%E4%B8%89%E9%83%A8%E5%88%86%20%EF%BC%881%EF%BC%89%E8%BD%AC%E5%BD%95%E7%BB%84%E5%9F%BA%E5%9B%A0%E6%B5%8B%E5%BA%8F\control_shRNA_2-VS-Magt1_shRNA_2.htm#gene146) | 1 (0.43%) | 0.6340599 | 0.8245419 | ko00565 |
| 147 | [B cell receptor signaling pathway](file:///E:\01%20%E5%8D%9A%E5%A3%AB%E8%AE%BA%E6%96%87%E6%92%B0%E5%86%99%202016-2-1\3%20%E7%AC%AC%E4%B8%89%E9%83%A8%E5%88%86%20%EF%BC%881%EF%BC%89%E8%BD%AC%E5%BD%95%E7%BB%84%E5%9F%BA%E5%9B%A0%E6%B5%8B%E5%BA%8F\control_shRNA_2-VS-Magt1_shRNA_2.htm#gene147) | 2 (0.87%) | 0.6345951 | 0.8245419 | ko04662 |
| 148 | [Phosphatidylinositol signaling system](file:///E:\01%20%E5%8D%9A%E5%A3%AB%E8%AE%BA%E6%96%87%E6%92%B0%E5%86%99%202016-2-1\3%20%E7%AC%AC%E4%B8%89%E9%83%A8%E5%88%86%20%EF%BC%881%EF%BC%89%E8%BD%AC%E5%BD%95%E7%BB%84%E5%9F%BA%E5%9B%A0%E6%B5%8B%E5%BA%8F\control_shRNA_2-VS-Magt1_shRNA_2.htm#gene148) | 2 (0.87%) | 0.643067 | 0.8299040 | ko04070 |
| 149 | [Fatty acid metabolism](file:///E:\01%20%E5%8D%9A%E5%A3%AB%E8%AE%BA%E6%96%87%E6%92%B0%E5%86%99%202016-2-1\3%20%E7%AC%AC%E4%B8%89%E9%83%A8%E5%88%86%20%EF%BC%881%EF%BC%89%E8%BD%AC%E5%BD%95%E7%BB%84%E5%9F%BA%E5%9B%A0%E6%B5%8B%E5%BA%8F\control_shRNA_2-VS-Magt1_shRNA_2.htm#gene149) | 1 (0.43%) | 0.6523362 | 0.8362162 | ko00071 |
| 150 | [Aminoacyl-tRNA biosynthesis](file:///E:\01%20%E5%8D%9A%E5%A3%AB%E8%AE%BA%E6%96%87%E6%92%B0%E5%86%99%202016-2-1\3%20%E7%AC%AC%E4%B8%89%E9%83%A8%E5%88%86%20%EF%BC%881%EF%BC%89%E8%BD%AC%E5%BD%95%E7%BB%84%E5%9F%BA%E5%9B%A0%E6%B5%8B%E5%BA%8F\control_shRNA_2-VS-Magt1_shRNA_2.htm#gene150) | 1 (0.43%) | 0.658224 | 0.8381386 | ko00970 |
| 151 | [Adherens junction](file:///E:\01%20%E5%8D%9A%E5%A3%AB%E8%AE%BA%E6%96%87%E6%92%B0%E5%86%99%202016-2-1\3%20%E7%AC%AC%E4%B8%89%E9%83%A8%E5%88%86%20%EF%BC%881%EF%BC%89%E8%BD%AC%E5%BD%95%E7%BB%84%E5%9F%BA%E5%9B%A0%E6%B5%8B%E5%BA%8F\control_shRNA_2-VS-Magt1_shRNA_2.htm#gene151) | 2 (0.87%) | 0.6715036 | 0.8389201 | ko04520 |
| 152 | [Huntington's disease](file:///E:\01%20%E5%8D%9A%E5%A3%AB%E8%AE%BA%E6%96%87%E6%92%B0%E5%86%99%202016-2-1\3%20%E7%AC%AC%E4%B8%89%E9%83%A8%E5%88%86%20%EF%BC%881%EF%BC%89%E8%BD%AC%E5%BD%95%E7%BB%84%E5%9F%BA%E5%9B%A0%E6%B5%8B%E5%BA%8F\control_shRNA_2-VS-Magt1_shRNA_2.htm#gene152) | 3 (1.3%) | 0.6721159 | 0.8389201 | ko05016 |
| 153 | [Carbohydrate digestion and absorption](file:///E:\01%20%E5%8D%9A%E5%A3%AB%E8%AE%BA%E6%96%87%E6%92%B0%E5%86%99%202016-2-1\3%20%E7%AC%AC%E4%B8%89%E9%83%A8%E5%88%86%20%EF%BC%881%EF%BC%89%E8%BD%AC%E5%BD%95%E7%BB%84%E5%9F%BA%E5%9B%A0%E6%B5%8B%E5%BA%8F\control_shRNA_2-VS-Magt1_shRNA_2.htm#gene153) | 1 (0.43%) | 0.6752985 | 0.8389201 | ko04973 |
| 154 | [Amino sugar and nucleotide sugar metabolism](file:///E:\01%20%E5%8D%9A%E5%A3%AB%E8%AE%BA%E6%96%87%E6%92%B0%E5%86%99%202016-2-1\3%20%E7%AC%AC%E4%B8%89%E9%83%A8%E5%88%86%20%EF%BC%881%EF%BC%89%E8%BD%AC%E5%BD%95%E7%BB%84%E5%9F%BA%E5%9B%A0%E6%B5%8B%E5%BA%8F\control_shRNA_2-VS-Magt1_shRNA_2.htm#gene154) | 1 (0.43%) | 0.680799 | 0.8389201 | ko00520 |
| 155 | [Valine, leucine and isoleucine degradation](file:///E:\01%20%E5%8D%9A%E5%A3%AB%E8%AE%BA%E6%96%87%E6%92%B0%E5%86%99%202016-2-1\3%20%E7%AC%AC%E4%B8%89%E9%83%A8%E5%88%86%20%EF%BC%881%EF%BC%89%E8%BD%AC%E5%BD%95%E7%BB%84%E5%9F%BA%E5%9B%A0%E6%B5%8B%E5%BA%8F\control_shRNA_2-VS-Magt1_shRNA_2.htm#gene155) | 1 (0.43%) | 0.680799 | 0.8389201 | ko00280 |
| 156 | [RIG-I-like receptor signaling pathway](file:///E:\01%20%E5%8D%9A%E5%A3%AB%E8%AE%BA%E6%96%87%E6%92%B0%E5%86%99%202016-2-1\3%20%E7%AC%AC%E4%B8%89%E9%83%A8%E5%88%86%20%EF%BC%881%EF%BC%89%E8%BD%AC%E5%BD%95%E7%BB%84%E5%9F%BA%E5%9B%A0%E6%B5%8B%E5%BA%8F\control_shRNA_2-VS-Magt1_shRNA_2.htm#gene156) | 1 (0.43%) | 0.6862068 | 0.8401635 | ko04622 |
| 157 | [Mineral absorption](file:///E:\01%20%E5%8D%9A%E5%A3%AB%E8%AE%BA%E6%96%87%E6%92%B0%E5%86%99%202016-2-1\3%20%E7%AC%AC%E4%B8%89%E9%83%A8%E5%88%86%20%EF%BC%881%EF%BC%89%E8%BD%AC%E5%BD%95%E7%BB%84%E5%9F%BA%E5%9B%A0%E6%B5%8B%E5%BA%8F\control_shRNA_2-VS-Magt1_shRNA_2.htm#gene157) | 1 (0.43%) | 0.701889 | 0.8463432 | ko04978 |
| 158 | [Lysine degradation](file:///E:\01%20%E5%8D%9A%E5%A3%AB%E8%AE%BA%E6%96%87%E6%92%B0%E5%86%99%202016-2-1\3%20%E7%AC%AC%E4%B8%89%E9%83%A8%E5%88%86%20%EF%BC%881%EF%BC%89%E8%BD%AC%E5%BD%95%E7%BB%84%E5%9F%BA%E5%9B%A0%E6%B5%8B%E5%BA%8F\control_shRNA_2-VS-Magt1_shRNA_2.htm#gene158) | 1 (0.43%) | 0.706941 | 0.8463432 | ko00310 |
| 159 | [Endometrial cancer](file:///E:\01%20%E5%8D%9A%E5%A3%AB%E8%AE%BA%E6%96%87%E6%92%B0%E5%86%99%202016-2-1\3%20%E7%AC%AC%E4%B8%89%E9%83%A8%E5%88%86%20%EF%BC%881%EF%BC%89%E8%BD%AC%E5%BD%95%E7%BB%84%E5%9F%BA%E5%9B%A0%E6%B5%8B%E5%BA%8F\control_shRNA_2-VS-Magt1_shRNA_2.htm#gene159) | 1 (0.43%) | 0.7167906 | 0.8463432 | ko05213 |
| 160 | [Cell cycle](file:///E:\01%20%E5%8D%9A%E5%A3%AB%E8%AE%BA%E6%96%87%E6%92%B0%E5%86%99%202016-2-1\3%20%E7%AC%AC%E4%B8%89%E9%83%A8%E5%88%86%20%EF%BC%881%EF%BC%89%E8%BD%AC%E5%BD%95%E7%BB%84%E5%9F%BA%E5%9B%A0%E6%B5%8B%E5%BA%8F\control_shRNA_2-VS-Magt1_shRNA_2.htm#gene160) | 2 (0.87%) | 0.7194347 | 0.8463432 | ko04110 |
| 161 | [Legionellosis](file:///E:\01%20%E5%8D%9A%E5%A3%AB%E8%AE%BA%E6%96%87%E6%92%B0%E5%86%99%202016-2-1\3%20%E7%AC%AC%E4%B8%89%E9%83%A8%E5%88%86%20%EF%BC%881%EF%BC%89%E8%BD%AC%E5%BD%95%E7%BB%84%E5%9F%BA%E5%9B%A0%E6%B5%8B%E5%BA%8F\control_shRNA_2-VS-Magt1_shRNA_2.htm#gene161) | 1 (0.43%) | 0.7215911 | 0.8463432 | ko05134 |
| 162 | [Steroid hormone biosynthesis](file:///E:\01%20%E5%8D%9A%E5%A3%AB%E8%AE%BA%E6%96%87%E6%92%B0%E5%86%99%202016-2-1\3%20%E7%AC%AC%E4%B8%89%E9%83%A8%E5%88%86%20%EF%BC%881%EF%BC%89%E8%BD%AC%E5%BD%95%E7%BB%84%E5%9F%BA%E5%9B%A0%E6%B5%8B%E5%BA%8F\control_shRNA_2-VS-Magt1_shRNA_2.htm#gene162) | 1 (0.43%) | 0.7215911 | 0.8463432 | ko00140 |
| 163 | [mTOR signaling pathway](file:///E:\01%20%E5%8D%9A%E5%A3%AB%E8%AE%BA%E6%96%87%E6%92%B0%E5%86%99%202016-2-1\3%20%E7%AC%AC%E4%B8%89%E9%83%A8%E5%88%86%20%EF%BC%881%EF%BC%89%E8%BD%AC%E5%BD%95%E7%BB%84%E5%9F%BA%E5%9B%A0%E6%B5%8B%E5%BA%8F\control_shRNA_2-VS-Magt1_shRNA_2.htm#gene163) | 1 (0.43%) | 0.7309504 | 0.8463432 | ko04150 |
| 164 | [Prion diseases](file:///E:\01%20%E5%8D%9A%E5%A3%AB%E8%AE%BA%E6%96%87%E6%92%B0%E5%86%99%202016-2-1\3%20%E7%AC%AC%E4%B8%89%E9%83%A8%E5%88%86%20%EF%BC%881%EF%BC%89%E8%BD%AC%E5%BD%95%E7%BB%84%E5%9F%BA%E5%9B%A0%E6%B5%8B%E5%BA%8F\control_shRNA_2-VS-Magt1_shRNA_2.htm#gene164) | 1 (0.43%) | 0.7309504 | 0.8463432 | ko05020 |
| 165 | [Non-small cell lung cancer](file:///E:\01%20%E5%8D%9A%E5%A3%AB%E8%AE%BA%E6%96%87%E6%92%B0%E5%86%99%202016-2-1\3%20%E7%AC%AC%E4%B8%89%E9%83%A8%E5%88%86%20%EF%BC%881%EF%BC%89%E8%BD%AC%E5%BD%95%E7%BB%84%E5%9F%BA%E5%9B%A0%E6%B5%8B%E5%BA%8F\control_shRNA_2-VS-Magt1_shRNA_2.htm#gene165) | 1 (0.43%) | 0.7355119 | 0.8463432 | ko05223 |
| 166 | [Colorectal cancer](file:///E:\01%20%E5%8D%9A%E5%A3%AB%E8%AE%BA%E6%96%87%E6%92%B0%E5%86%99%202016-2-1\3%20%E7%AC%AC%E4%B8%89%E9%83%A8%E5%88%86%20%EF%BC%881%EF%BC%89%E8%BD%AC%E5%BD%95%E7%BB%84%E5%9F%BA%E5%9B%A0%E6%B5%8B%E5%BA%8F\control_shRNA_2-VS-Magt1_shRNA_2.htm#gene166) | 1 (0.43%) | 0.7399964 | 0.8463432 | ko05210 |
| 167 | [Glycerolipid metabolism](file:///E:\01%20%E5%8D%9A%E5%A3%AB%E8%AE%BA%E6%96%87%E6%92%B0%E5%86%99%202016-2-1\3%20%E7%AC%AC%E4%B8%89%E9%83%A8%E5%88%86%20%EF%BC%881%EF%BC%89%E8%BD%AC%E5%BD%95%E7%BB%84%E5%9F%BA%E5%9B%A0%E6%B5%8B%E5%BA%8F\control_shRNA_2-VS-Magt1_shRNA_2.htm#gene167) | 1 (0.43%) | 0.7399964 | 0.8463432 | ko00561 |
| 168 | [Hepatitis C](file:///E:\01%20%E5%8D%9A%E5%A3%AB%E8%AE%BA%E6%96%87%E6%92%B0%E5%86%99%202016-2-1\3%20%E7%AC%AC%E4%B8%89%E9%83%A8%E5%88%86%20%EF%BC%881%EF%BC%89%E8%BD%AC%E5%BD%95%E7%BB%84%E5%9F%BA%E5%9B%A0%E6%B5%8B%E5%BA%8F\control_shRNA_2-VS-Magt1_shRNA_2.htm#gene168) | 2 (0.87%) | 0.7673115 | 0.8667519 | ko05160 |
| 169 | [Fc gamma R-mediated phagocytosis](file:///E:\01%20%E5%8D%9A%E5%A3%AB%E8%AE%BA%E6%96%87%E6%92%B0%E5%86%99%202016-2-1\3%20%E7%AC%AC%E4%B8%89%E9%83%A8%E5%88%86%20%EF%BC%881%EF%BC%89%E8%BD%AC%E5%BD%95%E7%BB%84%E5%9F%BA%E5%9B%A0%E6%B5%8B%E5%BA%8F\control_shRNA_2-VS-Magt1_shRNA_2.htm#gene169) | 2 (0.87%) | 0.7731306 | 0.8667519 | ko04666 |
| 170 | [Epithelial cell signaling in Helicobacter pylori infection](file:///E:\01%20%E5%8D%9A%E5%A3%AB%E8%AE%BA%E6%96%87%E6%92%B0%E5%86%99%202016-2-1\3%20%E7%AC%AC%E4%B8%89%E9%83%A8%E5%88%86%20%EF%BC%881%EF%BC%89%E8%BD%AC%E5%BD%95%E7%BB%84%E5%9F%BA%E5%9B%A0%E6%B5%8B%E5%BA%8F\control_shRNA_2-VS-Magt1_shRNA_2.htm#gene170) | 1 (0.43%) | 0.7732511 | 0.8667519 | ko05120 |
| 171 | [Cardiac muscle contraction](file:///E:\01%20%E5%8D%9A%E5%A3%AB%E8%AE%BA%E6%96%87%E6%92%B0%E5%86%99%202016-2-1\3%20%E7%AC%AC%E4%B8%89%E9%83%A8%E5%88%86%20%EF%BC%881%EF%BC%89%E8%BD%AC%E5%BD%95%E7%BB%84%E5%9F%BA%E5%9B%A0%E6%B5%8B%E5%BA%8F\control_shRNA_2-VS-Magt1_shRNA_2.htm#gene171) | 2 (0.87%) | 0.7759925 | 0.8667519 | ko04260 |
| 172 | [Ribosome biogenesis in eukaryotes](file:///E:\01%20%E5%8D%9A%E5%A3%AB%E8%AE%BA%E6%96%87%E6%92%B0%E5%86%99%202016-2-1\3%20%E7%AC%AC%E4%B8%89%E9%83%A8%E5%88%86%20%EF%BC%881%EF%BC%89%E8%BD%AC%E5%BD%95%E7%BB%84%E5%9F%BA%E5%9B%A0%E6%B5%8B%E5%BA%8F\control_shRNA_2-VS-Magt1_shRNA_2.htm#gene172) | 1 (0.43%) | 0.7882542 | 0.8680043 | ko03008 |
| 173 | [RNA degradation](file:///E:\01%20%E5%8D%9A%E5%A3%AB%E8%AE%BA%E6%96%87%E6%92%B0%E5%86%99%202016-2-1\3%20%E7%AC%AC%E4%B8%89%E9%83%A8%E5%88%86%20%EF%BC%881%EF%BC%89%E8%BD%AC%E5%BD%95%E7%BB%84%E5%9F%BA%E5%9B%A0%E6%B5%8B%E5%BA%8F\control_shRNA_2-VS-Magt1_shRNA_2.htm#gene173) | 1 (0.43%) | 0.7953808 | 0.8680043 | ko03018 |
| 174 | [Osteoclast differentiation](file:///E:\01%20%E5%8D%9A%E5%A3%AB%E8%AE%BA%E6%96%87%E6%92%B0%E5%86%99%202016-2-1\3%20%E7%AC%AC%E4%B8%89%E9%83%A8%E5%88%86%20%EF%BC%881%EF%BC%89%E8%BD%AC%E5%BD%95%E7%BB%84%E5%9F%BA%E5%9B%A0%E6%B5%8B%E5%BA%8F\control_shRNA_2-VS-Magt1_shRNA_2.htm#gene174) | 2 (0.87%) | 0.7977752 | 0.8680043 | ko04380 |
| 175 | [Rheumatoid arthritis](file:///E:\01%20%E5%8D%9A%E5%A3%AB%E8%AE%BA%E6%96%87%E6%92%B0%E5%86%99%202016-2-1\3%20%E7%AC%AC%E4%B8%89%E9%83%A8%E5%88%86%20%EF%BC%881%EF%BC%89%E8%BD%AC%E5%BD%95%E7%BB%84%E5%9F%BA%E5%9B%A0%E6%B5%8B%E5%BA%8F\control_shRNA_2-VS-Magt1_shRNA_2.htm#gene175) | 1 (0.43%) | 0.8022686 | 0.8680043 | ko05323 |
| 176 | [Staphylococcus aureus infection](file:///E:\01%20%E5%8D%9A%E5%A3%AB%E8%AE%BA%E6%96%87%E6%92%B0%E5%86%99%202016-2-1\3%20%E7%AC%AC%E4%B8%89%E9%83%A8%E5%88%86%20%EF%BC%881%EF%BC%89%E8%BD%AC%E5%BD%95%E7%BB%84%E5%9F%BA%E5%9B%A0%E6%B5%8B%E5%BA%8F\control_shRNA_2-VS-Magt1_shRNA_2.htm#gene176) | 1 (0.43%) | 0.8022686 | 0.8680043 | ko05150 |
| 177 | [Leishmaniasis](file:///E:\01%20%E5%8D%9A%E5%A3%AB%E8%AE%BA%E6%96%87%E6%92%B0%E5%86%99%202016-2-1\3%20%E7%AC%AC%E4%B8%89%E9%83%A8%E5%88%86%20%EF%BC%881%EF%BC%89%E8%BD%AC%E5%BD%95%E7%BB%84%E5%9F%BA%E5%9B%A0%E6%B5%8B%E5%BA%8F\control_shRNA_2-VS-Magt1_shRNA_2.htm#gene177) | 1 (0.43%) | 0.8089255 | 0.8680043 | ko05140 |
| 178 | [Pancreatic cancer](file:///E:\01%20%E5%8D%9A%E5%A3%AB%E8%AE%BA%E6%96%87%E6%92%B0%E5%86%99%202016-2-1\3%20%E7%AC%AC%E4%B8%89%E9%83%A8%E5%88%86%20%EF%BC%881%EF%BC%89%E8%BD%AC%E5%BD%95%E7%BB%84%E5%9F%BA%E5%9B%A0%E6%B5%8B%E5%BA%8F\control_shRNA_2-VS-Magt1_shRNA_2.htm#gene178) | 1 (0.43%) | 0.8089255 | 0.8680043 | ko05212 |
| 179 | [Ubiquitin mediated proteolysis](file:///E:\01%20%E5%8D%9A%E5%A3%AB%E8%AE%BA%E6%96%87%E6%92%B0%E5%86%99%202016-2-1\3%20%E7%AC%AC%E4%B8%89%E9%83%A8%E5%88%86%20%EF%BC%881%EF%BC%89%E8%BD%AC%E5%BD%95%E7%BB%84%E5%9F%BA%E5%9B%A0%E6%B5%8B%E5%BA%8F\control_shRNA_2-VS-Magt1_shRNA_2.htm#gene179) | 2 (0.87%) | 0.8246676 | 0.8764124 | ko04120 |
| 180 | [Cytokine-cytokine receptor interaction](file:///E:\01%20%E5%8D%9A%E5%A3%AB%E8%AE%BA%E6%96%87%E6%92%B0%E5%86%99%202016-2-1\3%20%E7%AC%AC%E4%B8%89%E9%83%A8%E5%88%86%20%EF%BC%881%EF%BC%89%E8%BD%AC%E5%BD%95%E7%BB%84%E5%9F%BA%E5%9B%A0%E6%B5%8B%E5%BA%8F\control_shRNA_2-VS-Magt1_shRNA_2.htm#gene180) | 3 (1.3%) | 0.8259384 | 0.8764124 | ko04060 |
| 181 | [p53 signaling pathway](file:///E:\01%20%E5%8D%9A%E5%A3%AB%E8%AE%BA%E6%96%87%E6%92%B0%E5%86%99%202016-2-1\3%20%E7%AC%AC%E4%B8%89%E9%83%A8%E5%88%86%20%EF%BC%881%EF%BC%89%E8%BD%AC%E5%BD%95%E7%BB%84%E5%9F%BA%E5%9B%A0%E6%B5%8B%E5%BA%8F\control_shRNA_2-VS-Magt1_shRNA_2.htm#gene181) | 1 (0.43%) | 0.8522303 | 0.8943736 | ko04115 |
| 182 | [GABAergic synapse](file:///E:\01%20%E5%8D%9A%E5%A3%AB%E8%AE%BA%E6%96%87%E6%92%B0%E5%86%99%202016-2-1\3%20%E7%AC%AC%E4%B8%89%E9%83%A8%E5%88%86%20%EF%BC%881%EF%BC%89%E8%BD%AC%E5%BD%95%E7%BB%84%E5%9F%BA%E5%9B%A0%E6%B5%8B%E5%BA%8F\control_shRNA_2-VS-Magt1_shRNA_2.htm#gene182) | 1 (0.43%) | 0.8522303 | 0.8943736 | ko04727 |
| 183 | [RNA transport](file:///E:\01%20%E5%8D%9A%E5%A3%AB%E8%AE%BA%E6%96%87%E6%92%B0%E5%86%99%202016-2-1\3%20%E7%AC%AC%E4%B8%89%E9%83%A8%E5%88%86%20%EF%BC%881%EF%BC%89%E8%BD%AC%E5%BD%95%E7%BB%84%E5%9F%BA%E5%9B%A0%E6%B5%8B%E5%BA%8F\control_shRNA_2-VS-Magt1_shRNA_2.htm#gene183) | 2 (0.87%) | 0.8672654 | 0.9051786 | ko03013 |
| 184 | [ErbB signaling pathway](file:///E:\01%20%E5%8D%9A%E5%A3%AB%E8%AE%BA%E6%96%87%E6%92%B0%E5%86%99%202016-2-1\3%20%E7%AC%AC%E4%B8%89%E9%83%A8%E5%88%86%20%EF%BC%881%EF%BC%89%E8%BD%AC%E5%BD%95%E7%BB%84%E5%9F%BA%E5%9B%A0%E6%B5%8B%E5%BA%8F\control_shRNA_2-VS-Magt1_shRNA_2.htm#gene184) | 1 (0.43%) | 0.8817642 | 0.9153096 | ko04012 |
| 185 | [Complement and coagulation cascades](file:///E:\01%20%E5%8D%9A%E5%A3%AB%E8%AE%BA%E6%96%87%E6%92%B0%E5%86%99%202016-2-1\3%20%E7%AC%AC%E4%B8%89%E9%83%A8%E5%88%86%20%EF%BC%881%EF%BC%89%E8%BD%AC%E5%BD%95%E7%BB%84%E5%9F%BA%E5%9B%A0%E6%B5%8B%E5%BA%8F\control_shRNA_2-VS-Magt1_shRNA_2.htm#gene185) | 1 (0.43%) | 0.896935 | 0.9260248 | ko04610 |
| 186 | [Pancreatic secretion](file:///E:\01%20%E5%8D%9A%E5%A3%AB%E8%AE%BA%E6%96%87%E6%92%B0%E5%86%99%202016-2-1\3%20%E7%AC%AC%E4%B8%89%E9%83%A8%E5%88%86%20%EF%BC%881%EF%BC%89%E8%BD%AC%E5%BD%95%E7%BB%84%E5%9F%BA%E5%9B%A0%E6%B5%8B%E5%BA%8F\control_shRNA_2-VS-Magt1_shRNA_2.htm#gene186) | 1 (0.43%) | 0.9217061 | 0.9464831 | ko04972 |
| 187 | [Serotonergic synapse](file:///E:\01%20%E5%8D%9A%E5%A3%AB%E8%AE%BA%E6%96%87%E6%92%B0%E5%86%99%202016-2-1\3%20%E7%AC%AC%E4%B8%89%E9%83%A8%E5%88%86%20%EF%BC%881%EF%BC%89%E8%BD%AC%E5%BD%95%E7%BB%84%E5%9F%BA%E5%9B%A0%E6%B5%8B%E5%BA%8F\control_shRNA_2-VS-Magt1_shRNA_2.htm#gene187) | 1 (0.43%) | 0.938461 | 0.9585350 | ko04726 |
| 188 | [Lysosome](file:///E:\01%20%E5%8D%9A%E5%A3%AB%E8%AE%BA%E6%96%87%E6%92%B0%E5%86%99%202016-2-1\3%20%E7%AC%AC%E4%B8%89%E9%83%A8%E5%88%86%20%EF%BC%881%EF%BC%89%E8%BD%AC%E5%BD%95%E7%BB%84%E5%9F%BA%E5%9B%A0%E6%B5%8B%E5%BA%8F\control_shRNA_2-VS-Magt1_shRNA_2.htm#gene188) | 1 (0.43%) | 0.9532804 | 0.9684923 | ko04142 |
| 189 | [Wnt signaling pathway](file:///E:\01%20%E5%8D%9A%E5%A3%AB%E8%AE%BA%E6%96%87%E6%92%B0%E5%86%99%202016-2-1\3%20%E7%AC%AC%E4%B8%89%E9%83%A8%E5%88%86%20%EF%BC%881%EF%BC%89%E8%BD%AC%E5%BD%95%E7%BB%84%E5%9F%BA%E5%9B%A0%E6%B5%8B%E5%BA%8F\control_shRNA_2-VS-Magt1_shRNA_2.htm#gene189) | 1 (0.43%) | 0.9706734 | 0.9762868 | ko04310 |
| 190 | [Protein processing in endoplasmic reticulum](file:///E:\01%20%E5%8D%9A%E5%A3%AB%E8%AE%BA%E6%96%87%E6%92%B0%E5%86%99%202016-2-1\3%20%E7%AC%AC%E4%B8%89%E9%83%A8%E5%88%86%20%EF%BC%881%EF%BC%89%E8%BD%AC%E5%BD%95%E7%BB%84%E5%9F%BA%E5%9B%A0%E6%B5%8B%E5%BA%8F\control_shRNA_2-VS-Magt1_shRNA_2.htm#gene190) | 1 (0.43%) | 0.9711754 | 0.9762868 | ko04141 |
| 191 | [Olfactory transduction](file:///E:\01%20%E5%8D%9A%E5%A3%AB%E8%AE%BA%E6%96%87%E6%92%B0%E5%86%99%202016-2-1\3%20%E7%AC%AC%E4%B8%89%E9%83%A8%E5%88%86%20%EF%BC%881%EF%BC%89%E8%BD%AC%E5%BD%95%E7%BB%84%E5%9F%BA%E5%9B%A0%E6%B5%8B%E5%BA%8F\control_shRNA_2-VS-Magt1_shRNA_2.htm#gene191) | 2 (0.87%) | 1 | 1.0000000 | ko04740 |
