# Supplementary material for: MagT1 regulated the odontogenic differentiation of BMMSCs induced byTGC-CM via ERK signaling pathway
Source: Stem Cell Res Ther. 2019 Jan 31;10:48. doi: 10.1186/s13287-019-1148-6 (PMC6357492; doi:10.1186/s13287-019-1148-6)
Supplement: Supplementary file 5 — Figure S2. A Original non-edited western blotting bands performed by Protein Simple Western of Fig. 1j in this article. B(a) Original non-edited western blotting bands performed by Protein Simple Western of Fig. 3a in this article. B(b) Original non-edited western blotting bands performed by Protein Simple Western of Fig. 3b in this article. C Original non-edited western blotting bands performed by Protein Simple Western of Fig. 4 in this article. D(a, b, c) Original non-edited western blotting bands performed by Protein Simple Western of Fig. 5b/C/E in this article. E Original non-edited western blotting bands performed by Protein Simple Western of Fig. 7d in this article. (PDF 706 kb) [file 13287_2019_1148_MOESM5_ESM.pdf]

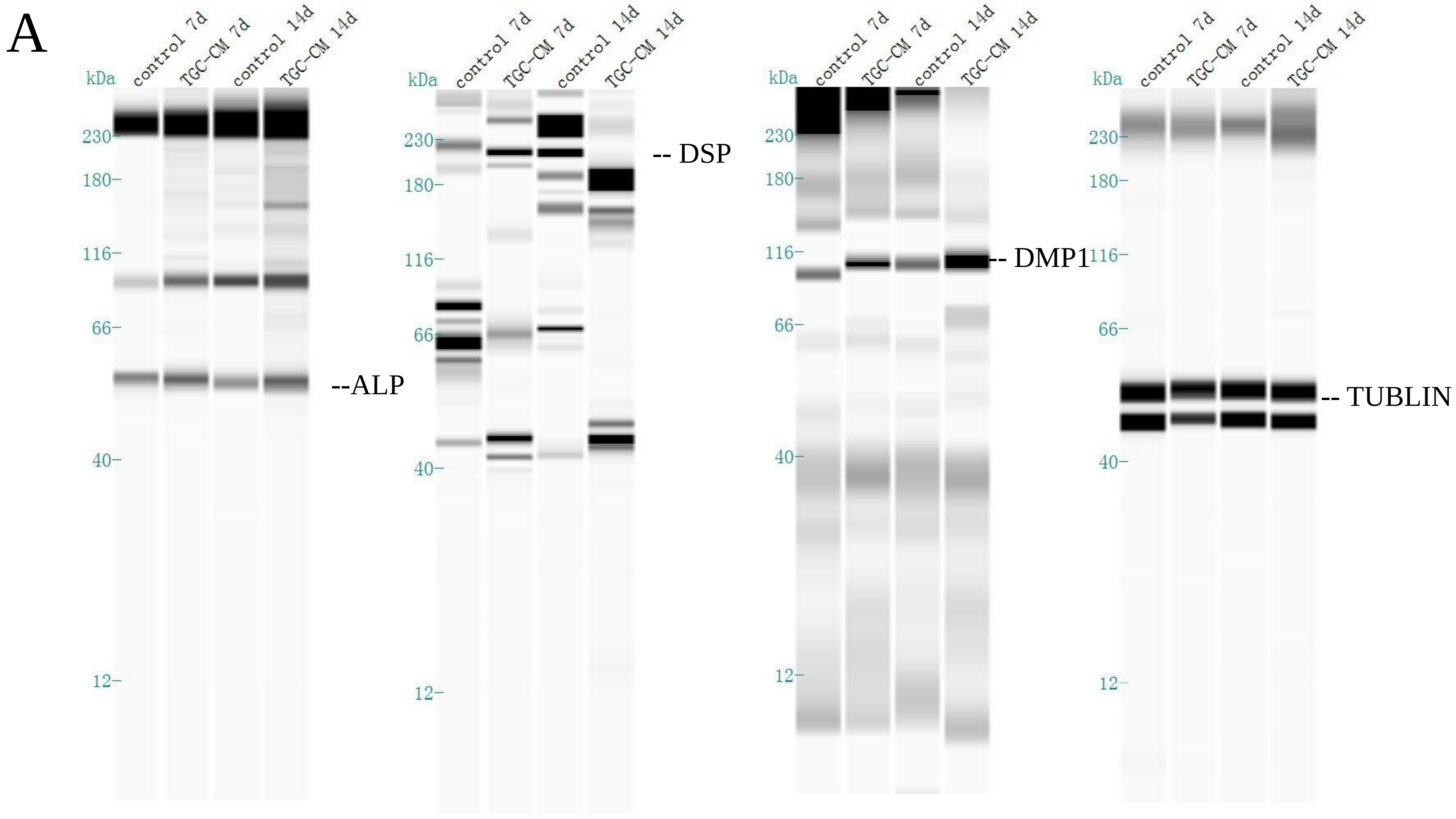

Figure S2 A. original non-edited western blotting bands performed by Protein Simple Western of Figure 1J in this article.

B(a)

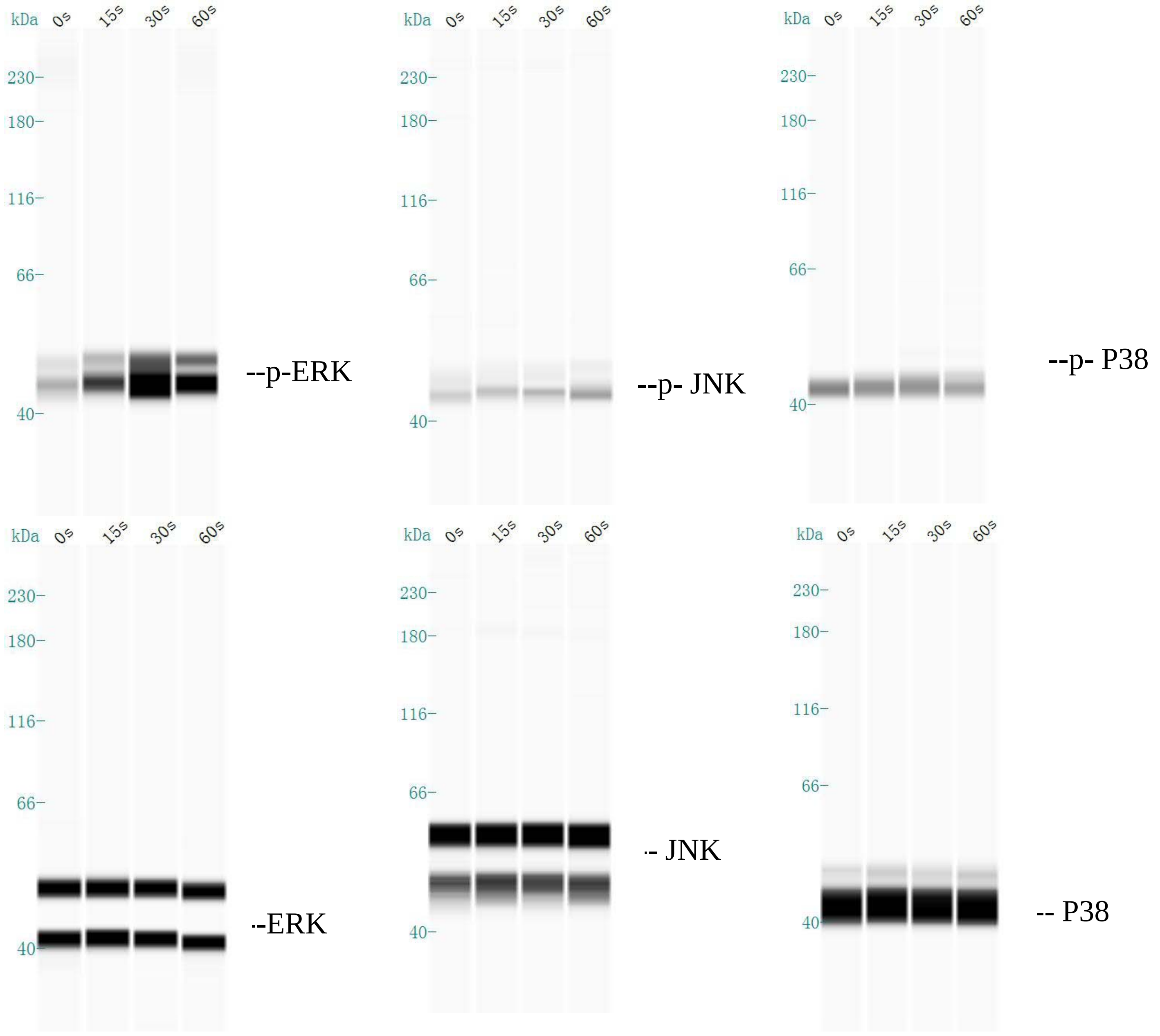

Figure S2 B(a). original non-edited western blotting bands performed by Protein Simple Western of Figure 3A in this article.

B(b)

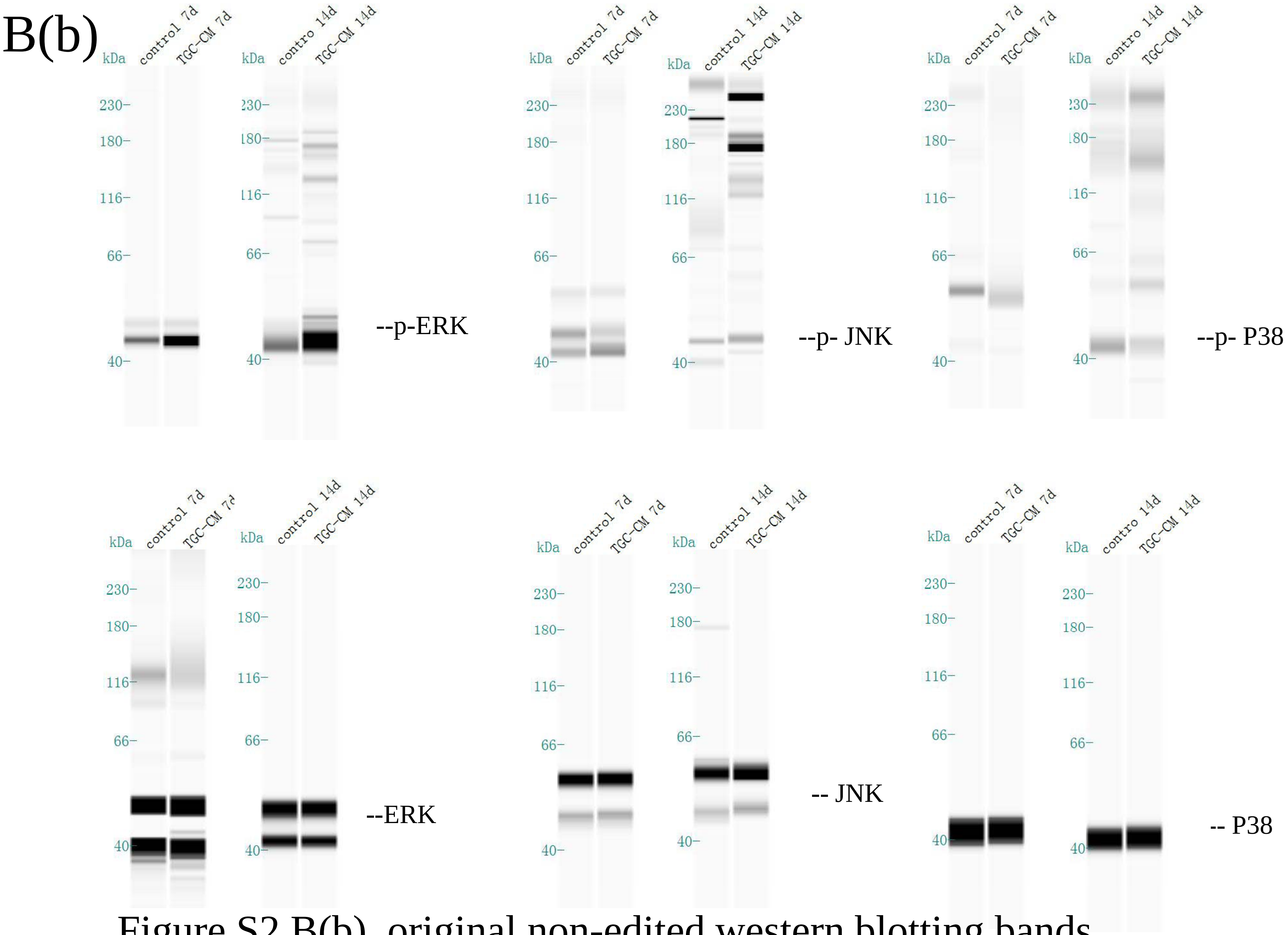

Figure S2 B(b). original non-edited western blotting bands performed by Protein Simple Western of Figure 3B in this article.

C

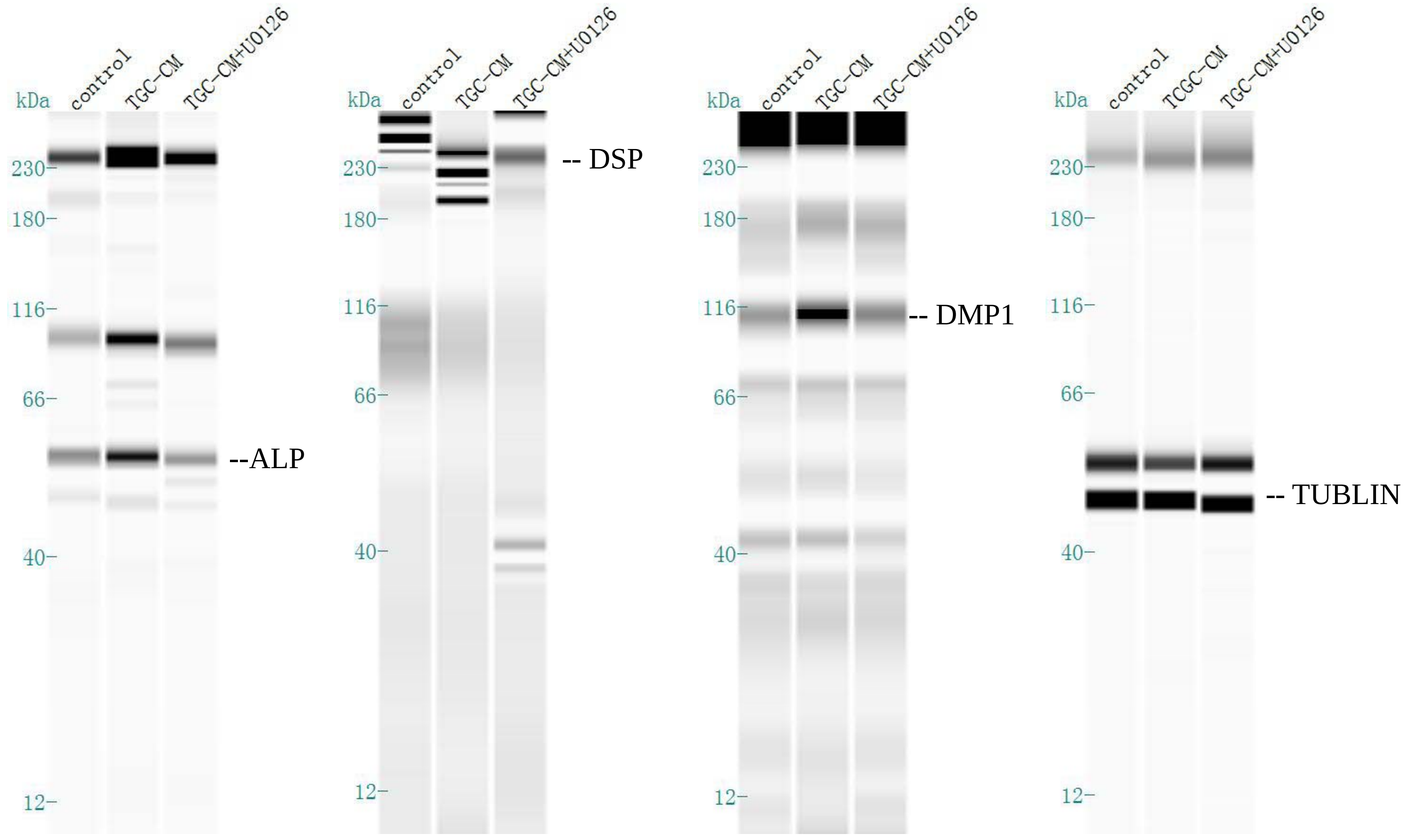

Figure S2 C. original non-edited western blotting bands performed by Protein Simple Western of Figure 4 in this article.

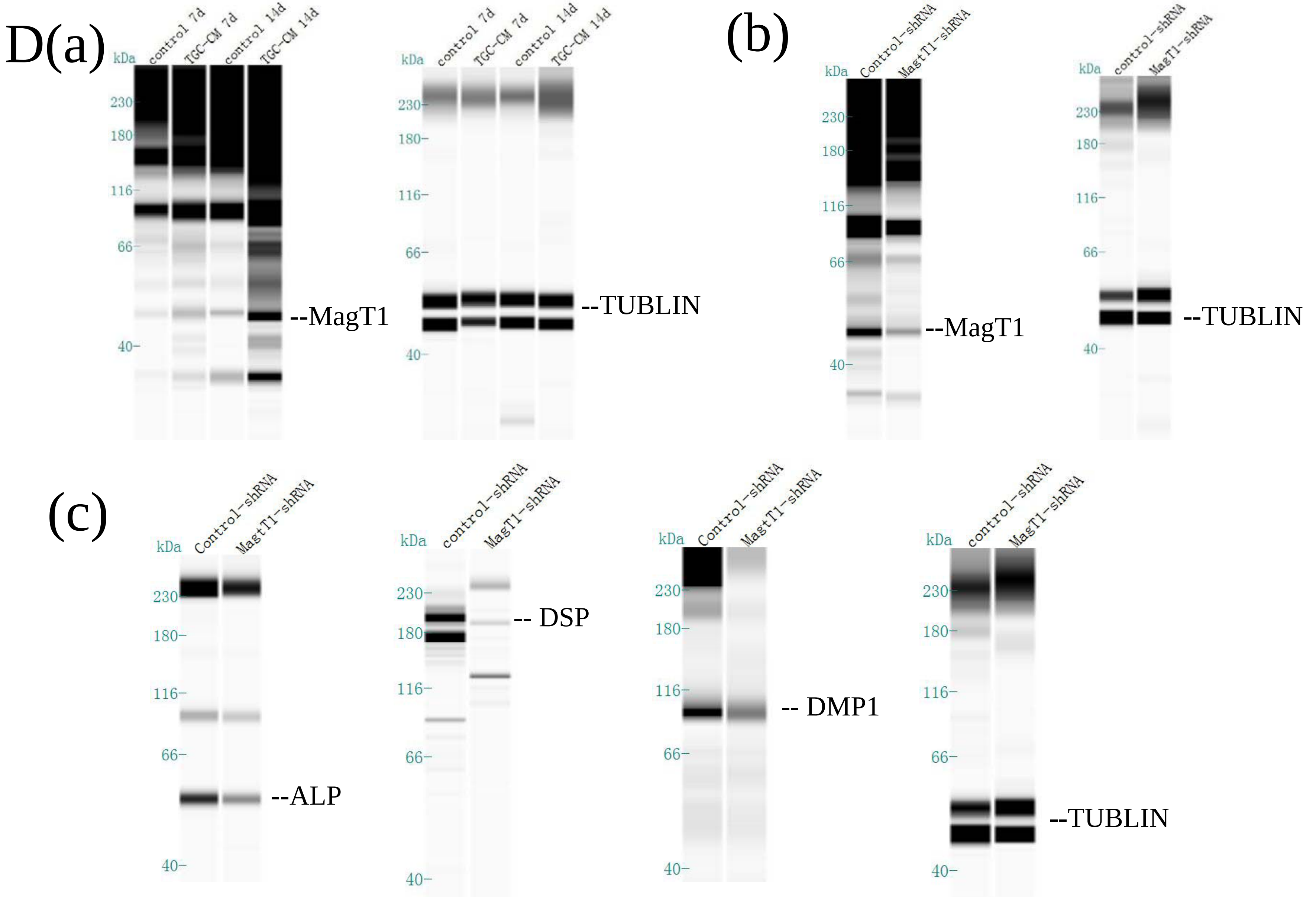

Figure S2 D(a, b, c). original non-edited western blotting bands performed by Protein Simple Western of Figure 5B/C/E in this article.

E

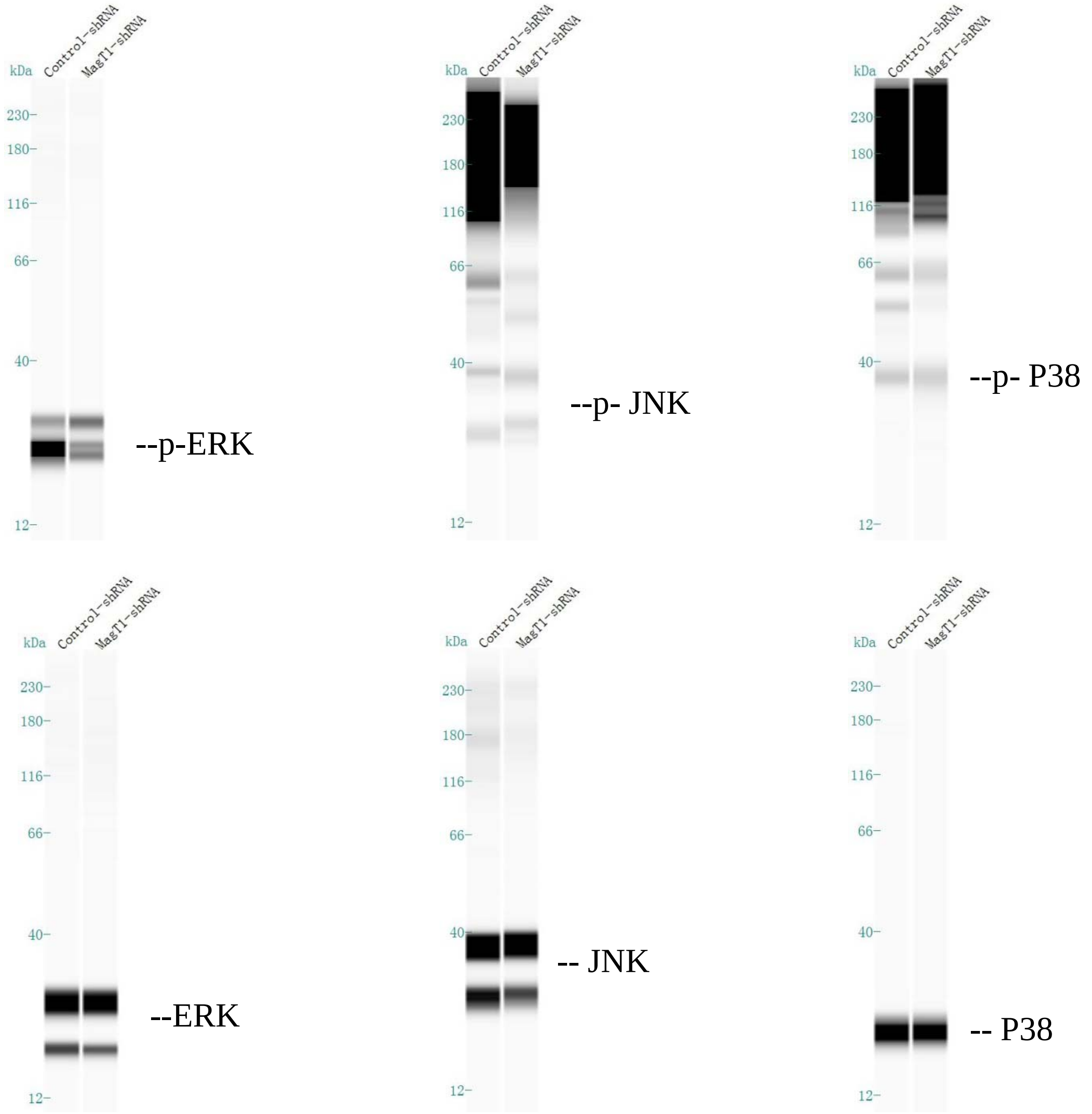

Figure S2 E. original non-edited western blotting bands performed by Protein Simple Western of Figure 7D in this article.
